# Synthesis and Insecticidal Evaluation of Chiral Neonicotinoids Analogs: The Laurel Wilt Case

**DOI:** 10.3390/molecules26144225

**Published:** 2021-07-12

**Authors:** Saúl A. Luna-Hernández, Israel Bonilla-Landa, Alfonso Reyes-Luna, Alfredo Rodríguez-Hernández, Ulises Cuapio-Muñoz, Luis A. Ibarra-Juárez, Gabriel Suarez-Mendez, Felipe Barrera-Méndez, Irving D. Pérez-Landa, Francisco J. Enríquez-Medrano, Ramón E. Díaz de León-Gómez, José L. Olivares-Romero

**Affiliations:** 1 Instituto de Ecología, A.C., Red de Estudios Moleculares Avanzados, Clúster Científico y Tecnológico BioMimic®, Campus III, Carretera Antigua a Coatepec No. 351, Xalapa 91073, Mexico; c7h5n3o6.lh@gmail.com (S.A.L.-H.); israel.bonilla@inecol.mx (I.B.-L.); alfonsoreyeslunauv@gmail.com (A.R.-L.); fredor2236@gmail.com (A.R.-H.); ulisescuapio@gmail.com (U.C.-M.); luis.ibarra@inecol.mx (L.A.I.-J.); investigacion.suarez@hotmail.com (G.S.-M.); felipe.barrera@inecol.mx (F.B.-M.); david.perez@posgrado.ecologia.edu.mx (I.D.P.-L.); 2 Cátedra CONACyT en el Instituto de Ecología, A.C. Carretera Antigua a Coatepec No. 351, Xalapa 91073, Mexico; 3 Centro de Investigación en Química Aplicada, Blvd. Enrique Reyna, No. 140, Saltillo 25294, Mexico; javier.enriquez@ciqa.edu.mx (F.J.E.-M.); ramon.diazdeleon@ciqa.edu.mx (R.E.D.d.L.-G.)

**Keywords:** neonicotinoids, nitroguanidines, chiral, enantiopure compound, insecticidal activity

## Abstract

*Xyleborus* sp beetles are types of ambrosia beetles invasive to the United States and recently also to Mexico. The beetle can carry a fungus responsible for the Laurel Wilt, a vascular lethal disease that can host over 300 tree species, including redbay and avocado. This problem has a great economic and environmental impact. Indeed, synthetic chemists have recently attempted to develop new neonicotinoids. This is also due to severe drug resistance to “classic” insecticides. In this research, a series of neonicotinoids analogs were synthesized, characterized, and evaluated against *Xyleborus* sp. Most of the target compounds showed good to excellent insecticidal activity. Generally, the cyclic compounds also showed better activity in comparison with open-chain compounds. Compounds *R*-**13**, **23**, *S*-**29**, and **43** showed a mortality percent of up to 73% after 12 h of exposure. These results highlight the enantioenriched compounds with absolute *R* configuration. The docking results correlated with experimental data which showed both cation-π interactions in relation to the aromatic ring and hydrogen bonds between the search cavity 3C79 and the novel molecules. The results suggest that these sorts of interactions are responsible for high insecticidal activity.

## 1. Introduction

The redbay ambrosia beetle, *Xyleborus glabratus*, is the vector of the laurel wilt disease fungal pathogen, *Raffaellea lauricola*. Since the vector’s initial detection in the USA in the early 2000s, laurel wilt has killed millions of redbay, *Persea borbonia*, trees, and other members of the plant family Lauraceae [1]. In this context, avocado (*Persea Americana* Mill.) is the most important agricultural crop susceptible to laurel wilt [2]. As the disease continues to move south and west from its original focus, it has caused significant concern in Florida, California, and other avocado-producing areas such as western Mexico. In the absence of effective control measures, monetary losses caused by laurel wilt could eventually range up to USD 54 million in the USA, and greater losses might occur if the disease moves elsewhere [3].

**Figure 1:**
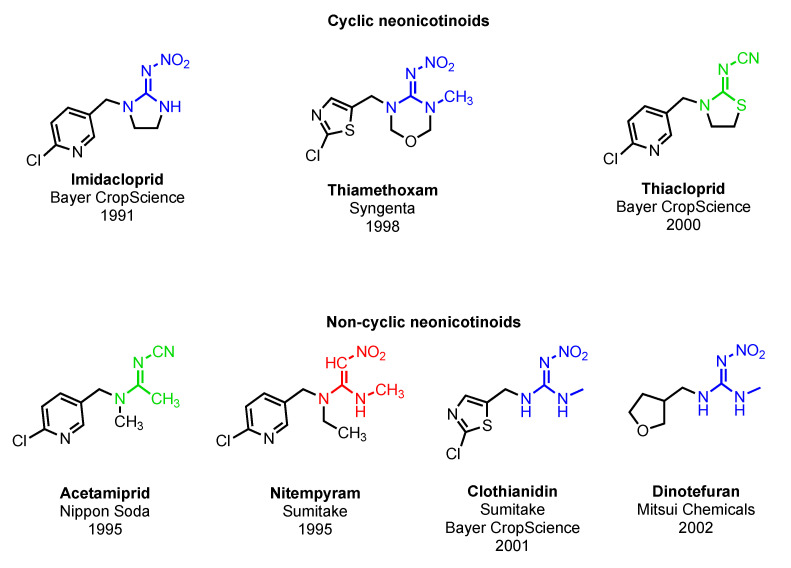
Neonicotinoids.

To the best of our knowledge, management is currently focused on monitoring, sanitation and direct control using contact or systemic insecticides [4], and these are still subject to ongoing investigations [5]. Indeed, efficacious and cost-effective measures are urgently needed to protect avocado from laurel wilt. In this context, neonicotinoids are an important type of compound with potent insecticidal activity. Since their introduction in the 1980s, these compounds have been established as the insecticides of choice for agricultural, animal health, and public health usages [6,7]. They are a type of insecticide that act selectively on the central nervous system of the insect and can be efficient ligands for the nicotinic acetylcholine receptors (nAChRs) of insects [8]. However, with the increase in neonicotinoids used for crop protection over a long time period, the problems of cross-resistance [9], and bee toxicity [10] have received more attention, and this calls for a new strategy of molecular design to find new leading compounds. Neonicotinoids act selectively on the insect central nervous system (CNS) as an agonist of the postsynaptic nicotinic acetylcholine receptors (nAChRs) [11,12,13]. The great attributes of neonicotinoids are their novel mode of action, low mammalian toxicity, broad insecticidal spectrum, and good systemic properties [14]. Another important property of neonicotinoids is their environmental footprint, which allows for the replacement of the more toxic and non-selective organophosphorus, pyrethroid, and carbamate insecticides. The reported neonicotinoids can be classified according to the pharmacophore as *N*-nitroguanidines, (imidacloprid, thiamethoxam, clothianidin, and dinotefuran), nitromethylenes (nitenpyram), and *N*-cyano-amidines (acetamiprid and thiacloprid) (Figure 1).

All these compounds are characterized by their high insecticidal activities against insects and relative safety toward mammals and aquatic life [15]. Encouraged by these reports, we envisaged that incorporating different substituents into dinotefuran by switching the heterocyclic ring with different chiral enantioenriched and non-chiral amines could improve their insecticidal activity. Therefore, in a search for improvement, neonicotinoids with enantiomers of chiral amines were designed, synthesized, and evaluated.

## 2. Results and Discussion

### 2.1. Synthesis

**Scheme 1:**
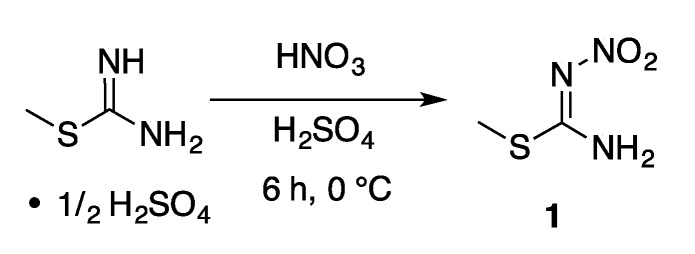
Preparation of starting material **1**.

The starting material one was prepared using 1 equivalent of *S*-methylisothiourea hemisulfate salt in the presence of nitric (10 mL) and sulfuric acids (10 mL) at 0 °C for 6 h (Scheme 1). Then, compound one was used to prepare the non-cyclic nitroguanidines **2**–**17**. After dissolving the corresponding amine in chloroform, compound one was added in one portion. Most of the target compounds were purified through flash column chromatography in yields of up to 95%.

**Scheme 2:**
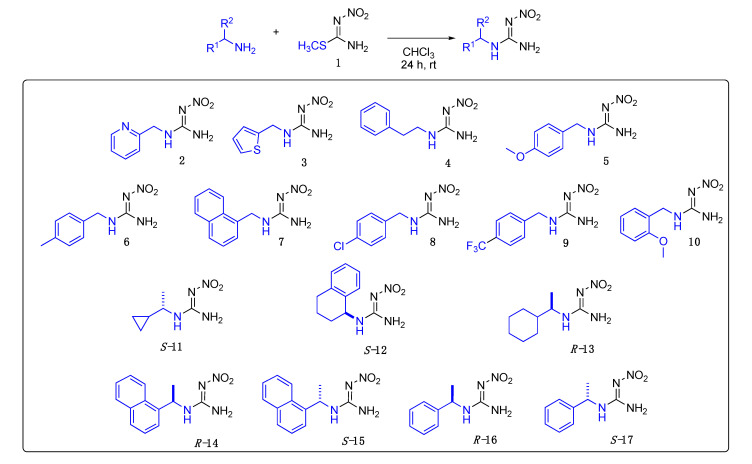
Synthesis of non-cyclic nitroguanidines.

Sixteen non-cyclic nitroguanidines were prepared and these produced different substitution patterns. For instance, nitroguanidines **2** and **3** had heterocyclic rings while **4** to **10** had an aromatic moiety with electron-donating and withdrawing substituents. Enantiopure non-cyclic nitroguanidines were also prepared using different sorts of chiral amines such as *R*- and *S*-phenylethylamine, and napthylethylamine, *S*-cyclopropylethylamine, and *R*-cyclohexylethylamine (Scheme 2). All these reactions proceeded through nucleophilic substitution: the lone pair of electrons on the amine attacked the sp^2^ carbon of compound one, then the -SCH_3_ moiety was displaced to obtain the desired nitroguanidine.

**Scheme 3:**
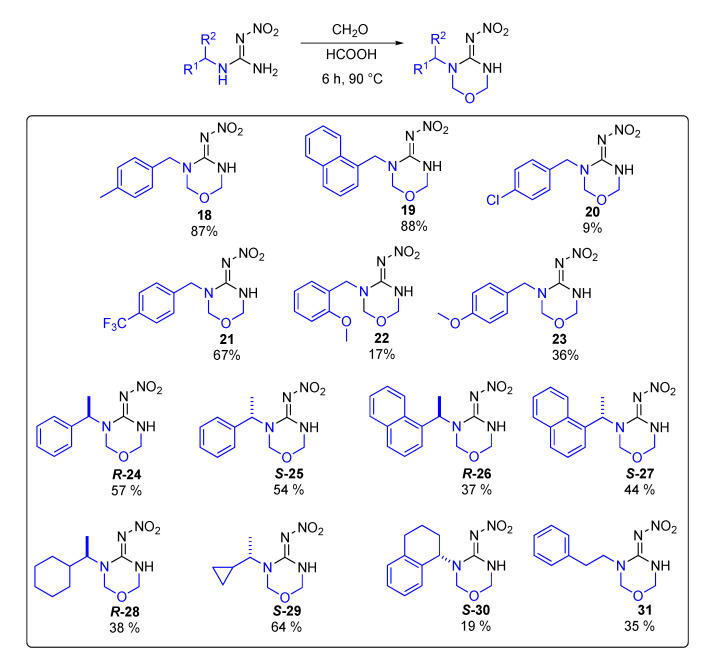
Synthesis of cyclic compounds.

Scheme 3 shows that cyclic nitroguanidines were prepared using 6 mL of the solution of formaldehyde and formic acid (1:1) and the corresponding nitroguanidine. The mixture was heated at 90 °C for 6 h. Most of the compounds were obtained from moderate to good yields.

**Scheme 4:**
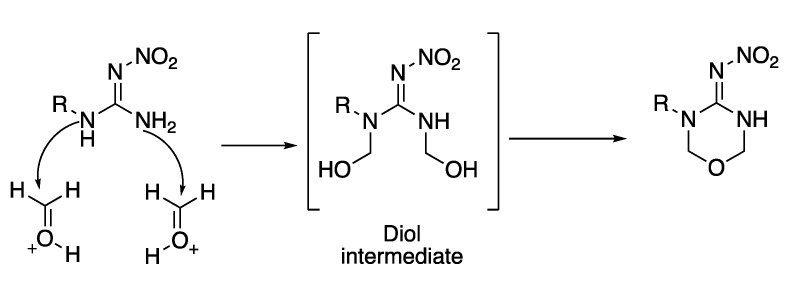
Plausible mechanism reaction.

The reaction proceeded through the Mannich-type reaction; here, the acid was used as a catalyst to promote the reaction where the key intermediate was the diol, which was then cyclized to obtain the corresponding cyclic nitroguanidine (Scheme 4) [16].

**Scheme 5:**
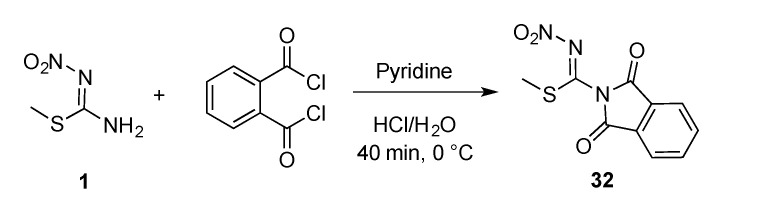
Synthesis of compound **32**.

**Scheme 6:**
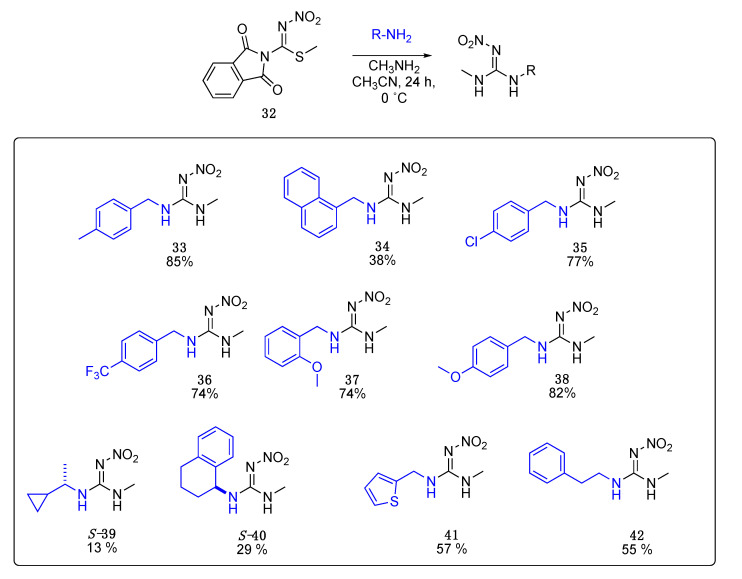
Synthesis of *N*-methylated compounds.

To obtain the *N*-methylated compounds **33**–**42**, compound **32** was synthesized using phthaloyl chloride, pyridine, and a solution of HCl/H_2_O. The resulting precipitate was filtered and recrystallized from EtOH to obtain the heterocyclic compound (Scheme 5). Afterwards, a stirred solution of **32** in acetonitrile was added dropwise to a solution of the corresponding amine at 0 °C. Then, the reaction was stirred for 30 min at the same temperature and a 2.0 M solution of methylamine in MeOH was immediately added. Most of the compounds were obtained with good yields (Scheme 6). It is important to point out that the reactions were carried out in one-pot. The addition of the amine and the addition of the methylamine were nucleophilic substitutions that took place at the sp^2^ carbon. 

**Scheme 7:**
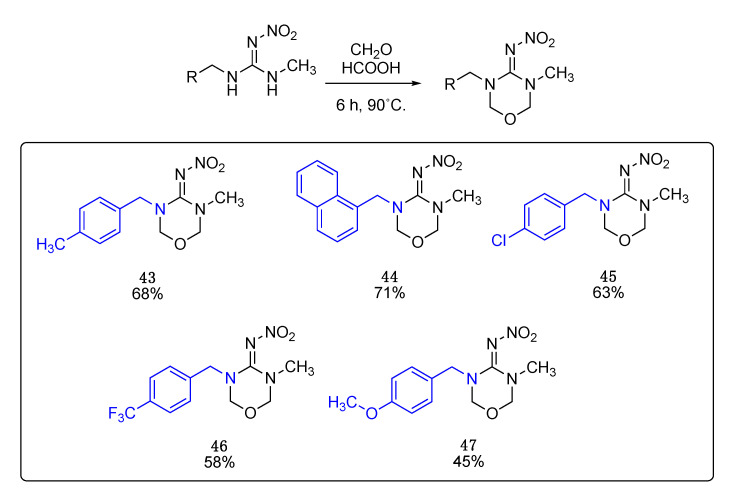
*N*-methylated cyclic nitroguanidines.

To synthesize compounds **43** to **47,** the same procedure described for compounds **18** to *R*-**31** was followed. Again, these reactions proceeded through a Mannich-type reaction. The five products were obtained in yields of up to 71% (Scheme 7). 

### 2.2. Biological Activities

**Figure 2:**
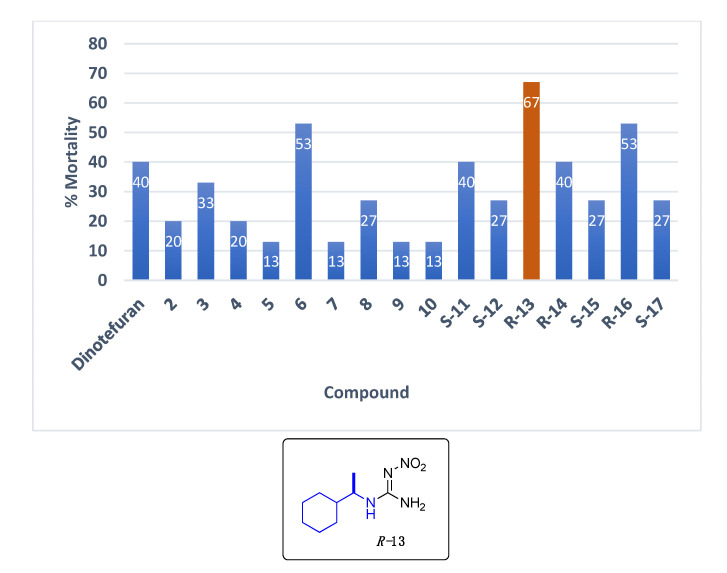
Analysis of non-cyclic nitroguanidines.

The insecticidal assays of the title compounds against *Xyleborus affinis* were tested using the filter paper contact toxicity method. The results were graphed for comparison. Most of the non-cyclic compounds showed a lower insecticidal activity than dinotefuran and the chiral and enantiopure compounds such as (*R,E*)-1-(cyclohexylethyl)-2-nitroguanidine *R*-**13**, which showed up to 67% mortality (Figure 2).

**Figure 3:**
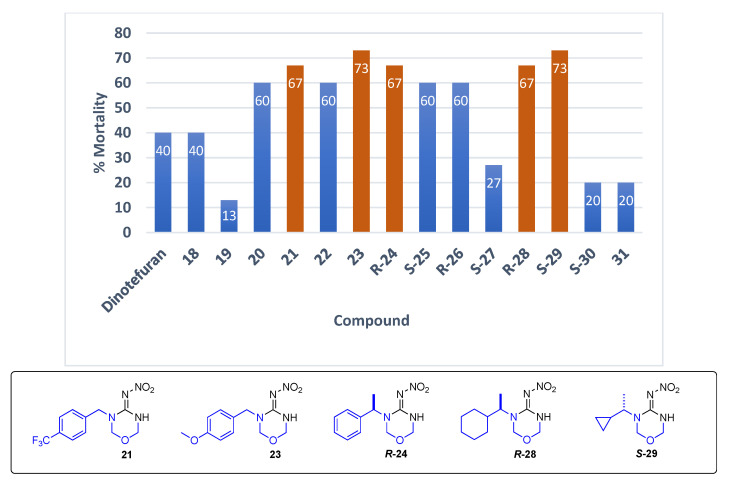
Analysis of cyclic nitroguanidines.

The cyclic nitroguanidines **21**, **23**, *R*-**24**, *R*-**28**, and *R*-**29** showed insecticidal activities of up to 73%. The comparison between nitroguanidines **21** and **23** suggest that compounds with an electron-donating group in the *para* position could have a higher mortality percent than compounds that have electron-withdrawing groups. It was also observed that enantiopure aliphatic nitroguanidines, *R*-**28** and *S*-**29**, have a good insecticidal activity up to 73% (Figure 3).

**Figure 4:**
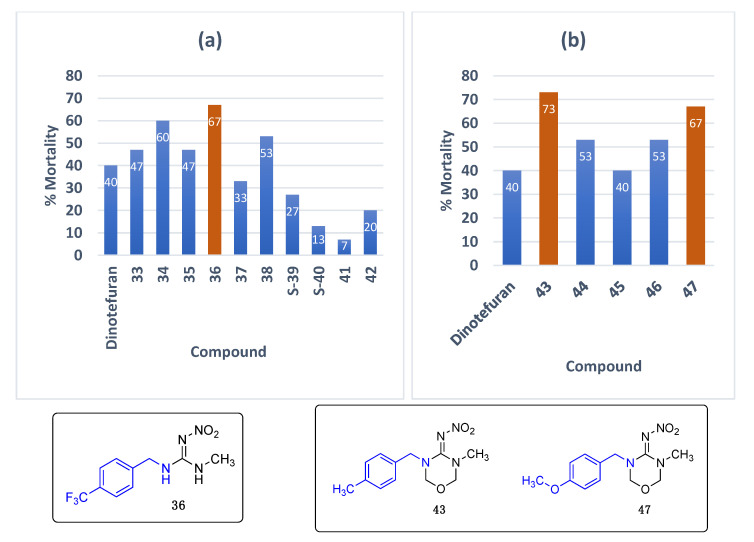
Analysis of (**a**) non-cyclic nitroguanidines; (**b**) cyclic and *N*-methylated nitroguanidines.

The analysis of non-cyclic and cyclic *N*-methylated nitroguanidines allows us to highlight the compounds **36**, **43**, and **47**. The structure–activity relationship suggests again that compounds with electron-donating or electron-withdrawing groups at the *para* position of the aromatic ring have higher insecticidal activities (Figure 4).

### 2.3. Structure–Activity Relationship (SAR) of the Para Halogenated Aromatic Derivatives

**Figure 5:**
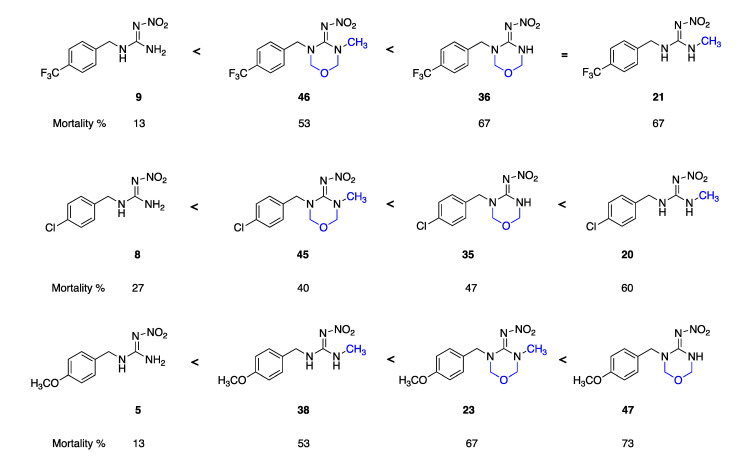
Structure–activity analysis of new neonicotinoids.

The SAR analysis showed that non-cyclic and *N*-methylated compounds had a higher insecticidal activity. On the other hand, compounds that incorporated the *para* electron-withdrawing group attached to the aromatic ring, such as CF_3_ or Cl, had the same tendency in terms of insecticidal activity. As a matter of fact, the presence of a halogen at a specific position in the molecule can influence its biological activity [17]. The *N*-methylated compounds have a better insecticidal activity than non-methylated compounds (compound **21** and **8** vs. compound **9** and **20**). In contrast to our results with neonicotinoids, Xu, et al. [18] designed and synthesized a series of 1,5-diphenyl-2-penten-1-one analogues with piperazine and evaluated their bioactivities. The SAR analysis showed that the *R* ring plays a crucial role, and that the antifungal activities and larvicide against mosquitoes of the compounds with *N*’-unsubstituted piperazine were better than those of the compounds with *N*’-methyl piperazine. Likewise, its larvicidal activity was severely decreased when the substituent on the benzene ring was changed from position four to position two or three. However, the improved biopotential of the *N*-methylated compounds studied in our work is in agreement with Dahiya et al. [19], who synthesized an *N*-methylated analog of a proline-rich cyclic tetracyclopeptide from marine bacteria with enhanced anthelmintic and antifungal activity, compared to the non-methylated tetracyclopeptide. Cyclic compounds showed up to 67% mortality for compound **36** and 47% mortality for compound **35** (Figure 5).

**Figure 6:**
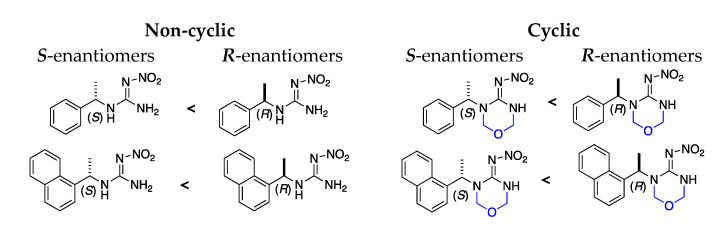
Absolute configuration–activity relationship (ACAR) of chiral nitroguanidines.

It is well known that chirality plays an important role when bioactive enantiopure compounds are administered to living organisms [20]. As a part of our research program in relation to biologically active and enantioenriched compounds, we prepared both the enantiomers of nitroguanidines derivatives that had the phenylethylamine or the naphtyletylamine in enantiopure form. Those compounds were bioassayed and they showed interesting differences in their insecticidal activity. For instance, the non-cyclic *R* enantiomers showed a higher insecticidal activity. Moreover, the cyclic enantiomers with an absolute *R* configuration showed a higher insecticidal activity when compared with the *S* enantiomers (Figure 6). The favored insecticidal activity of the *R* enantiomers has previously been reported, for instance, in limonene against *Tribolium confusum* [21], in cycloprothrins against the larvae of *Tetranychus cinnabarnus, Nilaparvatalegen, Mythimaseparata*, and *Aphismedicagini* [22], and also in pyridine methanesulfonates against *Diabrotica undecimpunctata howardi* and *Nephotettix cincticeps* [23].

### 2.4. Docking Analysis

To perform the docking analysis, the highly bioactive compounds **47**, *R*-**13**, **43**, **23**, **36**, and *S*-**29** were selected. First, the score of dinotefuran was obtained since this was our model insecticide. The score was −5.64 kcal/mol. The lower the score, the better the affinity with the receptor in the search cavity of 3C79. The compound with an outstanding affinity was the cyclic *N*-methylated compound **47** which has a *p*-methoxy-substituted aromatic ring. This compound was followed by the enantiopure compound *R*-**13** that possessed an absolute *R* configuration. The results suggest that the non-methylated **23** and non-cyclic **36** compounds presented a diminished affinity. The compound with an absolute *R* configuration had a better affinity when compared with the *S* compound. All the docking calculations agreed with the experimental results. Another interesting observation derived from docking was that the inhibitions constants (K*_i_*) were lower for compounds **47**, *R*-**13**, and **43**. For instance, the comparison between dinotefuran, *R*-**13**, and *S*-**29**, showed that these values were 24.23, 73.06, 263.75 μM, respectively. These results suggest again that compounds with absolute *R* configurations are more potent and improved inhibitors since the concentration to produce the half-maximum inhibition could be reduced by up to 33% in comparison with dinotefuran. The comparison of the K*_i_* between compounds with opposite absolute configurations of *R*-**13** and *S*-**29** showed a huge difference since these values were 24.23 and 263.75, respectively (Table 1). The experimental results and the docking results suggest again that compounds with an *R* configuration could have a highly insecticidal activity at lesser doses. Additionally, the in-silico calculations suggest that cyclic *N*-methylated compounds **47** and **43** are highly active insecticides against *Xyleborus affinis*.

**Figure 7:**
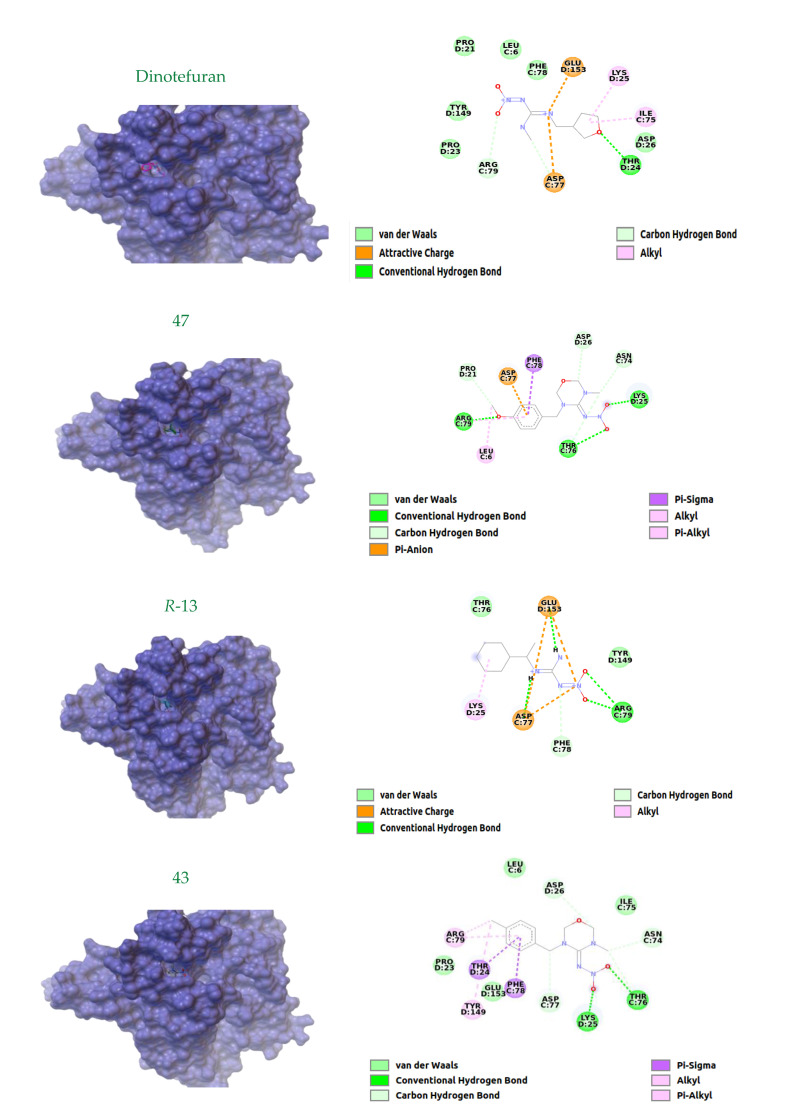
The 2D and 3D representations of the interaction of dinotefuran and compounds **47**, *R*-**13**, and **43**, with the residues of the search cavity in 3C79.

The 2D and 3D representations of the molecular recognition with the residues of the search cavity of the binding protein 3C79 (AChBP) with dinotefuran and the compounds selected to perform the docking showed different types of interactions among them (Figure 7). For instance, dinotefuran showed interactions between the nitrogen attached to a heterocyclic moiety and the receptor in Glu153 and Asp77. An interaction between the oxygen of the heterocyclic ring and the Thr24 of the receptor was also observed. On the other hand, a cation-π interaction between the aromatic ring of compounds **47** and **43** and Phe78 was found. These sorts of interactions play an important role in neonicotinoid insecticidal activity [24]. The enantiopure compound **13** showed interactions with Glu153 and As77; in comparison with dinotefuran, these interactions appear to be due to hydrogen bonds and the results suggest that these sorts of interactions are responsible for the higher insecticidal activity. These results also suggest that this strategy could result in a useful tool to fight cross-resistance, since the nature of the interactions between the novel molecules and the receptor are quite different. Examples of intragroup cross-resistance are well documented for most major insecticide groups, including pyrethroids, organophosphate, and neonicotinoids [25,26,27]. However, cross-resistance can also be unpredictable, affecting compounds with diverse structures and those that are considered to have different modes of action. 

## 3. Materials and Methods

### 3.1. Instrumentation and Chemicals

All reactions were carried out in flame-dried glassware with a nitrogen atmosphere and magnetic stirring unless otherwise noted. Analytical thin-layer chromatography (TLC) was performed on Merck pre-coated TLC plates (silica gel 60 GF254, 0.25 mm). Flash chromatography was performed on silica gel E. Merck 9385 or silica gel 60 extra pure (for final products). Dichloromethane (CH_2_Cl_2_) and toluene (PhCH_3_) were purchased from Sigma-Aldrich and were purified by distillation. Most of the chemicals were purchased from Aldrich, Stream, or Alfa Aesar and handled and stored in a desiccator. Molecular sieves were purchased from Sigma-Aldrich and activated with a microwave oven. All other reagents and starting materials, unless otherwise noted, were purchased from commercial vendors and used without further purification. Infrared (IR) spectra were recorded on an Agilent Technologies Cary 600 Series FTIR. ^1^H, ^13^C, DEPTQ 135, and bidimensional NMR spectra were recorded on a Bruker Avance III HD 500 spectrometer (Billerica, MA, USA). Chemical shift values (δ) are expressed in ppm downfield relative to an internal standard (tetramethylsilane at 0 ppm). Multiplicities are indicated as s (single), d (doublet), t (triplet), q (quartet), m (multiplet), and br s (broad signal). The solvent CDCl_3_ at 77.16, DMSO-*d*_6_ at 39.52, and CD_3_OD at 49 ppm were used as internal standards for ^13^C NMR spectra. Spectra were processed using TopSpin 4.0.7 from Bruker BioSpin © (Billerica, MA, USA). The ^1^H and ^13^C NMR spectra for all compounds can be found in the Supplementary Materials. Optical rotations were measured on a Bellingham + Stanley ADP 440 + polarimeter (Kent, UK). High-resolution mass spectra (HRMS) were obtained in a Q-TOF mass spectrometer equipped with electrospray ionization (ESI) interface Synapt G2-Si, Waters Inc. (Milford, MA, USA). Melting points were not corrected and determined on a Stuart SMP10 apparatus (Staffordshire, UK) using open glass capillaries. 

### 3.2. Synthetic Procedures

Unless otherwise noted, reagents and solvents were used as received from commercial suppliers. Yields were not optimized. The synthetic procedures have already been described in the literature and were followed to prepare the novel neonicotinoids. 

#### 3.2.1. Synthetic Procedure to Obtain the Methyl *N*-Carbamimidothioate **1**

Concentrated sulfuric acid (10 mL) was placed in a balloon flask equipped with a stirring bar and a thermometer and it was cooled with an ice bath to 0 °C. When this temperature was reached, 70% nitric acid was added (10 mL). The cooling was maintained until the initial temperature of 0 °C was reached, and then 1 equivalent of *S*-methylisothiourea hemisulfate was added. The mixture was stirred for 6 h at 0 °C and once the reaction time had elapsed, the contents of the flask were poured over a beaker with ice to obtain a precipitate. This mixture was kept under refrigeration for 24 h to melt the remaining ice. The isolation of the product was achieved by filtration under reduced pressure and washing of the solid with cold distilled water. Finally, the solid was dried under a high vacuum to obtain **1** [28].

#### 3.2.2. General Synthetic Procedure for Non-Cyclic Compounds **2**–**17**

To a stirred solution of the corresponding amine (1.1 mmol) in chloroform (1 mL) compound **1** was added (1.0 mmol) in one portion, after that the reaction was stirred for 24 h at room temperature. The solvent was evaporated under reduced pressure and the crude was purified using flash column chromatography [29]. See Data Compounds in Appendix A.

#### 3.2.3. General Synthetic Procedure for Cyclic Compounds **18**–**31**

In a solution of formaldehyde (37%) and formic acid (1:1, 6 mL) the corresponding nitroguanidine (1 mmol) was suspended. The mixture was heated at 90 °C for 6 h. The reaction was extracted using AcOEt (3 × 10 mL). The organic phases were collected, filtered over Na_2_SO_4_, and evaporated. The crude was purified using flash column chromatography [16]. See Data Compounds in Appendix B. 

#### 3.2.4. Synthetic Procedure to Obtain the Intermediate **32**

In a flask equipped with a stir bar, compound **1** (1 mmol) was dissolved in pyridine (1 mL) and cooled in an ice-water bath to 0 °C. Subsequently, 90% phthaloyl chloride solution (2 mmol) was added dropwise for 30 min. After the addition was completed, the reaction was stirred for an additional 10 min and at the end of that time, the mixture was poured over a 1:5 HCl/H_2_O solution (20 mL). The resulting precipitate was filtered under reduced pressure, recrystallized in EtOH, and dried under a high vacuum to obtain **32**.

#### 3.2.5. General Synthetic Procedure to Obtain *N*-Methylated Compounds 

To a stirred solution of **32** (1 mmol) in acetonitrile (2 mL) a solution of the corresponding amine (1.1 mmol) in acetonitrile (1.0 mL) was added over 20 min at 0 °C. The reaction was stirred for 30 min at the same temperature and a 2.0 M solution of methylamine in MeOH (3.0 mmol) was added immediately. The mixture was stirred for 3 h at room temperature. The solvent was evaporated under reduced pressure and the crude was purified using flash column chromatography [29]. See Data Compounds in Appendix C.

### 3.3. Biological Assay

The insecticidal activities of the title compounds against *Xyleborus affinis* were tested according to the previously reported procedure of the contact toxicity on filter paper [30]: A Whatman^TM^ (Buckinghamshire, UK) grade 42, ashless filter paper (5 cm diameter) was placed in a glass Petri dish (50 × 17 mm diameter). An aliquot of 0.25 mL of the solution 0.05 M of the compound in dimethylsulfoxide:water (1:1) was applied uniformly to the filter paper disc. The mixture of solvents was used as the negative control and the solution of dinotefuran at the same concentration was used as the positive control. The solvent was allowed to distribute evenly for 5 min prior to the introduction of 5 adult insects into each dish. Since the boiling point of dimethyl sulfoxide and water are high enough to be volatilized at room temperature, it was not necessary to replenish the solvent mixture. According to statistical requirements, each treatment was replicated 3 times at 25 °C ± 1 °C with the organism grown in the laboratory. *Xyleborus affinis* were reared in an artificial media according to Biedermann et al. [31], with some modification. Rearing media were maintained in a climatic chamber at 26 °C and 60% of RH in complete dark. Adults were obtained at 30 days after female inoculation by dissecting the media culture. Insect mortalities were recorded after 12 h. Insects were presumed dead if they remained immobile and did not respond to three probings with a blunt dissecting probe after a 5-min recovery period. 

## 4. Conclusions

A series of novel neonicotinoid analogs were designed and synthesized by introducing aliphatic and aromatic amines. Interestingly, enantiopure amines with opposite absolute configurations were prepared and analyzed as insecticides against *Xyleborus affinis*. The results showed us that the amines with an absolute *R* configuration have a superior biological activity when compared to the opposite enantiomer *S*. Four lead compounds that had percent mortalities up to 73% were derived from this study, while dinotefuran had only a 40% mortality.

The docking studies suggested some interesting interactions between the molecules and the search cavity 3C79 of the receptor, which were cation-π interactions between the aromatic ring moiety of the molecules and hydrogen bonds. These interactions are likely to be the reason for the high insecticidal activity. Hence, this technology has emerged as a strategic tool to develop new chiral and enantiopure neonicotinoids with high insecticidal activity to fight against cross-resistance and protect important economical crops while reducing the ecotoxicity of pollinators [32].

## Figures and Tables

**Table 1:** Affinity and inhibition constants of highly bioactive compounds ^a^.

Compound	Free Energy (kcal/mol) “Score”	Inhibition Constant (K*_i_*, µM)
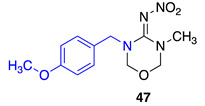	−6.53	16.29
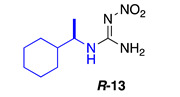	−6.3	24.23
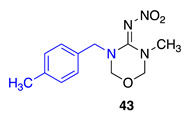	−6.08	35.22
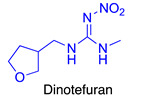	−5.64	73.06
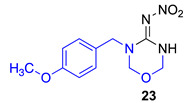	−5.47	98.40
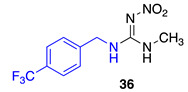	−5.19	157.1
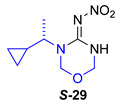	−4.88	263.75

^a^ To perform the simulations, the parameters of 100 runs with 5 million evaluations were used for each molecular coupling experiment using a Lamarckian genetic algorithm.

## Data Availability

Data are available from the authors on request.

## Supplementary Materials

The followings are available online. ^1^H and ^13^C NMR data for all compounds.

## Appendix A

*Data for 2-nitro-1-(pyridin-2-ylmethyl)guanidine* **2**. Gray solid, 82%, m.p. 175–177 °C (decomposes); TLC *R*_f_ 0.78 (MeOH); ^1^H NMR (500 MHz, DMSO-*d_6_*, 50 °C): δ 8.56 (ddd, *J* = 4.9, 1.8, 1.0 Hz, 1H), 7.93 (br s, 2H), 7.81 (td, *J* = 7.6, 1.8 Hz, 1H), 7.36–7.33 (m, 2H), 7.33–7.31 (m, 1H), 4.54 (s, 2H), ^13^C NMR (125 MHz, DMSO-*d*_6_, 50 °C): δ 149.4, 137.5, 123.0, 121.8, 46.0. HRMS (ESI): [M + Na]^+^ calculated for C_7_H_9_N_5_NaO_2_ [M + Na]^+^ 218.0648, found 218.0647 (5.0 ppm). IR (ATR) ν_max_: 3378, 3145, 3301, 1652.

*Data for 2-nitro-1-(thiophen-2-ylmethyl)guanidine***3**. White solid, 74% yield, m.p. 129–131 °C; TLC *R*_f_ 0.21 (AcOEt/Hexane, 1:1); ¹H NMR (500 MHz, DMSO-*d*_6_, 50 °C): δ 7.93 (br s, 2H), 7.43–7.42 (m, 1H), 7.07–7.05 (m, 1H), 7.01–6.98 (m, 1H), 4.58 (br s, 2H), ^13^C NMR (125 MHz, DMSO-*d*_6_, 50 °C): δ 159.0, 127.0, 126.8, 126.1, 125.6, 38.4. HRMS (ESI): calculated for C_6_H_9_N_4_O_2_S [M + H]^+^ 201.0441, found 201.0441 (5.0 ppm). IR (ATR) ν_max_: 3371, 3160, 3312, 1644.

*Data for 2-nitro-1-phenethylguanidine***4**. Yellow solid, 89% yield, m.p. 128–130 °C; TLC *R*_f_ 0.46 (AcOEt/Hexane, 6:4); ^1^H NMR (500 MHz, DMSO-*d*_6_, 50 °C): δ 7.35–7.29 (m, 2H), 7.29–7.20 (m, 3H), 3.48–3.40 (m, 2H), 2.83 (t, *J* = 7.4 Hz, 2H), ^13^C NMR (125 MHz, DMSO-*d*_6_, 50 °C): δ 159.7, 139.0, 129.2, 128.8, 126.8, 42.5, 34.4. HRMS (ESI): calculated for C_9_H_13_N_4_O_2_ [M + H]^+^ 209.1033, found 209.1030 (5.0 ppm). IR (ATR) ν_max_: 3367, 3170, 3302, 1644.

*Data for (E)-1-(4-methoxybenzyl)-2-nitroguanidine***5**. White solid, 91% yield, m.p. 192–194 °C; TLC *R*_f_ 0.19 (AcOEt/Hexane, 6:4); ^1^H NMR (500 MHz, DMSO-*d*_6_, 50 °C): δ 7.86 (br s, 2H), 7.26 (d, *J* = 8.6 Hz, 1H), 6.93 (d, *J* = 8.6 Hz, 1H), 4.34 (s, 2H), 3.75 (s, 3H), ^13^C NMR (125 MHz, DMSO-*d*_6_, 50 °C): δ 159.0, 129.1, 114.3, 55.5, 43.7. HRMS (ESI): calculated for C_9_H_13_N_4_0_3_ [M + H]^+^ 225.0982, found 225.0988 (−2.2 ppm). IR (ATR) ν_max_: 3382, 3322, 3166.522, 1648.

*Data for (E)-1-(4-methylbenzyl)-2-nitroguanidine***6**. White solid, 86% yield, m.p. 188–191 °C; TLC *R*_f_ 0.28 (AcOEt/Hexane, 6:4); ^1^H NMR (500 MHz, DMSO-*d*_6_, 50 °C): δ 7.87 (br s, 3H), 7.21 (d, *J* = 8.1 Hz, 2H), 7.17 (d, *J* = 8.1 Hz, 2H), 4.37 (br s, 2H), 2.30 (s, 3H), ^13^C NMR (125 MHz, DMSO-*d*_6_, 50 °C): δ 159.8, 136.9, 129.4, 127.7, 44.1, 21.0. HRMS (ESI): [M + H]^+^ calculated for C_9_H_13_N_4_O_2_ [M + H]^+^ 209.1033, found 209.1039 (−3.8 ppm). IR (ATR) ν_max_: 3374, 3305, 3155, 1648.

*Data for (E)-1-(naphthalen-1-ylmethyl)-2-nitroguanidine***7**. White solid, 73% yield, m.p. 189–192 °C; TLC *R*_f_ 0.34 (AcOEt/Hexane, 6:4); ^1^H NMR (500 MHz, DMSO-*d*_6_, 50 °C): δ 8.05 (d, *J* = 8.2 Hz, 1H), 7.97 (d, *J* = 8.0 Hz, 1H), 7.89 (d, *J* = 8.0 Hz, 1H), 7.63–7.54 (m, 2H), 7.54–7.45 (m, 2H), 4.89 (s, 2H), ^13^C NMR (125 MHz, DMSO-*d*_6_, 50 °C): δ 160.0, 133.8, 131.1, 129.0, 128.3, 126.8, 126.3, 125.8, 123.6, 42.6. HRMS (ESI): calculated for C_12_H_13_N_4_O_2_ [M + H]^+^ 245.1033 found 245.1039, (−2.9 ppm). IR (ATR) ν_max_: 3381, 3311, 3180, 1644.

*Data for (E)-1-(4-chlorobenzyl)-2-nitroguanidine***8**. White solid, 72% yield, m.p. 190–192 °C; TLC *R*_f_ 0.22 (AcOEt/Hexane, 6:4); ^1^H NMR (500 MHz, DMSO-*d*_6_): δ 7.95 (s, 2H), 7.46–7.38 (m, 3H), 7.33 (d, *J* = 8.5 Hz, 2H), 4.40 (s, 2H), ^13^C NMR (125 MHz, DMSO-*d*_6_): δ 159.9, 132.3, 129.5, 128.8, 43.6. HRMS (ESI): calculated for C_8_H_10_ClN_4_O_2_ [M + H]^+^ 229.0487, found 229.0492 (−3.9 ppm). IR (ATR) ν_max_: 3370, 3312, 3180, 1645.

*Data for (E)-2-nitro-1-(4-(trifluoromethyl)benzyl)guanidine***9**. White solid, 83% yield, m.p. 161–164 °C; TLC *R*_f_ 0.19 (AcOEt/Hexane, 6:4); ^1^H NMR (500 MHz, DMSO-*d*_6_, 50 °C): δ 7.97 (br s, 2H), 7.72 (d, *J* = 8.0 Hz, 2H), 7.52 (d, *J* = 7.9 Hz, 2H), 4.52 (s, 2H), ^13^C NMR (125 MHz, DMSO-*d*_6_, 50 °C): δ 159.8, 128.3, 128.1, 125.7 (q, J = 3.75 Hz), 125.3 (q, *J* = 270 Hz), 43.72; ^19^F NMR (470 MHz, DMSO-*d*_6_, 50 °C): δ −60.86. HRMS (ESI): calculated for C_9_H_10_F_3_N_4_O_2_ [M + H]^+^ 263.0750, found 263.0756 (−3.8 ppm). IR (ATR) ν_max_: 3378, 3305, 3178, 1648.

*Data for (E)-1-(2-methoxybenzyl)-2-nitroguanidine***10**. White solid, 85% yield, m.p. 203–205 °C; TLC *R*_f_ 0.25 (AcOEt/Hexane, 6:4); ¹H NMR (500 MHz, DMSO-*d*_6_): δ 7.82 (br s, 2H), 7.33–7.28 (m, 1H), 7.23 (dd, *J* = 1.0, 7.4 Hz, 1H), 7.04 (d, *J* = 8.2 Hz, 1H), 7.04 (d, *J* = 8.2 Hz, 1H), 6.95 (t, *J* = 7.4 Hz, 1H), 4.37 (br s, 2H), 3.84 (s, 3H), ^13^C NMR (125 MHz, DMSO-*d*_6_): δ 157.2, 129.3, 128.3, 120.7, 111.2, 55.8. HRMS (ESI): calculated for C_9_H_12_N_4_O_3_Na [M + Na]^+^ 247.0802, found 247.0807 (−1.6 ppm). IR (ATR) ν_max_: 3382, 3322, 3166, 1648.

*Data for (S)-1-(1-cyclopropylethyl)-2-nitroguanidine S*-**11**. White solid, 72%, m.p. 109–111 °C; TLC *R*_f_ 0.37 (AcOEt/Hexane, 7:3); [α]^26^ = +18.81 (*c* = 1.0, acetone); ¹H NMR (500 MHz, CDCl_3_): δ 7.78 (br s, 1H), 6.85 (br s, 2H), 3.10–3.06 (m, 1H), 1.38 (d, *J* = 6.5 Hz, 3H), 1.04–0.97 (m, 1H), 0.68–0.61 (m, 2H), 0.40–0.27 (m, 2H), ^13^C NMR (125 MHz, CDCl_3_): δ 158.6, 52.3, 20.0, 16.8, 3.8, 3.1. HRMS (ESI): calculated for C_6_H_13_N_4_O_2_ [M + H]^+^ 173.1033, found 173.1035 (5.0 ppm). IR (ATR) ν_max_: 3310, 3159, 3373, 1634.

*Data for (*S*)-2-nitro-1-(1,2,3,4-tetrahydronaphthalen-1-yl)guanidine S*-**12**. White solid, 59% yield, m.p. 180–182 °C; TLC *R*_f_ 0.40 (AcOEt/Hexane, 1:1); [α]^26^ = −49.29 (*c* = 0.1, isopropyl alcohol); ^1^H NMR (500 MHz, CD_3_OD, 50 °C): δ 7.29–7.11 (m, 4H), 5.00 (s, 1H), 2.91–2.74 (m, 2H), 2.13–2.05 (m, 1H), 1.97–1.81 (m, 3H). ^13^C NMR (126 MHz, CD_3_OD, 50 °C): δ 159.05, 137.35, 128.89, 128.02, 127.30, 125.94, 49.53, 29.62, 28.58, 19.48. HRMS (ESI): [M + Na]^+^ calculated for C_11_H_14_N_4_NaO_2_ [M + Na]^+^ 257.1009 found 257.1014 (5.0 ppm). IR (ATR) ν_max_: 3380, 3191, 3322, 1643.

*Data for (*R*)-1-(1-cyclohexylethyl)-2-nitroguanidine R*-**13**. White solid, 61% yield, m.p. 139–140 °C; TLC *R*_f_ 0.38 (AcOEt/Hexane, 6:4); [α]^26.3^ = −30.71 (*c* = 0.5, acetone); ¹H NMR (500 MHz, CDCl_3_): δ 8.45 (br s, 1H), 6.63 (br s, 2H), 3.32 (br s, 1H), 1.82–1.68 (m, 6H), 1.48 (br s, 1H), 1.29–0.95 (m, 9H), ^13^C NMR (125 MHz, CDCl_3_): δ 158.6, 53.3, 42.9, 28.9, 26.1, 25.9, 17.6. HRMS (ESI): calculated for C_9_H_19_N_4_O_2_ [M + H]^+^ 215.1503, found 215.1508 (5.0 ppm). IR (ATR) ν_max_: 3355, 3310, 3170, 1644.

*Data for (*R*)-1-(1-(naphthalen-1-yl)ethyl)-2-nitroguanidine R*-**14**. Yellow solid, 30% yield, m.p. 175 °C (decomposes); TLC *R*_f_ 0.38 (AcOEt/Hexane, 6:4); [α]^26.5^ = −67.5 (*c* = 0.5, acetone,); ^1^H NMR (500 MHz, CDCl_3_): δ 8.30 (br s, 1H), 8.02 (d, *J* = 8.4 Hz, 1H), 7.95 (d, *J* = 7.5 Hz, 1H), 7.87 (d, *J* = 8.2 Hz, 1H), 7.65–7.60 (m, 1H), 7.60–7.56 (m, 3H), 7.54–7.50 (m, 1H), 6.15 (br s, 2H), 5.42 (s, 1H), 1.78 (d, *J* = 6.8 Hz, 3H), ^13^C NMR (125 MHz, CDCl_3_): δ 158.9, 135.4, 134.1, 129.6, 129.6, 129.3, 127.2, 126.3, 125.8, 122.8, 121.5, 49.2, 22.9. HRMS (ESI): calculated for C_13_H_14_N_4_NaO_2_ [M + Na]^+^ 281.1009, found 281.1014 (5.0 ppm). IR (ATR) ν_max_: 3415, 3222, 3283, 1637.

*Data for (*S*)-1-(1-(naphthalen-1-yl)ethyl)-2-nitroguanidine S*-**15**. Yellow solid, 46% yield, m.p 178 °C; TLC *R*_f_ 0.38 (AcOEt/Hexane, 6:4); [α]^26.6^ = +74.77 (*c* = 0.5, acetone), ^1^H NMR (500 MHz, CDCl_3_): δ 8.35 (s, 1H), 8.02 (d, *J* = 8.4 Hz, 1H), 7.97–7.93 (m, 1H), 7.87 (d, *J* = 8.1 Hz, 1H), 7.62 (ddd, *J* = 8.5, 6.8, 1.5 Hz, 1H), 7.60–7.56 (m, 2H), 7.52 (dd, *J* = 8.2, 7.2 Hz, 1H), 6.19 (s, 2H), 5.42 (s, 1H), 1.78 (d, *J* = 6.7 Hz, 3H), ^13^C NMR (125 MHz, CDCl_3_): δ 158.9, 135.3, 134.1, 129.6, 129.5, 129.3, 127.2, 126.3, 125.8, 122.8, 121.5, 49.1, 22.9. HRMS (ESI): [M + Na]^+^ calculated for C_13_H_14_N_4_NaO_2_ [M + Na]^+^ 281.1009, found 281.1014 (5.0 ppm). IR (ATR) ν_max_: 3415, 3222, 3283, 1636.

*Data for (*R*)-2-nitro-1-(1-phenylethyl)guanidine R*-**16**. Yellow solid, 79% yield, m.p. 126–127 °C; TLC *R*_f_ 0.29 (AcOEt/Hexane, 6:4) [α]^24.4^ = +37.4 (*c* = 0.5, acetone), ¹H NMR (500 MHz, CDCl_3_): δ 8.36 (br s, 1H), 7.43–7.40 (m, 2H), 7.37–7.33 (m, 3H), 6.46 (br s, 2H), 4.62 (br s, 1H), 1.64 (d, *J* = 6.8 Hz, 3H), ^13^C NMR (125 MHz, CDCl_3_): δ 159.0, 140.6, 129.6, 128.7, 125.5, 52.7, 24.0. HRMS (ESI): calculated for C_9_H_13_N_4_O_2_ [M + H]^+^ 209.1033, found 209.1039 (5.0 ppm). IR (ATR) ν_max_: 3392, 3210.

*Data for (*S*)-2-nitro-1-(1-phenylethyl)guanidine S*-**17**. Yellow solid, 95% yield, m.p. 127–128 °C; TLC *R*_f_ 0.29 (AcOEt/Hexane, 6:4) [α]^23.9^ = −40.32 (*c* = 0.5, acetone,), ¹H NMR (500 MHz, CDCl_3_): δ 8.41 (br s, 1H), 7.43–7.40 (m, 2H), 7.36–7.33 (m, 3H), 6.52 (br s, 2H), 4.62 (br s, 1H), 1.63 (3H, d, *J* = 6.8 Hz), ^13^C NMR (125 MHz, CDCl_3_): δ 159.0, 140.6, 129.6, 128.6, 125.5, 52.6, 23.9. HRMS (ESI): calculated for C_9_H_13_N_4_O_2_ [M + H]^+^ 209.1033, found 209.1039 (2.86 ppm). IR (ATR) ν_max_: 3392, 3211.

## Appendix B

*Data for (E)-N-(3-(4-methylbenzyl)-1,3,5-oxadiazinan-4-ylidene)nitramide***18**. White solid, 87% yield, m.p. 110–113 °C; TLC *R*_f_ 0.44 (AcOEt/Hexane, 6:4); ^1^H NMR (500 MHz, DMSO-*d_6_*, 50 °C): δ 9.73 (br s, 1H), 7.21 (d, *J* = 8.2 Hz, 2H), 7.18 (d, *J* = 8.1 Hz, 2H), 4.97 (s, 2H), 4.93 (s, 2H), 4.56 (s, 2H), 2.30 (s, 3H), ^13^C NMR (125 MHz, DMSO-*d_6_*, 50 °C): δ 154.6, 137.2, 133.6, 129.6, 127.8, 77.3, 73.8, 48.5, 21.0. HRMS (ESI): calculated for C_11_H_14_N_4_O_3_Na [M + Na]^+^ 273.0958, found 273.0964 (−3.7 ppm). IR (ATR) ν_max_: 3275, 1545.

*Data for (E)-N-(3-(naphthalen-2-ylmethyl)-1,3,5-oxadiazinan-4-ylidene)nitramide***19**. White solid, 88% yield, m.p. 170–174 °C; TLC *R*_f_ 0.47 (AcOEt/Hexane, 6:4); ^1^H NMR (500 MHz, DMSO-*d_6_*, 50 °C): δ 9.85 (s, 1H), 8.12–8.07 (m, 1H), 8.01–7.95 (m, 1H), 7.90 (d, *J* = 8.2 Hz, 1H), 7.63–7.56 (m, 2H), 7.52 (dd, *J* = 8.2, 7.1 Hz, 1H), 7.43–7.39 (m, 1H), 5.13 (s, 2H), 5.04 (s, 2H), 4.97 (s, 2H), ^13^C NMR (125 MHz, DMSO-*d_6_*, 50 °C): δ 154.7, 133.9, 131.8, 131.1, 129.1, 128.4, 126.9, 126.5, 125.9, 124.6, 123.4, 78.1, 73.9, 47.1 ppm. HRMS (ESI): calculated for C_14_H_14_N_4_O_3_Na [M + Na]^+^ 309.0958, found 309.0964 (−2.6 ppm). IR (ATR) ν_max_: 3297, 1546.

*Data for (E)-N-(3-(4-chlorobenzyl)-1,3,5-oxadiazinan-4-ylidene)nitramide***20**. White solid, 9% yield, m.p. 123–127 °C; TLC *R*_f_ 0.34 (AcOEt/Hexane, 6:4); ^1^H NMR (500 MHz, DMSO-*d*_6_): δ 9.82 (s, 1H), 7.44 (d, *J* = 8.1 Hz, 3H), 7.33 (d, *J* = 8.1 Hz, 3H), 5.00–4.94 (m, 4H), 4.59 (s, 2H), ^13^C NMR (125 MHz, DMSO-*d*_6_): δ 154.4, 135.9, 132.5, 129.6, 129.1, 78.1, 73.86, 48.1. HRMS (ESI): calculated for C_10_H_11_CIN_4_O_3_Na [M + Na]^+^ 293.0412, found 293.0417 (−3.4 ppm). IR (ATR) ν_max_: 3308, 1589.

*Data for (E)-N-(3-(4-(trifluoromethyl)benzyl)-1,3,5-oxadiazinan-4-ylidene)nitramide***21**. White solid, 67% yield, m.p. 176–178 °C; TLC *R*_f_ 0.31 (AcOEt/Hexane, 6:4); ^1^H NMR (500 MHz, DMSO-*d*_6_): δ 9.86 (s, 1H), 7.75 (d, *J* = 8.1 Hz, 2H), 7.51 (d, *J* = 8.0 Hz, 2H), 5.00 (s, 2H), 4.99 (s, 2H), 4.69 (s, 2H), ^13^C NMR (125 MHz, DMSO-*d*_6_): δ 154.9, 138.9, 130.6 (q, *J* = 36.25 Hz), 128.0, 126.02 (q, *J* = 3.75 Hz), 123.8 (q, *J* = 270 Hz), 77.6, 73.5, 48.8, ^19^F NMR (470 MHz, DMSO-*d_6_*): δ −60.86. HRMS (ESI): calculated for C_11_H_12_F_3_N_4_O_3_^+^ [M + H]^+^ 305.0856, found 305.0861 (−4.9 ppm). IR (ATR) ν_max_: 3316, 1550.

*Data for (E)-N-(3-(2-methoxybenzyl)-1,3,5-oxadiazinan-4-ylidene)nitramide***22**. White solid, 17% yield, m.p. 143–145 °C; TLC *R*_f_ 0.44 (AcOEt/Hexane, 6:4); ^1^H NMR (500 MHz, DMSO-*d*_6_, 50 °C): δ 9.71 (s, 1H), 7.29 (td, *J* = 8.3, 7.4, 1.7 Hz, 1H), 7.21 (dd, *J* = 7.5, 1.7 Hz, 1H), 7.03 (dd, *J* = 8.2, 1.1 Hz, 1H), 6.95 (td, *J* = 7.5, 1.1 Hz, 1H), 4.98 (s, 2H), 4.96 (s, 2H), 4.54 (s, 2H), 3.83 (s, 3H), ^13^C NMR (125 MHz, DMSO-*d*_6_, 50 °C): δ 157.3, 154.7, 129.2, 128.3, 124.4, 120.8, 111.4, 78.5, 73.9, 55.9, 44.7. HRMS (ESI): calculated for C_11_H_14_N_4_0_4_Na [M + Na]^+^ 289.0907, found 289.0913 (−2.4 ppm). IR (ATR) ν_max_: 3284, 1551.

*Data for (E)-N-(3-(4-methoxybenzyl)-1,3,5-oxadiazinan-4-ylidene)nitramide***23**. White solid, 36% yield, m.p. 162–167 °C; TLC *R*_f_ 0.34 (AcOEt/Hexane, 6:4); ^1^H NMR (500 MHz, DMSO-*d*_6_, 50 °C): δ 9.72 (s, 1H), 7.29–7.23 (m, 2H), 6.93 (d, *J* = 8.7 Hz, 2H), 4.96 (d, *J* = 2.4 Hz, 2H), 4.93 (s, 2H), 4.54 (s, 2H), 3.76 (s, 3H), ^13^C NMR (125 MHz, DMSO-*d*_6_, 50 °C): δ 159.3, 154.5, 129.3, 128.6, 114.6, 77.8, 73.8, 55.6, 48.2. HRMS (ESI): calculated for C_11_H_14_N_4_O_4_Na [M + Na]^+^ 289.0907, found 289.0913 (−3.5 ppm). IR (ATR) ν_max_: 3266, 1547.

*Data for (R)-N-(3-(1-phenylethyl)-1,3,5-oxadiazinan-4-ylidene)nitramide R*-**24**. White solid, 57% yield, m.p. 108–109 °C; TLC *R*_f_ 0.44 (AcOEt/Hexane, 6:4) [α]^25.5^ = +169.78 (*c* = 0.5, acetone), ^1^H NMR (500 MHz, CDCl_3_): δ 10.00 (s, 1H), 7.41–7.36 (m, 3H), 7.35–7.30 (m, 4H), 6.06 (q, *J* = 7.1 Hz, 1H), 5.00 (dd, *J* = 8.6, 2.4 Hz, 1H), 4.95 (dd, *J* = 8.6, 2.2 Hz, 1H), 4.76 (d, *J* = 9.4 Hz, 1H), 4.51 (d, *J* = 9.4 Hz, 1H), ^13^C NMR (125 MHz, CDCl_3_): δ 154.4, 137.8, 128.9, 128.2, 127.2, 73.8, 73.7, 52.2, 16.1. HRMS (ESI): calculated for C_11_H_14_N_4_NaO_3_^+^ [M + Na]^+^ 273.0958, found 273.0964 (5.0 ppm). IR (ATR) ν_max_: 3280.

*Data for (*S*)-N-(3-(1-phenylethyl)-1,3,5-oxadiazinan-4-ylidene)nitramide S*-**25**. White solid, 54% yield, m.p. 104–105 °C; TLC *R*_f_ 0.44 (AcOEt/Hexane, 6:4) [α]^25.9^ = −165.12 (*c* = 0.5, acetone,), ^1^H NMR (500 MHz, CDCl_3_): δ 9.98 (s, 1H), 7.41–7.35 (m, 2H), 7.35–7.30 (m, 3H), 6.05 (q, *J* = 7.1 Hz, 1H), 5.01 (dd, *J* = 8.6, 2.6 Hz, 1H), 4.95 (dd, *J* = 8.6, 2.5 Hz, 1H), 4.76 (d, *J* = 9.4 Hz, 1H), 4.51 (d, *J* = 9.4 Hz, 1H), 1.59 (d, *J* = 7.2 Hz, 3H), ^13^C NMR (125 MHz, CDCl_3_): δ 154.4, 137.8, 128.9, 128.2, 127.2, 73.8, 73.7, 52.1, 16.1. HRMS (ESI): calculated for C_11_H_14_N_4_NaO_3_ [M + Na]^+^ 273.0958, found 273.0964 (5.0 ppm). IR (ATR) ν_max_: 3282, 1578.

*Data for (R)-N-(3-(1-(naphthalen-1-yl)ethyl)-1,3,5-oxadiazinan-4-ylidene)nitramide R*-**26**. White solid, 37% yield, m.p. 211–214 °C (decomposes); TLC *R*_f_ 0.55 (AcOEt/Hexane, 6:4) [α]^26.7^ = +174.44 (*c =* 0.5, acetone), ^1^H NMR (500 MHz, CDCl_3_): δ 10.00 (s, 1H), 8.06 (dd, *J* = 8.4, 1.1 Hz, 1H), 7.93–7.87 (m, 2H), 7.64–7.60 (m, 1H), 7.59–7.55 (m, 2H), 7.52–7.48 (m, 1H), 6.58 (q, *J* = 6.8 Hz, 1H), 4.98 (ddd, *J* = 8.7, 3.5, 1.2 Hz, 1H), 4.72 (ddd, *J* = 9.6, 7.7, 1.6 Hz, 2H), 3.97 (d, *J* = 9.6 Hz, 1H), 1.79 (d, *J* = 6.8 Hz, 3H), ^13^C NMR (125 MHz, CDCl_3_): δ 153.9, 133.8, 132.4, 131.5, 129.8, 128.9, 127.5, 126.4, 125.0, 124.8, 123.2, 73.7, 73.4, 49.9, 16.3. HRMS (ESI): [M + Na]^+^ calculated for C_15_H_16_N_4_NaO_3_ [M + Na]^+^ 323.1115, found 323.1120 (5.0 ppm). IR (ATR) ν_max_: 3269, 1572.

*Data for (S)-N-(3-(1-(naphthalen-1-yl)ethyl)-1,3,5-oxadiazinan-4-ylidene)nitramide S*-**27**. White solid*,* m.p. 210–213 °C; TLC *R*_f_ 0.55 (AcOEt/Hexane, 6:4) [α]^26.6^ = −198.29 (*c =* 0.5, acetone), ^1^H NMR (500 MHz, CDCl_3_): δ 9.98 (s, 1H), 8.04 (d, *J* = 8.3 Hz, 1H), 7.89 (t, *J* = 7.8 Hz, 2H), 7.60 (t, *J* = 6.9 Hz, 1H), 7.55 (dd, *J* = 7.0, 3.0 Hz, 2H), 7.50–7.46 (m, 1H), 6.56 (q, *J* = 6.8 Hz, 1H), 4.96 (ddd, *J* = 8.6, 3.5, 1.0 Hz, 1H), 4.73–4.66 (m, 2H), 3.95 (d, *J* = 9.6 Hz, 1H), 1.77 (d, *J* = 6.8 Hz, 3H), ^13^C NMR (125 MHz, CDCl_3_): δ 153.9, 133.8, 132.5, 131.5, 129.8, 128.9, 127.5, 126.4, 125.0, 124.8, 123.2, 73.7, 73.4, 49.9, 16.3. HRMS (ESI): calculated for C_15_H_16_N_4_NaO_3_^+^ [M + Na]^+^ 323.1115, found 323.1120 (5.0 ppm). IR (ATR) ν_max_: 3269, 1573.

*Data for (R)-N-(3-(1-cyclohexylethyl)-1,3,5-oxadiazinan-4-ylidene)nitramide R*-**28**. White solid, 38% yield, m.p. 139–140 °C; TLC *R*_f_ 0.51 (AcOEt/Hexane, 6:4) [α]^26.3^ = −22.39 (*c =* 0.5, acetone), ¹H NMR (500 MHz, CDCl_3_): δ 9.99 (br s, 1H), 5.01-5.00 (d, *J* = 2.4 Hz, 2H), 4.88–4.86 (d, *J* = 9.5 Hz, 1H), 4.82–4.80 (d, *J* = 9.4 Hz, 1H), 4.48–4.43 (m, 1H), 1.78–1.66 (m, 5H), 1.37–1.30 (m, 1H), 1.25–0.95 (m, 9H), ^13^C NMR (125 MHz, CDCl_3_): δ 154.90, 73.72, 73.31, 55.50, 40.82, 29.81, 29.73, 25.93, 25.80, 16.06. HRMS (ESI): calculated for C_11_H_21_N_4_O_3_ [M + H]^+^ 257.1608, found 257.1614 (5.0 ppm). IR (ATR) ν_max_: 3729, 1567.

*Data for (S)-N-(3-(1-cyclopropylethyl)-1,3,5-oxadiazinan-4-ylidene)nitramide S*-**29**. White solid, 64% yield, m.p. 111–113 °C; TLC *R*_f_ 0.63 (AcOEt/Hexane, 7:3) [α]^26^ = +12.15 (*c =* 1, acetone), ^1^H NMR (500 MHz, DMSO-*d*_6_, 50 °C): δ 9.67 (s, 1H), 5.09 (s, 2H), 4.93 (s, 2H), 3.71 (dq, *J* = 9.3, 6.9 Hz, 1H), 1.21 (d, *J* = 6.9 Hz, 3H), 1.10–1.01 (m, 1H), 0.61–0.53 (m, 1H), 0.49–0.42 (m, 1H), 0.36–0.30 (m, 1H), 0.29–0.23 (m, 1H), ^13^C NMR (125 MHz, DMSO-*d*_6_, 50 °C): δ 153.3, 73.5, 73.1, 55.2, 16.8, 13.7, 4.2, 3.0. HRMS (ESI): calculated for C_8_H_14_N_4_NaO_3_ [M + Na]^+^ 237.0958 found 237.0954 (5.0 ppm). IR (ATR) ν_max_: 3295, 1578.

*Data for (S)-N-(3-(1,2,3,4-tetrahydronaphthalen-1-yl)-1,3,5-oxadiazinan-4-ylidene)nitramide S**-*****30**. White solid, 19% yield, m.p. 167–169 °C; TLC *R*_f_ 0.25 (AcOEt/Hexane, 6:4) [α]^24^ = −47.14 (*c =* 1, acetone). ^1^H NMR (500 MHz, CDCl_3_): δ 10.05 (s, 1H), 7.21–7.11 (m, 4H), 5.94 (dd, *J* = 10.2, 6.1 Hz, 1H), 5.04 (d, *J* = 2.4 Hz, 2H), 4.69 (d, *J* = 9.6 Hz, 1H), 4.60 (d, *J* = 9.5 Hz, 1H), 2.86–2.73 (m, 2H), 2.24–2.17 (m, 1H), 1.99–1.91 (m, 1H), 1.89–1.79 (m, 1H), 1.72 (tdd, *J* = 12.2, 10.0, 2.9 Hz, 1H). ^13^C NMR (125 MHz, CDCl_3_): δ 155.19, 138.67, 132.49, 129.64, 127.76, 127.36, 126.59, 74.76, 74.03, 54.17, 29.19, 28.21, 21.43. HRMS (ESI): calculated for C_13_H_16_N_4_NaO_3_ [M + Na]^+^ 299.1115, found 299.1111 (5.0 ppm). IR (ATR) ν_max_: 3303, 1557.

*Data for N-(3-phenethyl-1,3,5-oxadiazinan-4-ylidene)nitramide R*-**31**. White solid, 35% yield, m.p. 134–136 °C; TLC *R*_f_ 0.45 (AcOEt/Hexane, 6:4) ^1^H NMR (500 MHz, CDCl_3_): δ 9.77 (br s, 1H), 7.35–7.31 (m, 2H), 7.28–7.25 (m, 1H), 7.23–7.19 (m, 2H), 4.89 (d, *J* = 2.7 Hz, 2H), 4.46 (s, 2H), 3.67 (t, *J* = 6.8 Hz, 2H), 2.96 (t, *J* = 6.8 Hz, 2H), ^13^C NMR (125 MHz, CDCl_3_): δ 154.5, 138.1, 128.9, 128.8, 126.9, 78.8, 73.2, 48.9, 34.4. HRMS (ESI): [M + Na]^+^ calculated for C_11_H_14_N_4_NaO_3_ [M + Na]^+^ 273.0958, found 273.0956. (5.0 ppm). IR (ATR) ν_max_: 3324, 1587.

## Appendix C

*Data for (E)-1-methyl-3-(4-methylbenzyl)-2-nitroguanidine***33**. White solid, 85%, m.p. 166–168 °C; TLC *R*_f_ 0.33 (AcOEt/Hexane, 6:4); ^1^H NMR (500 MHz, DMSO-*d*_6_): δ 9.17 (br s, 1H), 7.79 (br s, 1H), 7.19 (d, *J* = 7.8 Hz, 2H), 7.15 (d, *J* = 7.8 Hz, 2H), 4.38 (s, 2H), 2.84 (br s, 3H), 2.28 (s, 3H), ^13^C NMR (125 MHz, DMSO-*d*_6_): δ 158.3, 136.6, 129.4, 127.4, 44.2, 28.7, 21.1. HRMS (ESI): calculated for C_10_H_14_N_4_O_2_Na [M + Na]^+^ 245.1009, found 245.1014 (−2.9 ppm). IR (ATR) ν_max_: 3285, 1627.

*Data for (E)-1-methyl-3-(naphthalen-2-ylmethyl)-2-nitroguanidine***34**. White solid, 38% yield, m.p. 159–162 °C; TLC *R*_f_ 0.37 (AcOEt/Hexane, 6:4); ^1^H NMR (500 MHz, DMSO-*d*_6_, 50 °C): δ 8.11 (d, *J* = 8.2 Hz, 1H), 7.99–7.95 (m, 1H), 7.87 (d, *J* = 8.1 Hz, 1H), 7.62–7.54 (m, 2H), 7.50 (dd, *J* = 8.1, 7.1 Hz, 1H), 7.46 (dd, *J* = 7.0, 1.3 Hz, 1H), 7.33 (s, 2H), 4.93 (d, *J* = 4.7 Hz, 2H), 2.88 (s, 3H), ^13^C NMR (125 MHz, DMSO-*d*_6_, 50 °C): δ 158.5, 133.7, 130.9, 128.1, 124.6, 42.8, 28.8. HRMS (ESI): calculated for C_13_H_14_N_4_O_2_Na [M + Na]^+^ 281.1009, found 281.1014 (−1.8 ppm). IR (ATR) ν_max_: 3352, 1623.

*Data for (E)-1-(4-chlorobenzyl)-3-methyl-2-nitroguanidine***35**. White solid, 77% yield, m.p. 191–194 °C; TLC *R*_f_ 0.23 (AcOEt/Hexane, 6:4); ^1^H NMR (500 MHz, DMSO-*d*_6_): δ 9.19 (br s, 1H), 7.85 (br s, 1H), 7.41 (d, *J* = 8.1 Hz, 2H), 7.32 (d, *J* = 8.0 Hz, 2H), 4.41 (s, 2H), 2.85 (br s, 3H), ^13^C NMR (125 MHz, DMSO-*d*_6_): δ 158.3, 132.0, 129.3, 128.7, 43.8, 28.8. HRMS (ESI): calculated for C_9_H_11_CIN_4_O_2_Na [M + Na]^+^ 265.0463, found 265.0468 (−4.2 ppm). IR (ATR) ν_max_: 3282, 1625.

*Data for (E)-1-methyl-2-nitro-3-(4-(trifluoromethyl)benzyl)guanidine***36**. White solid, 74% yield, m.p. 170–173 °C; TLC *R*_f_ 0.20 (AcOEt/Hexane, 6:4); ^1^H NMR (500 MHz, DMSO-*d*_6_): δ 9.22 (br s, 1H), 7.88 (br s, 1H), 7.72 (d, *J* = 8.0 Hz, 2H), 7.51 (d, *J* = 8.0 Hz, 2H), 4.51 (s, 2H), 2.88 (br s, 3H), ^13^C NMR (125 MHz, DMSO-*d*_6_): δ 158.4, 131.2, 128.0, 125.7 (q, *J* = 3.5 Hz), 124.7 (q, *J* = 261.25 Hz), 123.7, 44.1, 28.8, ^19^F NMR (470 MHz, DMSO-*d*_6_): δ −60.77. HRMS (ESI): calculated for C_10_H_12_F_3_N_4_O_2_ [M + H]^+^ 277.0907, found 277.0912 (−2.2 ppm). IR (ATR) ν_max_: 3235, 1625.

*Data for (E)-1-(2-methoxybenzyl)-3-methyl-2-nitroguanidine***37**. White solid, 74% yield, m.p. 148–152 °C; TLC *R*_f_ 0.33 (AcOEt/Hexane, 6:4); ^1^H NMR (500 MHz, DMSO-*d*_6_, 50 °C): δ 8.20 (d, *J* = 164.9 Hz, 2H), 7.29 (td, *J* = 7.9, 1.7 Hz, 1H), 7.20 (d, *J* = 7.4 Hz, 1H), 7.03 (d, *J* = 8.2 Hz, 1H), 6.95 (td, *J* = 7.4, 1.0 Hz, 1H), 4.42 (d, *J* = 5.5 Hz, 2H), 3.84 (s, 3H), 2.85 (s, 3H), ^13^C NMR (125 MHz, DMSO-*d*_6_): δ 169.0, 136.8, 129.6, 127.9, 120.7, 111.0, 55.8, 28.7, 26.5. HRMS (ESI): [M + Na]^+^ calculated for C_10_H_14_N_4_O_3_Na [M + Na]^+^ 261.0958, found 261.0964 (−1.9 ppm). IR (ATR) ν_max_: 3328, 1621.

*Data for (E)-1-(4-methoxybenzyl)-3-methyl-2-nitroguanidine***38**. White solid, 82% yield, m.p. 189–192 °C; TLC *R*_f_ 0.23 (AcOEt/Hexane, 6:4); ^1^H NMR (500 MHz, DMSO-*d*_6_): δ 9.16 (s, 1H), 7.77 (s, 1H), 7.24 (d, *J* = 8.1 Hz, 2H), 6.91 (d, *J* = 8.2 Hz, 2H), 4.35 (d, *J* = 5.2 Hz, 2H), 3.73 (s, 3H), 2.83 (s, 3H), ^13^C NMR (125 MHz, DMSO-*d*_6_): δ 158.9, 158.2, 131.0, 129.0, 114.2, 55.5, 43.9, 28.7. HRMS (ESI): calculated for C_10_H_14_N_4_O_3_Na^+^ [M + Na]^+^ 261.0958, found 261.0964 (−1.5 ppm). IR (ATR) ν_max_: 3264, 1627.

*Data for (S)-1-(1-cyclopropylethyl)-3-methyl-2-nitroguanidine S*-**39**. White solid, 13% yield, m.p. 148–150 °C; TLC *R*_f_ 0.31 (AcOEt/Hexane, 6:4) [α]^25^ = +31.89 (*c* = 1, acetone), ^1^H NMR (500 MHz, DMSO-*d*_6_, 50 °C): δ 8.27 (s, 2H), 3.36–3.26 (m, 1H), 2.81 (d, *J* = 4.9 Hz, 3H), 1.21 (d, *J* = 6.6 Hz, 3H), 1.08–0.99 (m, 1H), 0.53–0.46 (m, 1H), 0.46–0.39 (m, 1H), 0.34–0.28 (m, 1H), 0.24–0.17 (m, 1H), ^13^C NMR (125 MHz, DMSO-*d*_6_, 50 °C): δ 157.25, 51.01, 28.01, 19.89, 16.68, 2.91, 2.70. HRMS (ESI): calculated for C_7_H_14_N_4_NaO_2_ [M + Na]^+^ 209.1009, found 209.1006 (5.0 ppm). IR (ATR) ν_max_: 3334, 3290, 1622.

*Data for (S)-1-methyl-2-nitro-3-(1,2,3,4-tetrahydronaphthalen-1-yl)guanidine S*-**40**. White solid, 29% yield, m.p. 192–194 °C; TLC *R*_f_ 0.43 (AcOEt/Hexane, 6:4) [α]^26^ = −2.98 (*c* = 1, acetone), ^1^H NMR (500 MHz, DMSO-*d*_6_, 50 °C): δ 8.28 (br s, 2H), 7.28–7.09 (m, 4H), 5.05 (s, 1H), 2.86 (s, 3H), 2.83–2.68 (m, 2H), 2.06–1.97 (m, 1H), 1.91 (d, *J* = 6.8 Hz, 1H), 1.86-1.80 (m, 1H), 1.79–1.70 (m, 1H), ^13^C NMR (125 MHz, DMSO-*d*_6_, 50°C): δ 157.6, 137.1, 128.6, 127.4, 126.9, 125.8, 49.3, 29.5, 28.4, 28.1, 19.9. HRMS (ESI): calculated for C_12_H_17_N_4_O_2_ [M + H]^+^ 249.1346, found 249.1340 (5.0 ppm) IR (ATR) ν_max_: 3337, 3302, 1623.

*Data for 1-methyl-2-nitro-3-(thiophen-2-ylmethyl)guanidine***41**. Yellow solid, 57%, m.p. 100–102 °C; TLC *R*_f_ 0.29 (AcOEt/Hexane, 65:35) ^1^H NMR (500 MHz, DMSO-*d*_6_): δ 9.18 (s, 1H), 8.10–7.82 (m, 1H), 7.42 (d, *J* = 5.1 Hz, 1H), 7.03 (s, 1H), 7.00–6.95 (m, 1H), 4.57 (s, 2H), 2.81 (s, 3H), ^13^C NMR (125 MHz, DMSO-*d*_6_): δ 157.78, 126.86, 126.10, 125.68, 39.48, 28.51. HRMS (ESI): calculated for C_7_H_10_N_4_NaO_2_S [M + Na]^+^ 237.0417, found 237.0413 (5.0 ppm). IR (ATR) ν_max_: 3286, 3210.

*Data for 1-methyl-2-nitro-3-phenethylguanidine***42**. White solid, 55% yield, m.p. 151–153 °C; TLC *R*_f_ 0.18 (AcOEt/Hexane, 6:4) ^1^H NMR (500 MHz, DMSO-*d*_6_): δ 7.31 (t, *J* = 7.5 Hz, 2H), 7.27–7.19 (m, 3H), 3.49–3.41 (m, 2H), 2.86 (t, *J* = 7.4 Hz, 2H), 2.78 (d, *J* = 2.8 Hz, 3H), ^13^C NMR (125 MHz, DMSO-*d*_6_): δ 157.83, 138.53, 128.51, 128.20, 126.13, 42.22, 34.59, 27.97. HRMS (ESI): calculated for C_10_H_14_N_4_NaO_2_ [M + Na]^+^ 245.1009, found 245.1009 (5.0 ppm). IR (ATR) ν_max_: 3336, 3284, 1619.

These compounds were prepared following the same procedure used for the cyclization of non-methylated nitroguanidines.

*N-(3-methyl-5-(4-methylbenzyl)-1,3,5-oxadiazinan-4-ylidene)nitramide***43**. White solid, 68% yield, m.p. 146–151 °C; TLC *R*_f_ 0.06 (AcOEt/Hexane, 6:4); ^1^H NMR (500 MHz, DMSO-*d*_6_): δ 7.22–7.15 (m, 4H), 5.00 (s, 2H), 4.95 (s, 2H), 4.57 (s, 2H), 2.87 (s, 3H), 2.29 (s, 3H), ^13^C NMR (125 MHz, DMSO-*d*_6_): δ 156.8, 137.5, 132.9, 129.6, 128.1, 79.6, 77.4, 50.7, 33.9, 21.1. HRMS (ESI): calculated for C_12_H_17_N_4_O_3_ [M + H]^+^ 265.1295, found 265.1301 (−0.8 ppm). IR (ATR) ν_max_: 1601.

*Data for N-(3-methyl-5-(naphthalen-2-ylmethyl)-1,3,5-oxadiazinan-4-ylidene)nitramide***44**. White solid, 71% yield, m.p. 151–153 °C; TLC *R*_f_ 0.06 (AcOEt/Hexane, 6:4); ^1^H NMR (500 MHz, CDCl_3_): δ 7.99 (dd, *J* = 8.4, 1.1 Hz, 1H), 7.92–7.85 (m, 2H), 7.62–7.57 (m, 1H), 7.56–7.51 (m, 1H), 7.48–7.41 (m, 2H), 5.19 (s, 2H), 4.81 (s, 2H), 4.65 (s, 2H), 3.12 (s, 3H), ^13^C NMR (125 MHz, CDCl_3_): δ 157.8, 133.9, 131.2, 129.8, 129.0, 128.9, 127.7, 127.3, 126.4, 125.2, 123.0, 79.6, 50.0, 34.8. HRMS (ESI): calculated for C_15_H_17_N_4_O_3_ [M + H]^+^ 301.1295, found 301.1301 (0.3 ppm). IR (ATR) ν_max_: 1609.

*Data for (E)-N-(3-(4-chlorobenzyl)-5-methyl-1,3,5-oxadiazinan-4-ylidene)nitramide* **45**. White solid, 63% yield, m.p. 163–166 °C; TLC *R*_f_ 0.03 (AcOEt/Hexane, 6:4); ¹H NMR (500 MHz, DMSO-*d*_6_): δ 7.50–7.40 (m, 2H), 7.36–7.29 (m, 2H), 5.02 (s, 2H), 5.0 (s, 2H), 4.62 (s, 2H), 2.87 (s, 3H), ^13^C NMR (125 MHz, DMSO-*d*_6_): δ 156.9, 135.3, 132.8, 129.9, 129.0, 79.6, 77.6, 50.3, 34.0. HRMS (ESI): calculated for C_11_H_14_ClN_4_O_3_ [M + H]^+^ 285.0749, found 285.0754 (0.7 ppm). IR (ATR) ν_max_: 1608.

*Data for N-(3-methyl-5-(4-(trifluoromethyl)benzyl)-1,3,5-oxadiazinan-4-ylidene)nitramide***46**. White solid, 58% yield, m.p. 126–129 °C; TLC *R*_f_ 0.03 (AcOEt/Hexane, 6:4); ¹H NMR (500 MHz, DMSO-*d*_6_): δ 7.72 (d, *J* = 7.9 Hz, 2H), 7.50 (d, *J* = 8.0 Hz, 2H), 5.02 (s, 4H), 4.71 (s, 2H), 2.87 (s, 3H), ^13^C NMR (125 MHz, DMSO-*d*_6_): δ 157.0, 141.1, 128.7 (q*,* J= 31.25 Hz), 128.5, 125.9 (q, *J* = 3.75 Hz), 124.6 (q, *J* = 271.25 Hz), 79.6, 77.9, 50.6, 33.9, ^19^F (470 MHz, DMSO-*d*_6_): δ −60.77. HRMS (ESI): calculated for C_12_H_13_F_3_N_4_O_3_Na [M + Na]^+^ 341.0837, found 341.0837 (0.9 ppm). IR (ATR) ν_max_: 1609.

*Data for N-(3-(4-methoxybenzyl)-5-methyl-1,3,5-oxadiazinan-4-ylidene)nitramide***47**. White solid, 45% yield, m.p. 139–142 °C; TLC *R*_f_ 0.03 (AcOEt/Hexane, 6:4); ^1^H NMR (500 MHz, DMSO-*d*_6_): δ 7.24 (d, *J* = 8.4 Hz, 2H), 6.92 (d, *J* = 8.6 Hz, 1H), 4.99 (s, 2H), 4.94 (s, 2H), 4.54 (s, 2H), 3.74 (s, 2H), 2.88 (s, 3H), ^13^C NMR (125 MHz, DMSO-*d*_6_): δ 159.4, 156.8, 129.7, 127.7, 114.5, 79.5, 77.3, 55.5, 50.4, 33.9. HRMS (ESI): calculated for C_12_H_17_N_4_O_4_^+^ [M + H]^+^ 281.1244, found 281.1250 (−1.4 ppm). IR (ATR) ν_max_: 1587.

## References

[1] Hughes M.A., Martini X., Kuhns E., Colee J., Mafra-Neto A., Stelinski L.L., Smith J.A. (2017). Evaluation of repellents for the redbay ambrosia beetle, *Xyleborus glabratus*, vector of the laurel wilt pathogen. J. Appl. Entomol..

[2] Ploetz R.C., Pérez-Martínez J.M., Evans E.A., Inch S.A. (2011). Toward fungicidal management of laurel wilt of avocado. Plant Dis..

[3] Evans E.A., Crane J., Hodges A., Osborne J.L. (2010). Potential economic impact of laurel wilt disease on the Florida avocado industry. Horttechnology.

[4] McCullough D.G., Mercader R.J., Siegert N.W. (2015). Developing and integrating tactics to slow ash (*Oleaceae*) mortality caused by emerald ash borer (*Coleoptera: Buprestidae*). Can. Entomol..

[5] Bonilla-Landa I., Cuapio-Muñoz U., Luna-Hernández A., Reyes-Luna A., Rodríguez-Hernández A., Ibarra-Juarez A., Suarez-Mendez G., Barrera-Méndez F., Caram-Salas N., Enríquez-Medrano J.F. (2021). l-Proline as a Valuable Scaffold for the Synthesis of Novel Enantiopure Neonicotinoids Analogs. J. Agric. Food Chem..

[6] Elbert A., Haas M., Springer B., Thielert W., Nauen R. (2008). Applied aspects of neonicotinoid uses in crop protection. Pest Manag. Sci..

[7] Jeschke P., Nauen R. (2008). Neonicotinoids—From zero to hero in insecticide chemistry. Pest Manag. Sci..

[8] Tomizawa M., Casida J.E. (2005). Neonicotinoid insecticide toxicology: Mechanisms of selective action. Annu. Rev. Pharmacol. Toxicol..

[9] Nauen R., Denholm I. (2005). Resistance of insect pests to neonicotinoid insecticides: Current status and future prospects. Arch. Insect Biochem. Physiol..

[10] Henry M., Béguin M., Requier F., Rollin O., Odoux J.F., Aupinel P., Aptel J., Tchamitchian S., Decourtye A. (2012). A common pesticide decreases foraging success and survival in honey bees. Science.

[11] Talley T.T., Harel M., Hibbs R.E., Radic Z., Tomizawa M., Casida J.E., Taylor P. (2008). Atomic interactions of neonicotinoid agonists with AChBP: Molecular recognition of the distinctive electronegative pharmacophore. Proc. Natl. Acad. Sci. USA.

[12] Tomizawa M., Maltby D., Talley T.T., Durkin K., Medzihradszky K.F., Burlingame A.L., Taylor P., Casida J.E. (2008). Atypical nicotinic agonist bound conformations conferring subtype selectivity. Proc. Natl. Acad. Sci. USA.

[13] Ohno I., Tomizawa M., Durkin K.A., Naruse Y., Casida J.E., Kagabu S. (2009). Molecular features of neonicotinoid pharmacophore variants interacting with the insect nicotinic receptor. Chem. Res. Toxicol..

[14] Tomizawa M., Casida J.E. (2009). Molecular Recognition of Neonicotinoid Insecticides: The Determinants of Life or Death. Acc. Chem. Res..

[15] Dai H., Yu H., Liu J., Qin X., Wang T., Zhang X., Qin Z., Fang J. (2013). Synthesis and bioactivities of novel pyrazole oxime ether derivatives containing a thiazolyl moiety. Chinese J. Org. Chem..

[16] Qu W.Y., She D.M., Zhao J., Lin D.J., Huang Q.L., Li F.M. (2012). Mannich-type reaction for synthesis of 3-methyl-4-nitroimino-tetrahydro-1, 3,5-oxadiazine. Synth. Commun..

[17] Bonilla-Landa I., de la Cruz O.L., Sanchéz-Rangel D., Ortíz-Castro R., Rodriguez-Haas B., Barrera-Méndez F., de León Gómez R.E.D., Enríquez-Medrano F.J., Olivares-Romero J.L. (2018). Design, synthesis and biological evaluation of novel fungicides for the management of *Fusarium Dieback* disease. J. Mex. Chem. Soc..

[18] Xu G., Yang X., Jiang B., Lei P., Liu X., Wang Q., Zhang X., Ling Y. (2016). Synthesis and bioactivities of novel piperazine-containing 1,5-Diphenyl-2-penten-1-one analogues from natural product lead. Bioorganic Med. Chem. Lett..

[19] Dahiya R., Kumar S., Khokra S.L., Gupta S.V., Sutariya V.B., Bhatia D., Sharma A., Singh S., Maharaj S. (2018). Toward the synthesis and improved biopotential of an n-methylated analog of a proline-rich cyclic tetrapeptide from marine bacteria. Mar. Drugs.

[20] Eliel E.L., Wilen S.H. (1994). Stereochemistry of Organic Compounds.

[21] Malacrinò A., Campolo O., Laudani F., Palmeri V. (2016). Fumigant and Repellent Activity of Limonene Enantiomers Against *Tribolium confusum* du Val. Neotrop. Entomol..

[22] Jiang B., Wang H., Fu Q.-M., Li Z.-Y. (2008). The Chiral Pyrethroid Cycloprothrin: Stereoisomer Synthesis and Separation and Stereoselective Insecticidal Activity. Chirality.

[23] Finkelstein B.L., Martz M.A., Strock C.J. (1997). Synthesis and Insecticidal Activity of Novel Pyridine Methanesulfonates. Pestic. Sci..

[24] Schmitt J.D., Sharples C.G.V., Caldwell W.S. (1999). Molecular recognition in nicotinic acetylcholine receptors: The importance of π-cation interactions. J. Med. Chem..

[25] Prabhaker N., Castle S., Henneberry T.J., Toscano N.C. (2005). Assessment of cross-resistance potential to neonicotinoid insecticide in *Bemisia tabaci (Hemiptera: Aleyrodidae)*. Bull. Entomol. Res..

[26] Sivasupramaniam S., Dennehy T.J., Williams L. Management of pyrethroid-resistant whiteflies in Arizona cotton: Selection, cross-resistance and dynamics. Proceedings of the Beltwide Cotton Conferences.

[27] Kranthi K.R., Jadhav D.R., Wanjari R.R., Ali S.S., Russell D. (2001). Carbamate and organophosphate resistance in cotton pests in India, 1995 to 1999. Bull. Entomol. Res..

[28] Fishbein L., Gallaghan J.A. (1954). The preparation and reactions of 2-alkyl-1-(or 3)-nitro-2-thiopseudourea. Part I. Reaction with amines. J. Am. Chem. Soc..

[29] Maienfisch P., Huerlimann H., Rindlisbacher A., Gsell L., Dettwiler H., Haettenschwiler J., Sieger E., Walti M. (2001). The discovery of thiamethoxam: A second-generation neonicotinoid ^†^. Pest Manag. Sci..

[30] Obeng-Ofori D., Reichmuth C.H., Bekele J., Hassanali S. (2009). Biological activity of 1,8 cineole, a major component of essential oil of *Ocimum kenyense* (Ayobangira) against stored product beetles. J. Appl. Entomol..

[31] Biedermann P.H.W., Klepzig K.D., Taborsky M. (2009). Fungus Cultivation by Ambrosia Beetles: Behavior and Laboratory Breeding Success in Three Xyleborine Species. Environ. Entomol..

[32] Chen Z., Yao X., Dong F., Duan H., Shao X., Chen X., Yang T., Wang G., Zheng Y. (2019). Ecological toxicity reduction of dinotefuran to honeybee: New perspective from an enantiomeric level. Environ. Int..

