# Supplementary material for: Synthesis and Insecticidal Evaluation of Chiral Neonicotinoids Analogs: The Laurel Wilt Case
Source: Molecules. 2021 Jul 12;26(14):4225. doi: 10.3390/molecules26144225 (PMC8307524; doi:10.3390/molecules26144225)
Supplement: Supplementary file 1 [file molecules-26-04225-s001.zip › molecules-1263653-supplementary.pdf]

Synthesis and insecticidal evaluation of chiral neonicotionoids analogs: The Laurelt wilt case

Saúl A. Luna-Hernández [1], Israel Bonilla-Landa [1], Alfonso Reyes-Luna [1], Alfredo Rodríguez-Hernández [1], Ulises Cuapio-Muñoz [1], Luis A. Ibarra-Juárez [1], Gabriel Suarez-Mendez,[1] Felipe Barrera-Méndez [1,3], Irving D. Pérez-Landa [1], Francisco J. Enríquez-Medrano [2], Ramón E. Díaz de León-Gómez [2], and José L. Olivares-Romero [1,*]

[1] Instituto de Ecología, A.C., Red de Estudios Moleculares Avanzados, Clúster Científico y Tecnológico BioMimic®, Campus III, Carretera Antigua a Coatepec No. 351, 91073, Xalapa, Veracruz, México; c7h5n3o6.lh@gmail.com (S.A.L.H.); israel.bonilla@inecol.mx (I.B.L.); alfonsoreyeslunauv@gmail.com (A.R.L); fredor2236@gmail.com (A.R.H); ulisescuapio@gmail.com (U.C.H.); luis.Ibarra@inecol.mx (L.A.I.J); investigacion.suarez@hotmail.com (G.S.M.) felipe.barrera@inecol.mx (F.B.M); david.perez@posgrado.ecología.edu.mx (I.D.P.L)

[2] Centro de Investigación en Química Aplicada, Blvd. Enrique Reyna, No. 140, 25294, Saltillo, Coahuila, México.; javier.enriquez@ciqa.edu.mx (F.J.E.M.); ramon.diazdeleon@ciqa.edu.mx (R.E.D.d.L.G.)

[3] Cátedra CONACyT en el Instituto de Ecología, A.C; felipe.barrera@inecol.mx

[*] Correspondence: jose.olivares@inecol.mx

This document contains all the ^{1}H and ^{13}C NMR spectra for all compounds.

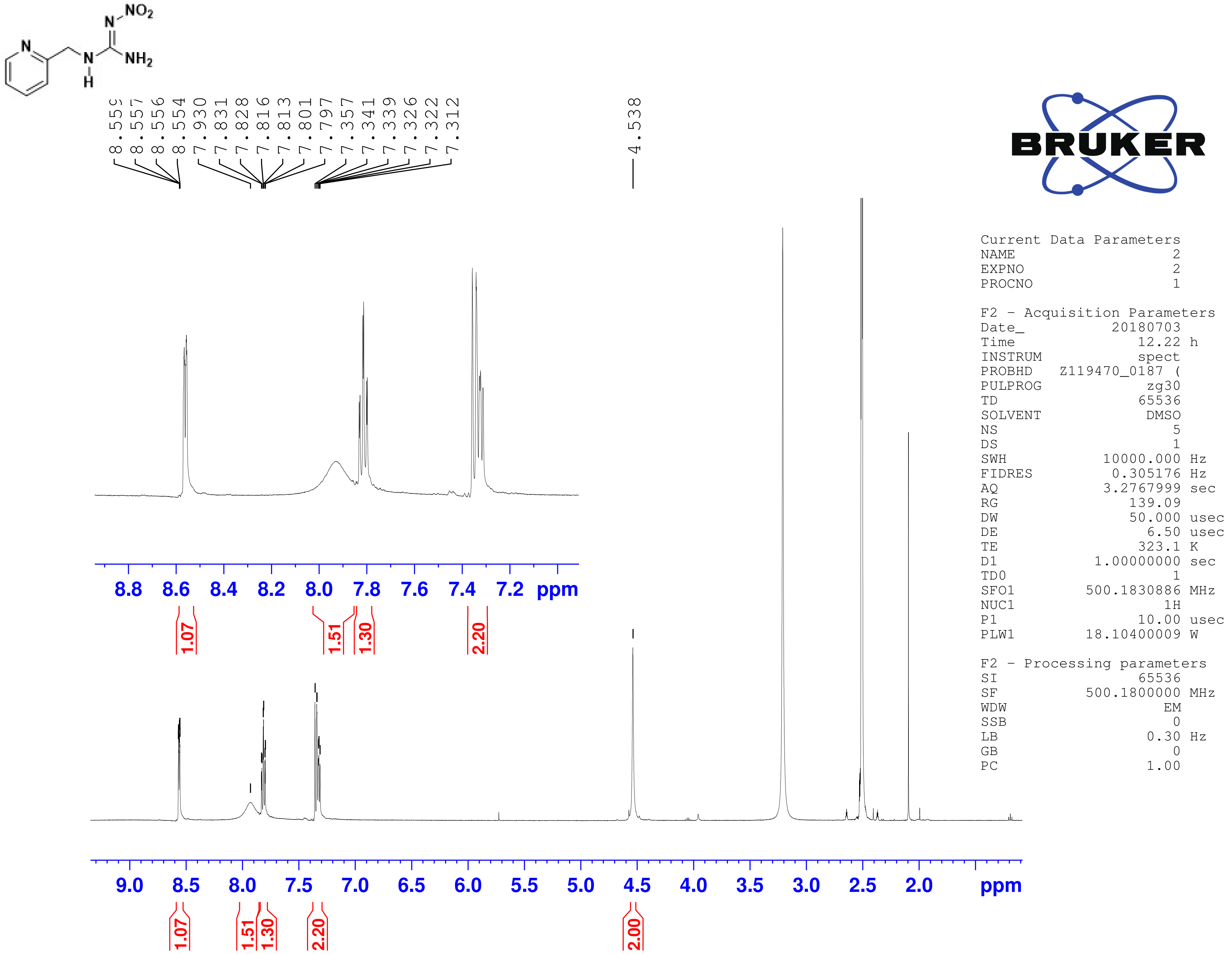

^{1}H NMR (500 MHz, DMSO-d_6, 50°C) of **2**

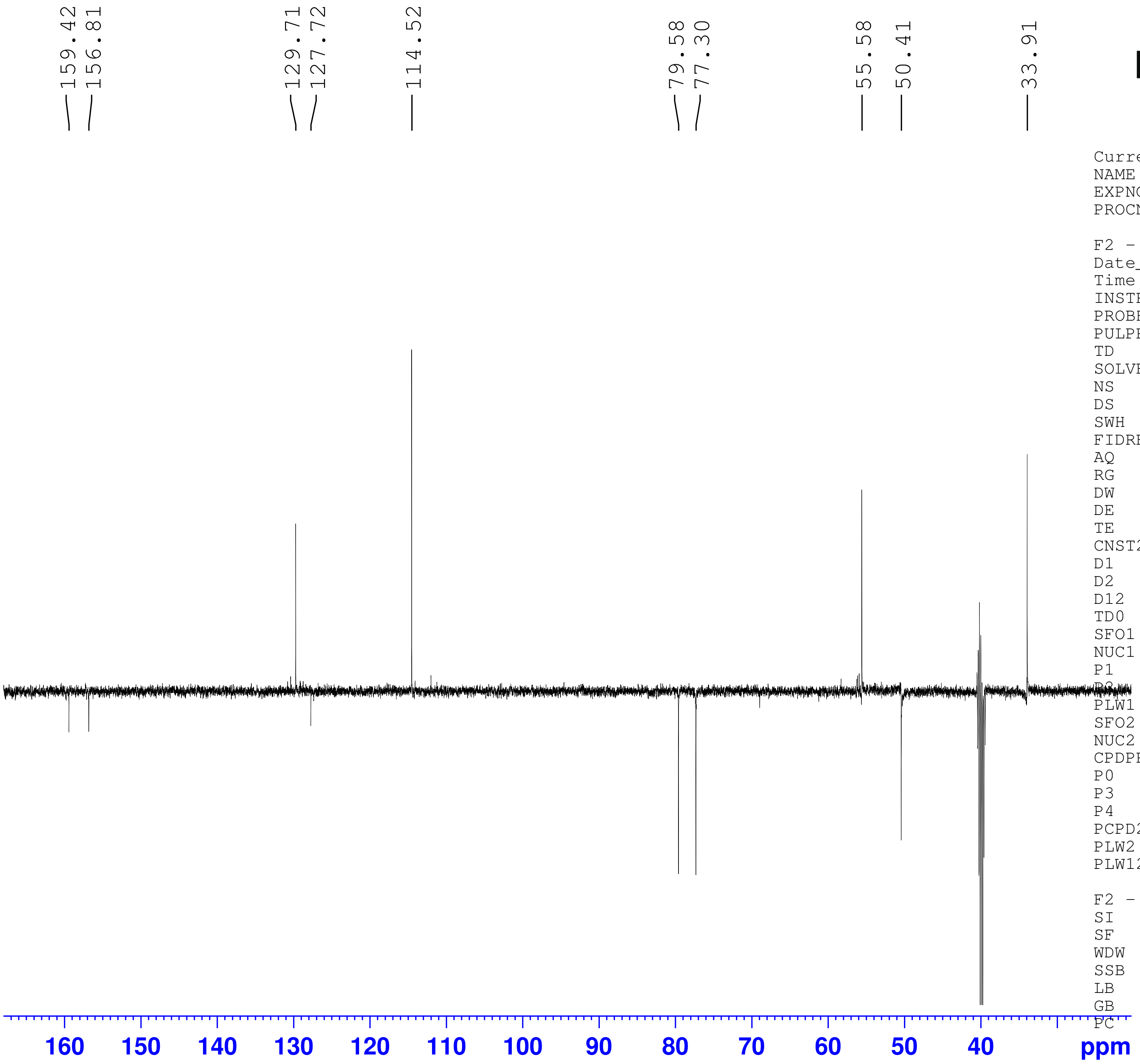

Current Data Parameters
NAME 47
EXPNO 3
PROCNO 1

F2 - Acquisition Parameters
Date_ 20190611
Time 18.18 h
INSTRUM spect
PROBHD Z119470_0187 (
PULPROG deptq135
TD 65536
SOLVENT DMSO
NS 200
DS 8
SWH 29761.904 Hz
FIDRES 0.908261 Hz
AQ 1.1010048 sec
RG 192.72
DW 16.800 usec
DE 6.50 usec
TE 298.2 K
CNST2 145.0000000
D1 2.00000000 sec
D2 0.00344828 sec
D12 0.00002000 sec
TD0 1
SFO1 125.7829381 MHz
NUC1 13C
P1 10.00 usec
P2 20.00 usec
PLW1 82.09700012 W
SFO2 500.1820007 MHz
NUC2 1H
CPDPRG[2 waltz16
P0 15.70 usec
P3 10.47 usec
P4 20.94 usec
PCPD2 80.00 usec
PLW2 18.10400009 W
PLW12 0.31009001 W

F2 - Processing parameters
SI 32768
SF 125.7703610 MHz
WDW EM
SSB 0
LB 1.00 Hz
GB 0
PC 1.40

^{13}C (DEPTQ135) NMR (125 MHz, DMSO-d_6) of **47**

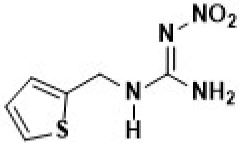

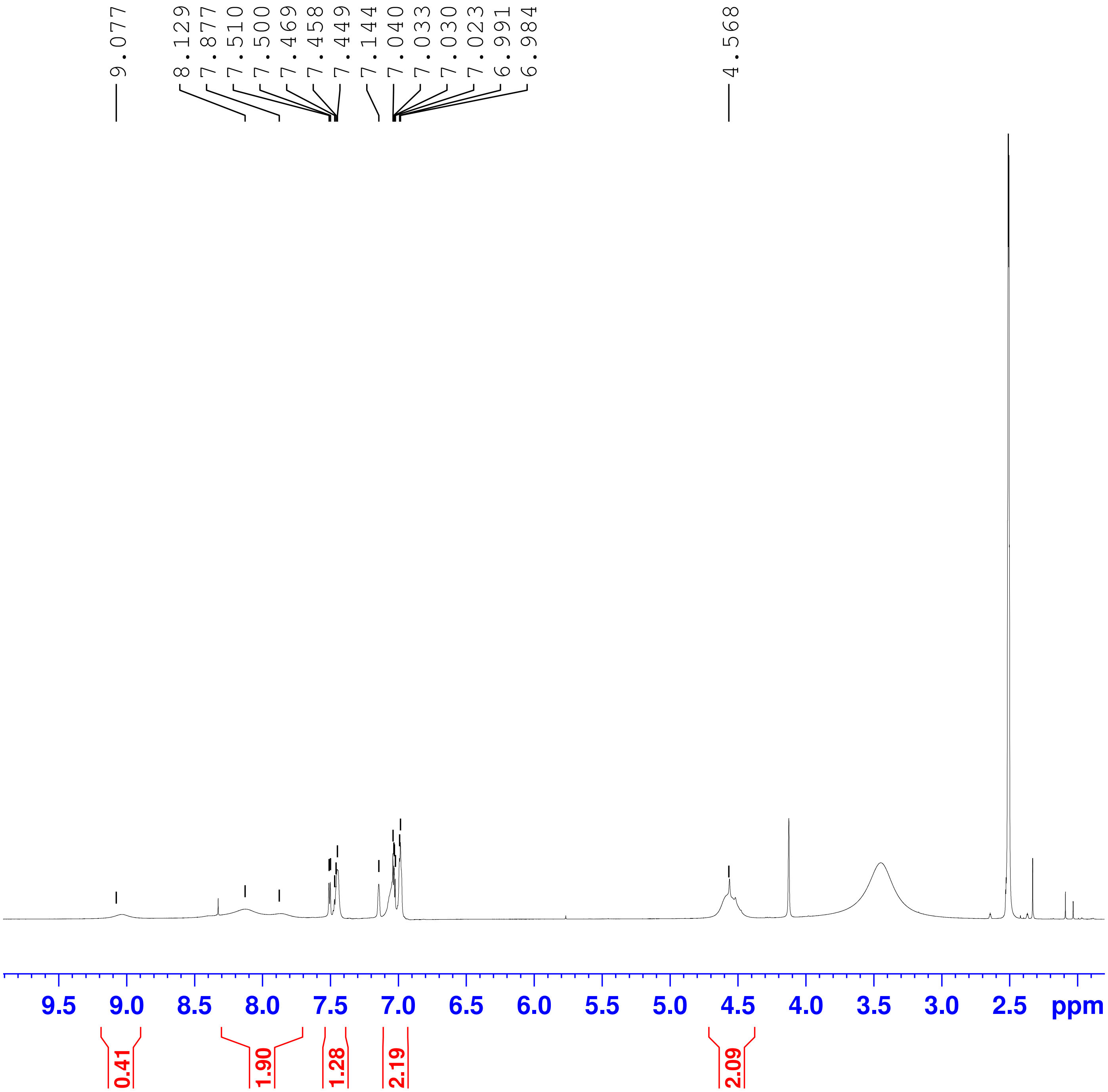


```
Current Data Parameters
NAME                   3
EXPNO                  2
PROCNO                 1

F2 - Acquisition Parameters
Date_           20180712
Time               18.33 h
INSTRUM            spect
PROBHD   Z119470_0187 (
PULPROG             zg30
TD                 65536
SOLVENT             DMSO
NS                    16
DS                     2
SWH            10000.000 Hz
FIDRES          0.305176 Hz
AQ             3.2767999 sec
RG                 88.37
DW                50.000 usec
DE                 12.72 usec
TE                 287.5 K
D1            5.00000000 sec
TD0                    1
SFO1         500.1830886 MHz
NUC1                  1H
P0                  3.33 usec
P1                 10.00 usec
PLW1         18.10400009 W

F2 - Processing parameters
SI                 65536
SF           500.1800000 MHz
WDW                   EM
SSB                    0
LB                  0.30 Hz
GB                     0
PC                  1.00
```

^{1}H NMR (500 MHz, DMSO-d_6) of **3**

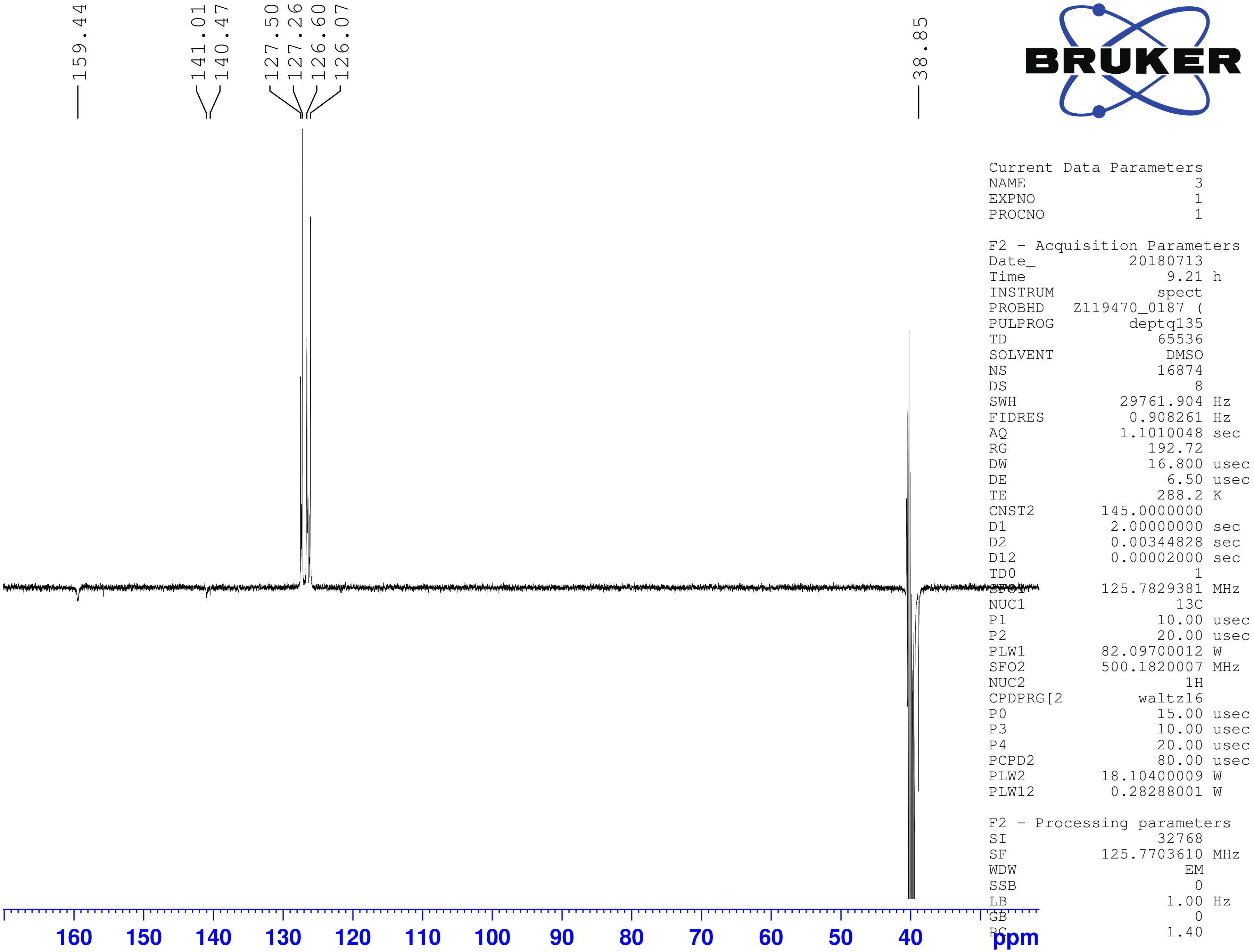

^{13}C (DEPTQ135) NMR (125 MHz, DMSO-d_6) of **3**

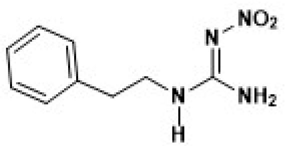

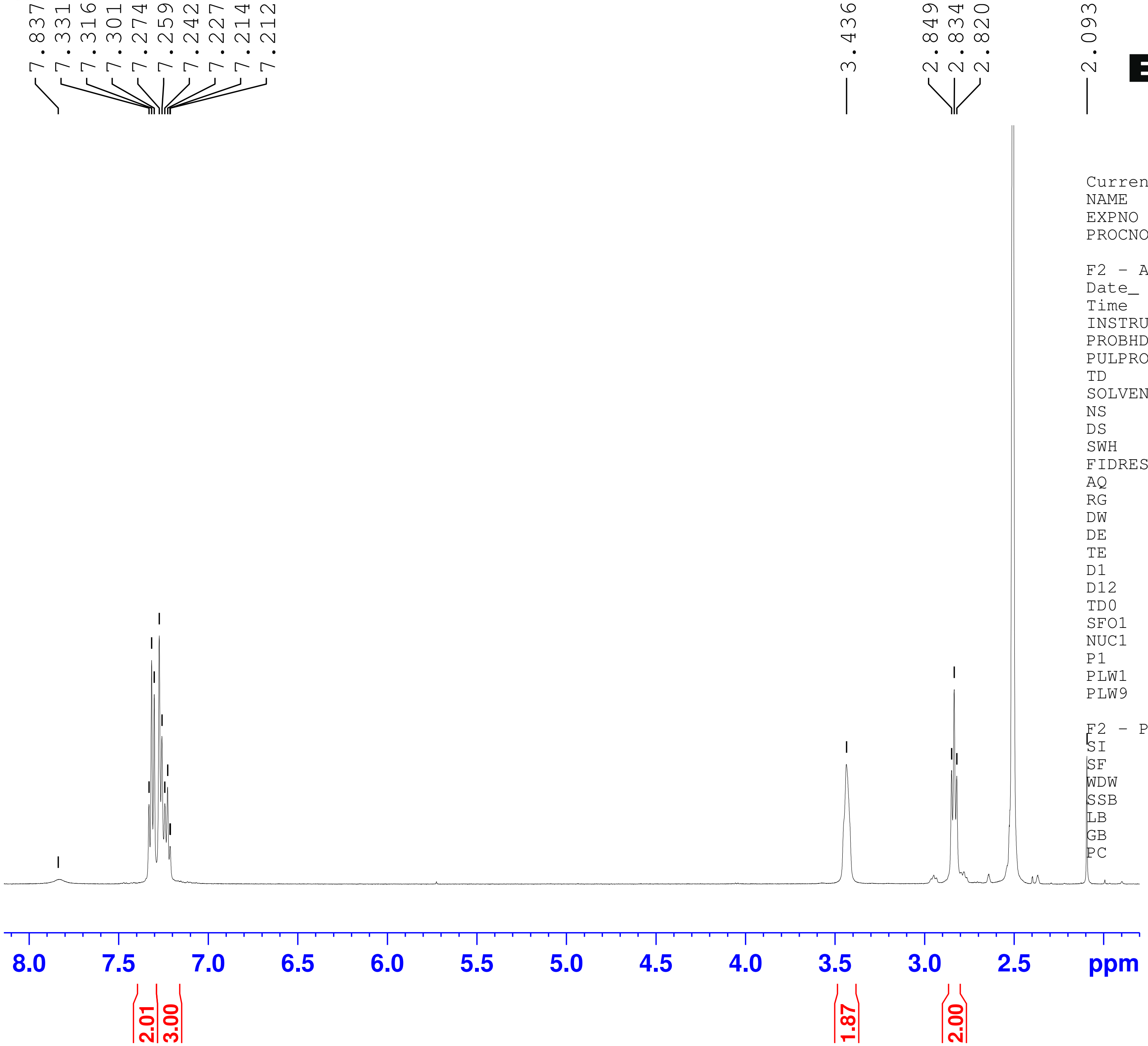


```
Current Data Parameters
NAME                    4
EXPNO                   3
PROCNO                  1

F2 - Acquisition Parameters
Date_            20180703
Time                12.11 h
INSTRUM             spect
PROBHD   Z119470_0187 (
PULPROG           zgcppr
TD                  32768
SOLVENT              DMSO
NS                      8
DS                      4
SWH              8012.820 Hz
FIDRES           0.489064 Hz
AQ              2.0447233 sec
RG                 123.82
DW                 62.400 usec
DE                   6.50 usec
TE                  323.2 K
D1             2.00000000 sec
D12            0.00002000 sec
TD0                     1
SFO1          500.1816031 MHz
NUC1                   1H
P1                  10.00 usec
PLW1          18.10400009 W
PLW9           0.00007242 W

F2 - Processing parameters
SI                  32768
SF            500.1800000 MHz
WDW                    EM
SSB                     0
LB                   0.30 Hz
GB                      0
PC                   1.00
```

^{1}H NMR (500 MHz, DMSO-d_6, 50°C) of **4**

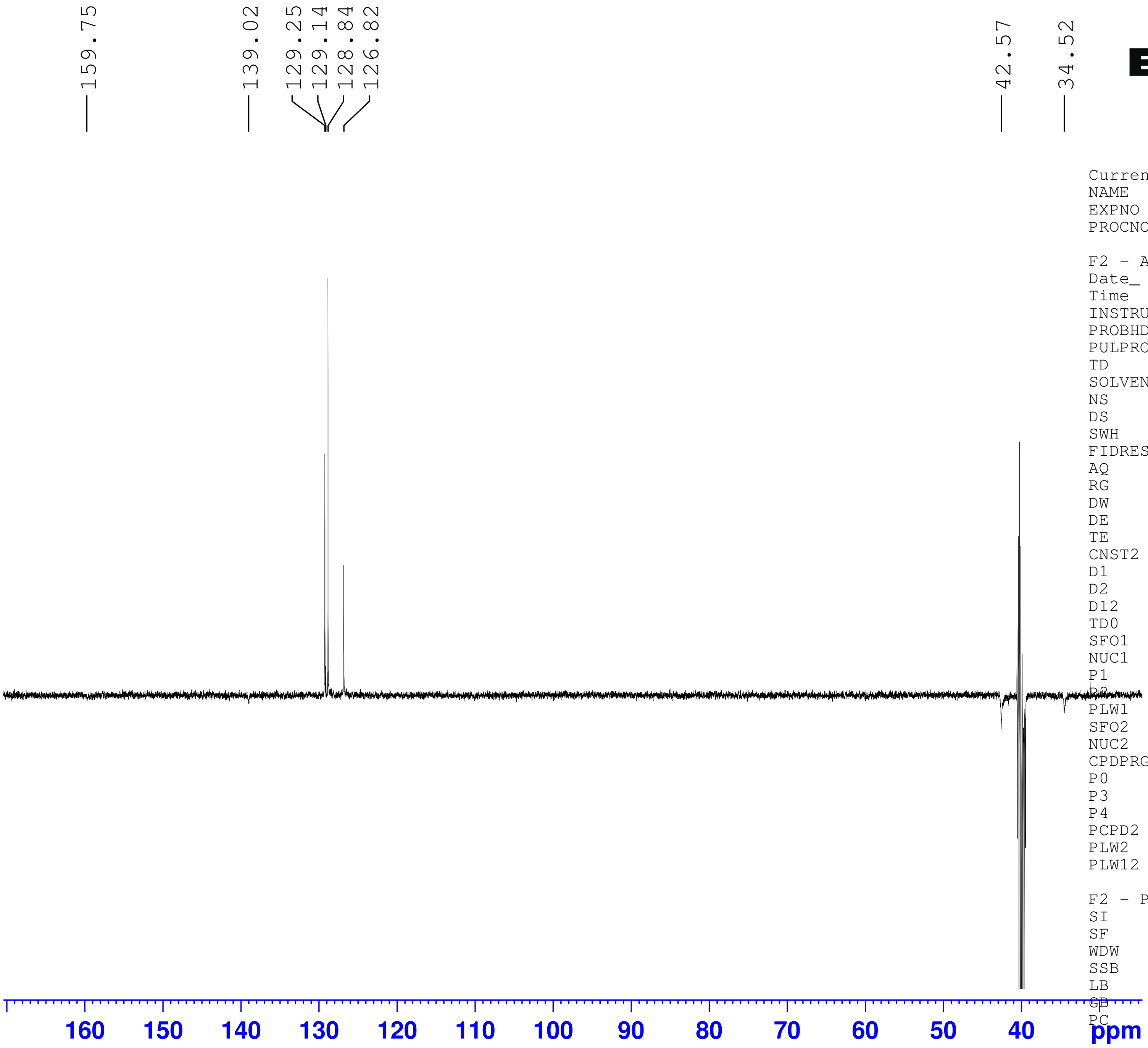

Current Data Parameters

NAME	4	
EXPNO	4	
PROCNO	1	

F2 - Acquisition Parameters

Date_	20180704	
Time	12.07	h
INSTRUM	spect	
PROBHD	Z119470_0187 (	
PULPROG	deptq135	
TD	65536	
SOLVENT	DMSO	
NS	2422	
DS	8	
SWH	29761.904	Hz
FIDRES	0.908261	Hz
AQ	1.1010048	sec
RG	192.72	
DW	16.800	usec
DE	6.50	usec
TE	291.4	K
CNST2	145.0000000	
D1	2.00000000	sec
D2	0.00344828	sec
D12	0.00002000	sec
TD0	1	
SFO1	125.7829381	MHz
NUC1	13C	
P1	10.00	usec
P2	20.00	usec
PLW1	82.09700012	W
SFO2	500.1820007	MHz
NUC2	1H	
CPDPRG[2	waltz16	
P0	15.00	usec
P3	10.00	usec
P4	20.00	usec
PCPD2	80.00	usec
PLW2	18.10400009	W
PLW12	0.28288001	W

F2 - Processing parameters

SI	32768	
SF	125.7703610	MHz
WDW	EM	
SSB	0	
LB	1.00	Hz
GB	0	
PC	1.40	

^{13}C (DEPTQ135) NMR (125 MHz, DMSO-d_6) of **4**

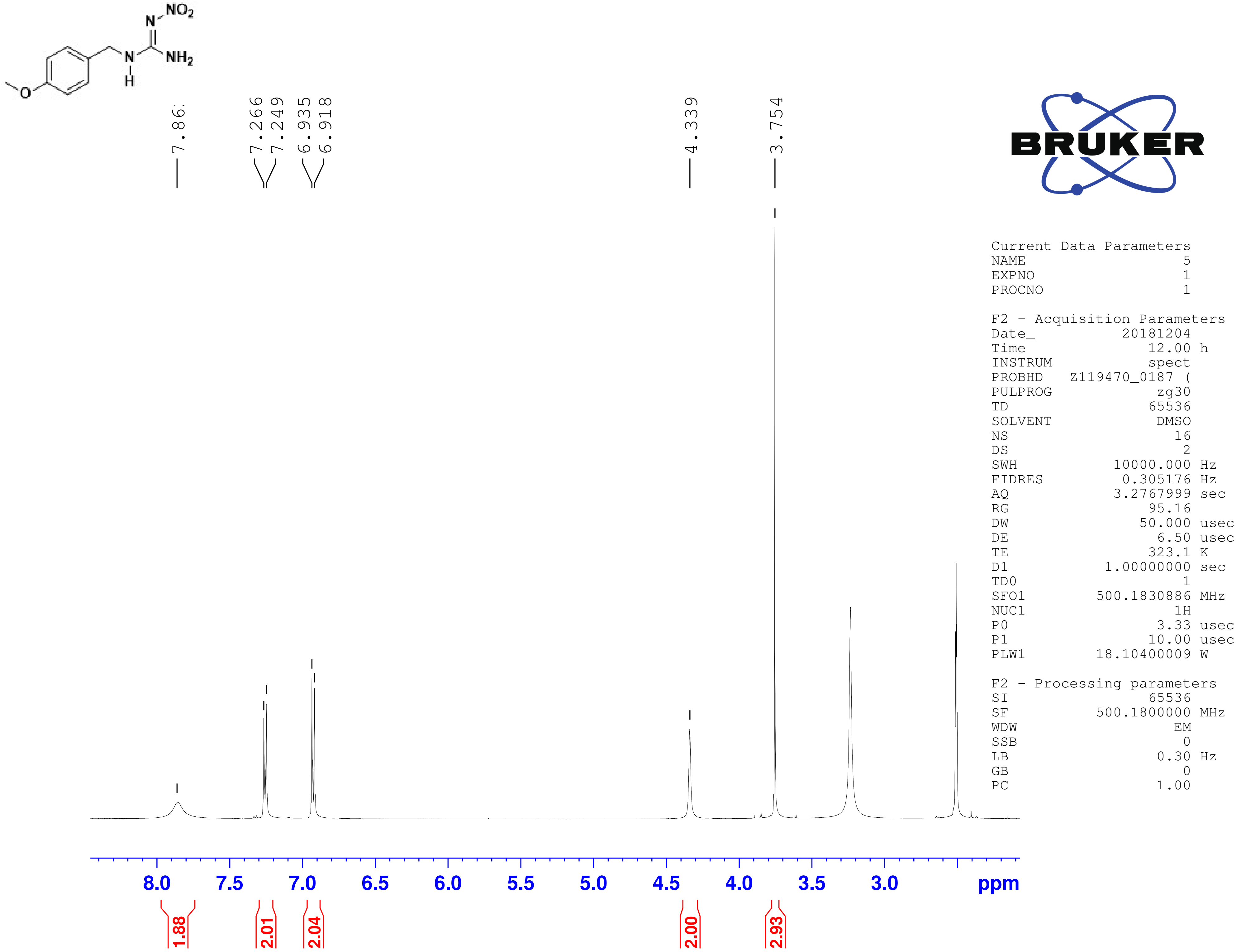

^{1}H NMR (500 MHz, DMSO-d_6, 50°C) of **5**

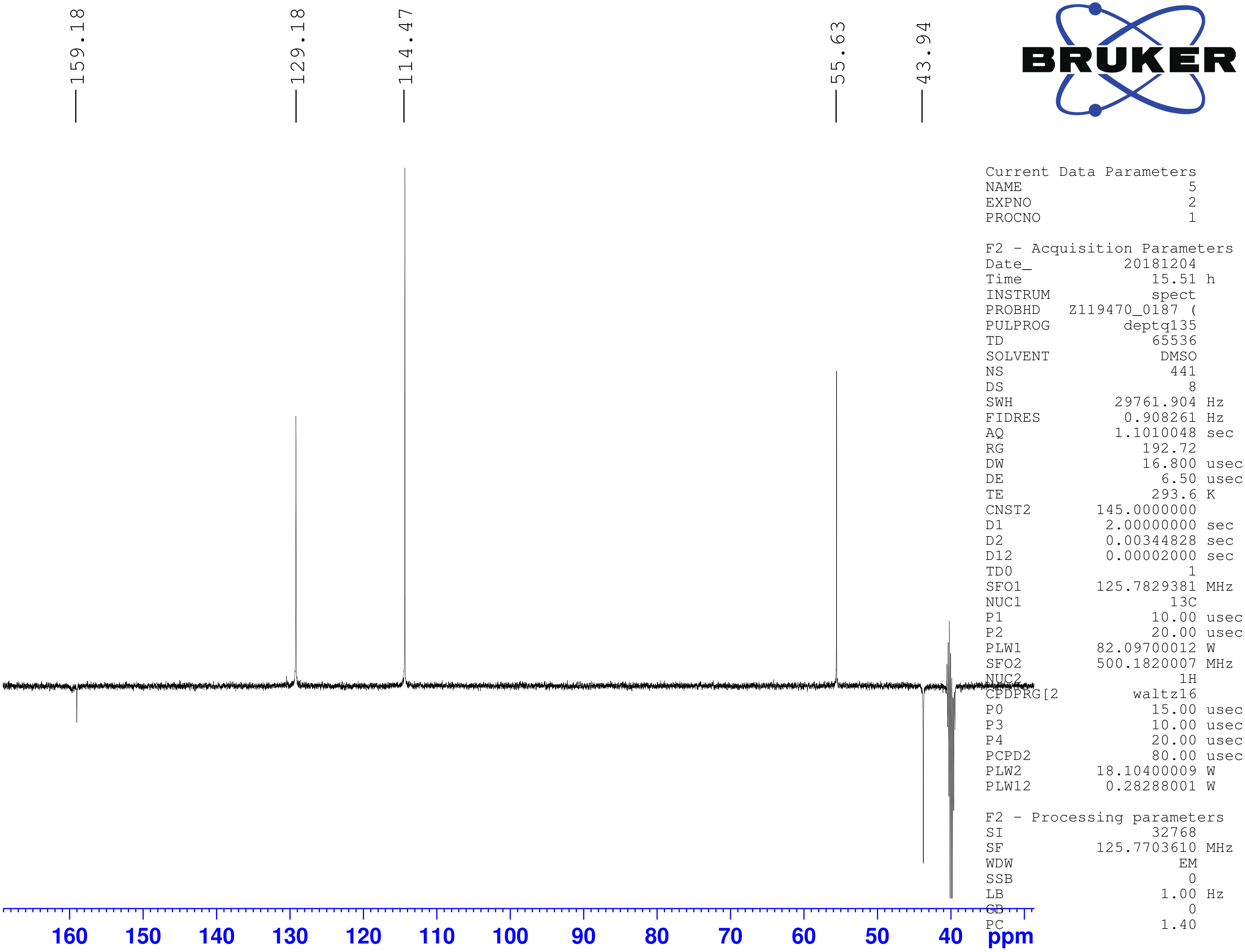

^{13}C (DEPTQ135) NMR (125 MHz, DMSO-d_6, 50°C) of **5**

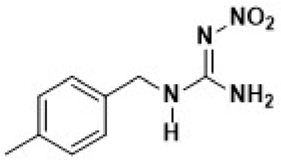

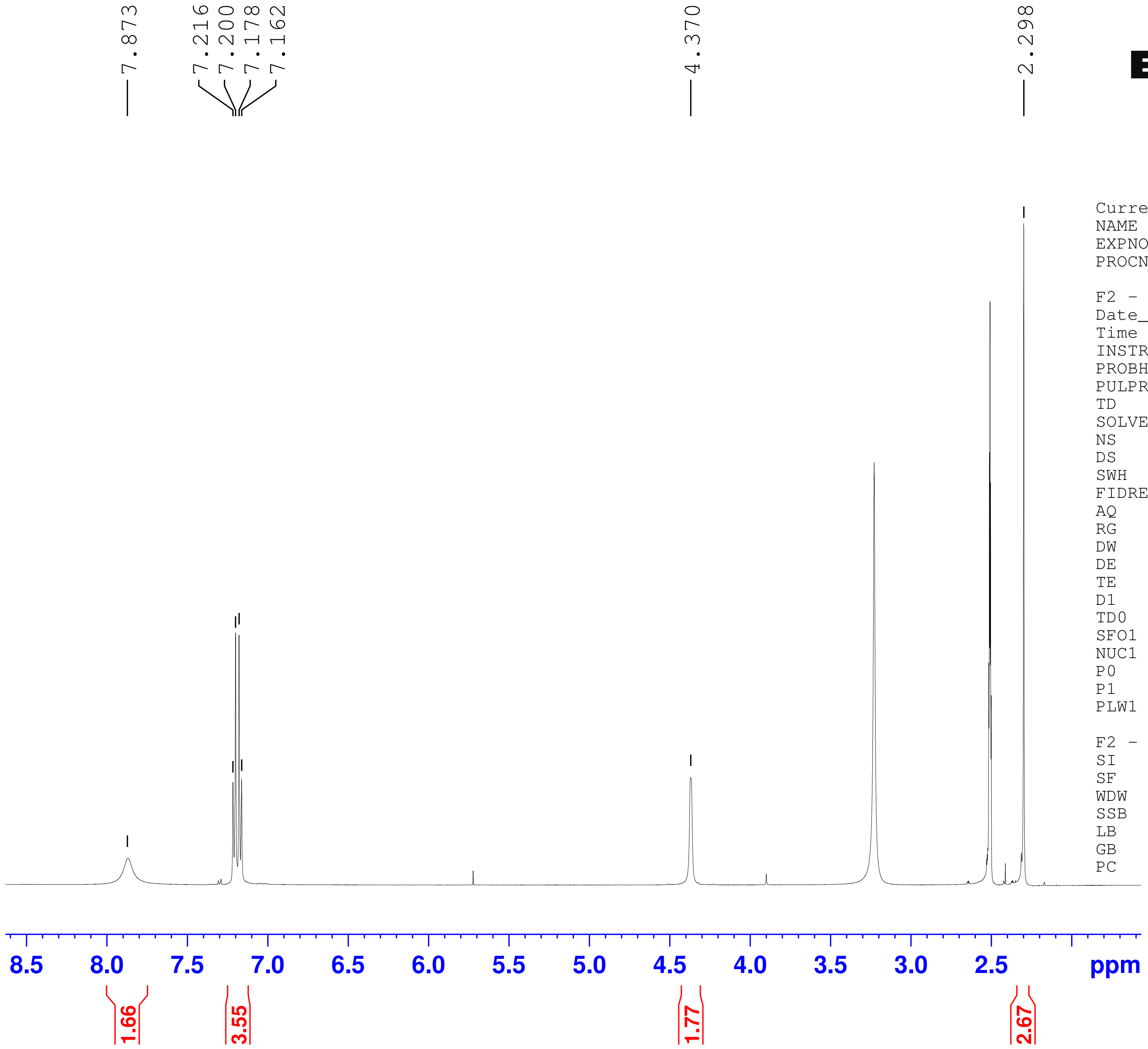


```
Current Data Parameters
NAME                    6
EXPNO                   3
PROCNO                  1

F2 - Acquisition Parameters
Date_            20181204
Time                10.07 h
INSTRUM             spect
PROBHD   Z119470_0187 (
PULPROG              zg30
TD                  65536
SOLVENT              DMSO
NS                     16
DS                      2
SWH             10000.000 Hz
FIDRES           0.305176 Hz
AQ              3.2767999 sec
RG                  108.3
DW                 50.000 usec
DE                   6.50 usec
TE                  323.2 K
D1             1.00000000 sec
TD0                     1
SFO1          500.1830886 MHz
NUC1                   1H
P0                   3.33 usec
P1                  10.00 usec
PLW1          18.10400009 W

F2 - Processing parameters
SI                  65536
SF            500.1800000 MHz
WDW                    EM
SSB                     0
LB                   0.30 Hz
GB                      0
PC                   1.00
```

^{1}H NMR (500 MHz, DMSO-d_6, 50°C) of **6**

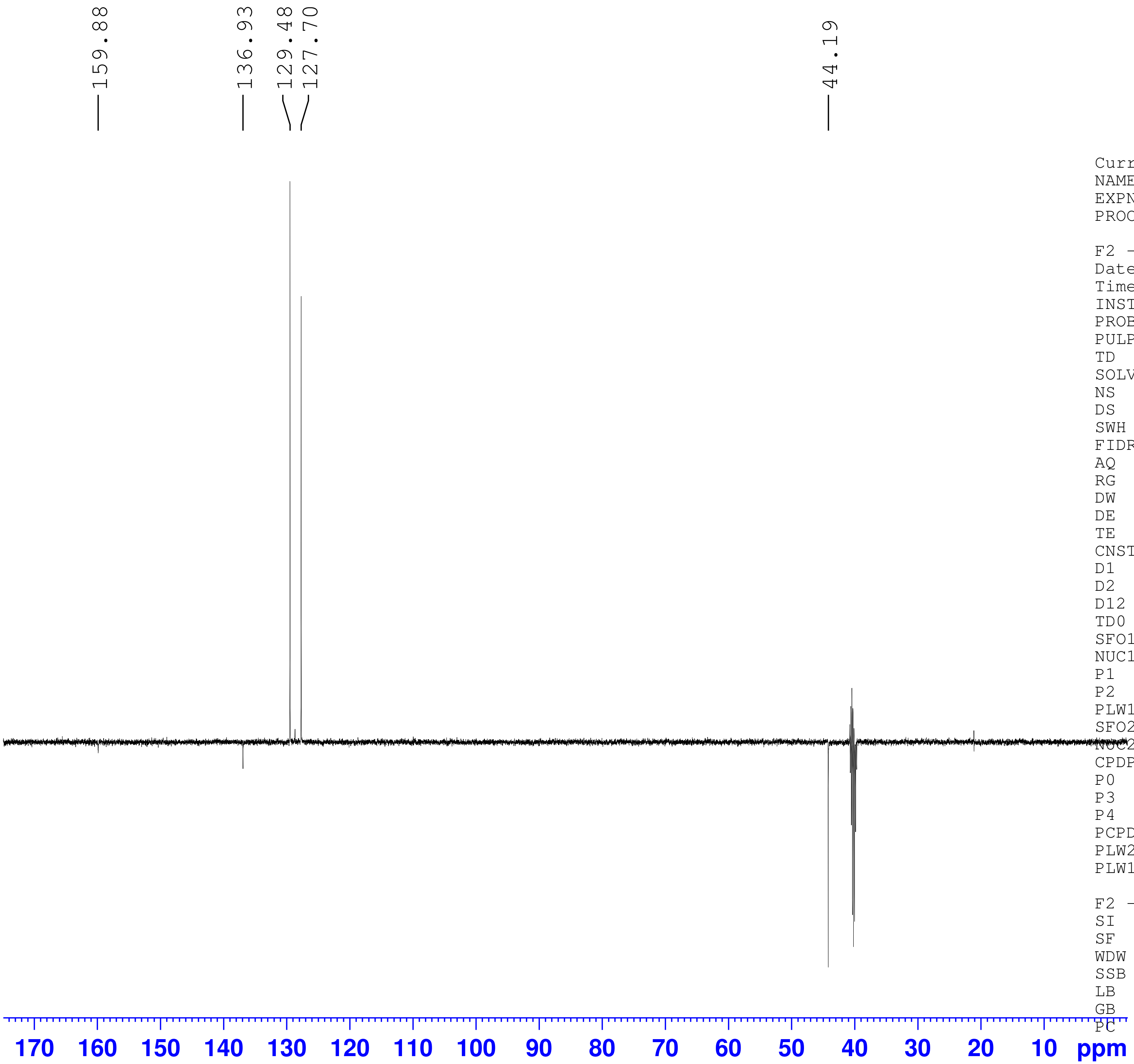

Current Data Parameters
NAME 6
EXPNO 2
PROCNO 1

F2 - Acquisition Parameters
Date_ 20181204
Time 10.28 h
INSTRUM spect
PROBHD Z119470_0187 (
PULPROG deptq135
TD 65536
SOLVENT DMSO
NS 366
DS 8
SWH 29761.904 Hz
FIDRES 0.908261 Hz
AQ 1.1010048 sec
RG 192.72
DW 16.800 usec
DE 6.50 usec
TE 323.2 K
CNST2 145.0000000
D1 2.00000000 sec
D2 0.00344828 sec
D12 0.00002000 sec
TD0 1
SFO1 125.7829381 MHz
NUC1 13C
P1 10.00 usec
P2 20.00 usec
PLW1 82.09700012 W
SFO2 500.1820007 MHz
NUC2 1H
CPDPRG[2 waltz16
P0 15.00 usec
P3 10.00 usec
P4 20.00 usec
PCPD2 80.00 usec
PLW2 18.10400009 W
PLW12 0.28288001 W

F2 - Processing parameters
SI 32768
SF 125.7703610 MHz
WDW EM
SSB 0
LB 1.00 Hz
GB 0
PC 1.40

^{13}C (DEPTQ135) NMR (125 MHz, DMSO-d_6, 50°C) of **6**

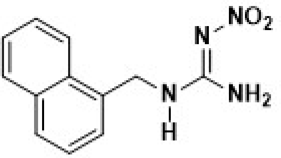

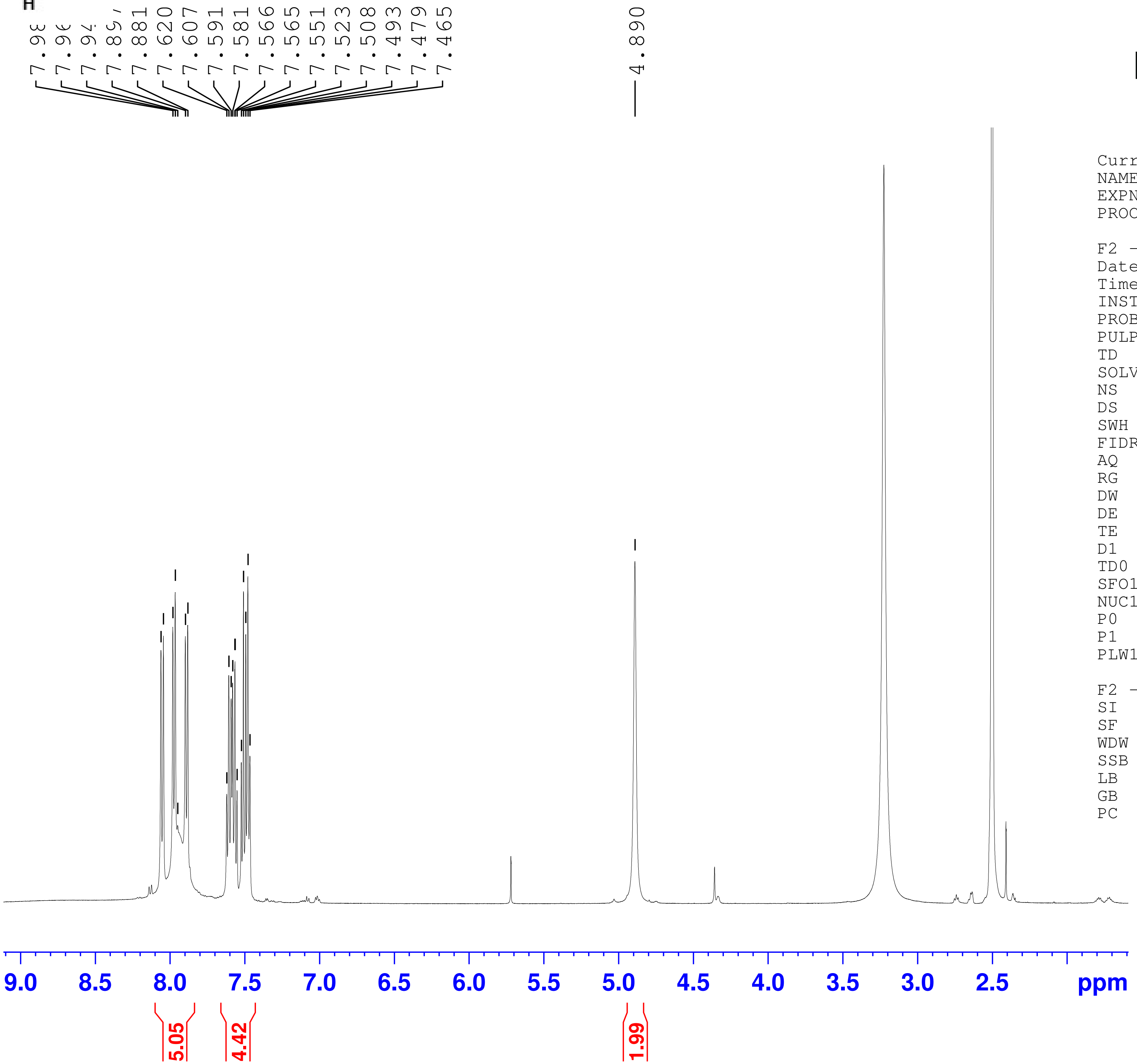


```
Current Data Parameters
NAME                        7
EXPNO                       1
PROCNO                      1

F2 - Acquisition Parameters
Date_              20181204
Time                  13.21 h
INSTRUM               spect
PROBHD   Z119470_0187 (
PULPROG                zg30
TD                    65536
SOLVENT                DMSO
NS                       16
DS                        2
SWH               10000.000 Hz
FIDRES             0.305176 Hz
AQ                3.2767999 sec
RG                   139.09
DW                   50.000 usec
DE                     6.50 usec
TE                    323.2 K
D1               1.00000000 sec
TD0                       1
SFO1            500.1830886 MHz
NUC1                     1H
P0                     3.33 usec
P1                    10.00 usec
PLW1            18.10400009 W

F2 - Processing parameters
SI                    65536
SF              500.1800000 MHz
WDW                      EM
SSB                       0
LB                     0.30 Hz
GB                        0
PC                     1.00
```


^{1}H NMR (500 MHz, DMSO-d_6, 50°C) of **7**

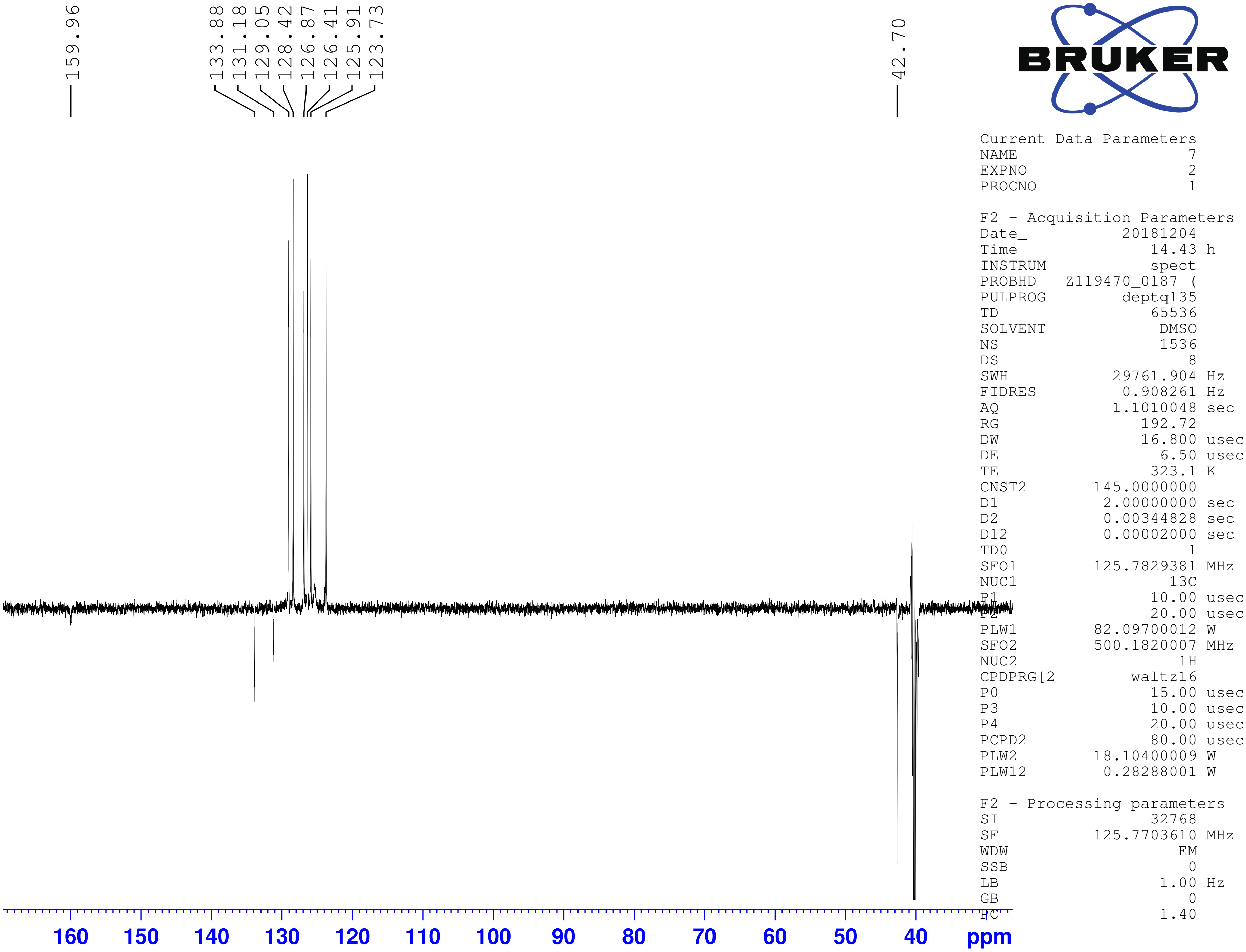

^{13}C (DEPTQ135) NMR (125 MHz, DMSO-d_6, 50°C) of **7**

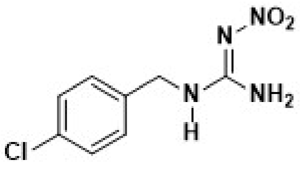

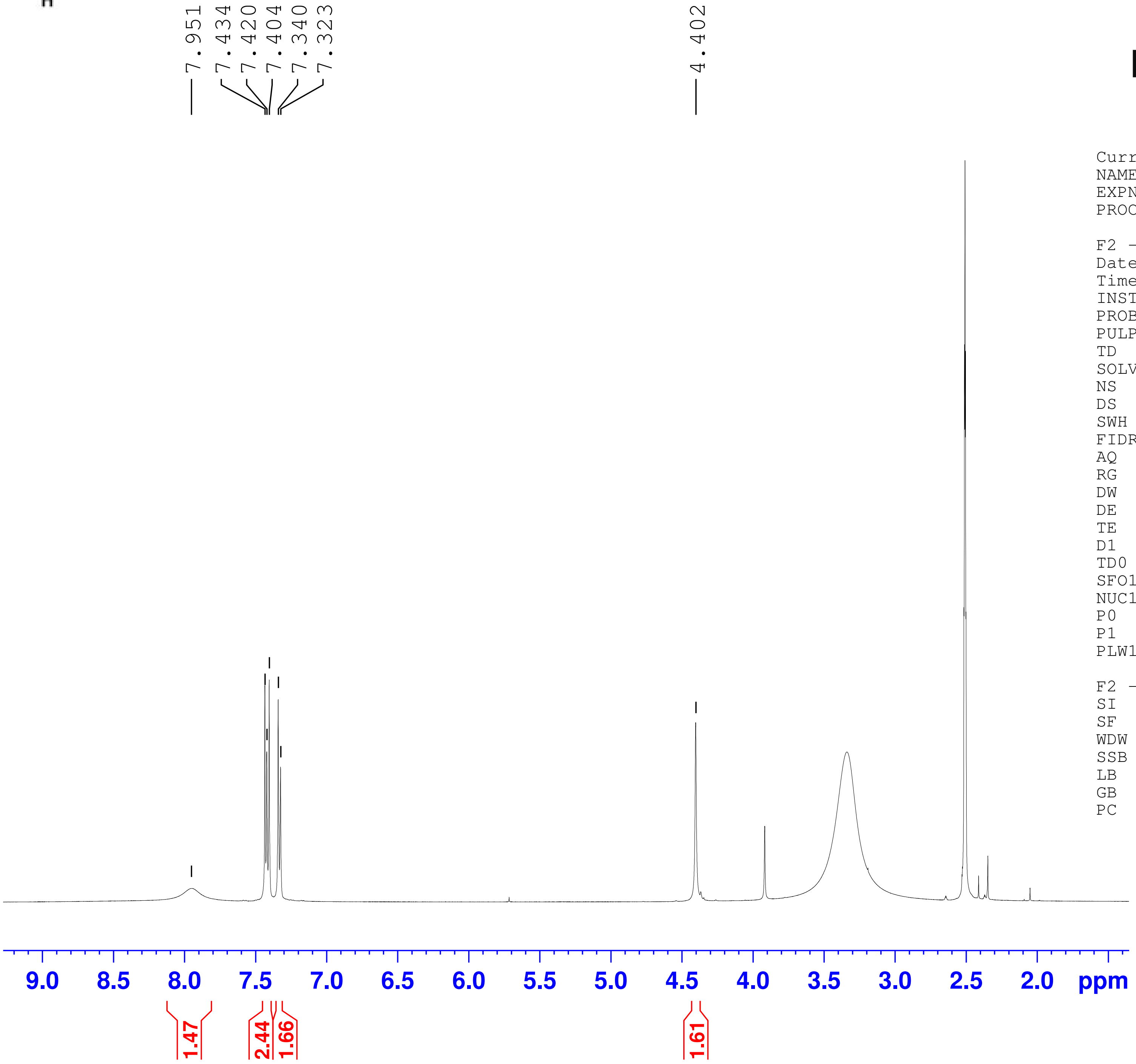


```
Current Data Parameters
NAME                 8
EXPNO                1
PROCNO               1

F2 - Acquisition Parameters
Date_          20181204
Time               9.21 h
INSTRUM           spect
PROBHD   Z119470_0187 (
PULPROG            zg30
TD                65536
SOLVENT            DMSO
NS                   16
DS                    2
SWH           10000.000 Hz
FIDRES         0.305176 Hz
AQ            3.2767999 sec
RG                95.16
DW               50.000 usec
DE                 6.50 usec
TE                323.2 K
D1           1.00000000 sec
TD0                   1
SFO1        500.1830886 MHz
NUC1                 1H
P0                 3.33 usec
P1                10.00 usec
PLW1        18.10400009 W

F2 - Processing parameters
SI                65536
SF          500.1800000 MHz
WDW                  EM
SSB                   0
LB                 0.30 Hz
GB                    0
PC                 1.00
```

^{1}H NMR (500 MHz, DMSO-d_6, 50°C) of **8**

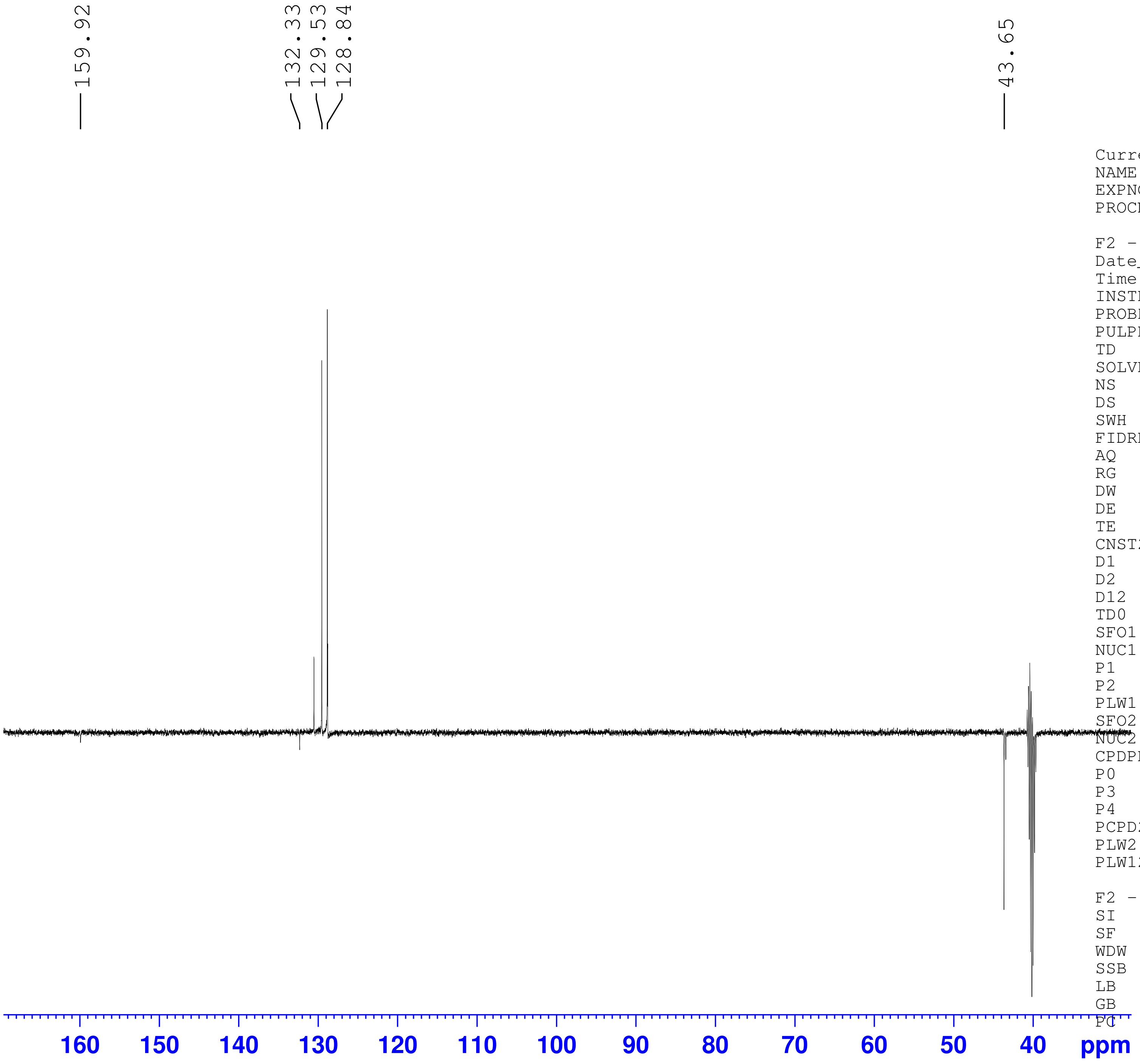


```
Current Data Parameters
NAME                    8
EXPNO                   2
PROCNO                  1

F2 - Acquisition Parameters
Date_            20181204
Time                 9.54 h
INSTRUM             spect
PROBHD   Z119470_0187 (
PULPROG          deptq135
TD                  65536
SOLVENT              DMSO
NS                    578
DS                      8
SWH             29761.904 Hz
FIDRES           0.908261 Hz
AQ              1.1010048 sec
RG                 192.72
DW                 16.800 usec
DE                   6.50 usec
TE                  323.1 K
CNST2         145.0000000
D1             2.00000000 sec
D2             0.00344828 sec
D12            0.00002000 sec
TD0                     1
SFO1          125.7829381 MHz
NUC1                  13C
P1                  10.00 usec
P2                  20.00 usec
PLW1          82.09700012 W
SFO2          500.1820007 MHz
NUC2                   1H
CPDPRG[2          waltz16
P0                  15.00 usec
P3                  10.00 usec
P4                  20.00 usec
PCPD2               80.00 usec
PLW2          18.10400009 W
PLW12          0.28288001 W

F2 - Processing parameters
SI                  32768
SF            125.7703610 MHz
WDW                    EM
SSB                     0
LB                   1.00 Hz
GB                      0
PC                   1.40
```


^{13}C (DEPTQ135) NMR (125 MHz, DMSO-d_6, 50°C) of **8**

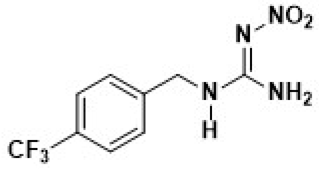

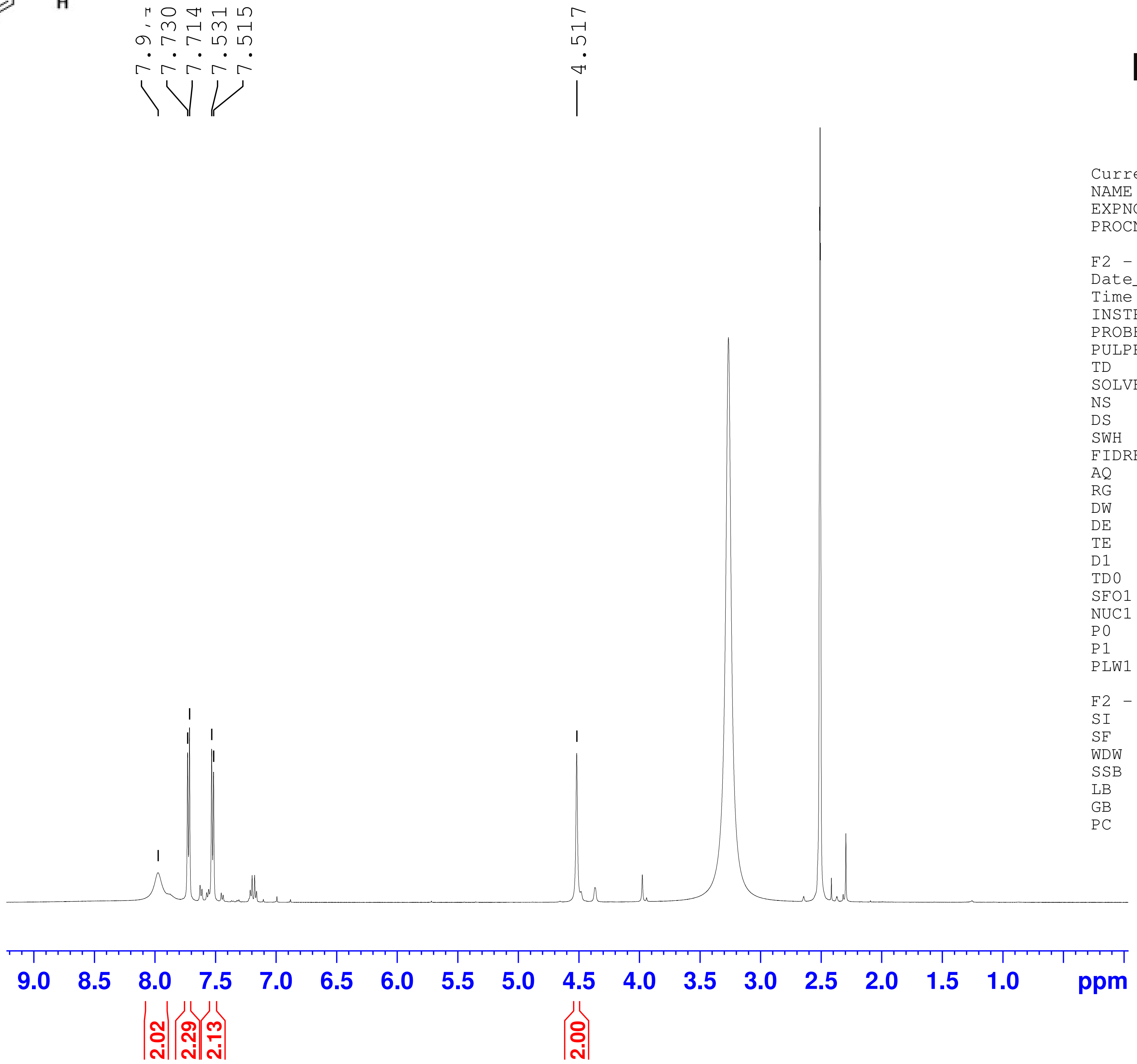

Current Data Parameters
NAME 9
EXPNO 2
PROCNO 1

F2 - Acquisition Parameters
Date_ 20180816
Time 20.01 h
INSTRUM spect
PROBHD Z119470_0187 (
PULPROG zg30
TD 65536
SOLVENT DMSO
NS 16
DS 2
SWH 10000.000 Hz
FIDRES 0.305176 Hz
AQ 3.2767999 sec
RG 88.37
DW 50.000 usec
DE 6.50 usec
TE 323.4 K
D1 1.00000000 sec
TD0 1
SFO1 500.1830886 MHz
NUC1 1H
P0 3.33 usec
P1 10.00 usec
PLW1 18.10400009 W

F2 - Processing parameters
SI 65536
SF 500.1800000 MHz
WDW EM
SSB 0
LB 0.30 Hz
GB 0
PC 1.00

^{1}H NMR (500 MHz, DMSO-d_6, 50°C) of **9**

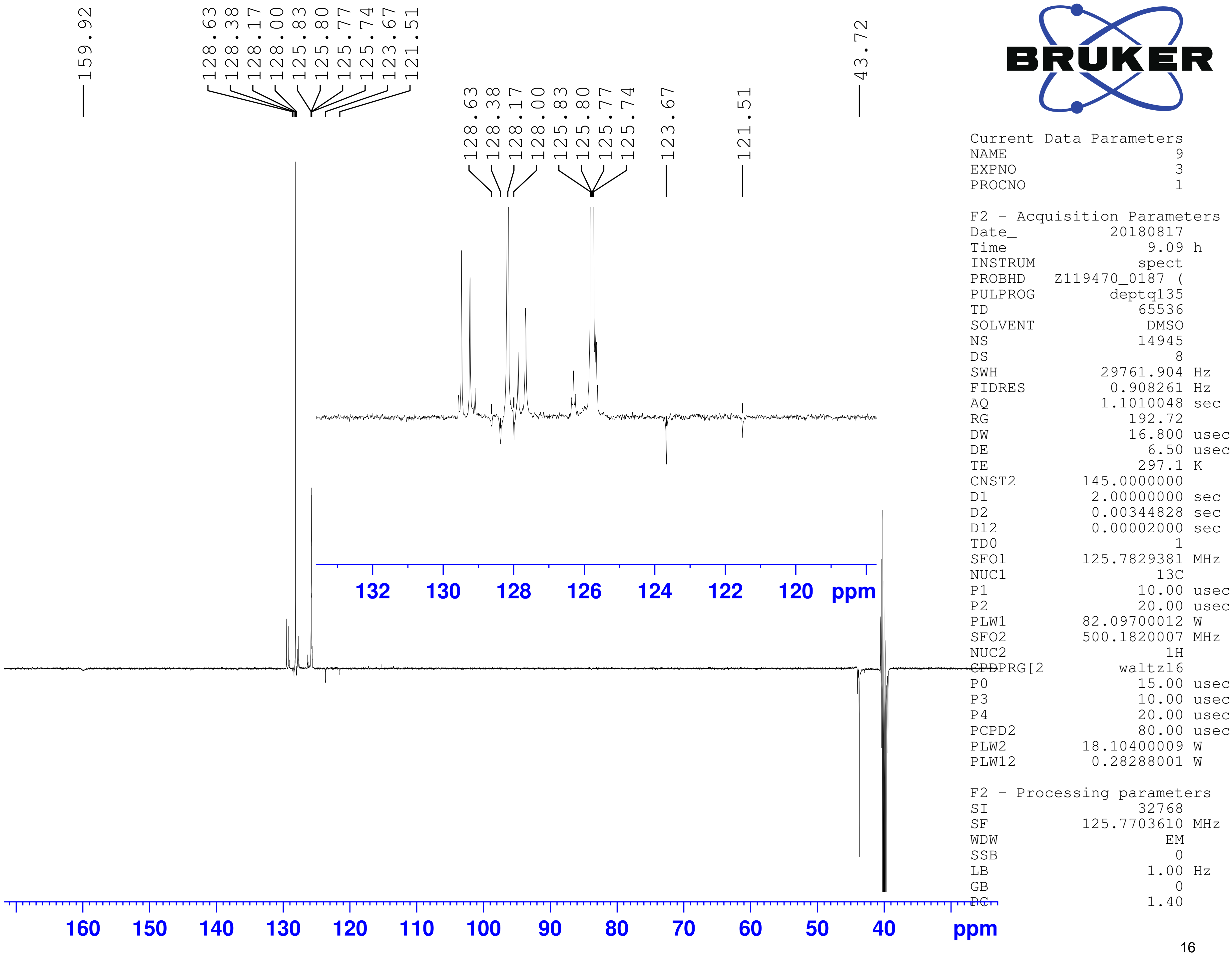

^{13}C (DEPTQ135) NMR (125 MHz, DMSO-d_6) of **9**

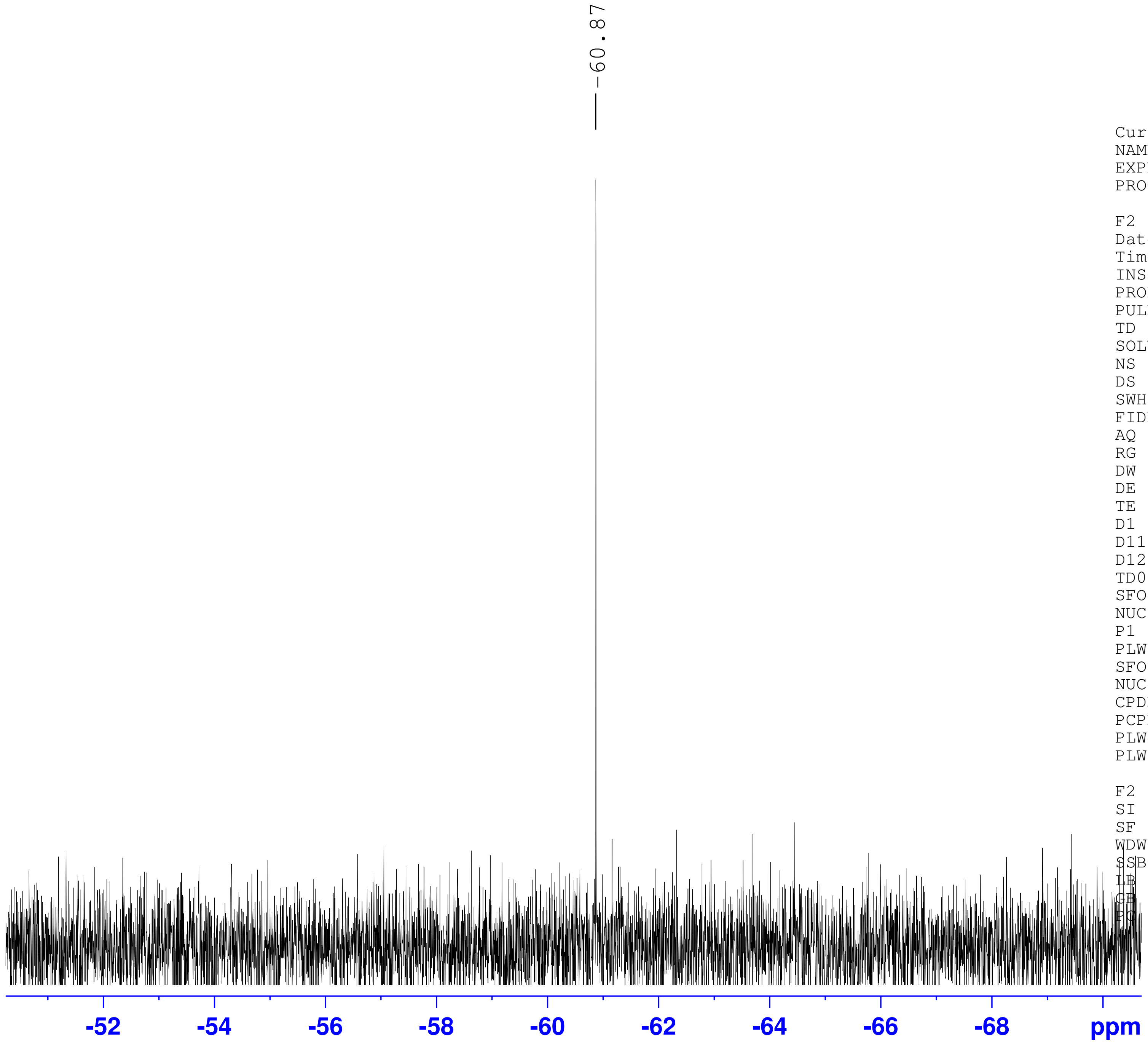

Current Data Parameters
NAME 9
EXPNO 6
PROCNO 1

F2 - Acquisition Parameters
Date_ 20190801
Time 14.44 h
INSTRUM spect
PROBHD Z119470_0187 (
PULPROG zgfhigqn.2
TD 131072
SOLVENT DMSO
NS 16
DS 4
SWH 113636.367 Hz
FIDRES 1.733953 Hz
AQ 0.5767168 sec
RG 11.27
DW 4.400 usec
DE 6.50 usec
TE 293.4 K
D1 1.00000000 sec
D11 0.03000000 sec
D12 0.00002000 sec
TD0 1
SFO1 470.5923603 MHz
NUC1 19F
P1 16.00 usec
PLW1 36.93899918 W
SFO2 500.1820007 MHz
NUC2 1H
CPDPRG[2 waltz16
PCPD2 80.00 usec
PLW2 18.10400009 W
PLW12 0.31009001 W

F2 - Processing parameters
SI 65536
SF 470.6394242 MHz
WDW EM
SSB 0
LB 0.30 Hz
GB 0
PC 1.00

^{19}F NMR (470 MHz, DMSO-d_6) of **9**

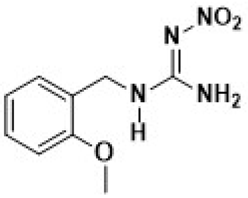

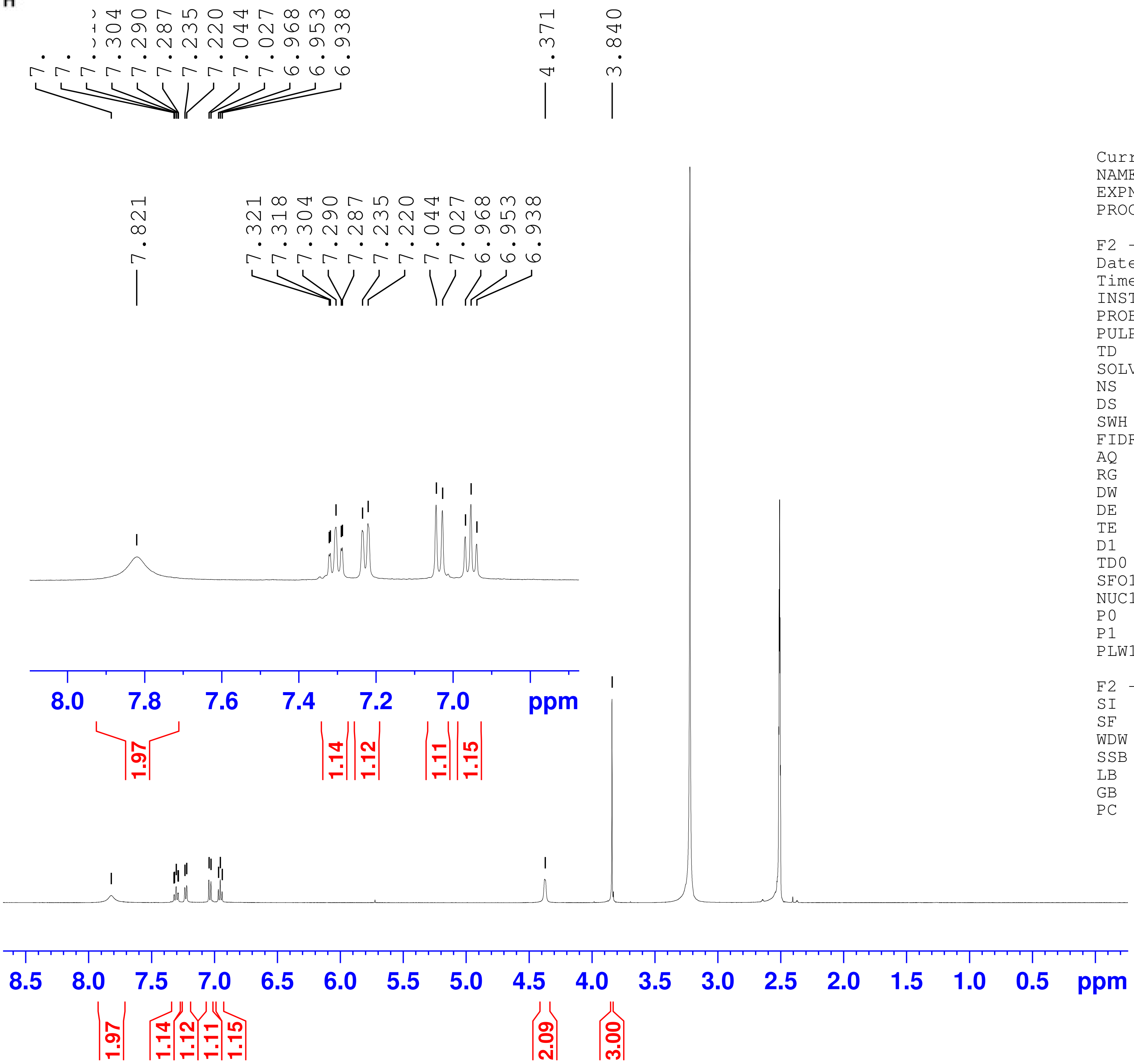


```
Current Data Parameters
NAME                    10
EXPNO                    1
PROCNO                   1

F2 - Acquisition Parameters
Date_             20180817
Time                 11.06 h
INSTRUM              spect
PROBHD    Z119470_0187 (
PULPROG               zg30
TD                   65536
SOLVENT               DMSO
NS                       5
DS                       1
SWH              10000.000 Hz
FIDRES            0.305176 Hz
AQ               3.2767999 sec
RG                  139.09
DW                  50.000 usec
DE                    6.50 usec
TE                   323.1 K
D1              1.00000000 sec
TD0                      1
SFO1           500.1830886 MHz
NUC1                    1H
P0                    3.33 usec
P1                   10.00 usec
PLW1           18.10400009 W

F2 - Processing parameters
SI                   65536
SF             500.1800000 MHz
WDW                     EM
SSB                      0
LB                    0.30 Hz
GB                       0
PC                    1.00
```


^{1}H NMR (500 MHz, DMSO-d$_6$, 50°C) of **10**

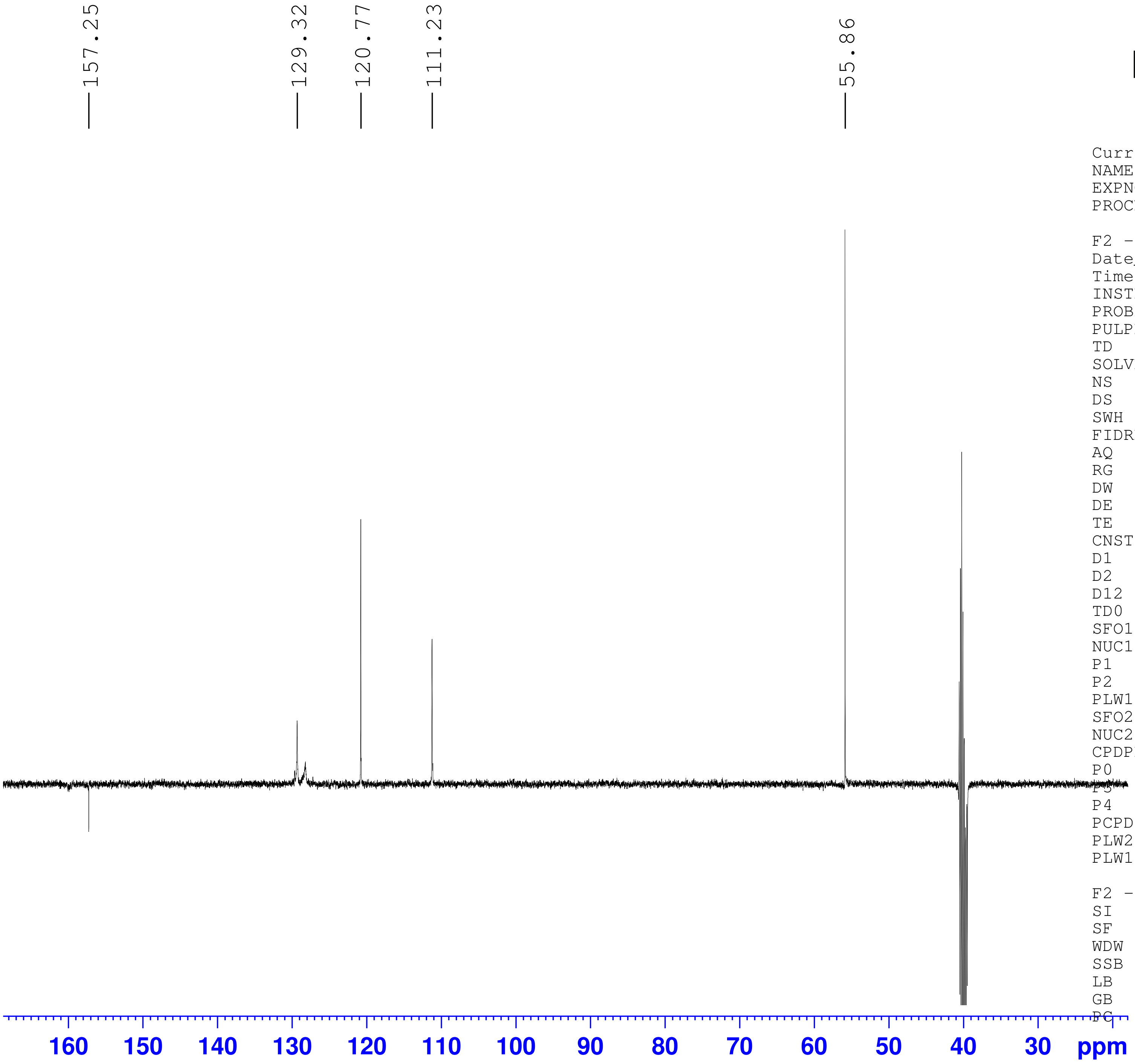

Current Data Parameters
NAME 10
EXPNO 2
PROCNO 1

F2 - Acquisition Parameters
Date_ 20180817
Time 15.58 h
INSTRUM spect
PROBHD Z119470_0187 (
PULPROG deptq135
TD 65536
SOLVENT DMSO
NS 5120
DS 8
SWH 29761.904 Hz
FIDRES 0.908261 Hz
AQ 1.1010048 sec
RG 192.72
DW 16.800 usec
DE 6.50 usec
TE 290.9 K
CNST2 145.0000000
D1 2.00000000 sec
D2 0.00344828 sec
D12 0.00002000 sec
TD0 1
SFO1 125.7829381 MHz
NUC1 13C
P1 10.00 usec
P2 20.00 usec
PLW1 82.09700012 W
SFO2 500.1820007 MHz
NUC2 1H
CPDPRG[2 waltz16
P0 15.00 usec
P3 10.00 usec
P4 20.00 usec
PCPD2 80.00 usec
PLW2 18.10400009 W
PLW12 0.28288001 W

F2 - Processing parameters
SI 32768
SF 125.7703610 MHz
WDW EM
SSB 0
LB 1.00 Hz
GB 0
PC 1.40

^{13}C (DEPTQ135) NMR (125 MHz, DMSO-d$_6$) of **10**

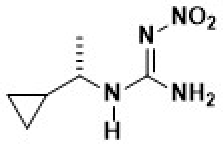

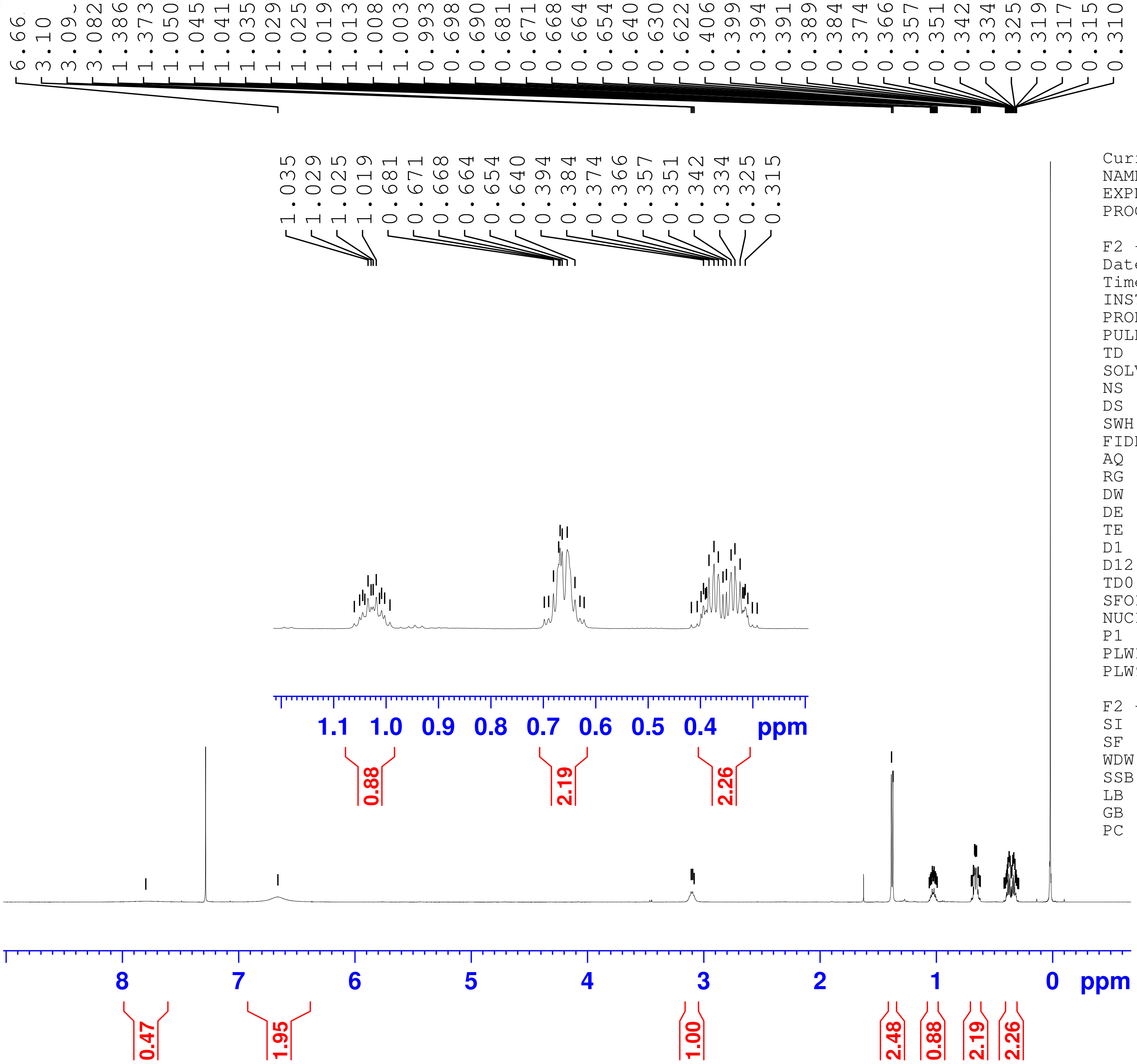

Current Data Parameters
NAME S-11
EXPNO 4
PROCNO 1

F2 - Acquisition Parameters
Date_ 20180601
Time 10.34 h
INSTRUM spect
PROBHD Z119470_0187 (
PULPROG zgcppr
TD 32768
SOLVENT CDCl3
NS 16
DS 4
SWH 8012.820 Hz
FIDRES 0.489064 Hz
AQ 2.0447233 sec
RG 139.09
DW 62.400 usec
DE 6.50 usec
TE 291.0 K
D1 2.00000000 sec
D12 0.00002000 sec
TD0 1
SFO1 500.1808034 MHz
NUC1 1H
P1 10.00 usec
PLW1 18.10400009 W
PLW9 0.00007242 W

F2 - Processing parameters
SI 32768
SF 500.1800000 MHz
WDW EM
SSB 0
LB 0.30 Hz
GB 0
PC 1.00

^{1}H NMR (500 MHz, $CDCl_3$) of ***S*-11**

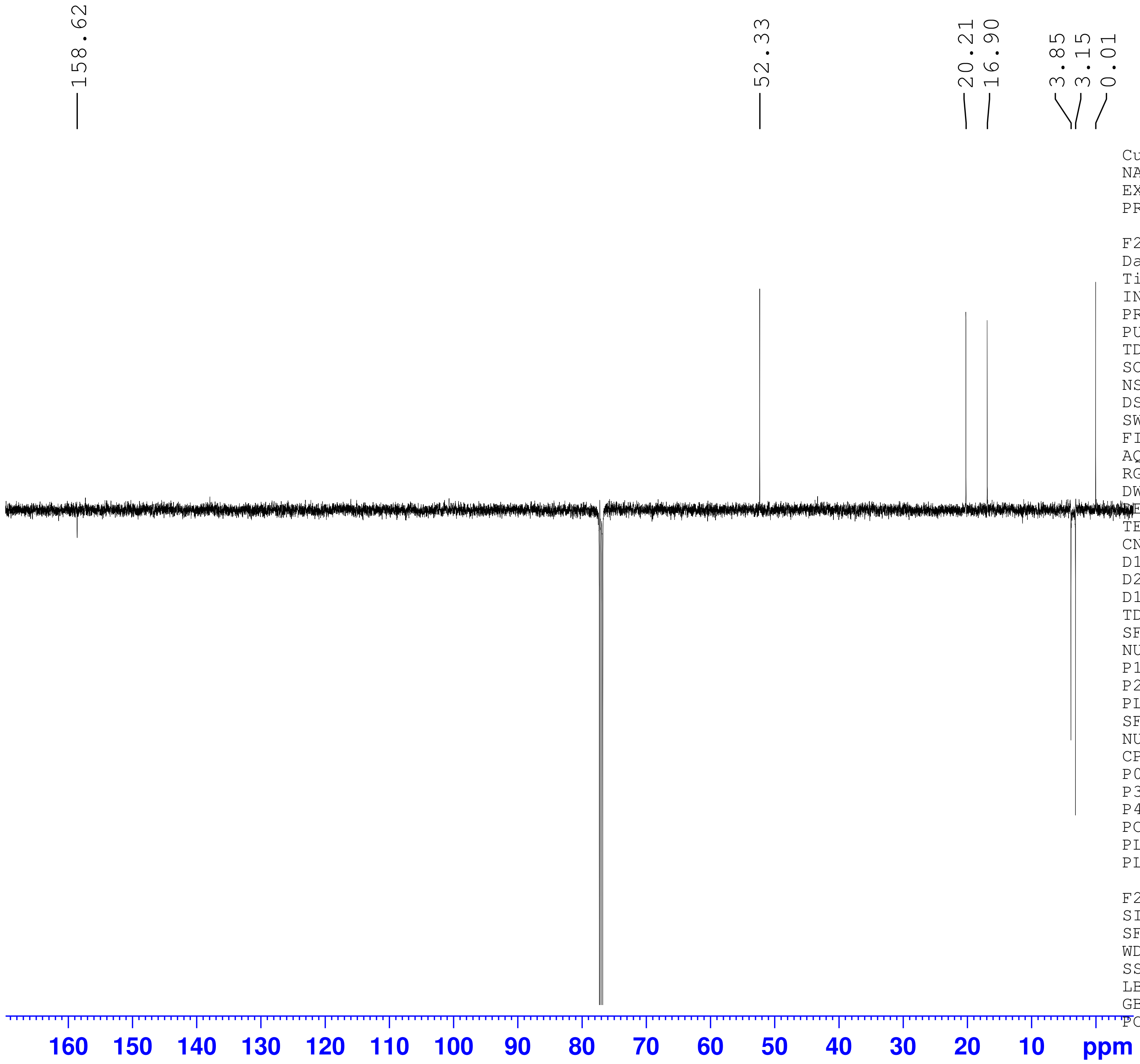


```
Current Data Parameters
NAME                  S-11
EXPNO                    3
PROCNO                   1

F2 - Acquisition Parameters
Date_             20180601
Time                 13.59 h
INSTRUM              spect
PROBHD    Z119470_0187 (
PULPROG          deptq135
TD                   65536
SOLVENT              CDCl3
NS                    3832
DS                       8
SWH              29761.904 Hz
FIDRES            0.908261 Hz
AQ               1.1010048 sec
RG                  192.72
DW                  16.800 usec
DE                    6.50 usec
TE                   291.7 K
CNST2          145.0000000
D1              2.00000000 sec
D2              0.00344828 sec
D12             0.00002000 sec
TD0                      1
SFO1           125.7829381 MHz
NUC1                   13C
P1                   10.00 usec
P2                   20.00 usec
PLW1           82.09700012 W
SFO2           500.1820007 MHz
NUC2                    1H
CPDPRG[2           waltz16
P0                   15.00 usec
P3                   10.00 usec
P4                   20.00 usec
PCPD2                80.00 usec
PLW2           18.10400009 W
PLW12           0.28288001 W

F2 - Processing parameters
SI                   32768
SF             125.7703610 MHz
WDW                     EM
SSB                      0
LB                    1.00 Hz
GB                       0
PC                    1.40
```

^{13}C (DEPTQ135) NMR (125 MHz, $CDCl_3$) of ***S*-11**

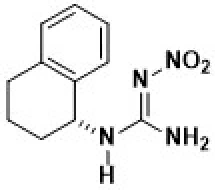

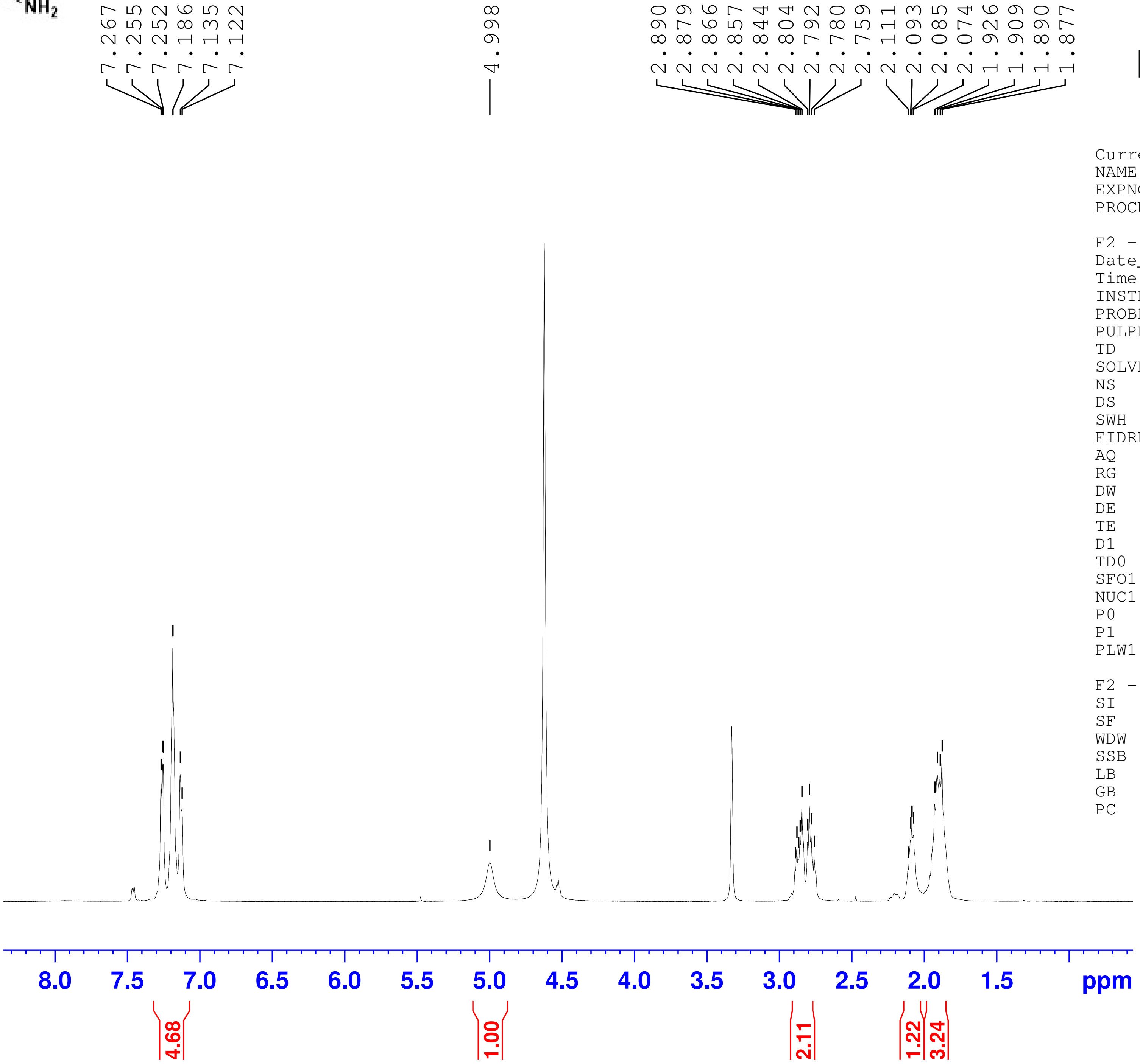

Current Data Parameters
NAME S-12
EXPNO 3
PROCNO 1

F2 - Acquisition Parameters
Date_ 20200410
Time 13.04 h
INSTRUM spect
PROBHD Z119470_0187 (
PULPROG zg30
TD 65536
SOLVENT MeOD
NS 16
DS 2
SWH 10000.000 Hz
FIDRES 0.305176 Hz
AQ 3.2767999 sec
RG 95.16
DW 50.000 usec
DE 6.50 usec
TE 323.2 K
D1 1.00000000 sec
TD0 1
SFO1 500.1830886 MHz
NUC1 1H
P0 3.49 usec
P1 10.47 usec
PLW1 18.10400009 W

F2 - Processing parameters
SI 65536
SF 500.1800000 MHz
WDW EM
SSB 0
LB 0.30 Hz
GB 0
PC 1.00

^{1}H NMR (500 MHz, CD_3OD, 50°C) of ***S-12***

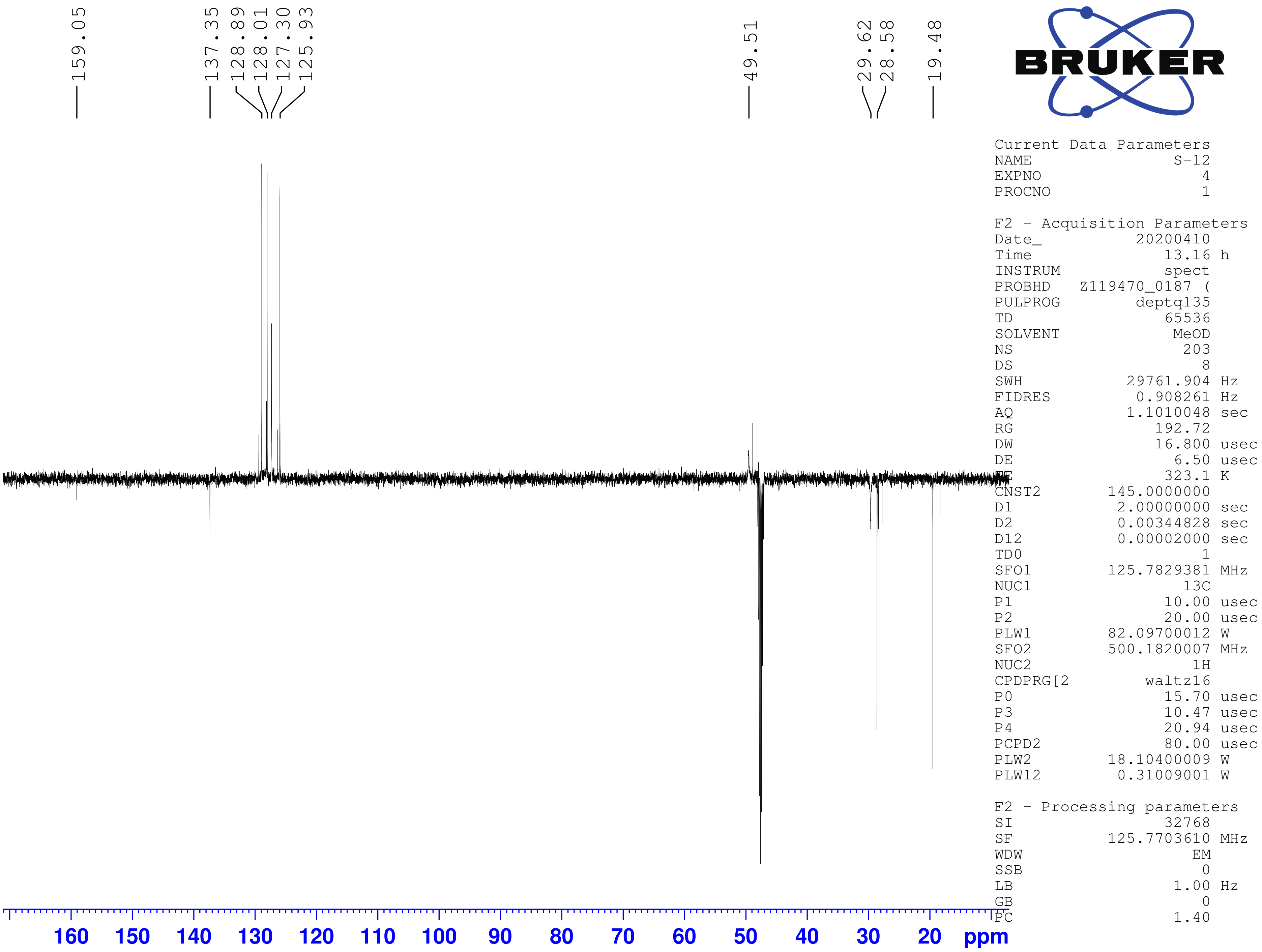

^{13}C (DEPTQ135) NMR (125 MHz, CD_3OD, 50°C) of ***S*-12**

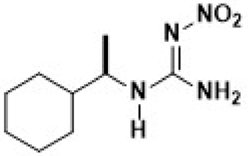

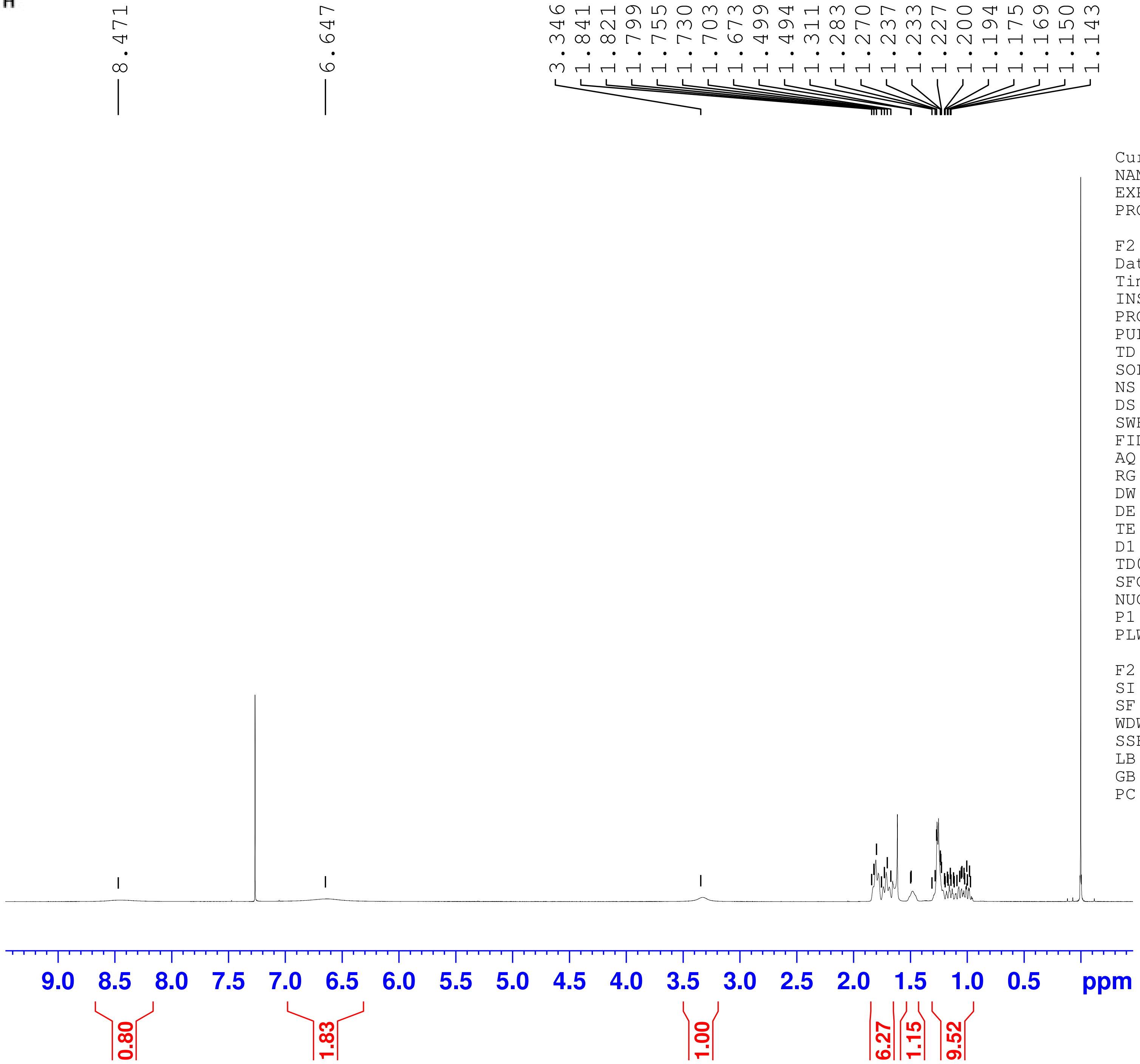


```
Current Data Parameters
NAME                  R-13
EXPNO                    1
PROCNO                   1

F2 - Acquisition Parameters
Date_             20170810
Time                 10.48 h
INSTRUM              spect
PROBHD     Z119470_0187 (
PULPROG               zg30
TD                   65536
SOLVENT              CDCl3
NS                      16
DS                       2
SWH              10000.000 Hz
FIDRES            0.305176 Hz
AQ               3.2767999 sec
RG                  139.09
DW                  50.000 usec
DE                    6.50 usec
TE                   292.0 K
D1              1.00000000 sec
TD0                      1
SFO1           500.1830886 MHz
NUC1                    1H
P1                   10.00 usec
PLW1           18.10400009 W

F2 - Processing parameters
SI                   65536
SF             500.1800094 MHz
WDW                     EM
SSB                      0
LB                    0.30 Hz
GB                       0
PC                    1.00
```

^{1}H NMR (500 MHz, $CDCl_3$) of ***R*-13**

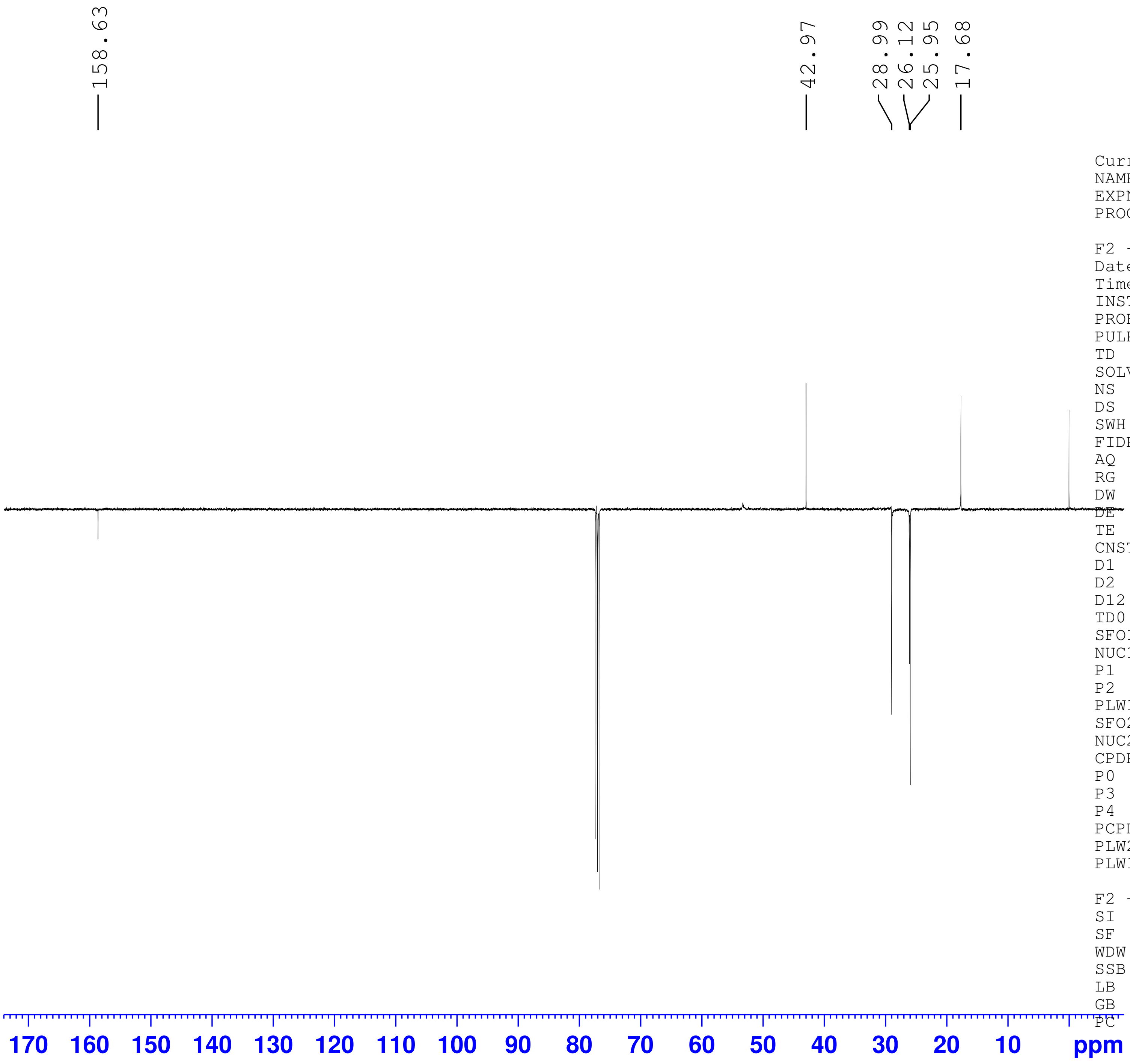


```
Current Data Parameters
NAME                 R-13
EXPNO                   2
PROCNO                  1

F2 - Acquisition Parameters
Date_            20170813
Time                 1.58 h
INSTRUM             spect
PROBHD     Z119470_0187 (
PULPROG         deptq135
TD                  65536
SOLVENT             CDCl3
NS                  10240
DS                      8
SWH             29761.904 Hz
FIDRES           0.908261 Hz
AQ              1.1010048 sec
RG                 192.72
DW                 16.800 usec
DE                   6.50 usec
TE                  291.3 K
CNST2         145.0000000
D1             2.00000000 sec
D2             0.00344828 sec
D12            0.00002000 sec
TD0                     1
SFO1          125.7829381 MHz
NUC1                  13C
P1                  10.00 usec
P2                  20.00 usec
PLW1          82.09700012 W
SFO2          500.1820007 MHz
NUC2                   1H
CPDPRG[2          waltz16
P0                  15.00 usec
P3                  10.00 usec
P4                  20.00 usec
PCPD2               80.00 usec
PLW2          18.10400009 W
PLW12          0.28680280 W

F2 - Processing parameters
SI                  32768
SF            125.7703610 MHz
WDW                    EM
SSB                     0
LB                   1.00 Hz
GB                      0
PC                   1.40
```

^{13}C (DEPTQ135) NMR (125 MHz, $CDCl_3$) of ***R*-13**

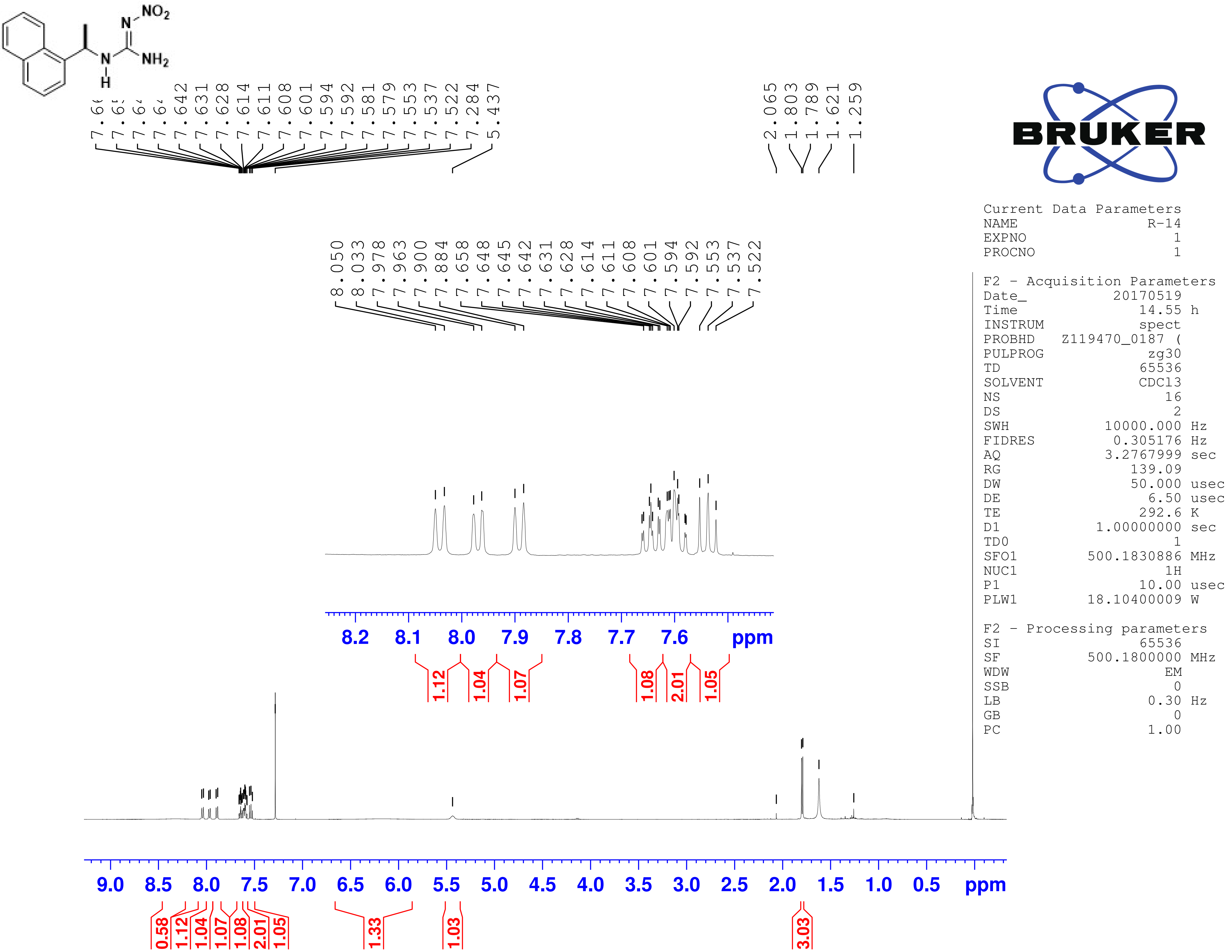

^{1}H NMR (500 MHz, $CDCl_3$) of ***R*-14**

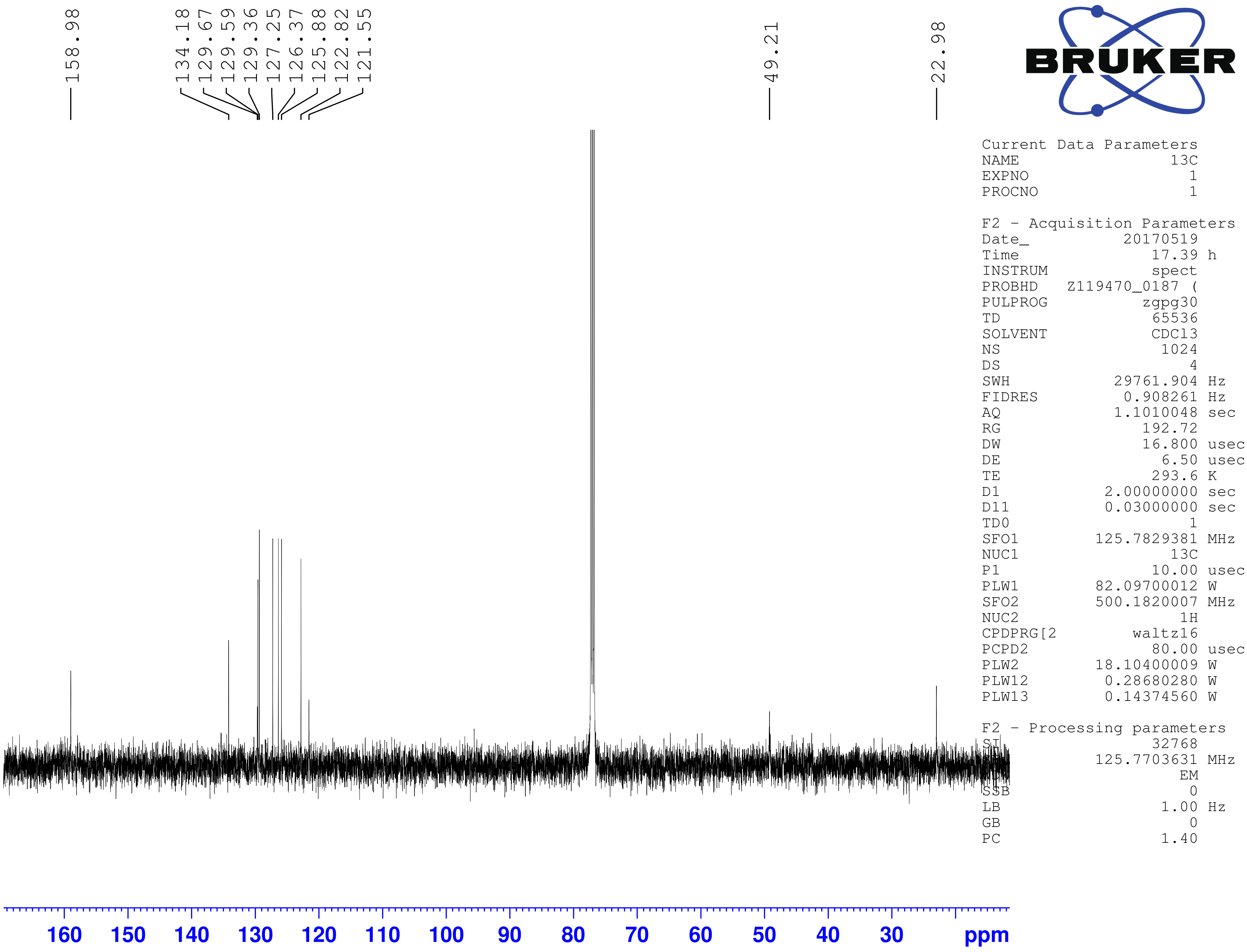

^{13}C NMR (125 MHz, $CDCl_3$) of ***R*-14**

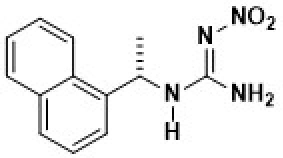

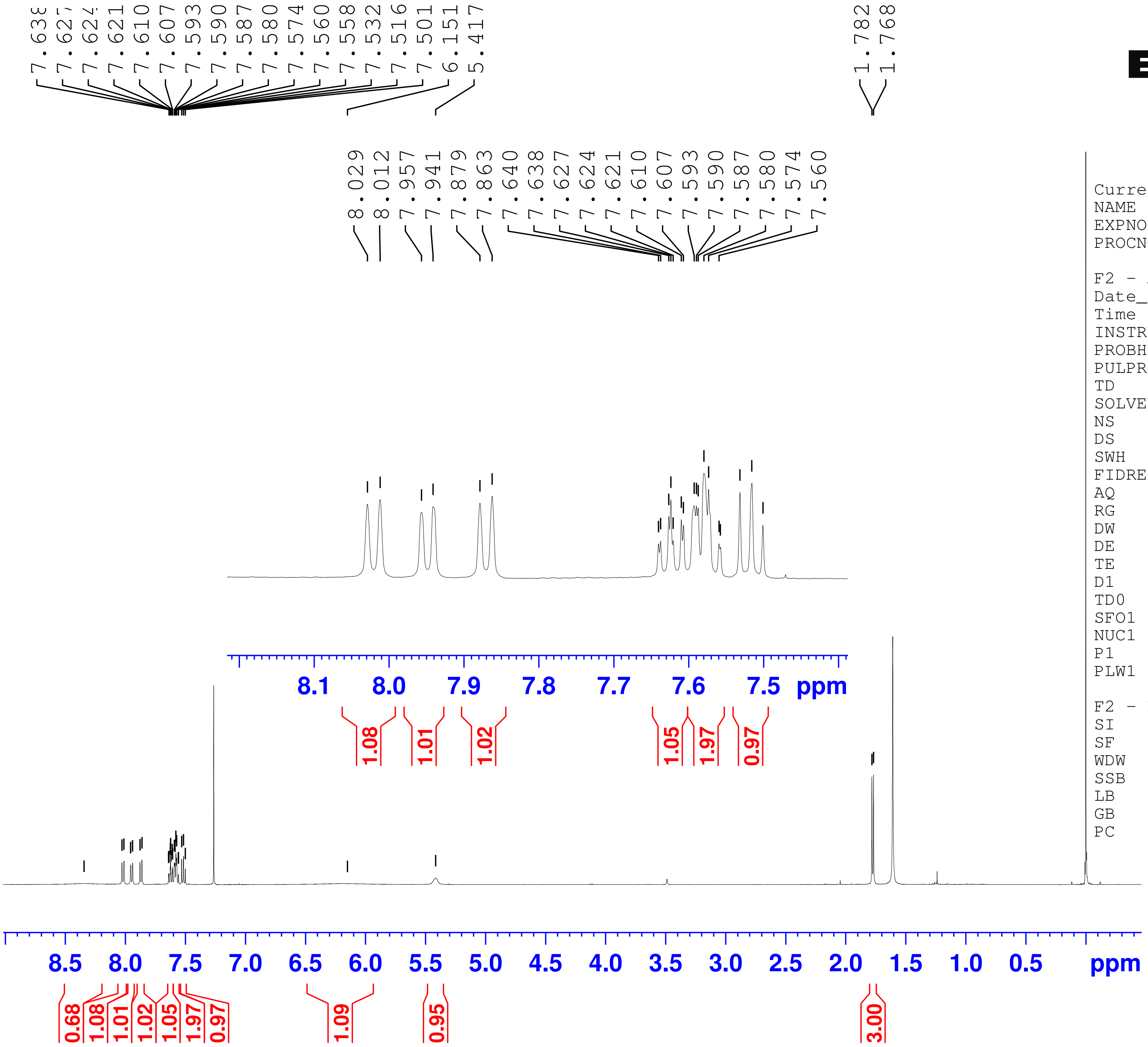

Current Data Parameters
NAME S-15
EXPNO 1
PROCNO 1

F2 - Acquisition Parameters
Date_ 20170531
Time 17.19 h
INSTRUM spect
PROBHD Z119470_0187 (
PULPROG zg30
TD 65536
SOLVENT CDCl3
NS 16
DS 2
SWH 10000.000 Hz
FIDRES 0.305176 Hz
AQ 3.2767999 sec
RG 139.09
DW 50.000 usec
DE 6.50 usec
TE 291.4 K
D1 1.00000000 sec
TD0 1
SFO1 500.1830886 MHz
NUC1 1H
P1 10.00 usec
PLW1 18.10400009 W

F2 - Processing parameters
SI 65536
SF 500.1800101 MHz
WDW EM
SSB 0
LB 0.30 Hz
GB 0
PC 1.00

^{1}H NMR (500 MHz, $CDCl_3$) of ***S*-15**

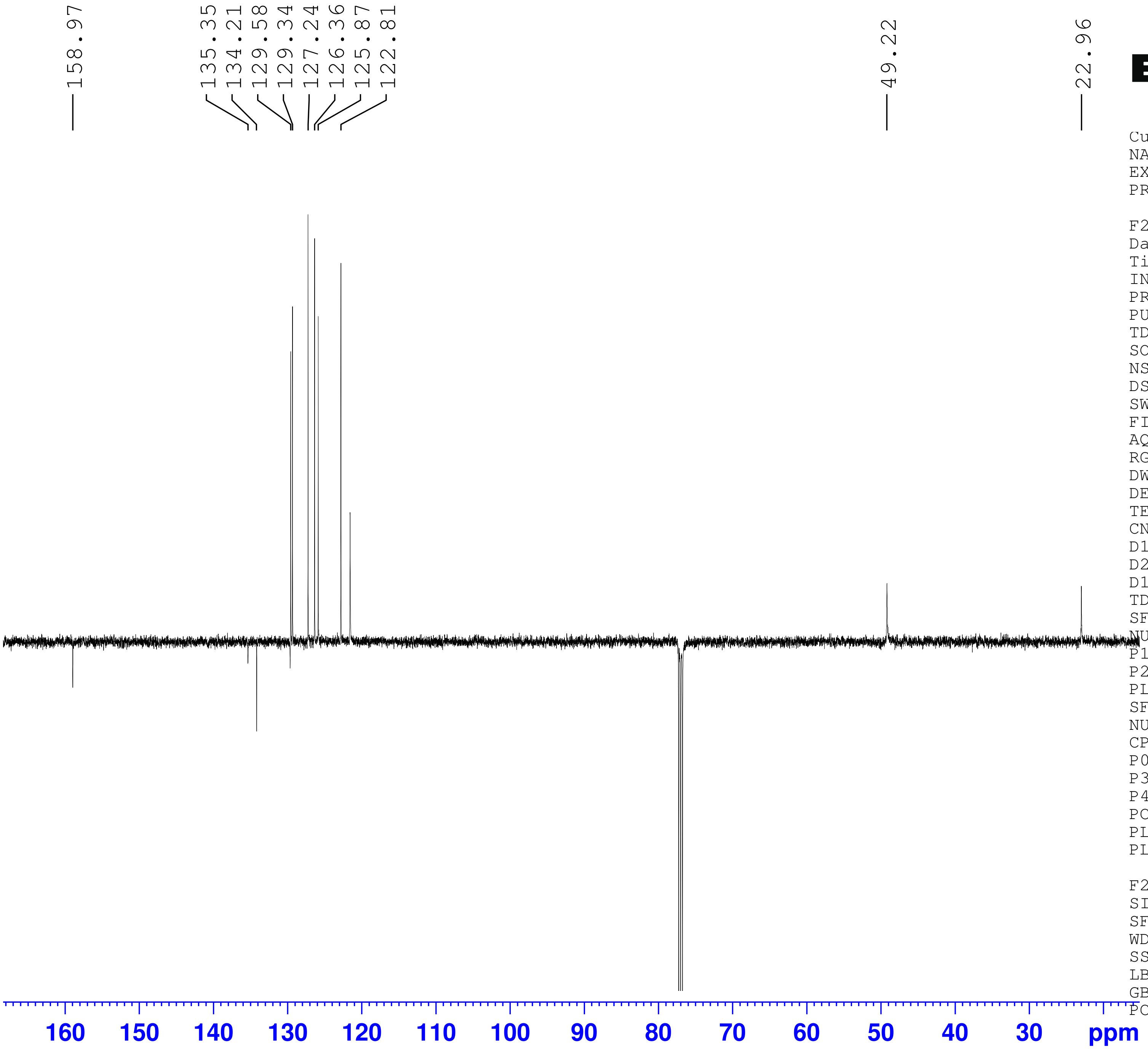

Current Data Parameters
NAME S-15
EXPNO 2
PROCNO 1

F2 - Acquisition Parameters
Date_ 20170601
Time 2.18 h
INSTRUM spect
PROBHD Z119470_0187 (
PULPROG deptq135
TD 65536
SOLVENT CDCl3
NS 10240
DS 8
SWH 29761.904 Hz
FIDRES 0.908261 Hz
AQ 1.1010048 sec
RG 192.72
DW 16.800 usec
DE 6.50 usec
TE 291.5 K
CNST2 145.0000000
D1 2.00000000 sec
D2 0.00344828 sec
D12 0.00002000 sec
TD0 1
SFO1 125.7829381 MHz
NUC1 13C
P1 10.00 usec
P2 20.00 usec
PLW1 82.09700012 W
SFO2 500.1820007 MHz
NUC2 1H
CPDPRG[2 waltz16
P0 15.00 usec
P3 10.00 usec
P4 20.00 usec
PCPD2 80.00 usec
PLW2 18.10400009 W
PLW12 0.28680280 W

F2 - Processing parameters
SI 32768
SF 125.7703639 MHz
WDW EM
SSB 0
LB 1.00 Hz
GB 0
PC 1.40

^{13}C (DEPTQ135) NMR (125 MHz, $CDCl_3$) of ***S*-15**

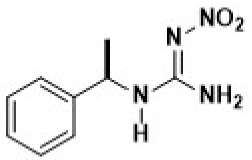

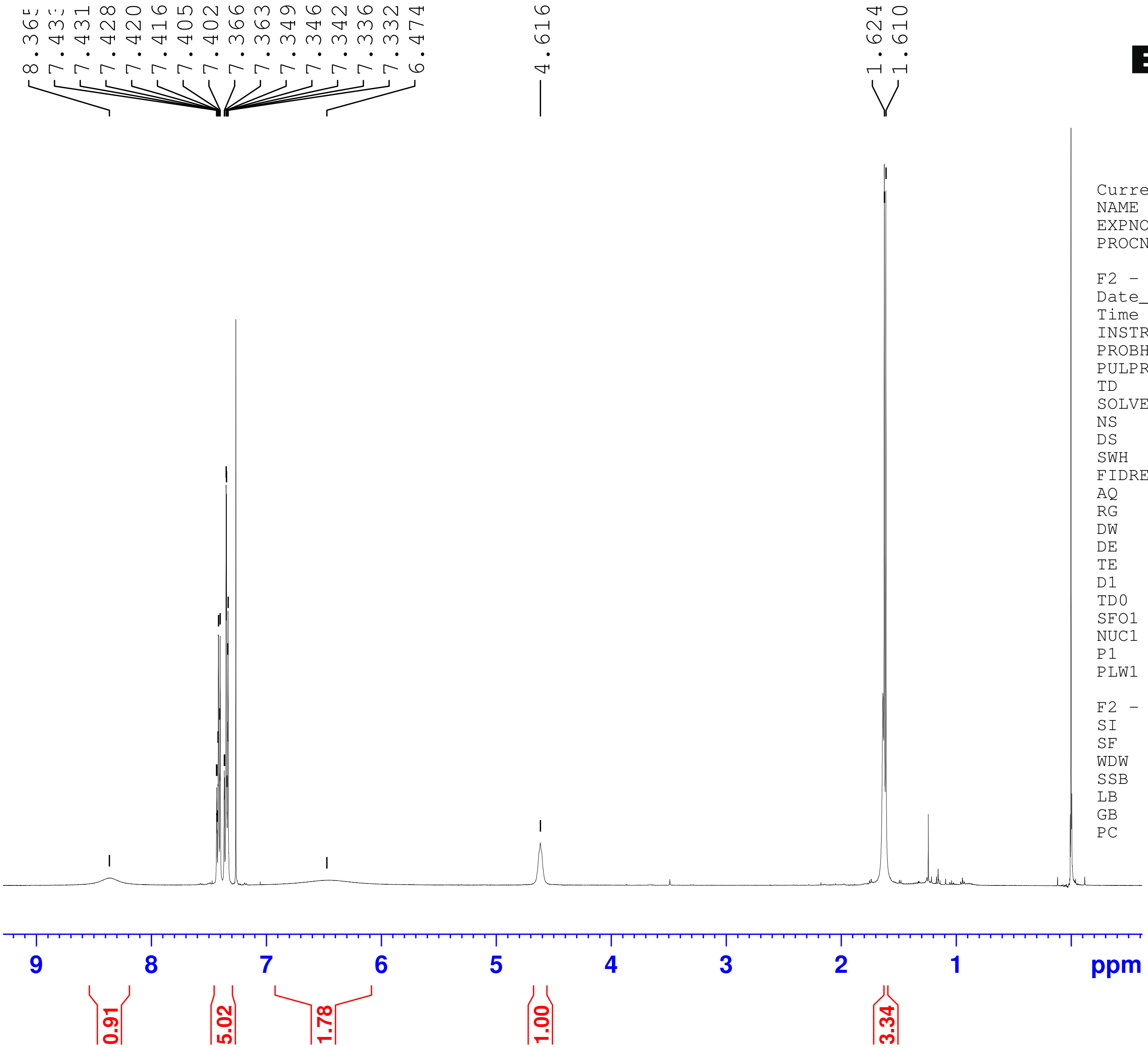


```
Current Data Parameters
NAME                    1
EXPNO                   1
PROCNO                  1

F2 - Acquisition Parameters
Date_            20170519
Time                14.42 h
INSTRUM             spect
PROBHD    Z119470_0187 (
PULPROG              zg30
TD                  65536
SOLVENT             CDCl3
NS                     16
DS                      2
SWH             10000.000 Hz
FIDRES           0.305176 Hz
AQ              3.2767999 sec
RG                 139.09
DW                 50.000 usec
DE                   6.50 usec
TE                  292.6 K
D1             1.00000000 sec
TD0                     1
SFO1          500.1830886 MHz
NUC1                   1H
P1                  10.00 usec
PLW1          18.10400009 W

F2 - Processing parameters
SI                  65536
SF            500.1800096 MHz
WDW                    EM
SSB                     0
LB                   0.30 Hz
GB                      0
PC                   1.00
```


^{1}H NMR (500 MHz, $CDCl_3$) of ***R*-16**

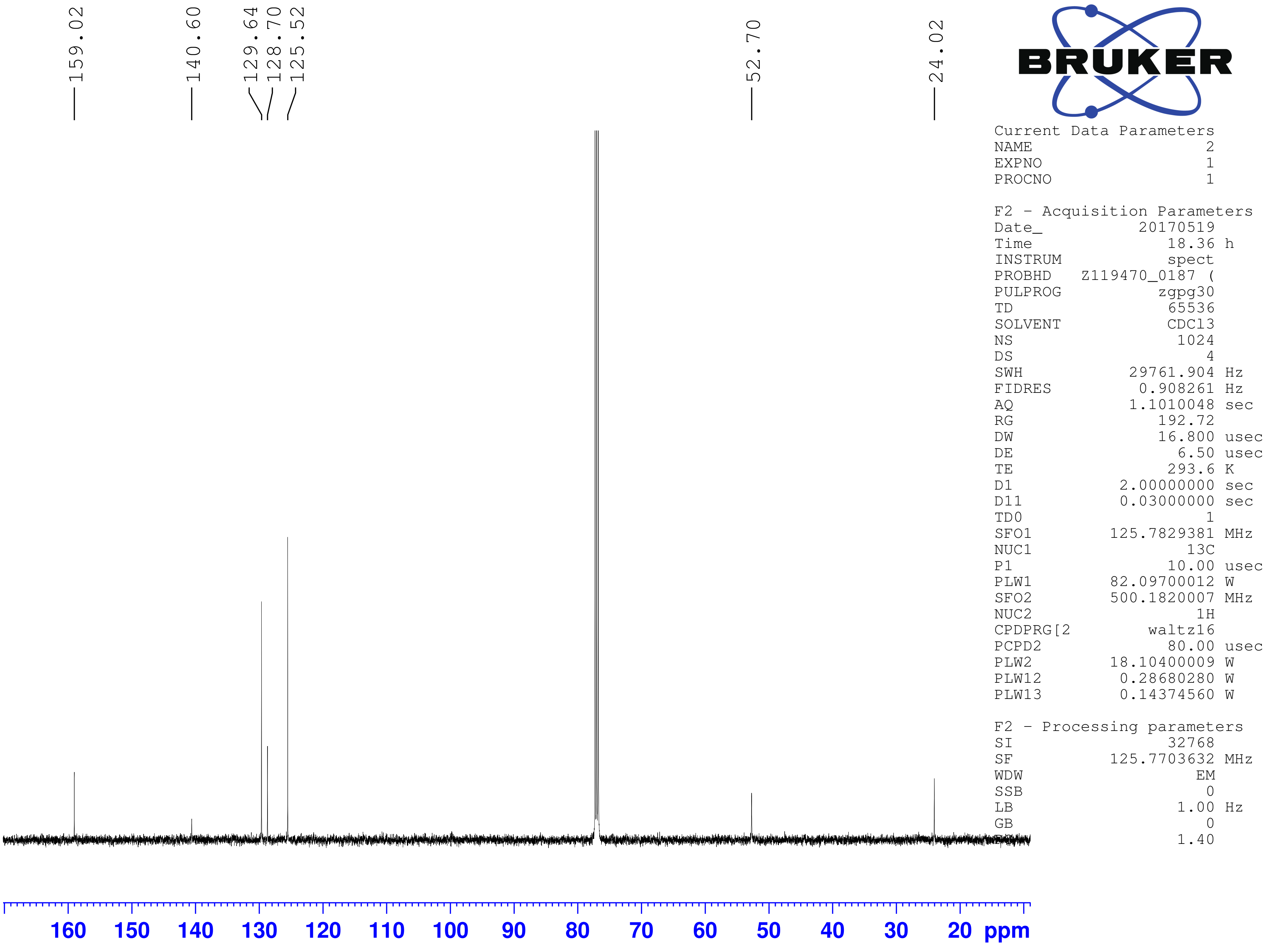

^{13}C NMR (125 MHz, $CDCl_3$) of ***R*-16**

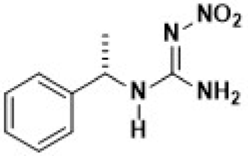

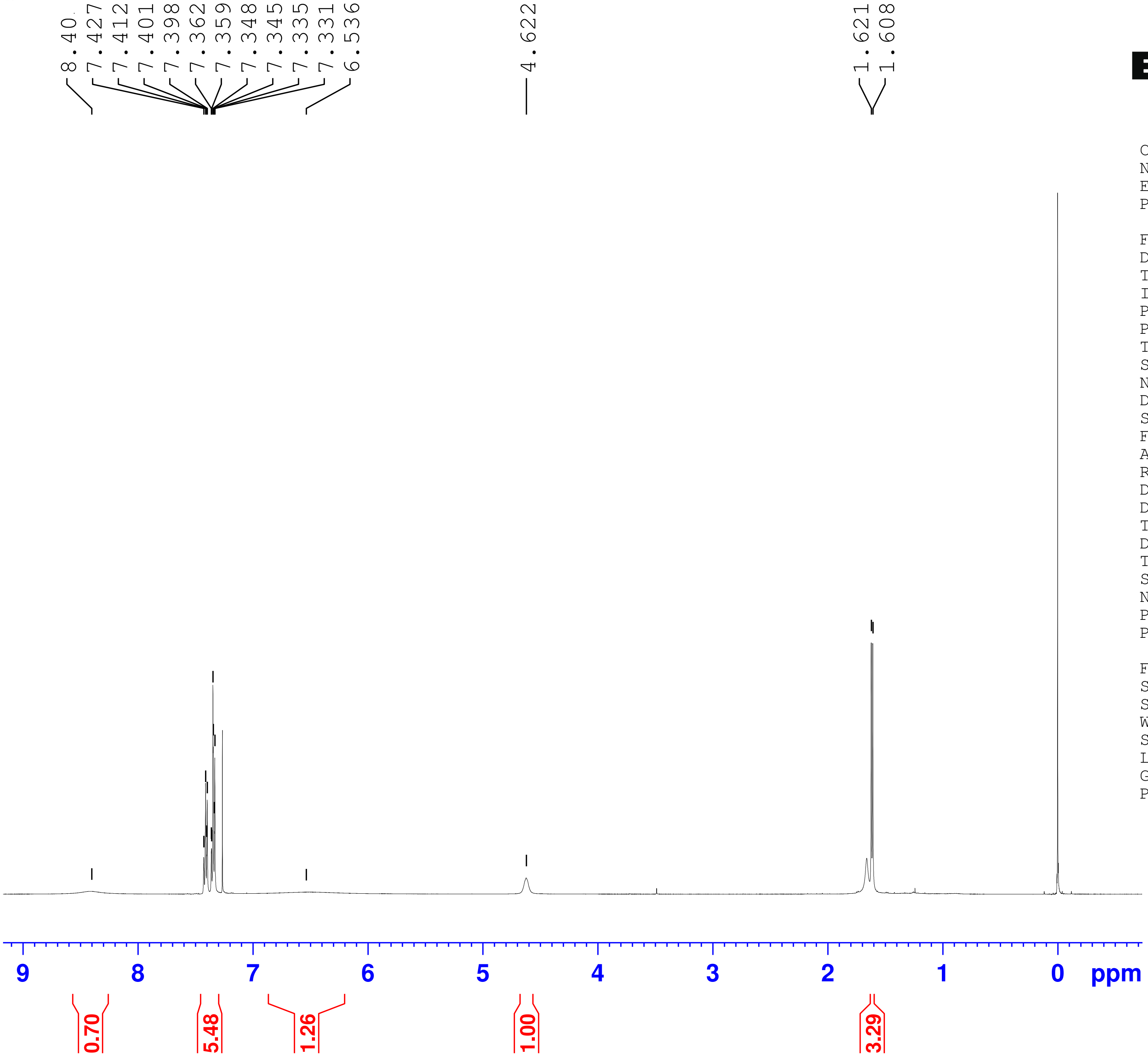

Current Data Parameters
NAME 1H
EXPNO 1
PROCNO 1

F2 - Acquisition Parameters
Date_ 20170519
Time 15.34 h
INSTRUM spect
PROBHD Z119470_0187 (
PULPROG zg30
TD 65536
SOLVENT CDCl3
NS 16
DS 2
SWH 10000.000 Hz
FIDRES 0.305176 Hz
AQ 3.2767999 sec
RG 139.09
DW 50.000 usec
DE 6.50 usec
TE 292.2 K
D1 1.00000000 sec
TD0 1
SFO1 500.1830886 MHz
NUC1 1H
P1 10.00 usec
PLW1 18.10400009 W

F2 - Processing parameters
SI 65536
SF 500.1800094 MHz
WDW EM
SSB 0
LB 0.30 Hz
GB 0
PC 1.00

^{1}H NMR (500 MHz, $CDCl_3$) of ***S*-17**

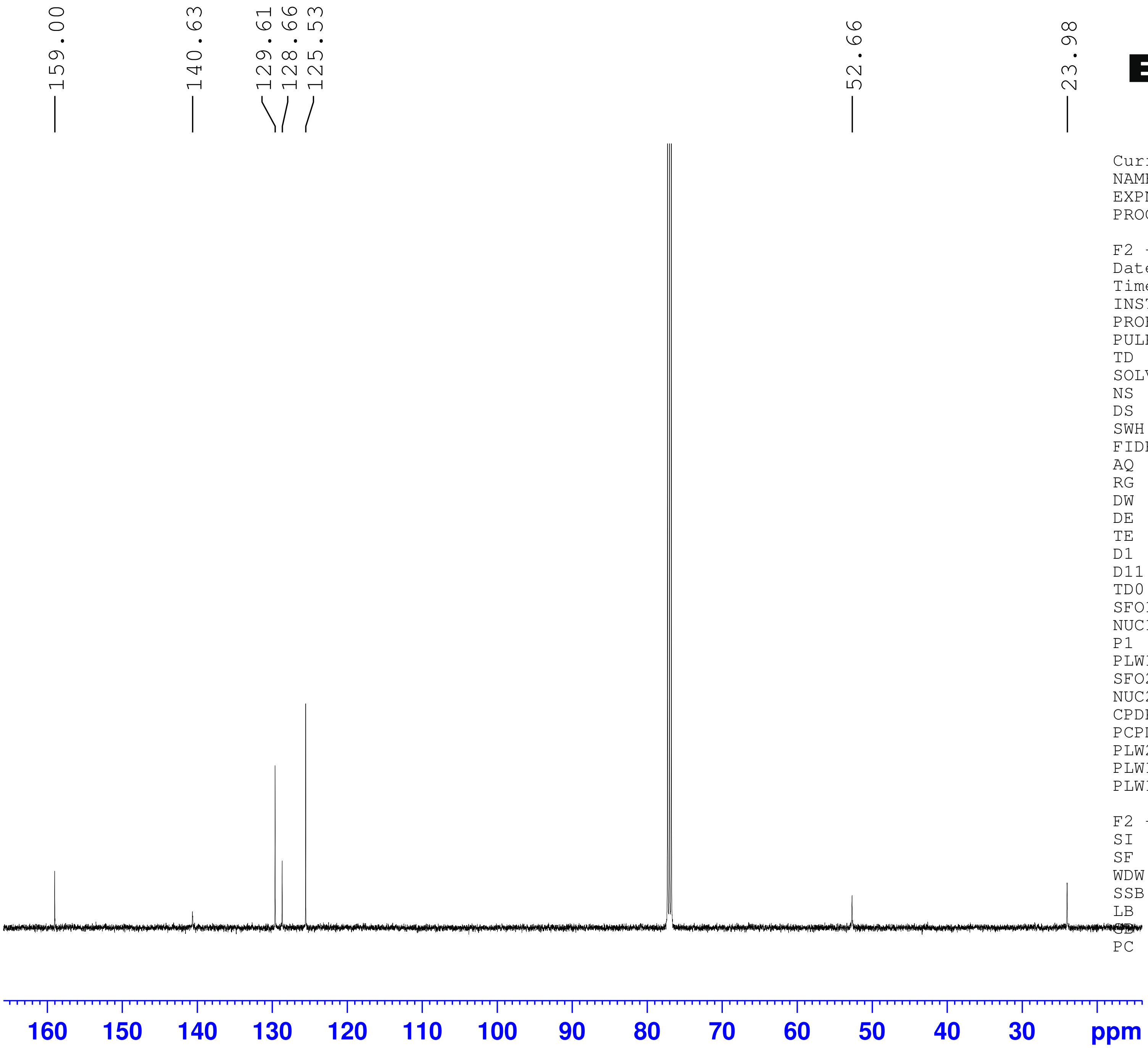

Current Data Parameters

NAME	13C
EXPNO	1
PROCNO	1

F2 - Acquisition Parameters

Date_	20170519
Time	16.41 h
INSTRUM	spect
PROBHD	Z119470_0187 (
PULPROG	zgpg30
TD	65536
SOLVENT	CDCl3
NS	1024
DS	4
SWH	29761.904 Hz
FIDRES	0.908261 Hz
AQ	1.1010048 sec
RG	192.72
DW	16.800 usec
DE	6.50 usec
TE	293.5 K
D1	2.00000000 sec
D11	0.03000000 sec
TD0	1
SFO1	125.7829381 MHz
NUC1	13C
P1	10.00 usec
PLW1	82.09700012 W
SFO2	500.1820007 MHz
NUC2	1H
CPDPRG[2	waltz16
PCPD2	80.00 usec
PLW2	18.10400009 W
PLW12	0.28680280 W
PLW13	0.14374560 W

F2 - Processing parameters

SI	32768
SF	125.7703633 MHz
WDW	EM
SSB	0
LB	1.00 Hz
GB	0
PC	1.40

^{13}C NMR (125 MHz, $CDCl_3$) of ***S*-17**

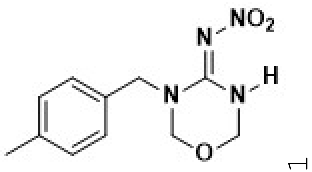

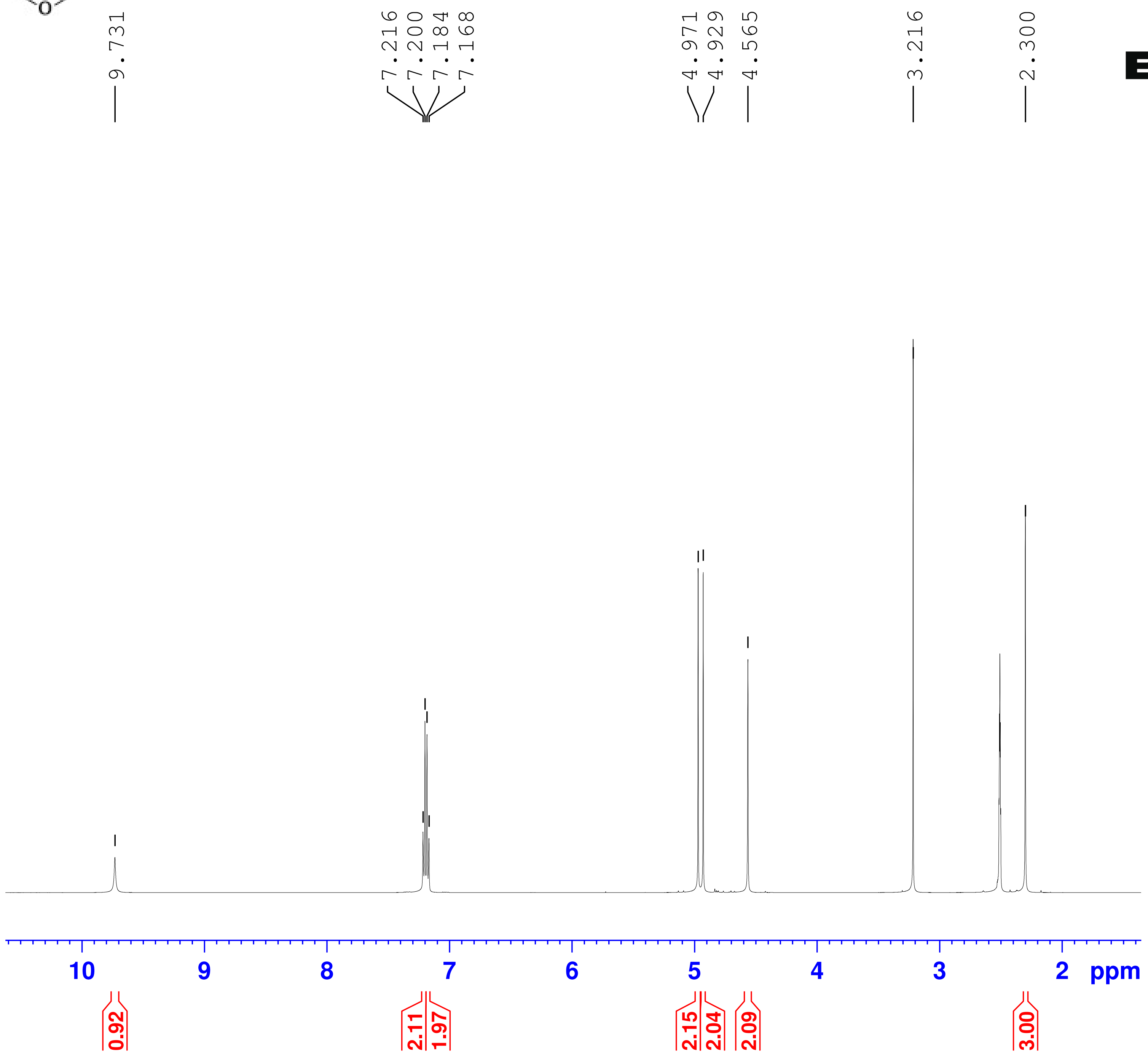

Current Data Parameters
NAME 18
EXPNO 1
PROCNO 1

F2 - Acquisition Parameters
Date_ 20181204
Time 11.33 h
INSTRUM spect
PROBHD Z119470_0187 (
PULPROG zg30
TD 65536
SOLVENT DMSO
NS 16
DS 2
SWH 10000.000 Hz
FIDRES 0.305176 Hz
AQ 3.2767999 sec
RG 108.3
DW 50.000 usec
DE 6.50 usec
TE 323.1 K
D1 1.00000000 sec
TD0 1
SFO1 500.1830886 MHz
NUC1 1H
P0 3.33 usec
P1 10.00 usec
PLW1 18.10400009 W

F2 - Processing parameters
SI 65536
SF 500.1800000 MHz
WDW EM
SSB 0
LB 0.30 Hz
GB 0
PC 1.00

^{1}H NMR (500 MHz, DMSO-d_6, 50°C) of **18**

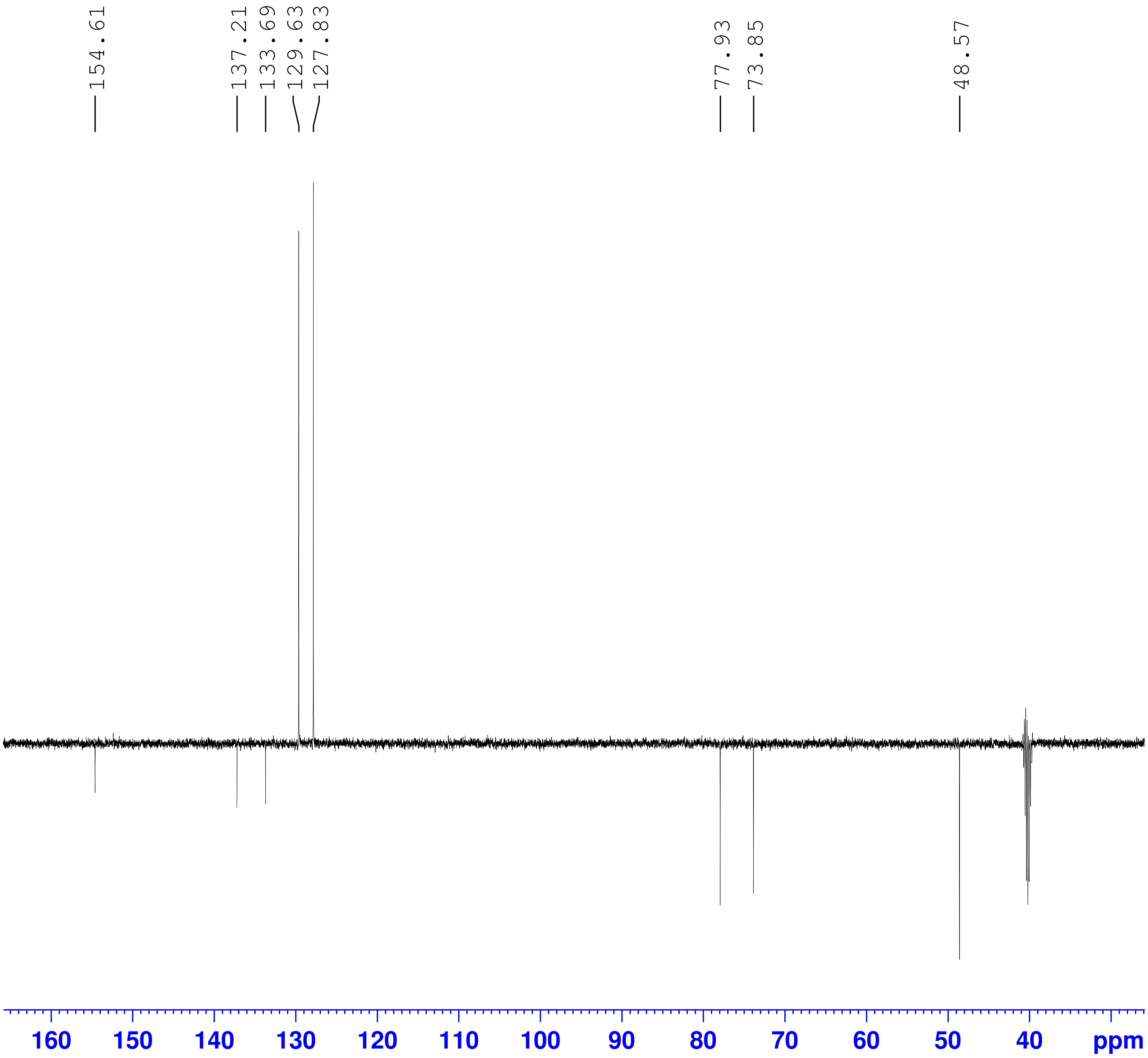

Current Data Parameters
NAME 18
EXPNO 2
PROCNO 1

F2 - Acquisition Parameters

Date_	20181204
Time	11.38 h
INSTRUM	spect
PROBHD	Z119470_0187 (
PULPROG	deptq135
TD	65536
SOLVENT	DMSO
NS	78
DS	8
SWH	29761.904 Hz
FIDRES	0.908261 Hz
AQ	1.1010048 sec
RG	192.72
DW	16.800 usec
DE	6.50 usec
TE	323.2 K
CNST2	145.0000000
D1	2.00000000 sec
D2	0.00344828 sec
D12	0.00002000 sec
TD0	1
SFO1	125.7829381 MHz
NUC1	13C
P1	10.00 usec
P2	20.00 usec
PLW1	82.09700012 W
SFO2	500.1820007 MHz
NUC2	1H
CPDPRG[2	waltz16
P0	15.00 usec
P3	10.00 usec
P4	20.00 usec
PCPD2	80.00 usec
PLW2	18.10400009 W
PLW12	0.28288001 W

F2 - Processing parameters

SI	32768
SF	125.7703610 MHz
WDW	EM
SSB	0
LB	1.00 Hz
GB	0
PC	1.40

^{13}C (DEPTQ135) NMR (125 MHz, DMSO-d_6, 50°C) of **18**

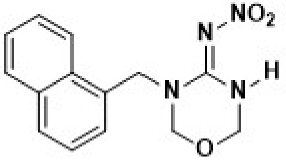

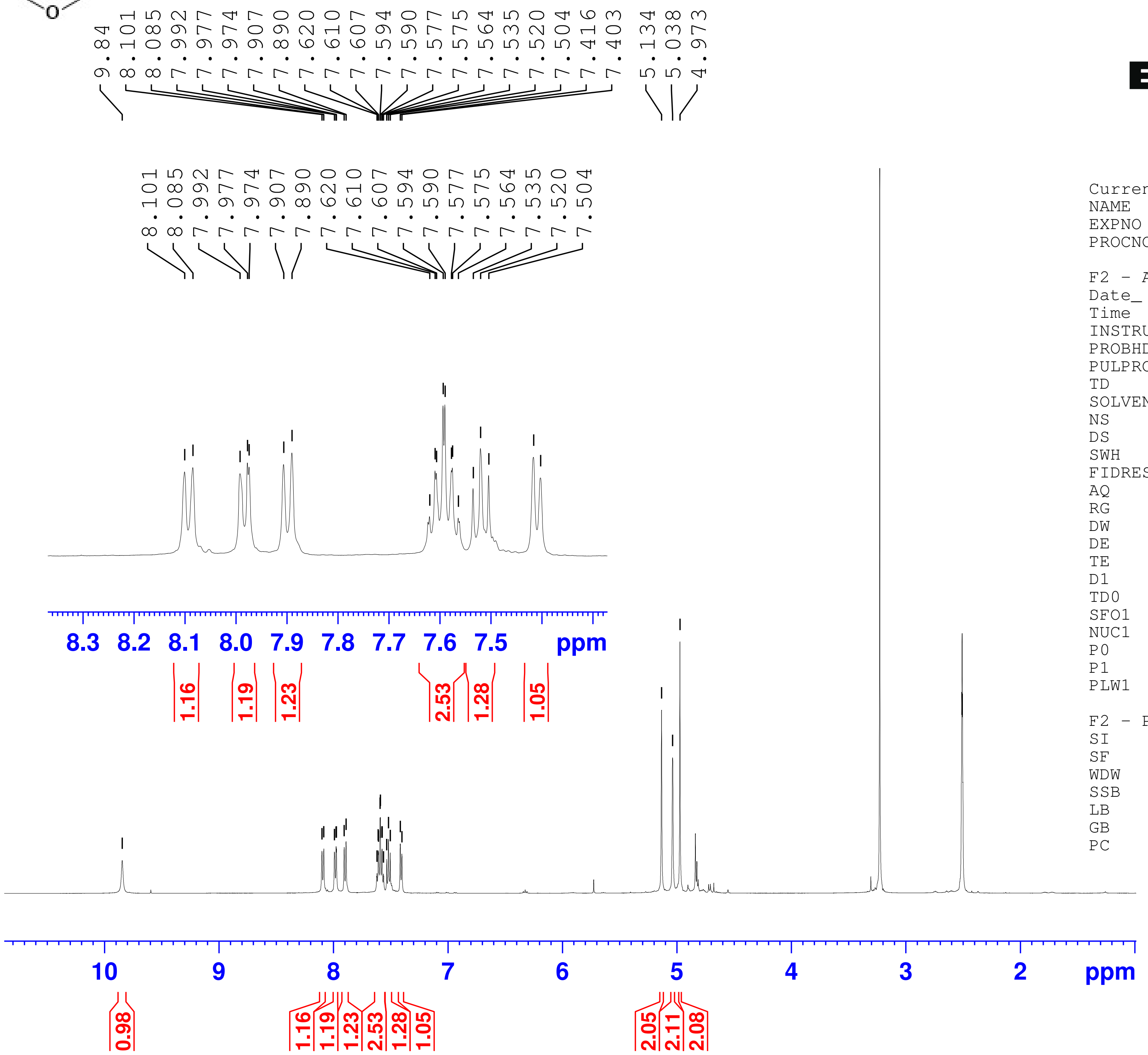

Current Data Parameters
NAME 19
EXPNO 1
PROCNO 1

F2 - Acquisition Parameters
Date_ 20181204
Time 9.03 h
INSTRUM spect
PROBHD Z119470_0187 (
PULPROG zg30
TD 65536
SOLVENT DMSO
NS 16
DS 2
SWH 10000.000 Hz
FIDRES 0.305176 Hz
AQ 3.2767999 sec
RG 95.16
DW 50.000 usec
DE 6.50 usec
TE 323.1 K
D1 1.00000000 sec
TD0 1
SFO1 500.1830886 MHz
NUC1 1H
P0 3.33 usec
P1 10.00 usec
PLW1 18.10400009 W

F2 - Processing parameters
SI 65536
SF 500.1800000 MHz
WDW EM
SSB 0
LB 0.30 Hz
GB 0
PC 1.00

^{1}H NMR (500 MHz, DMSO-d_6, 50°C) of **19**

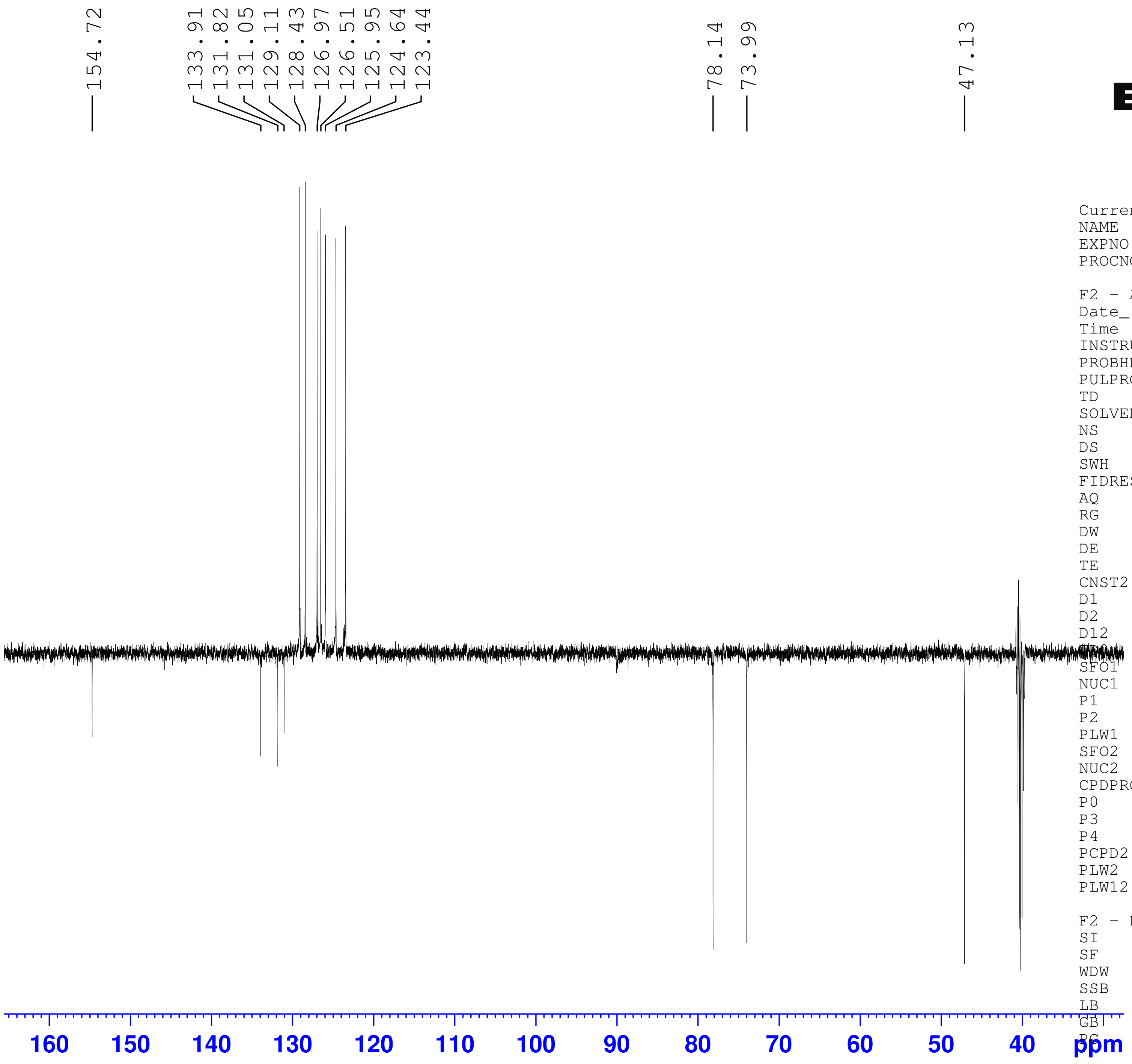

Current Data Parameters
NAME 19
EXPNO 2
PROCNO 1

F2 - Acquisition Parameters
Date_ 20181204
Time 9.13 h
INSTRUM spect
PROBHD Z119470_0187 (
PULPROG deptq135
TD 65536
SOLVENT DMSO
NS 172
DS 8
SWH 29761.904 Hz
FIDRES 0.908261 Hz
AQ 1.1010048 sec
RG 192.72
DW 16.800 usec
DE 6.50 usec
TE 323.2 K
CNST2 145.0000000
D1 2.00000000 sec
D2 0.00344828 sec
D12 0.00002000 sec
TD0 1
SFO1 125.7829381 MHz
NUC1 13C
P1 10.00 usec
P2 20.00 usec
PLW1 82.09700012 W
SFO2 500.1820007 MHz
NUC2 1H
CPDPRG[2 waltz16
P0 15.00 usec
P3 10.00 usec
P4 20.00 usec
PCPD2 80.00 usec
PLW2 18.10400009 W
PLW12 0.28288001 W

F2 - Processing parameters
SI 32768
SF 125.7703610 MHz
WDW EM
SSB 0
LB 1.00 Hz
GB 0
PC 1.40

^{13}C (DEPTQ135) NMR (125 MHz, DMSO-d_6, 50°C) of **19**

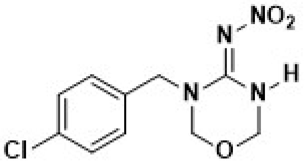

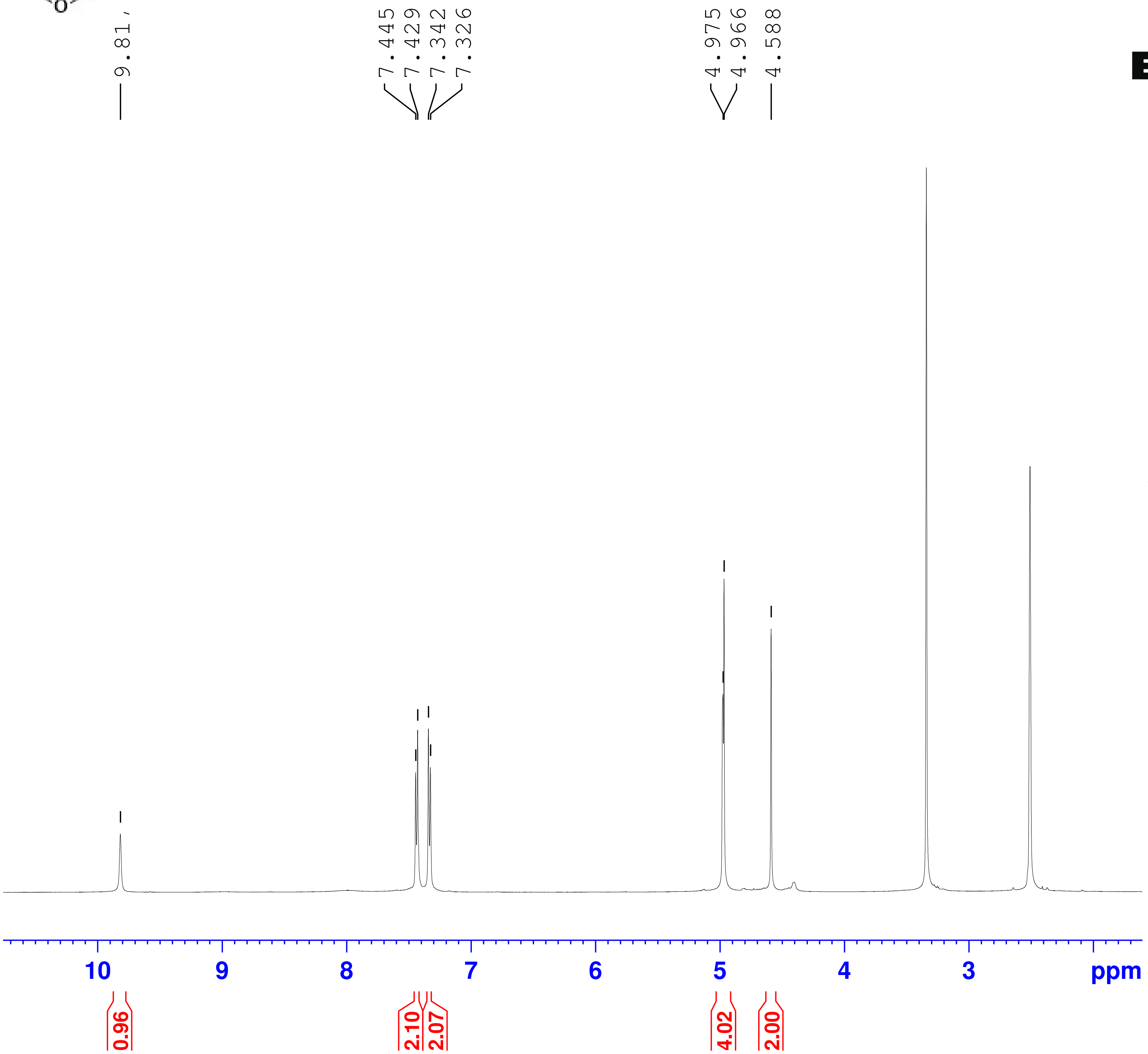

Current Data Parameters
NAME 20
EXPNO 1
PROCNO 1

F2 - Acquisition Parameters
Date_ 20181204
Time 15.14 h
INSTRUM spect
PROBHD Z119470_0187 (
PULPROG zg30
TD 65536
SOLVENT DMSO
NS 16
DS 1
SWH 10000.000 Hz
FIDRES 0.305176 Hz
AQ 3.2767999 sec
RG 139.09
DW 50.000 usec
DE 6.50 usec
TE 298.0 K
D1 1.00000000 sec
TD0 1
SFO1 500.1830886 MHz
NUC1 1H
P0 3.33 usec
P1 10.00 usec
PLW1 18.10400009 W

F2 - Processing parameters
SI 65536
SF 500.1800000 MHz
WDW EM
SSB 0
LB 0.30 Hz
GB 0
PC 1.00

^{1}H NMR (500 MHz, DMSO-d_6) of **20**

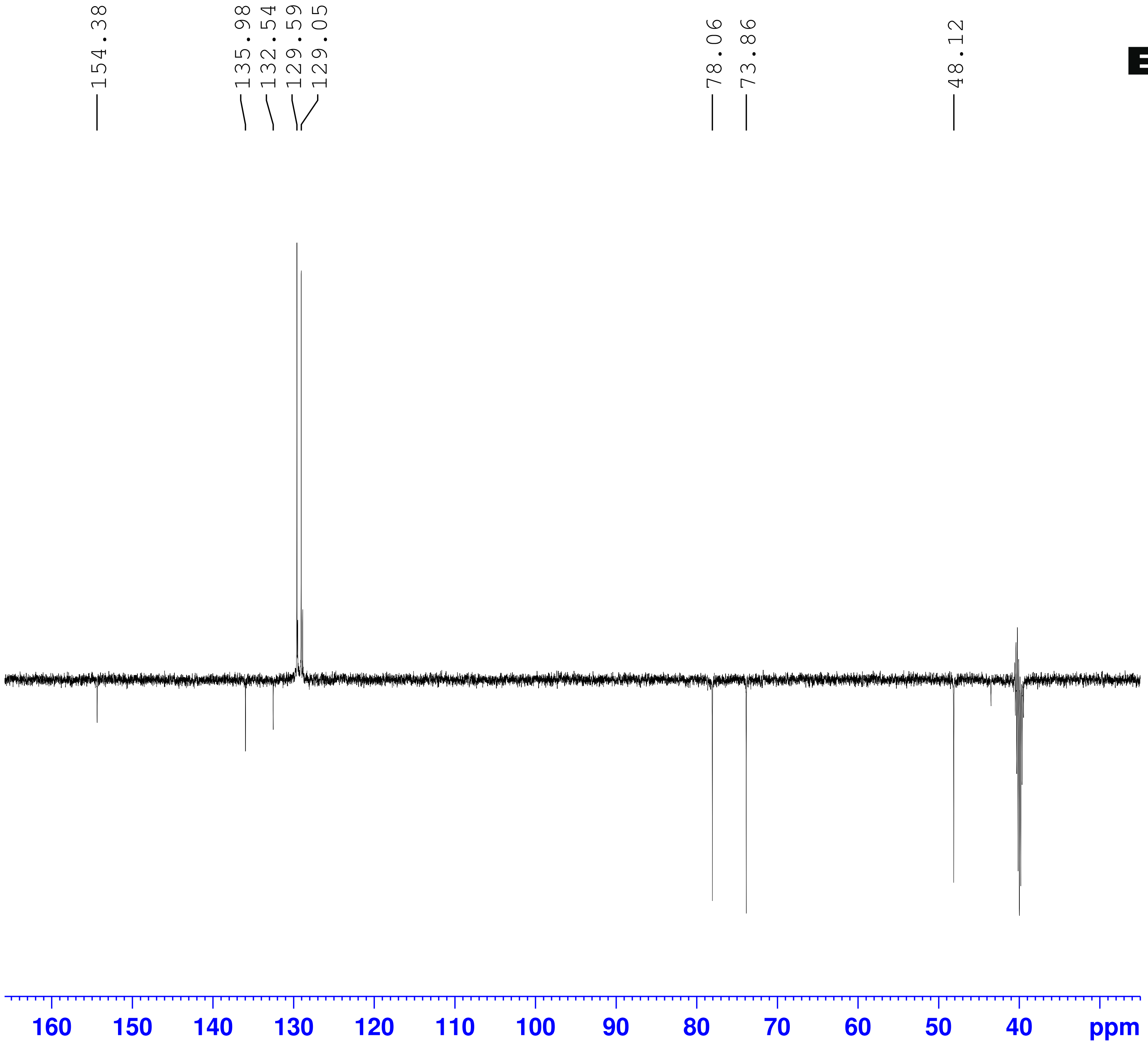

Current Data Parameters
NAME 20
EXPNO 2
PROCNO 1

F2 - Acquisition Parameters
Date_ 20181204
Time 15.21 h
INSTRUM spect
PROBHD Z119470_0187 (
PULPROG deptq135
TD 65536
SOLVENT DMSO
NS 122
DS 8
SWH 29761.904 Hz
FIDRES 0.908261 Hz
AQ 1.1010048 sec
RG 192.72
DW 16.800 usec
DE 6.50 usec
TE 296.0 K
CNST2 145.0000000
D1 2.00000000 sec
D2 0.00344828 sec
D12 0.00002000 sec
TD0 1
SFO1 125.7829381 MHz
NUC1 13C
P1 10.00 usec
P2 20.00 usec
PLW1 82.09700012 W
SFO2 500.1820007 MHz
NUC2 1H
CPDPRG[2 waltz16
P0 15.00 usec
P3 10.00 usec
P4 20.00 usec
PCPD2 80.00 usec
PLW2 18.10400009 W
PLW12 0.28288001 W

F2 - Processing parameters
SI 32768
SF 125.7703610 MHz
WDW EM
SSB 0
LB 1.00 Hz
GB 0
PC 1.40

^{13}C (DEPTQ135) NMR (125 MHz, DMSO-d_6) of **20**

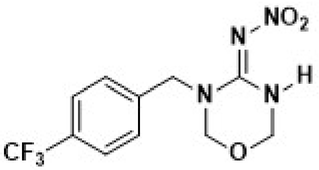

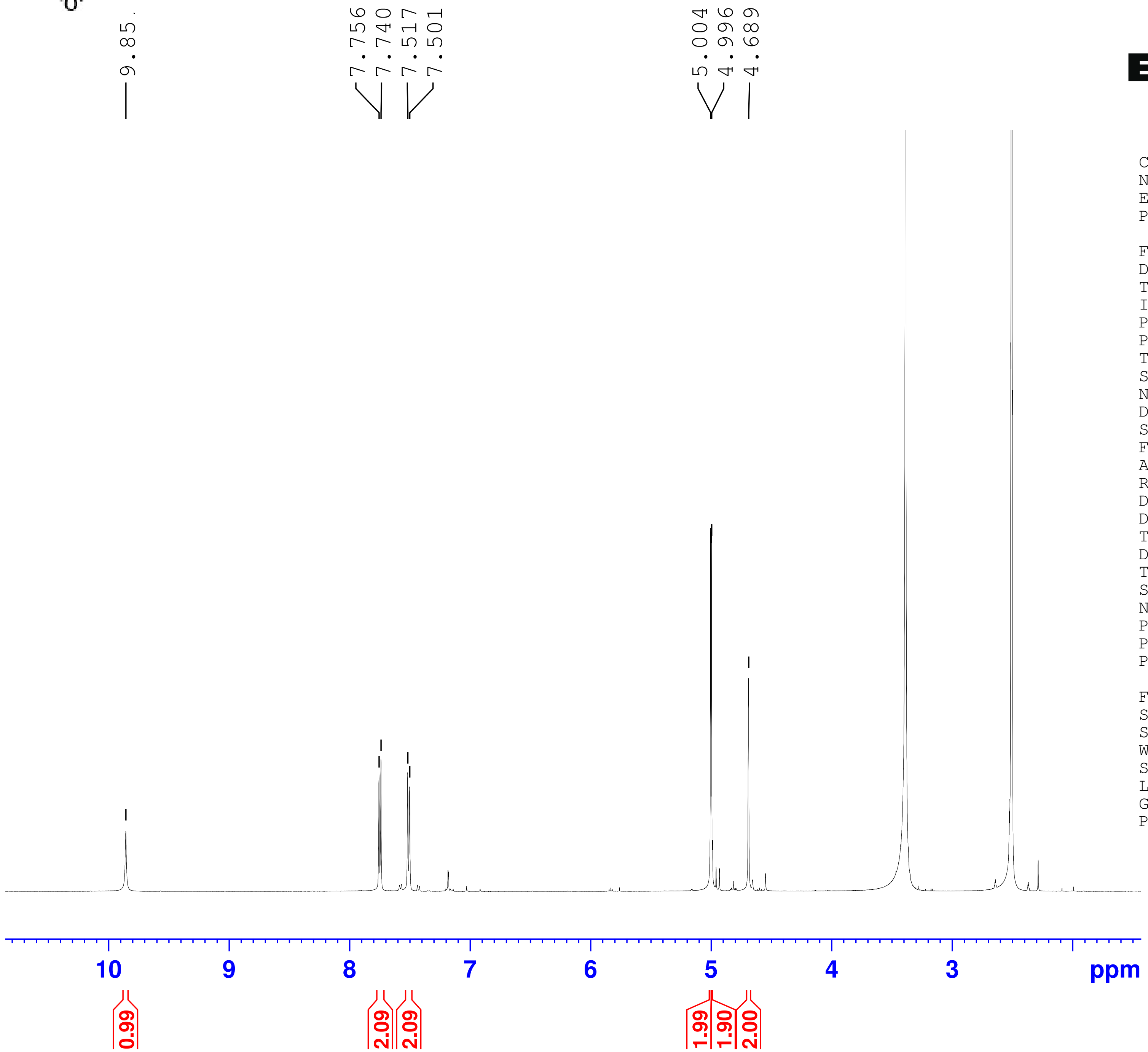

Current Data Parameters
NAME 21
EXPNO 3
PROCNO 1

F2 - Acquisition Parameters
Date_ 20180907
Time 12.51 h
INSTRUM spect
PROBHD Z119470_0187 (
PULPROG zg30
TD 65536
SOLVENT DMSO
NS 16
DS 2
SWH 10000.000 Hz
FIDRES 0.305176 Hz
AQ 3.2767999 sec
RG 88.37
DW 50.000 usec
DE 6.50 usec
TE 291.9 K
D1 1.00000000 sec
TD0 1
SFO1 500.1830886 MHz
NUC1 1H
P0 3.33 usec
P1 10.00 usec
PLW1 18.10400009 W

F2 - Processing parameters
SI 65536
SF 500.1800000 MHz
WDW EM
SSB 0
LB 0.30 Hz
GB 0
PC 1.00

^{1}H NMR (500 MHz, DMSO-d_6) of **21**

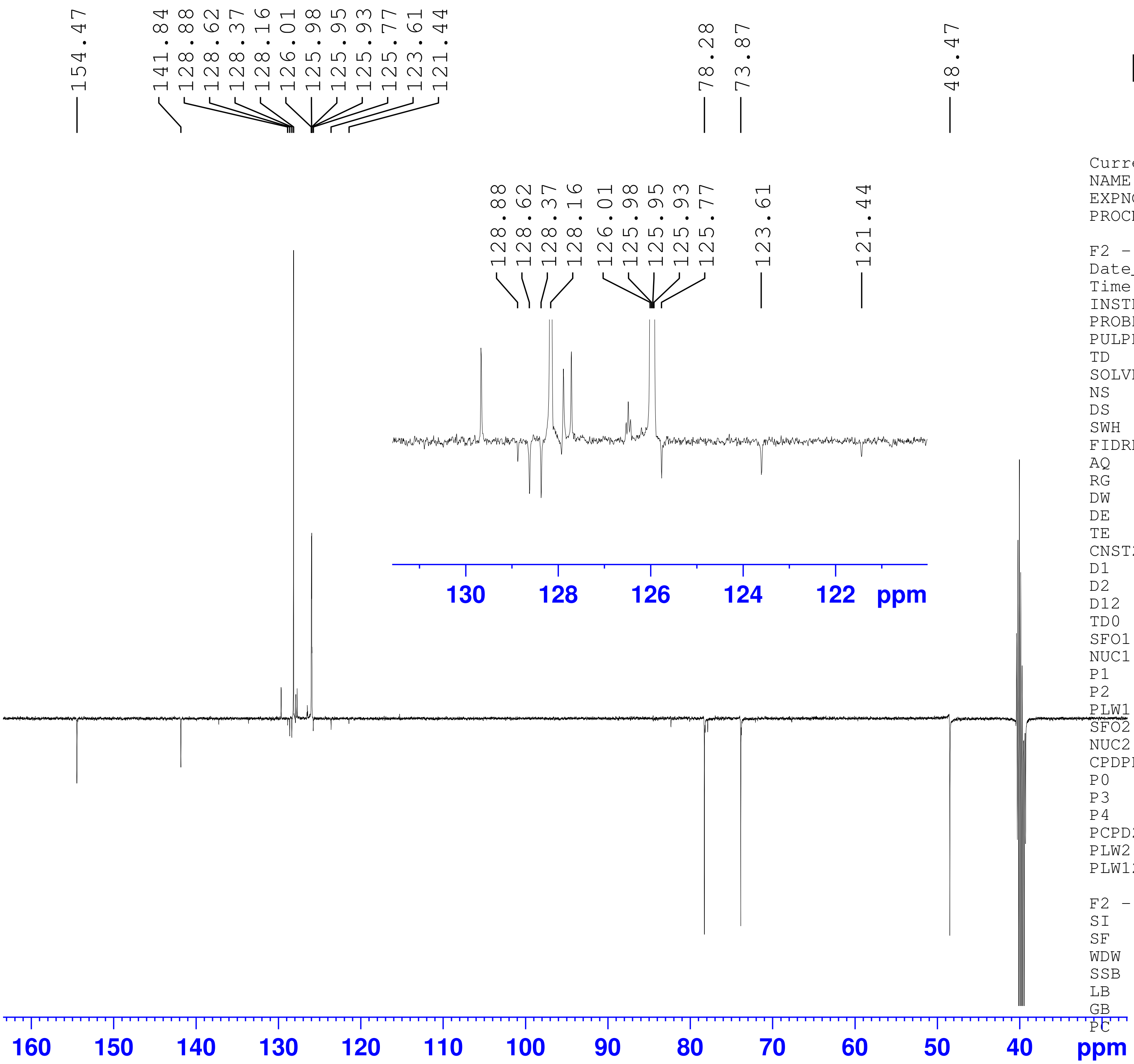

Current Data Parameters
NAME 21
EXPNO 4
PROCNO 1

F2 - Acquisition Parameters
Date_ 20180926
Time 10.05 h
INSTRUM spect
PROBHD Z119470_0187 (
PULPROG deptq135
TD 65536
SOLVENT DMSO
NS 17064
DS 8
SWH 29761.904 Hz
FIDRES 0.908261 Hz
AQ 1.1010048 sec
RG 192.72
DW 16.800 usec
DE 6.50 usec
TE 295.4 K
CNST2 145.0000000
D1 2.00000000 sec
D2 0.00344828 sec
D12 0.00002000 sec
TD0 1
SFO1 125.7829381 MHz
NUC1 13C
P1 10.00 usec
P2 20.00 usec
PLW1 82.09700012 W
SFO2 500.1820007 MHz
NUC2 1H
CPDPRG[2 waltz16
P0 15.00 usec
P3 10.00 usec
P4 20.00 usec
PCPD2 80.00 usec
PLW2 18.10400009 W
PLW12 0.28288001 W

F2 - Processing parameters
SI 32768
SF 125.7703610 MHz
WDW EM
SSB 0
LB 1.00 Hz
GB 0
PC 1.40

^{13}C (DEPTQ135) NMR (125 MHz, DMSO-d_6) of **21**

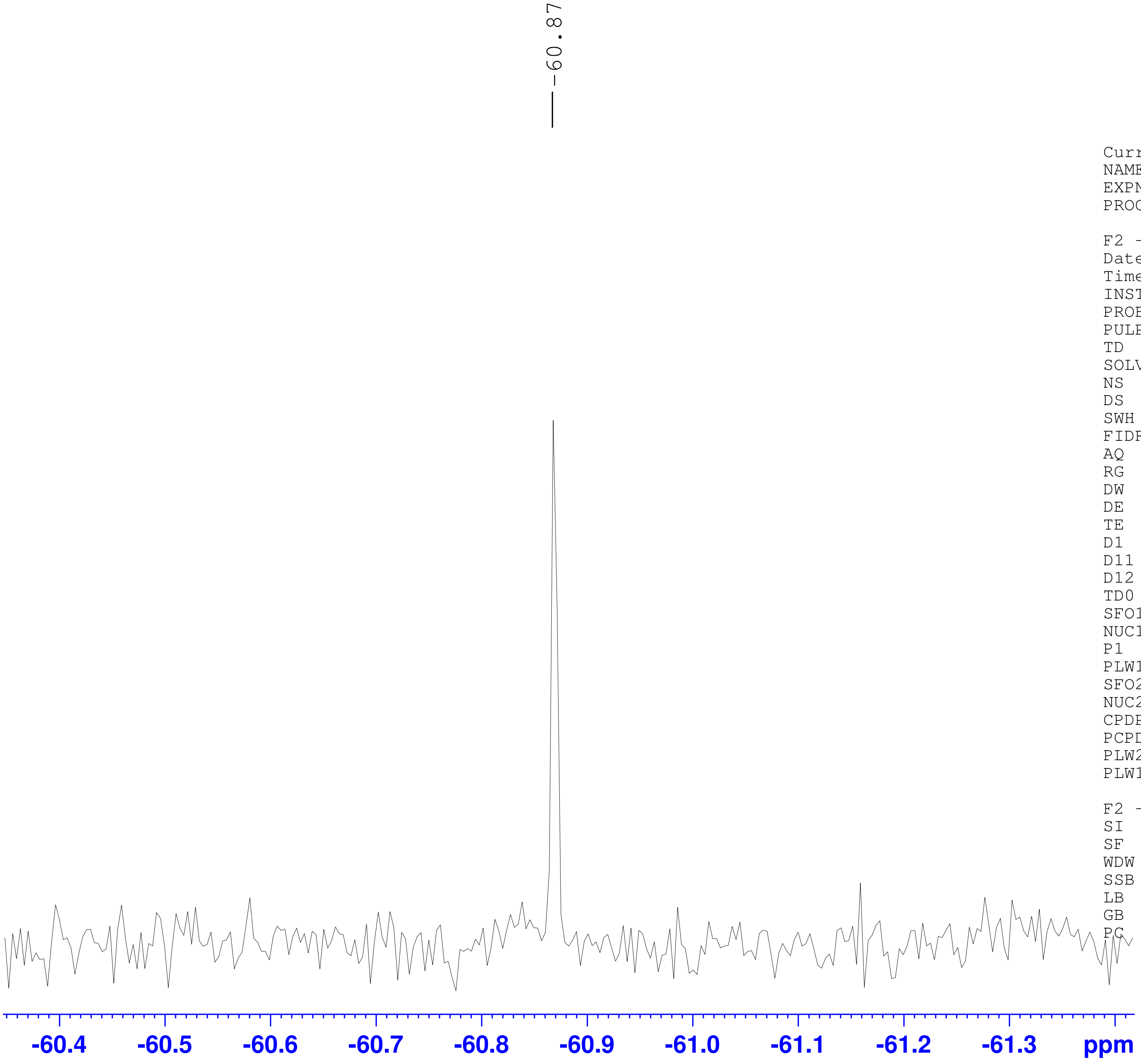

Current Data Parameters

NAME	ARH-8
EXPNO	1
PROCNO	1

F2 - Acquisition Parameters

Date_	20190801
Time	14.44 h
INSTRUM	spect
PROBHD	Z119470_0187 (
PULPROG	zgfhigqn.2
TD	131072
SOLVENT	DMSO
NS	16
DS	4
SWH	113636.367 Hz
FIDRES	1.733953 Hz
AQ	0.5767168 sec
RG	11.27
DW	4.400 usec
DE	6.50 usec
TE	293.4 K
D1	1.00000000 sec
D11	0.03000000 sec
D12	0.00002000 sec
TD0	1
SFO1	470.5923603 MHz
NUC1	19F
P1	16.00 usec
PLW1	36.93899918 W
SFO2	500.1820007 MHz
NUC2	1H
CPDPRG[2	waltz16
PCPD2	80.00 usec
PLW2	18.10400009 W
PLW12	0.31009001 W

F2 - Processing parameters

SI	65536
SF	470.6394242 MHz
WDW	EM
SSB	0
LB	0.30 Hz
GB	0
PC	1.00

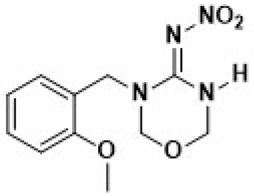

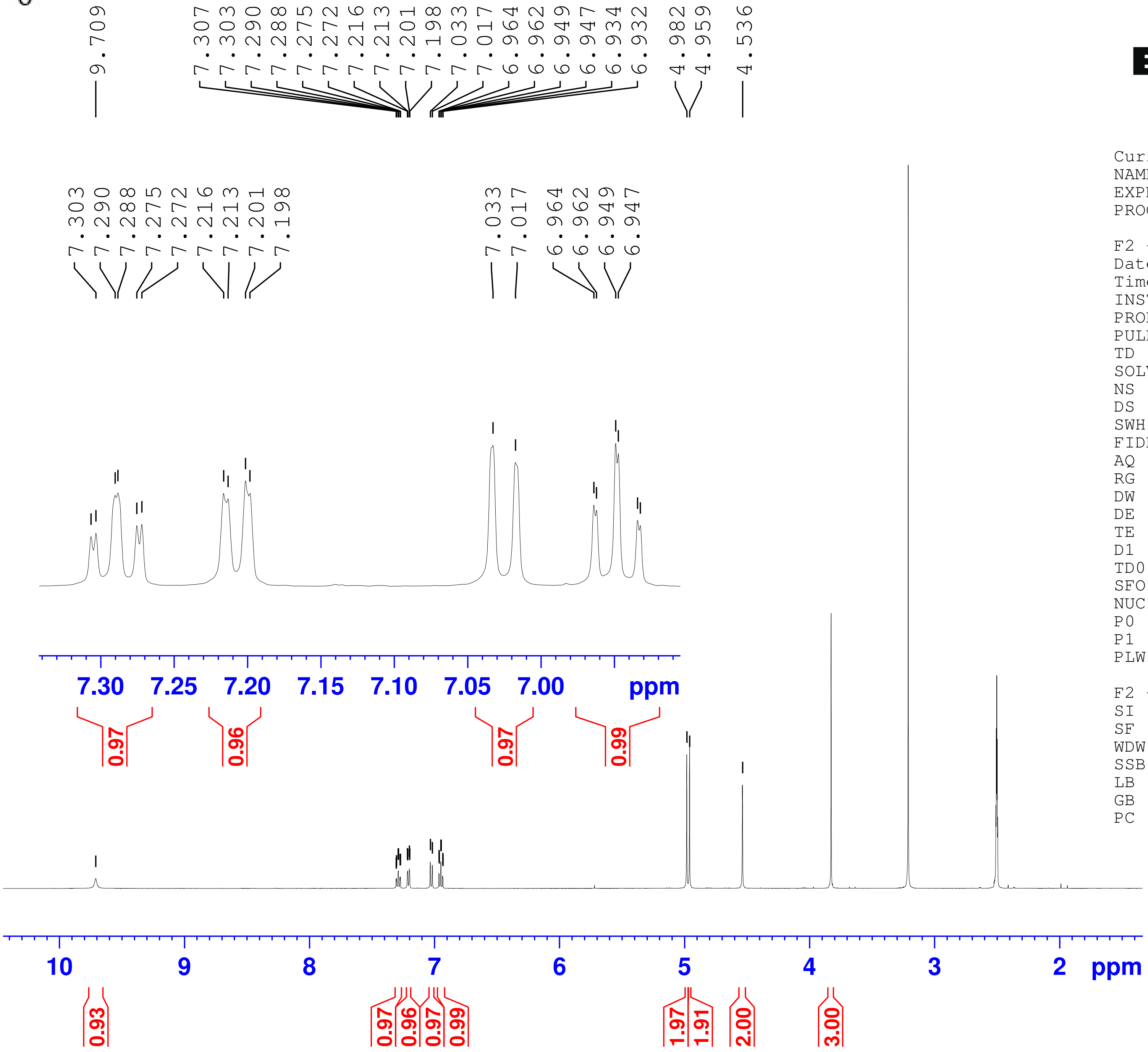

Current Data Parameters
NAME 22
EXPNO 1
PROCNO 1

F2 - Acquisition Parameters
Date_ 20181204
Time 12.30 h
INSTRUM spect
PROBHD Z119470_0187 (
PULPROG zg30
TD 65536
SOLVENT DMSO
NS 16
DS 2
SWH 10000.000 Hz
FIDRES 0.305176 Hz
AQ 3.2767999 sec
RG 139.09
DW 50.000 usec
DE 6.50 usec
TE 323.0 K
D1 1.00000000 sec
TD0 1
SFO1 500.1830886 MHz
NUC1 1H
P0 3.33 usec
P1 10.00 usec
PLW1 18.10400009 W

F2 - Processing parameters
SI 65536
SF 500.1800000 MHz
WDW EM
SSB 0
LB 0.30 Hz
GB 0
PC 1.00

^{1}H NMR (500 MHz, DMSO-d_6, 50°C) of **22**

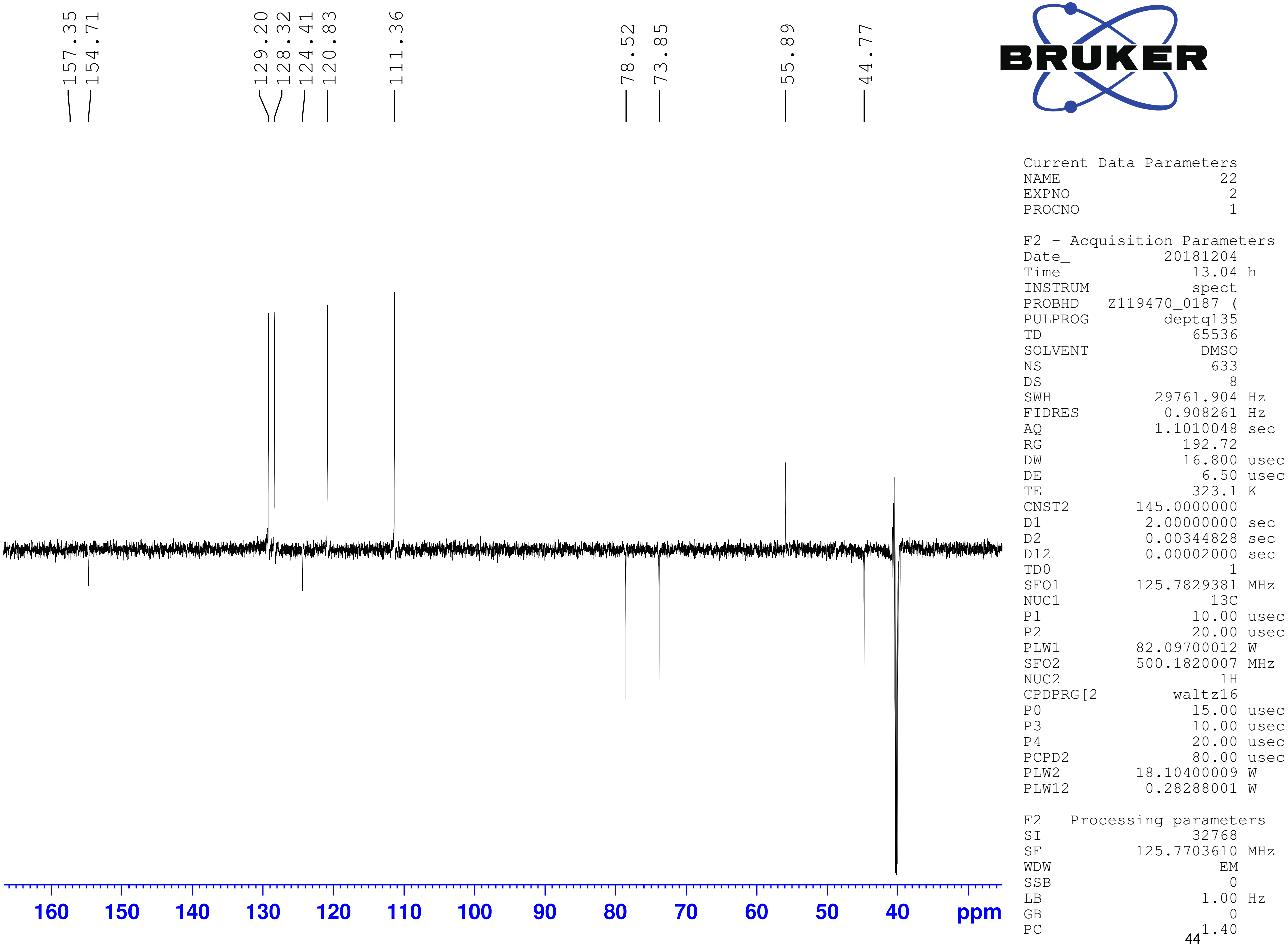

^{13}C (DEPTQ135) NMR (125 MHz, DMSO-d_6, 50°C) of **22**

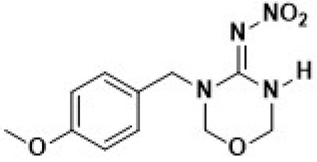

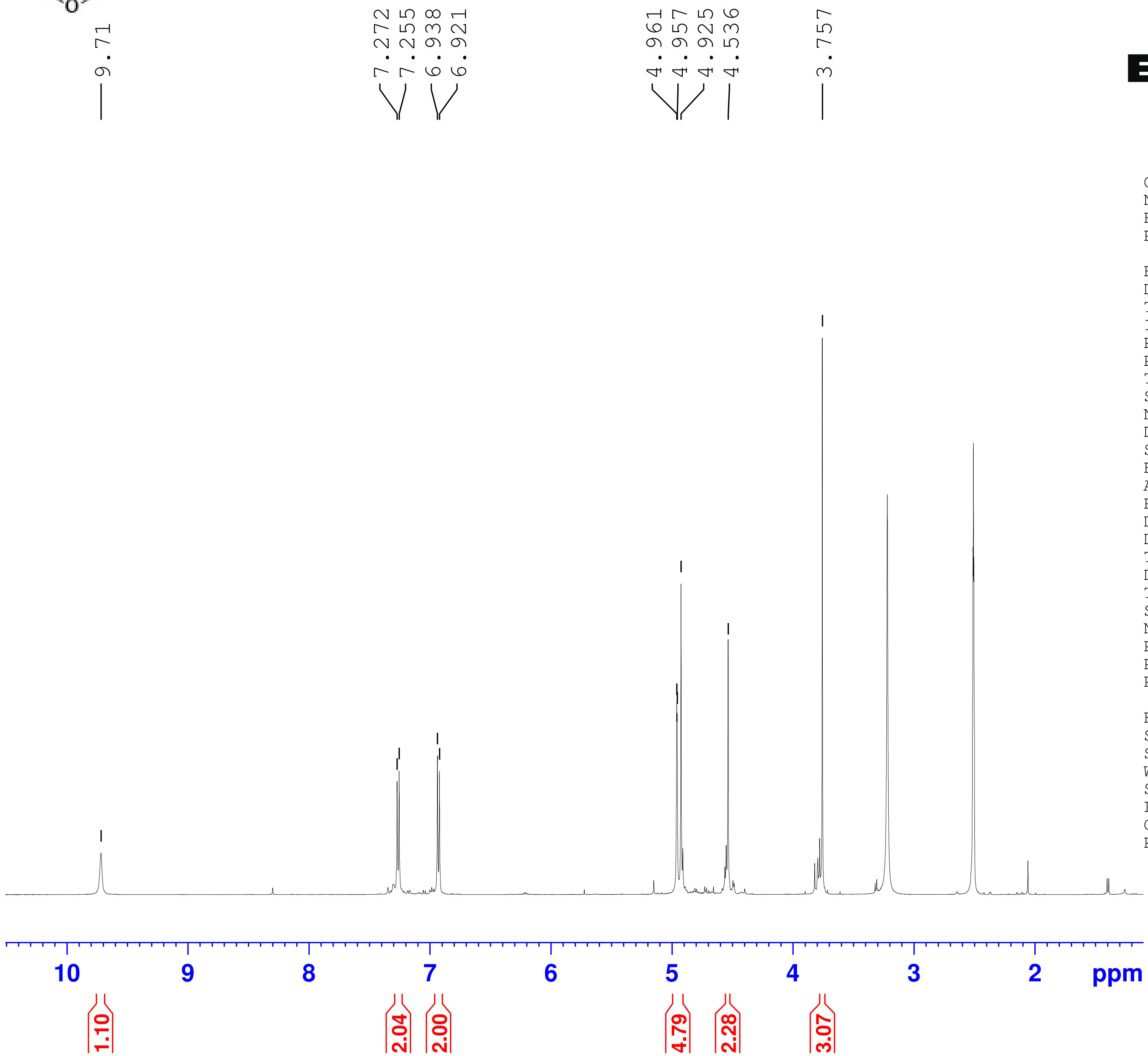

Current Data Parameters
NAME 23
EXPNO 1
PROCNO 1

F2 - Acquisition Parameters
Date_ 20181204
Time 11.43 h
INSTRUM spect
PROBHD Z119470_0187 (
PULPROG zg30
TD 65536
SOLVENT DMSO
NS 16
DS 2
SWH 10000.000 Hz
FIDRES 0.305176 Hz
AQ 3.2767999 sec
RG 139.09
DW 50.000 usec
DE 6.50 usec
TE 323.1 K
D1 1.00000000 sec
TD0 1
SFO1 500.1830886 MHz
NUC1 1H
P0 3.33 usec
P1 10.00 usec
PLW1 18.10400009 W

F2 - Processing parameters
SI 65536
SF 500.1800000 MHz
WDW EM
SSB 0
LB 0.30 Hz
GB 0
PC 1.00

^{1}H NMR (500 MHz, DMSO-d$_6$, 50°C) of **23**

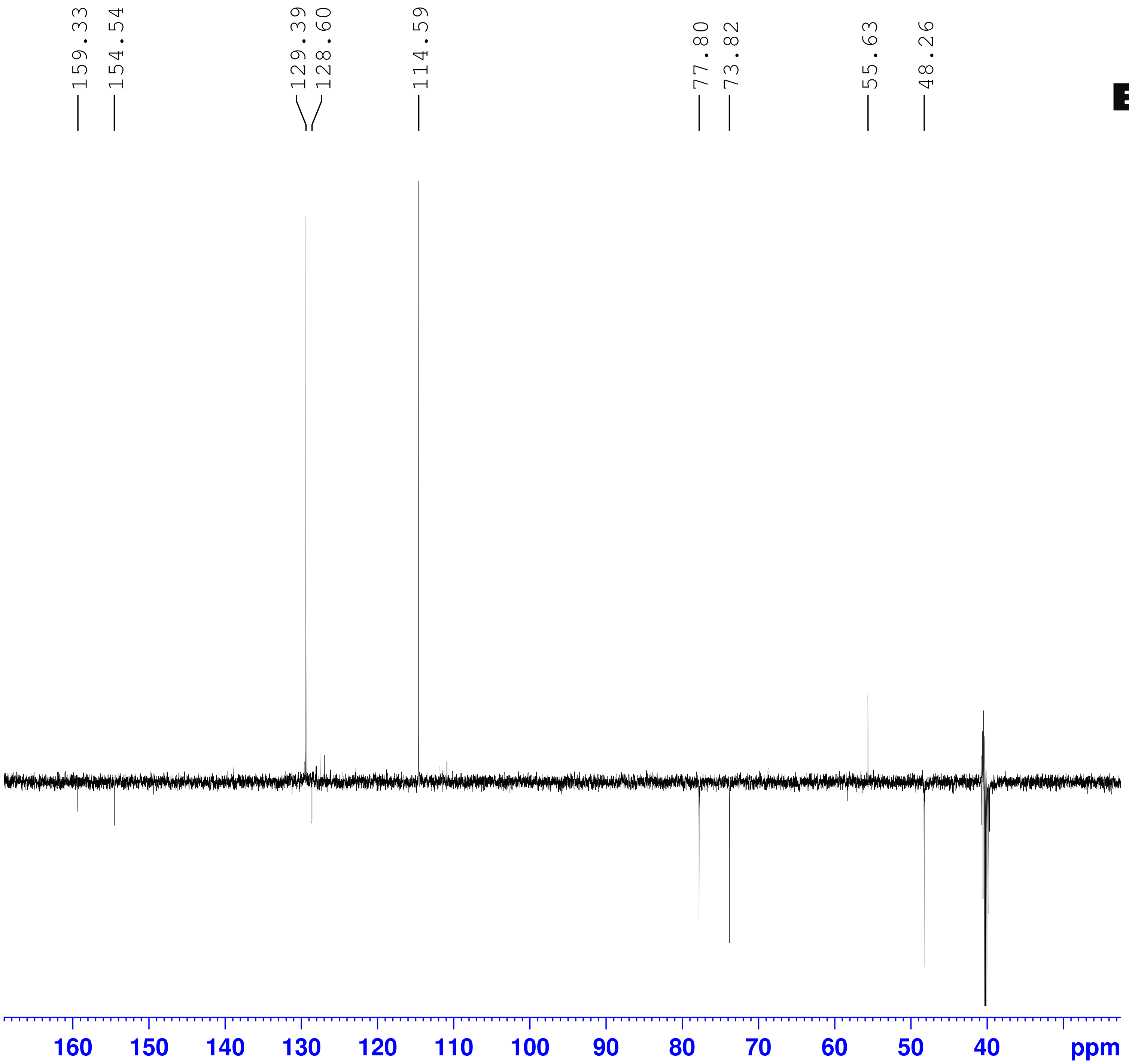

Current Data Parameters
NAME 23
EXPNO 2
PROCNO 1

F2 - Acquisition Parameters
Date_ 20181204
Time 11.49 h
INSTRUM spect
PROBHD Z119470_0187 (
PULPROG deptq135
TD 65536
SOLVENT DMSO
NS 104
DS 8
SWH 29761.904 Hz
FIDRES 0.908261 Hz
AQ 1.1010048 sec
RG 192.72
DW 16.800 usec
DE 6.50 usec
TE 323.2 K
CNST2 145.0000000
D1 2.00000000 sec
D2 0.00344828 sec
D12 0.00002000 sec
TD0 1
SFO1 125.7829381 MHz
NUC1 13C
P1 10.00 usec
P2 20.00 usec
PLW1 82.09700012 W
SFO2 500.1820007 MHz
NUC2 1H
CPDPRG[2 waltz16
P0 15.00 usec
P3 10.00 usec
P4 20.00 usec
PCPD2 80.00 usec
PLW2 18.10400009 W
PLW12 0.28288001 W

F2 - Processing parameters
SI 32768
SF 125.7703610 MHz
WDW EM
SSB 0
LB 1.00 Hz
GB 0
PC 1.40

^{13}C (DEPTQ135) NMR (125 MHz, DMSO-d_6, 50°C) of **23**

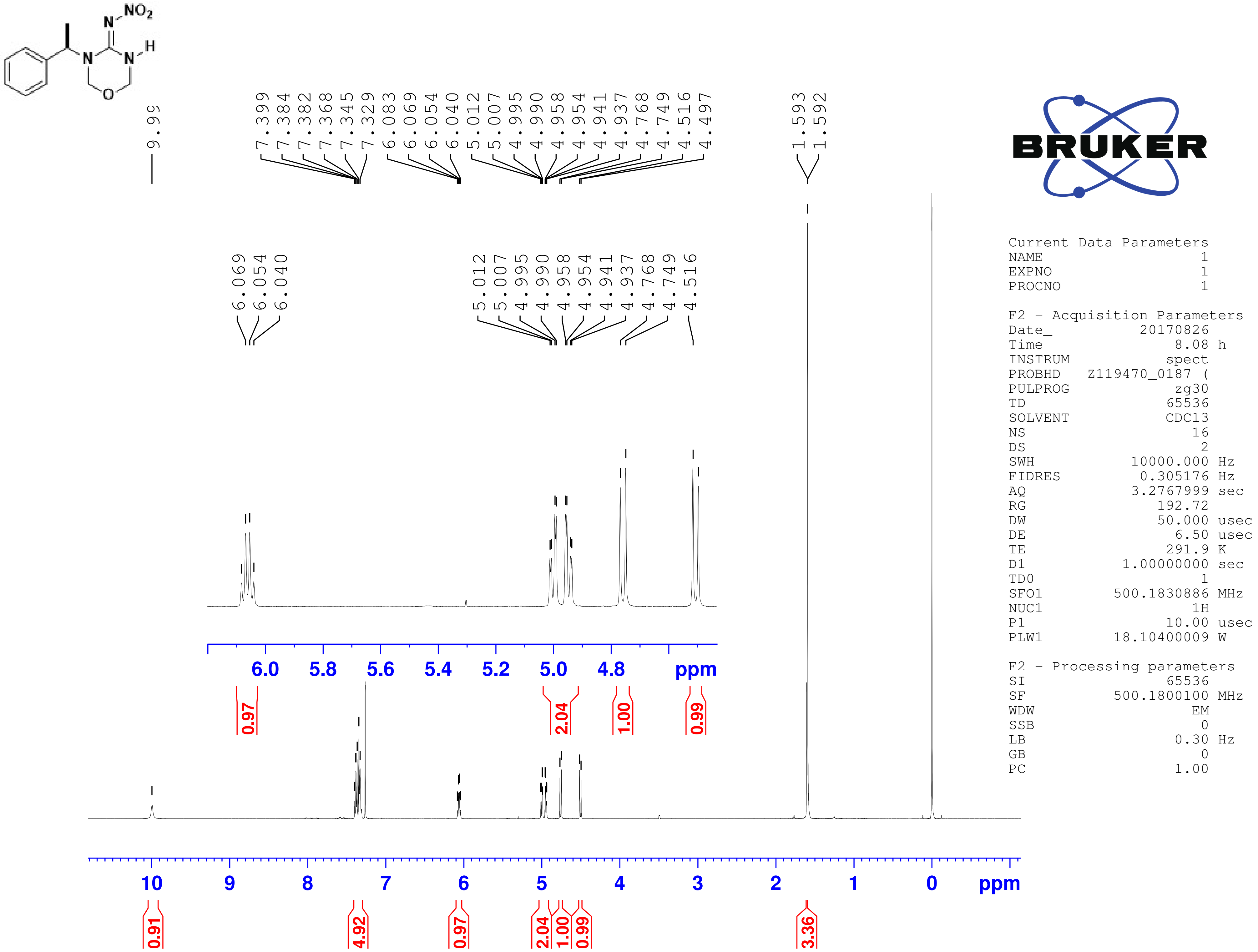

^{1}H NMR (500 MHz, $CDCl_3$) of ***R*-24**

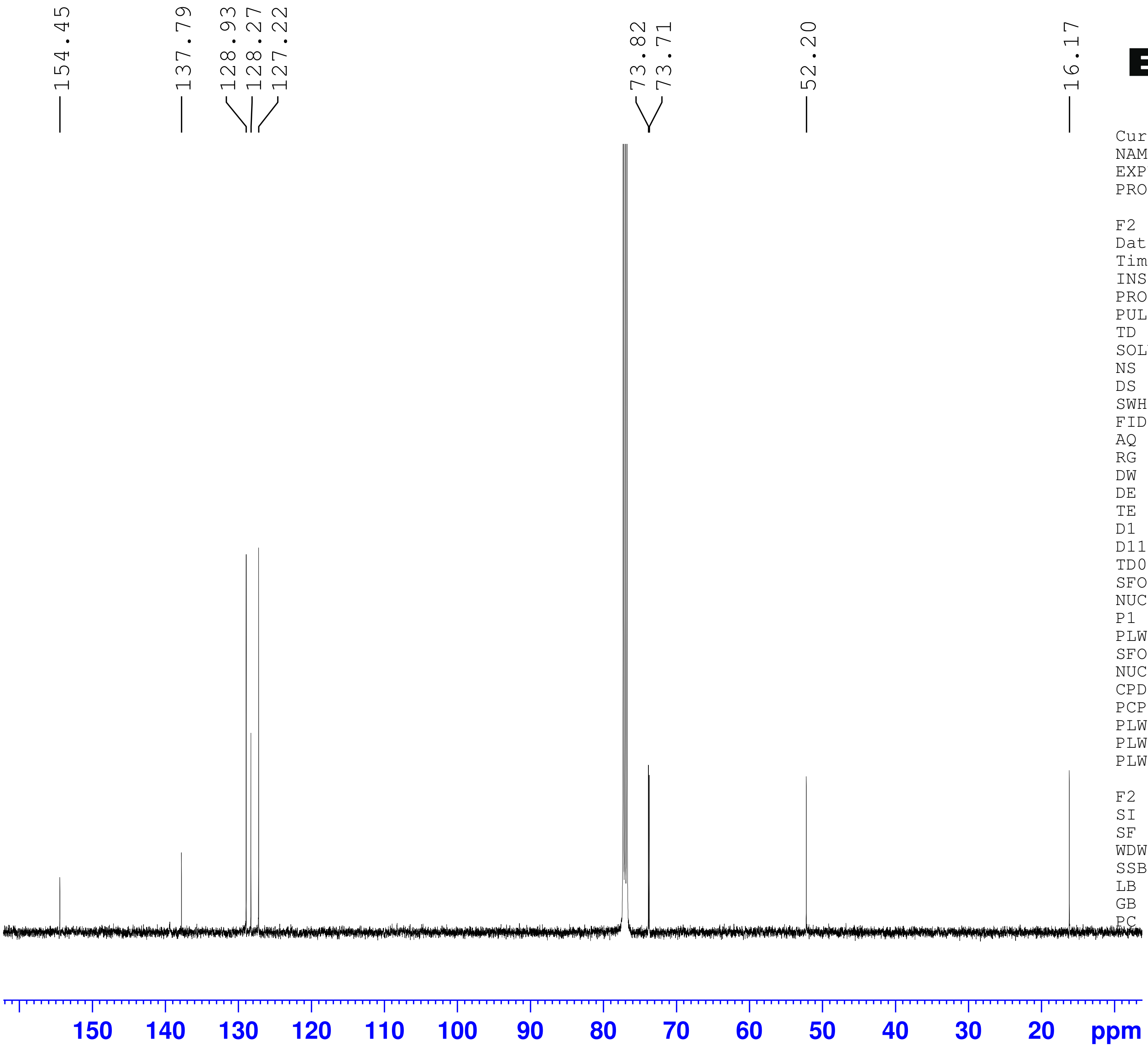


```
Current Data Parameters
NAME                 13C
EXPNO                  1
PROCNO                 1

F2 - Acquisition Parameters
Date_           20170826
Time                8.05 h
INSTRUM            spect
PROBHD    Z119470_0187 (
PULPROG          zgpg30
TD                 65536
SOLVENT            CDCl3
NS                  5120
DS                     4
SWH            29761.904 Hz
FIDRES          0.908261 Hz
AQ             1.1010048 sec
RG                192.72
DW                16.800 usec
DE                  6.50 usec
TE                 292.6 K
D1            2.00000000 sec
D11           0.03000000 sec
TD0                    1
SFO1         125.7829381 MHz
NUC1                 13C
P1                 10.00 usec
PLW1         82.09700012 W
SFO2         500.1820007 MHz
NUC2                  1H
CPDPRG[2         waltz16
PCPD2              80.00 usec
PLW2         18.10400009 W
PLW12         0.28680280 W
PLW13         0.14374560 W

F2 - Processing parameters
SI                 32768
SF           125.7703636 MHz
WDW                   EM
SSB                    0
LB                  1.00 Hz
GB                     0
PC                  1.40
```

^{13}C NMR (125 MHz, $CDCl_3$) of ***R*-24**

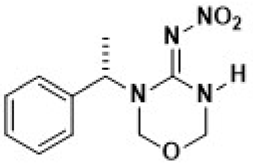

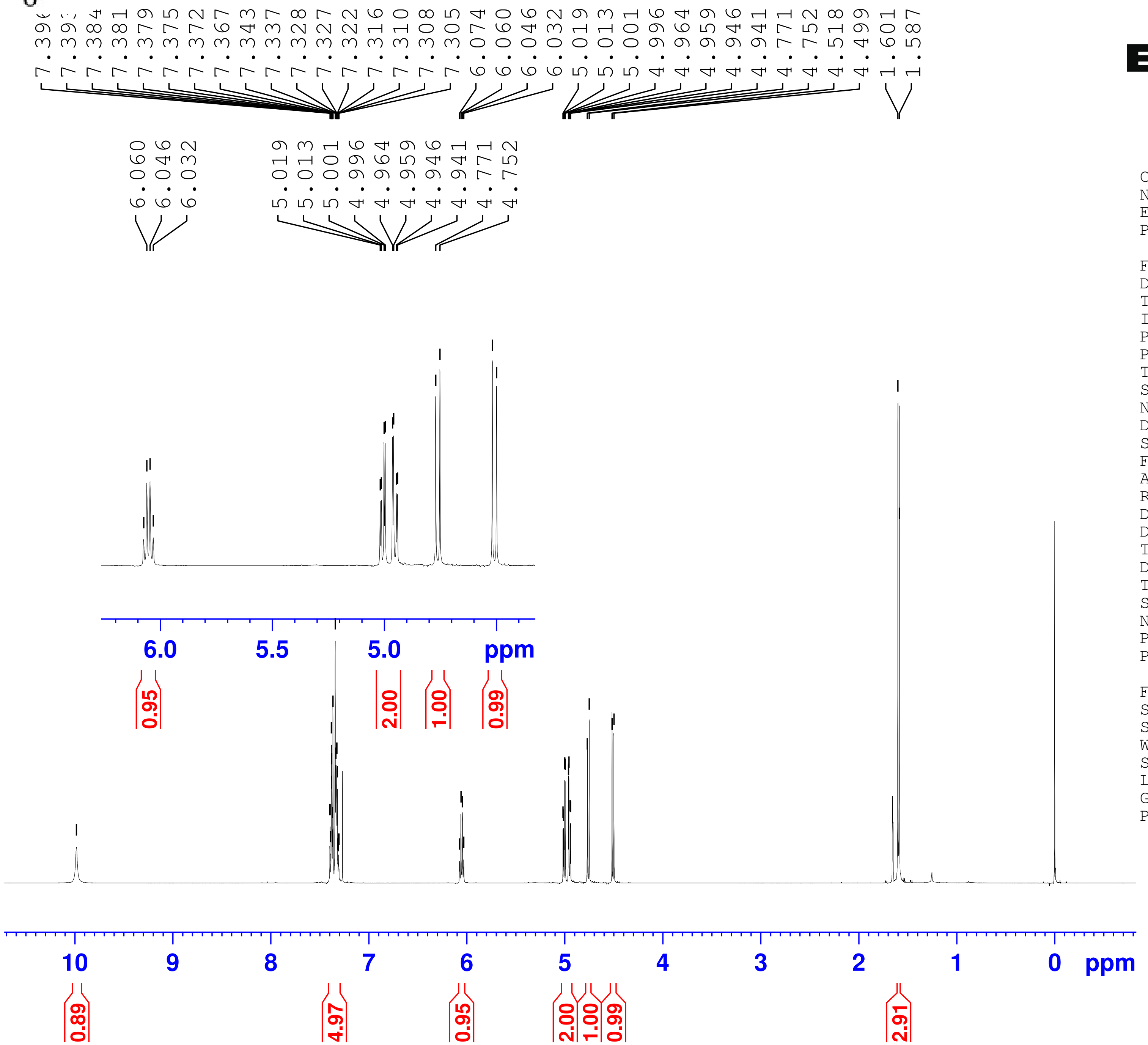


```
Current Data Parameters
NAME                  S-25
EXPNO                    1
PROCNO                   1

F2 - Acquisition Parameters
Date_             20170622
Time                 10.51 h
INSTRUM              spect
PROBHD    Z119470_0187 (
PULPROG               zg30
TD                   65536
SOLVENT              CDCl3
NS                      16
DS                       2
SWH              10000.000 Hz
FIDRES            0.305176 Hz
AQ               3.2767999 sec
RG                   95.16
DW                  50.000 usec
DE                    6.50 usec
TE                   291.3 K
D1              1.00000000 sec
TD0                      1
SFO1           500.1830886 MHz
NUC1                    1H
P1                   10.00 usec
PLW1           18.10400009 W

F2 - Processing parameters
SI                   65536
SF             500.1800074 MHz
WDW                     EM
SSB                      0
LB                    0.30 Hz
GB                       0
PC                    1.00
```


^{1}H NMR (500 MHz, $CDCl_3$) of ***S-25***

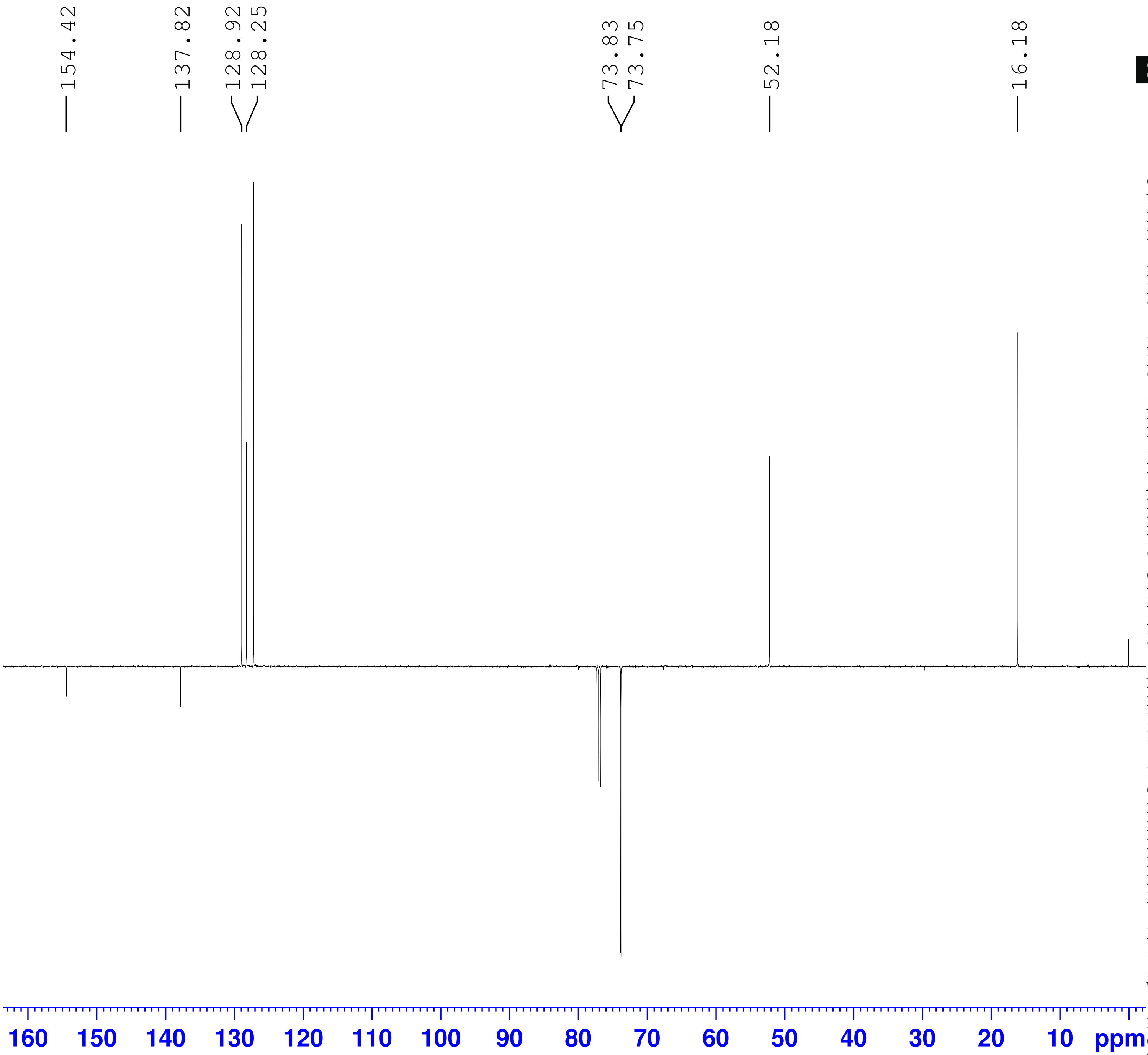

Current Data Parameters
NAME S-25
EXPNO 2
PROCNO 1

F2 - Acquisition Parameters
Date_ 20170622
Time 14.35 h
INSTRUM spect
PROBHD Z119470_0187 (
PULPROG deptq135
TD 65536
SOLVENT CDCl3
NS 4097
DS 8
SWH 29761.904 Hz
FIDRES 0.908261 Hz
AQ 1.1010048 sec
RG 192.72
DW 16.800 usec
DE 6.50 usec
TE 291.7 K
CNST2 145.0000000
D1 2.00000000 sec
D2 0.00344828 sec
D12 0.00002000 sec
TD0 1
SFO1 125.7829381 MHz
NUC1 13C
P1 10.00 usec
P2 20.00 usec
PLW1 82.09700012 W
SFO2 500.1820007 MHz
NUC2 1H
CPDPRG[2 waltz16
P0 15.00 usec
P3 10.00 usec
P4 20.00 usec
PCPD2 80.00 usec
PLW2 18.10400009 W
PLW12 0.28680280 W

F2 - Processing parameters
SI 32768
SF 125.7703641 MHz
WDW EM
SSB 0
LB 1.00 Hz
GB 0
PC 1.40

^{13}C (DEPTQ135) NMR (125 MHz, $CDCl_3$) of ***S*-25**

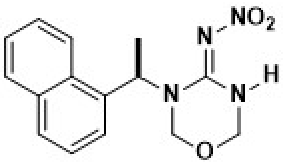

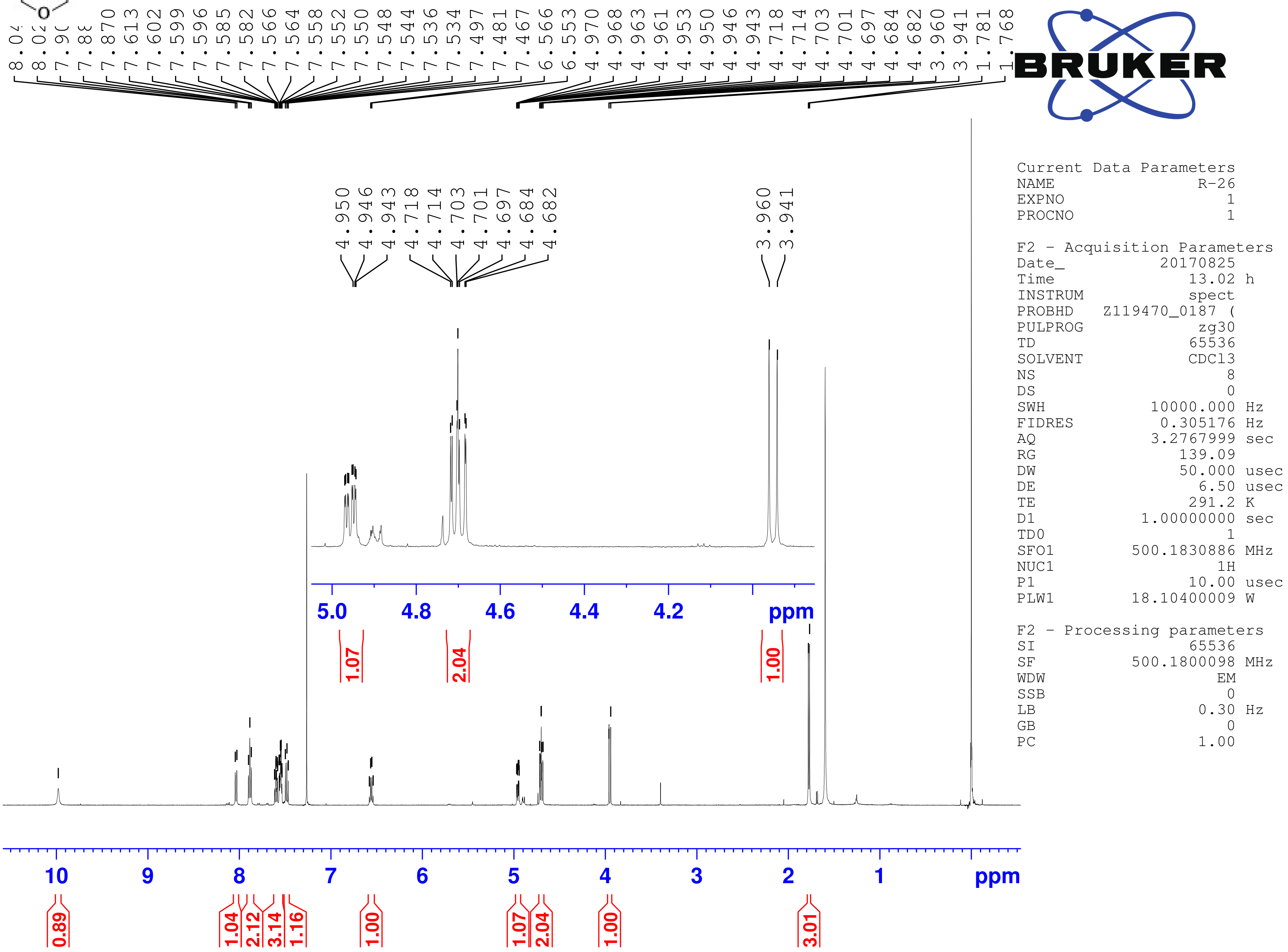

^1H NMR (500 MHz, $CDCl_3$) of ***R*-26**

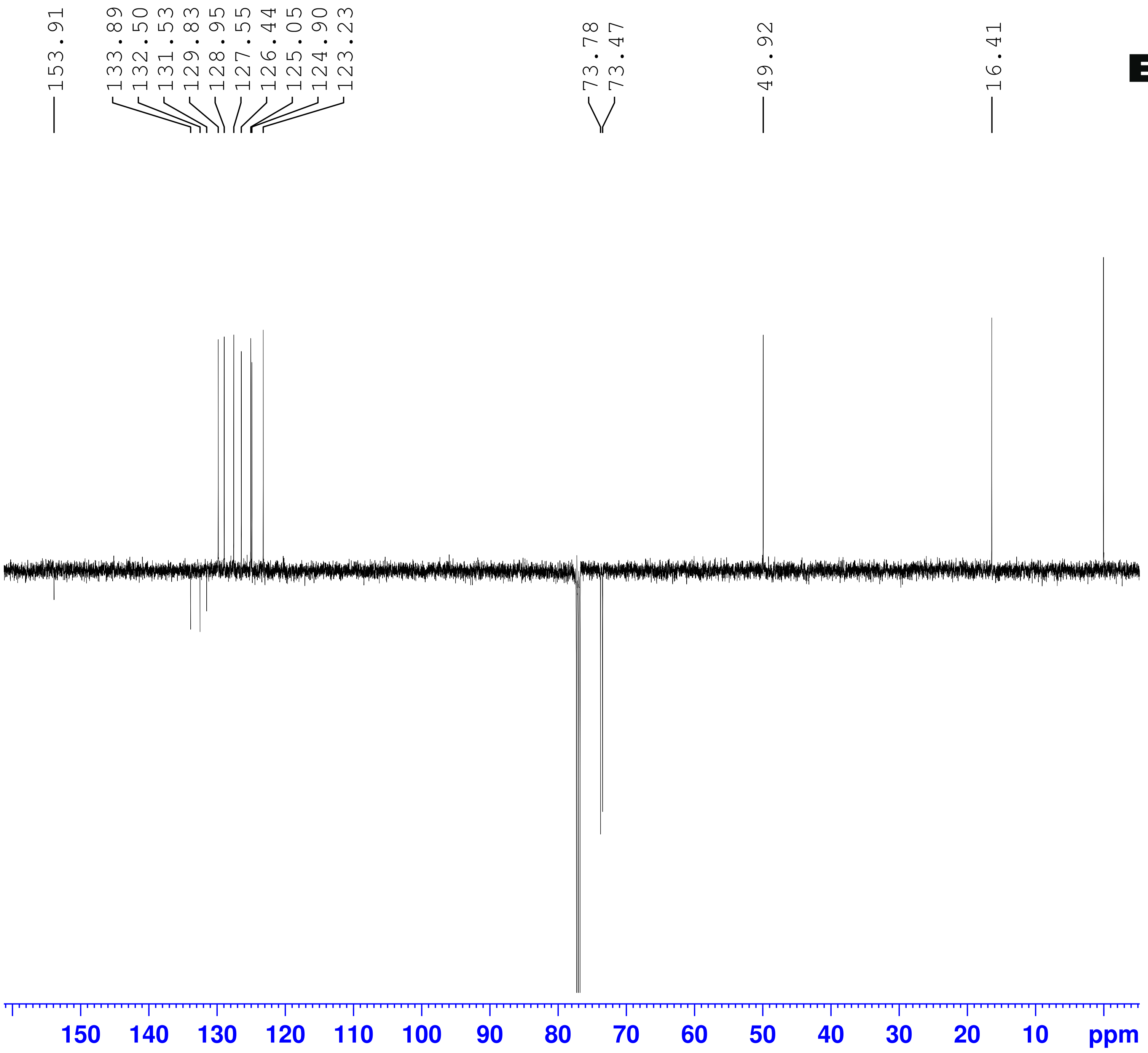

BRUKER

```
Current Data Parameters
NAME                 R-26
EXPNO                   2
PROCNO                  1

F2 - Acquisition Parameters
Date_            20170825
Time                14.23 h
INSTRUM             spect
PROBHD      Z119470_0187 (
PULPROG          deptq135
TD                  65536
SOLVENT             CDCl3
NS                   1479
DS                      8
SWH             29761.904 Hz
FIDRES           0.908261 Hz
AQ              1.1010048 sec
RG                 192.72
DW                 16.800 usec
DE                   6.50 usec
TE                  291.6 K
CNST2         145.0000000
D1             2.00000000 sec
D2             0.00344828 sec
D12            0.00002000 sec
TD0                     1
SFO1          125.7829381 MHz
NUC1                  13C
P1                  10.00 usec
P2                  20.00 usec
PLW1          82.09700012 W
SFO2          500.1820007 MHz
NUC2                   1H
CPDPRG[2          waltz16
P0                  15.00 usec
P3                  10.00 usec
P4                  20.00 usec
PCPD2               80.00 usec
PLW2          18.10400009 W
PLW12          0.28680280 W

F2 - Processing parameters
SI                  32768
SF            125.7703610 MHz
WDW                    EM
SSB                     0
LB                   1.00 Hz
GB                      0
PC                   1.40
```

^{13}C (DEPTQ135) NMR (125 MHz, $CDCl_3$) of ***R*-26**

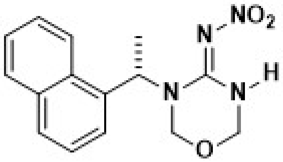

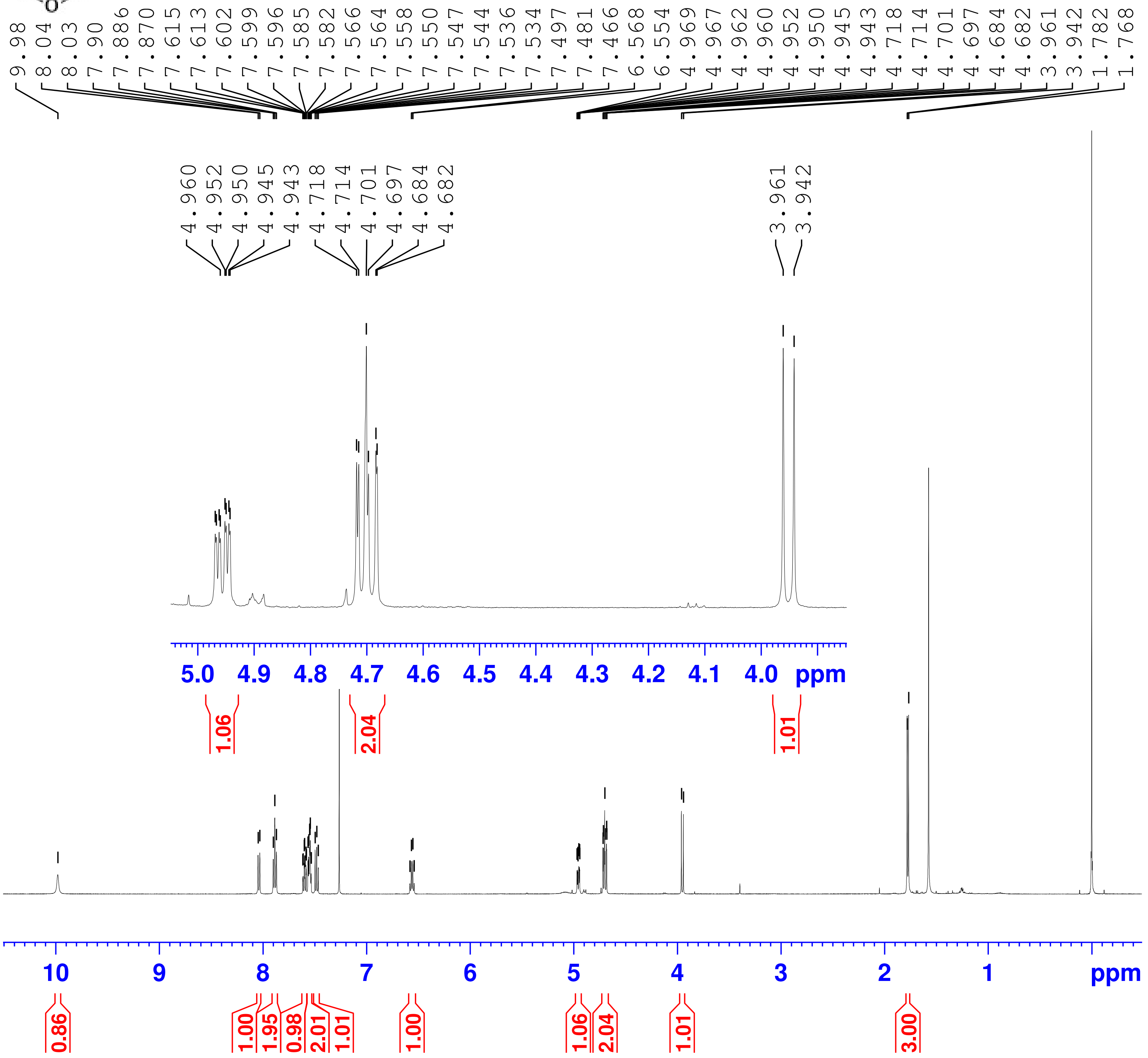


```
Current Data Parameters
NAME                1H
EXPNO                1
PROCNO               1

F2 - Acquisition Parameters
Date_          20170826
Time            20.01 h
INSTRUM           spect
PROBHD     Z119470_0187 (
PULPROG            zg30
TD                65536
SOLVENT           CDCl3
NS                   16
DS                    2
SWH           10000.000 Hz
FIDRES         0.305176 Hz
AQ            3.2767999 sec
RG               192.72
DW               50.000 usec
DE                 6.50 usec
TE                292.1 K
D1           1.00000000 sec
TD0                   1
SFO1        500.1830886 MHz
NUC1                 1H
P1                10.00 usec
PLW1        18.10400009 W

F2 - Processing parameters
SI                65536
SF          500.1800101 MHz
WDW                  EM
SSB                   0
LB                 0.30 Hz
GB                    0
PC                 1.00
```

^{1}H NMR (500 MHz, $CDCl_3$) of ***S*-27**

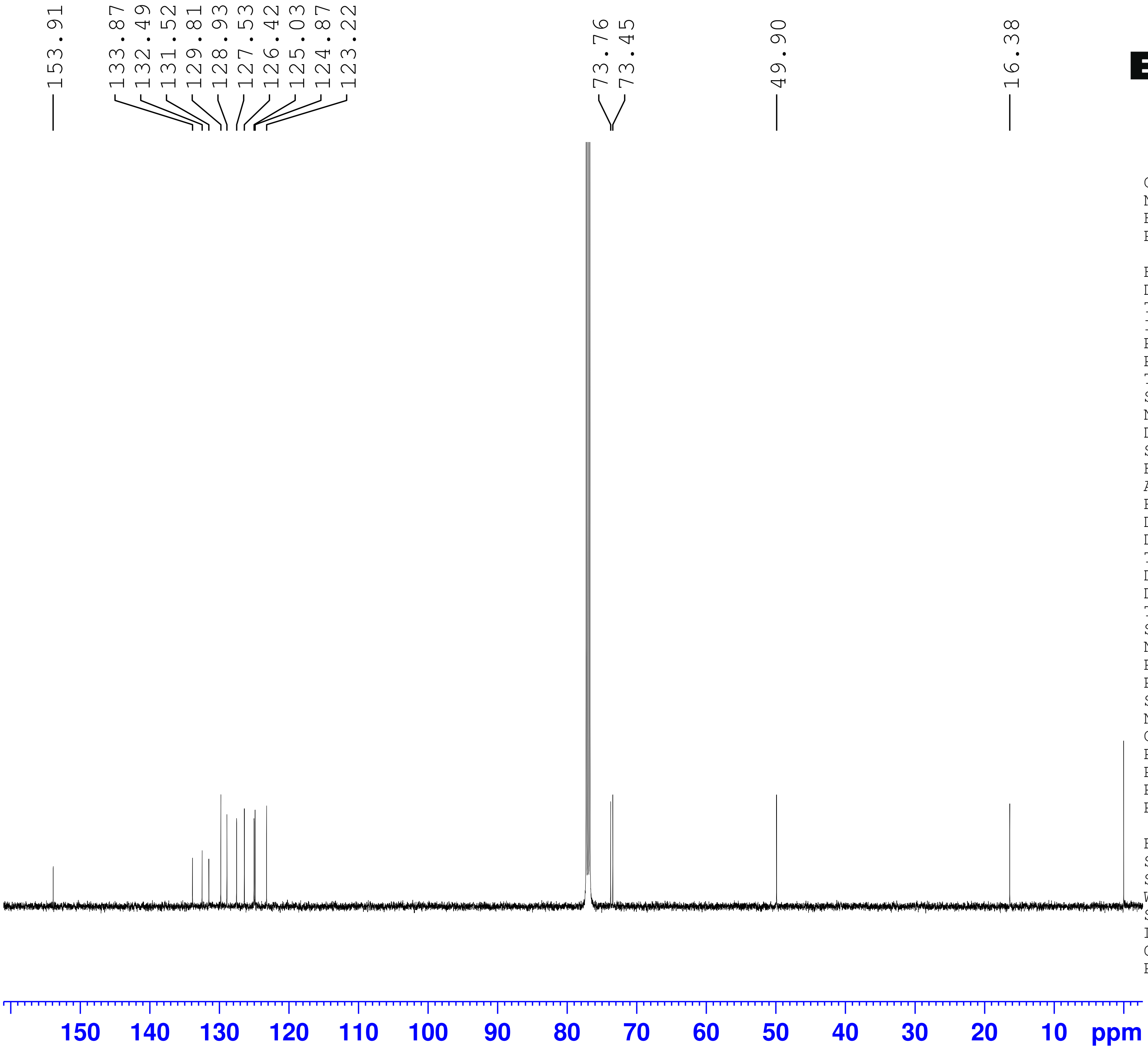

Current Data Parameters
NAME 13C
EXPNO 1
PROCNO 1

F2 - Acquisition Parameters
Date_ 20170826
Time 19.59 h
INSTRUM spect
PROBHD Z119470_0187 (
PULPROG zgpg30
TD 65536
SOLVENT CDCl3
NS 5120
DS 4
SWH 29761.904 Hz
FIDRES 0.908261 Hz
AQ 1.1010048 sec
RG 192.72
DW 16.800 usec
DE 6.50 usec
TE 292.8 K
D1 2.00000000 sec
D11 0.03000000 sec
TD0 1
SFO1 125.7829381 MHz
NUC1 13C
P1 10.00 usec
PLW1 82.09700012 W
SFO2 500.1820007 MHz
NUC2 1H
CPDPRG[2 waltz16
PCPD2 80.00 usec
PLW2 18.10400009 W
PLW12 0.28680280 W
PLW13 0.14374560 W

F2 - Processing parameters
SI 32768
SF 125.7703632 MHz
WDW EM
SSB 0
LB 1.00 Hz
GB 0
PC 1.40

^{13}C NMR (125 MHz, $CDCl_3$) of ***S*-27**

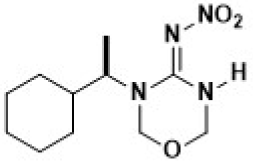

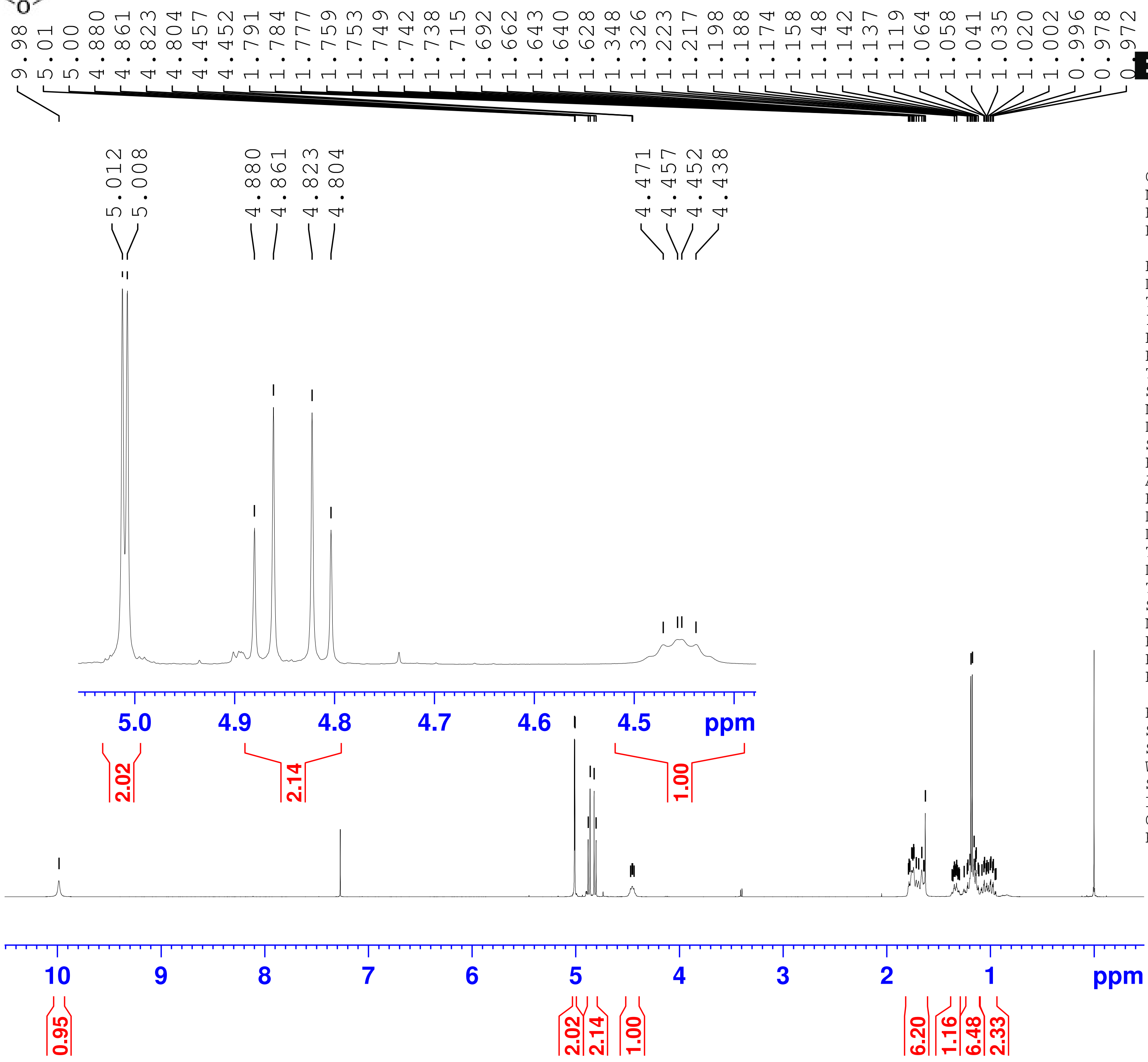

Current Data Parameters
NAME 1H
EXPNO 1
PROCNO 1

F2 - Acquisition Parameters
Date_ 20181101
Time 11.56 h
INSTRUM spect
PROBHD Z119470_0187 (
PULPROG zg30
TD 65536
SOLVENT CDCl3
NS 5
DS 1
SWH 10000.000 Hz
FIDRES 0.305176 Hz
AQ 3.2767999 sec
RG 95.16
DW 50.000 usec
DE 6.50 usec
TE 292.1 K
D1 1.00000000 sec
TD0 1
SFO1 500.1830886 MHz
NUC1 1H
P0 3.33 usec
P1 10.00 usec
PLW1 18.10400009 W

F2 - Processing parameters
SI 65536
SF 500.1800069 MHz
WDW EM
SSB 0
LB 0.30 Hz
GB 0
PC 1.00

^{1}H NMR (500 MHz, $CDCl_3$) of ***R*-28**

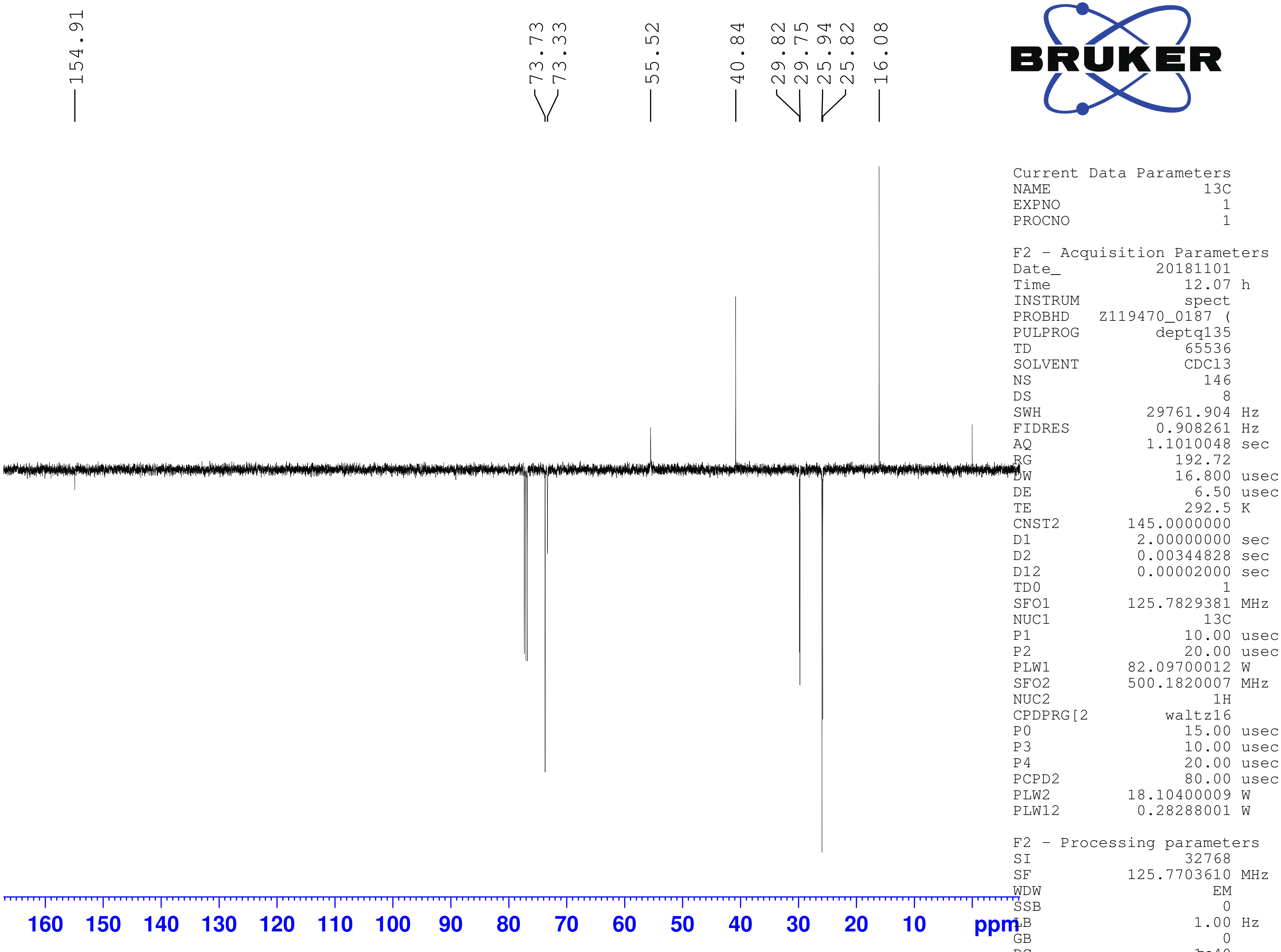

^{13}C (DEPTQ135) NMR (125 MHz, $CDCl_3$) of ***R*-28**

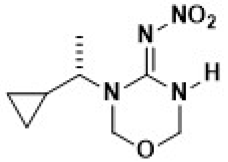

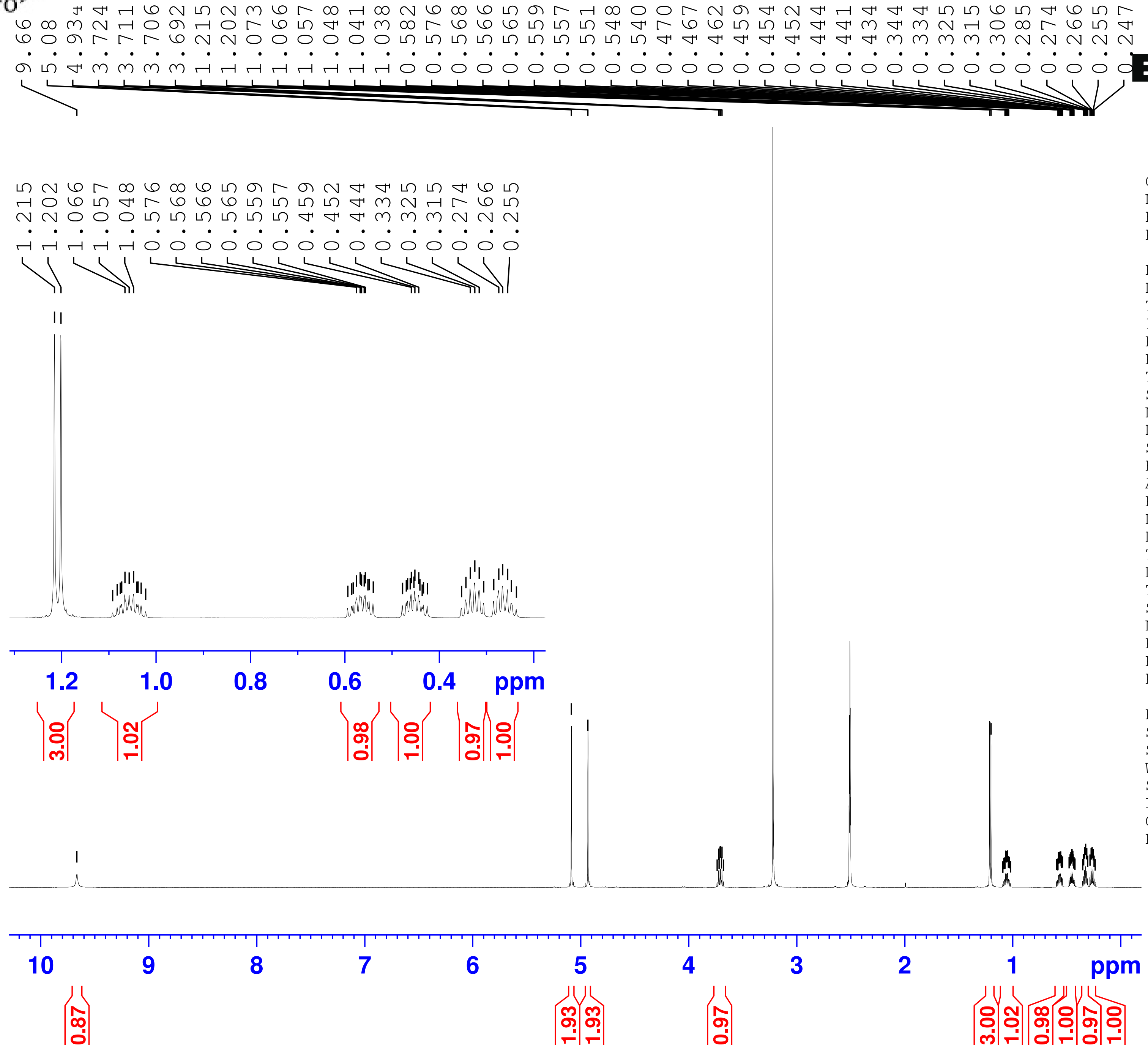

Current Data Parameters

NAME	S-29
EXPNO	1
PROCNO	1

F2 - Acquisition Parameters

Date_	20180920
Time	11.21 h
INSTRUM	spect
PROBHD	Z119470_0187 (
PULPROG	zg30
TD	65536
SOLVENT	DMSO
NS	5
DS	1
SWH	10000.000 Hz
FIDRES	0.305176 Hz
AQ	3.2767999 sec
RG	108.3
DW	50.000 usec
DE	6.50 usec
TE	323.1 K
D1	1.00000000 sec
TD0	1
SFO1	500.1830886 MHz
NUC1	1H
P0	3.33 usec
P1	10.00 usec
PLW1	18.10400009 W

F2 - Processing parameters

SI	65536
SF	500.1800000 MHz
WDW	EM
SSB	0
LB	0.30 Hz
GB	0
PC	1.00

^{1}H NMR (500 MHz, DMSO-d_6, 50°C) of ***S*-29**

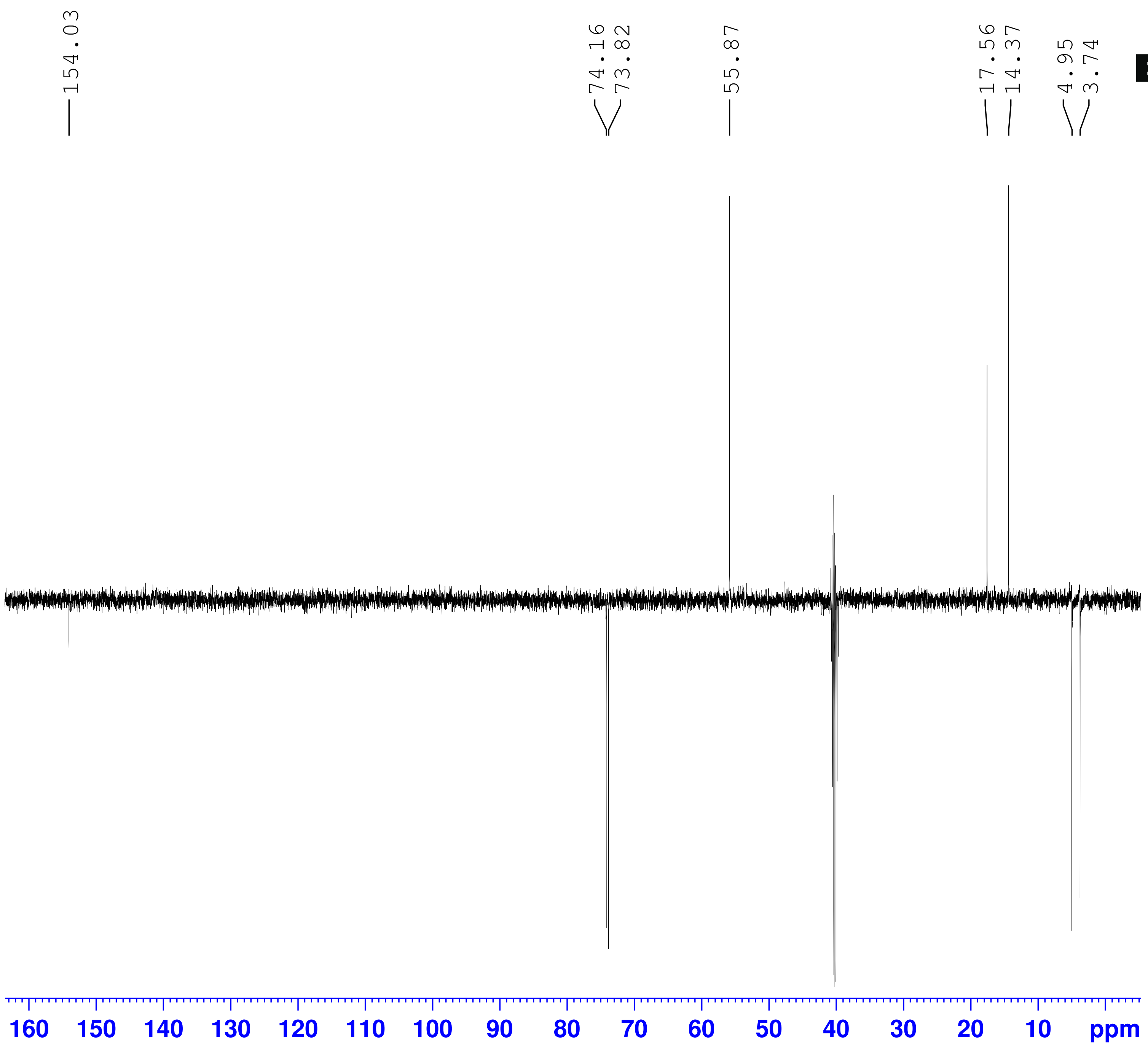

BRUKER

Current Data Parameters
NAME S-29
EXPNO 2
PROCNO 1

F2 - Acquisition Parameters
Date_ 20180920
Time 11.32 h
INSTRUM spect
PROBHD Z119470_0187 (
PULPROG deptq135
TD 65536
SOLVENT DMSO
NS 185
DS 8
SWH 29761.904 Hz
FIDRES 0.908261 Hz
AQ 1.1010048 sec
RG 192.72
DW 16.800 usec
DE 6.50 usec
TE 323.2 K
CNST2 145.0000000
D1 2.00000000 sec
D2 0.00344828 sec
D12 0.00002000 sec
TD0 1
SFO1 125.7829381 MHz
NUC1 13C
P1 10.00 usec
P2 20.00 usec
PLW1 82.09700012 W
SFO2 500.1820007 MHz
NUC2 1H
CPDPRG[2 waltz16
P0 15.00 usec
P3 10.00 usec
P4 20.00 usec
PCPD2 80.00 usec
PLW2 18.10400009 W
PLW12 0.28288001 W

F2 - Processing parameters
SI 32768
SF 125.7703610 MHz
WDW EM
SSB 0
LB 1.00 Hz
GB 0
PC 1.40

^{13}C (DEPTQ135) NMR (125 MHz, DMSO-d_6, 50°C) of ***S*-29**

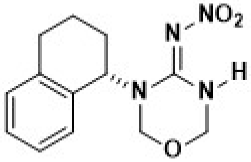

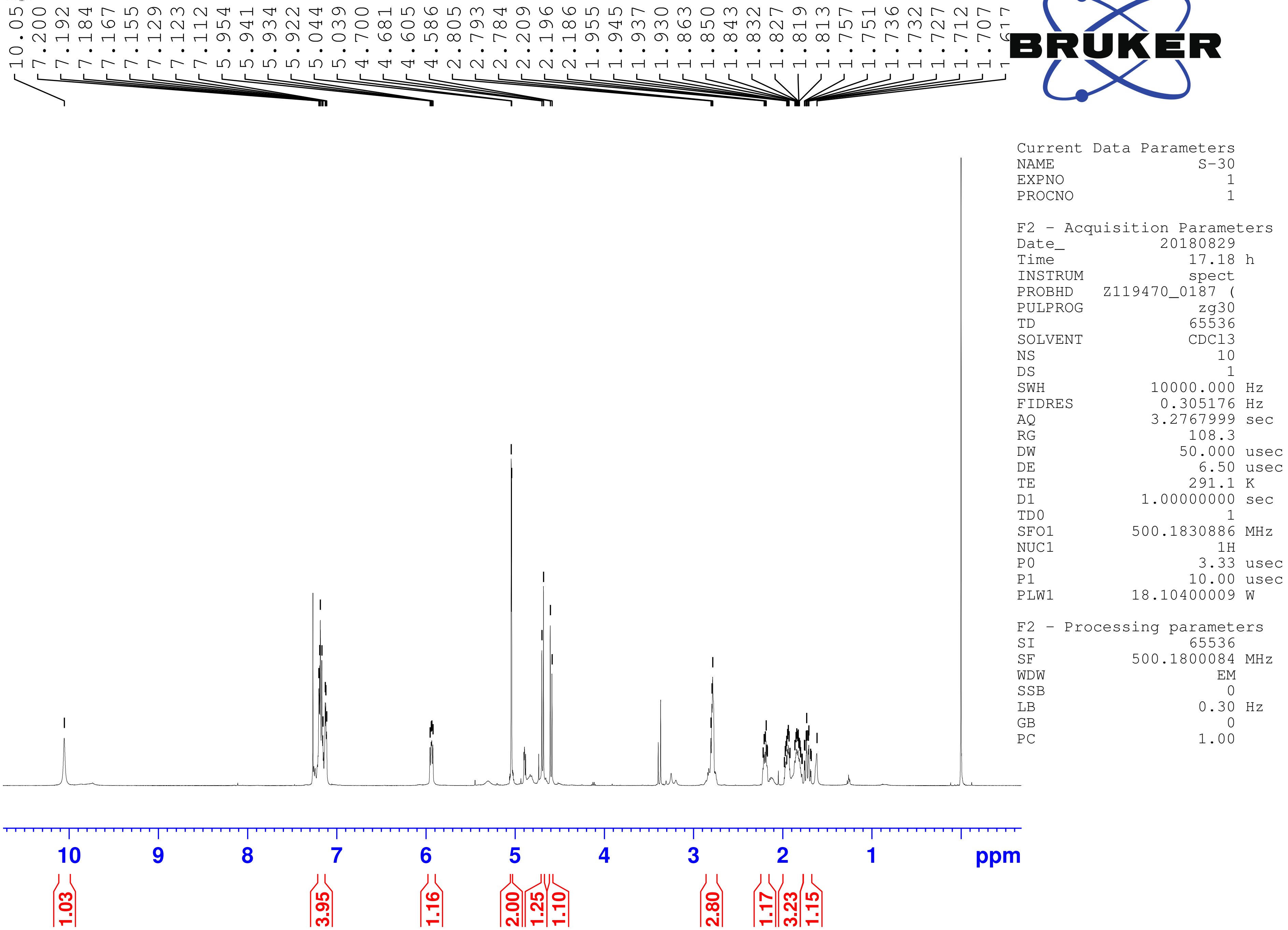

^{1}H NMR (500 MHz, $CDCl_3$) of ***S*-30**

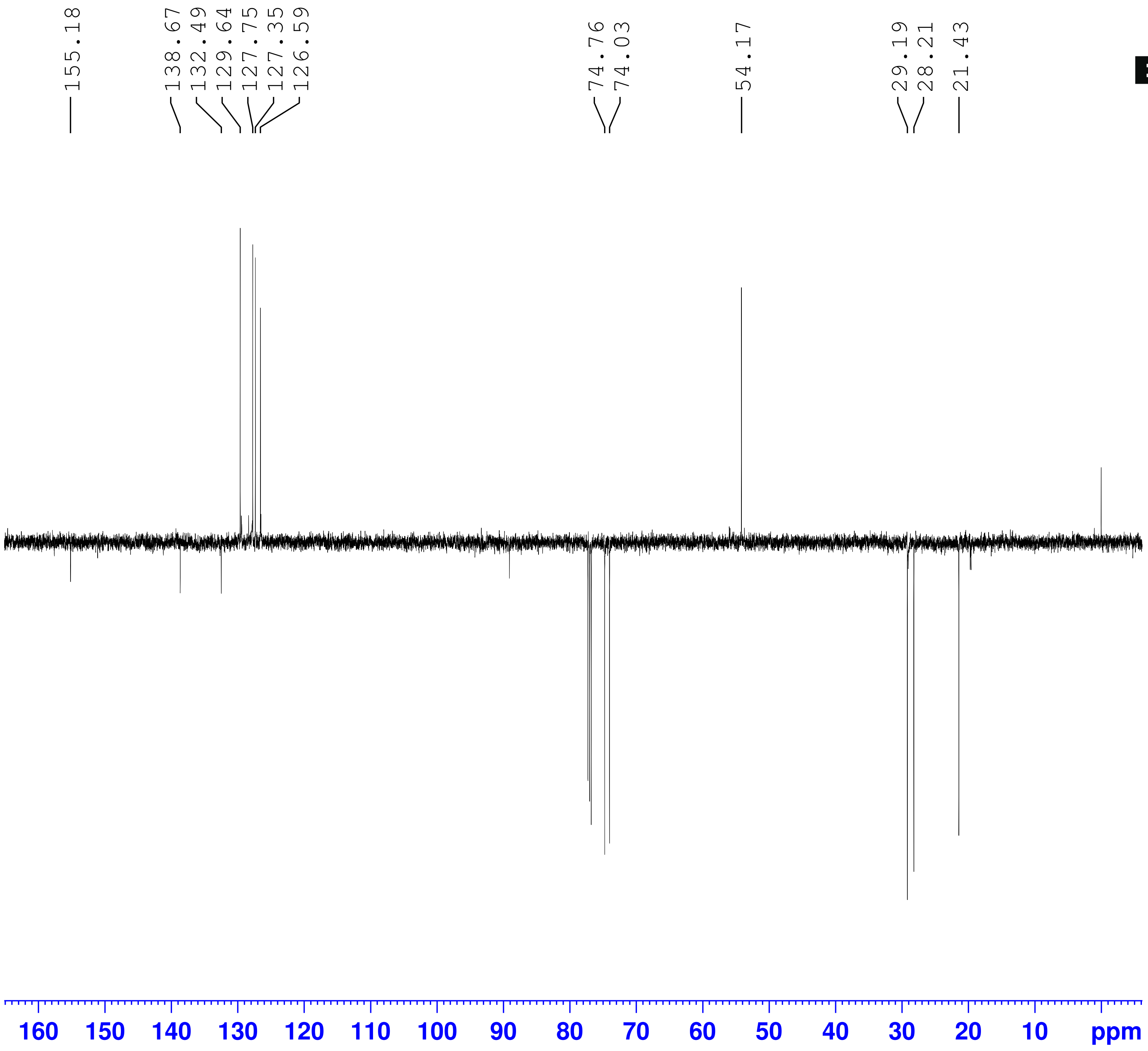

Current Data Parameters
NAME S-30
EXPNO 2
PROCNO 1

F2 - Acquisition Parameters
Date_ 20180829
Time 17.29 h
INSTRUM spect
PROBHD Z119470_0187 (
PULPROG deptq135
TD 65536
SOLVENT CDCl3
NS 157
DS 8
SWH 29761.904 Hz
FIDRES 0.908261 Hz
AQ 1.1010048 sec
RG 192.72
DW 16.800 usec
DE 6.50 usec
TE 291.7 K
CNST2 145.0000000
D1 2.00000000 sec
D2 0.00344828 sec
D12 0.00002000 sec
TD0 1
SFO1 125.7829381 MHz
NUC1 13C
P1 10.00 usec
P2 20.00 usec
PLW1 82.09700012 W
SFO2 500.1820007 MHz
NUC2 1H
CPDPRG[2 waltz16
P0 15.00 usec
P3 10.00 usec
P4 20.00 usec
PCPD2 80.00 usec
PLW2 18.10400009 W
PLW12 0.28288001 W

F2 - Processing parameters
SI 32768
SF 125.7703642 MHz
WDW EM
SSB 0
LB 1.00 Hz
GB 0
PC 1.40

^{13}C (DEPTQ135) NMR (125 MHz, $CDCl_3$) of ***S*-30**

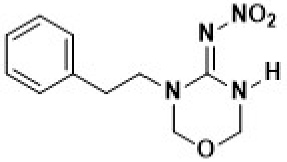

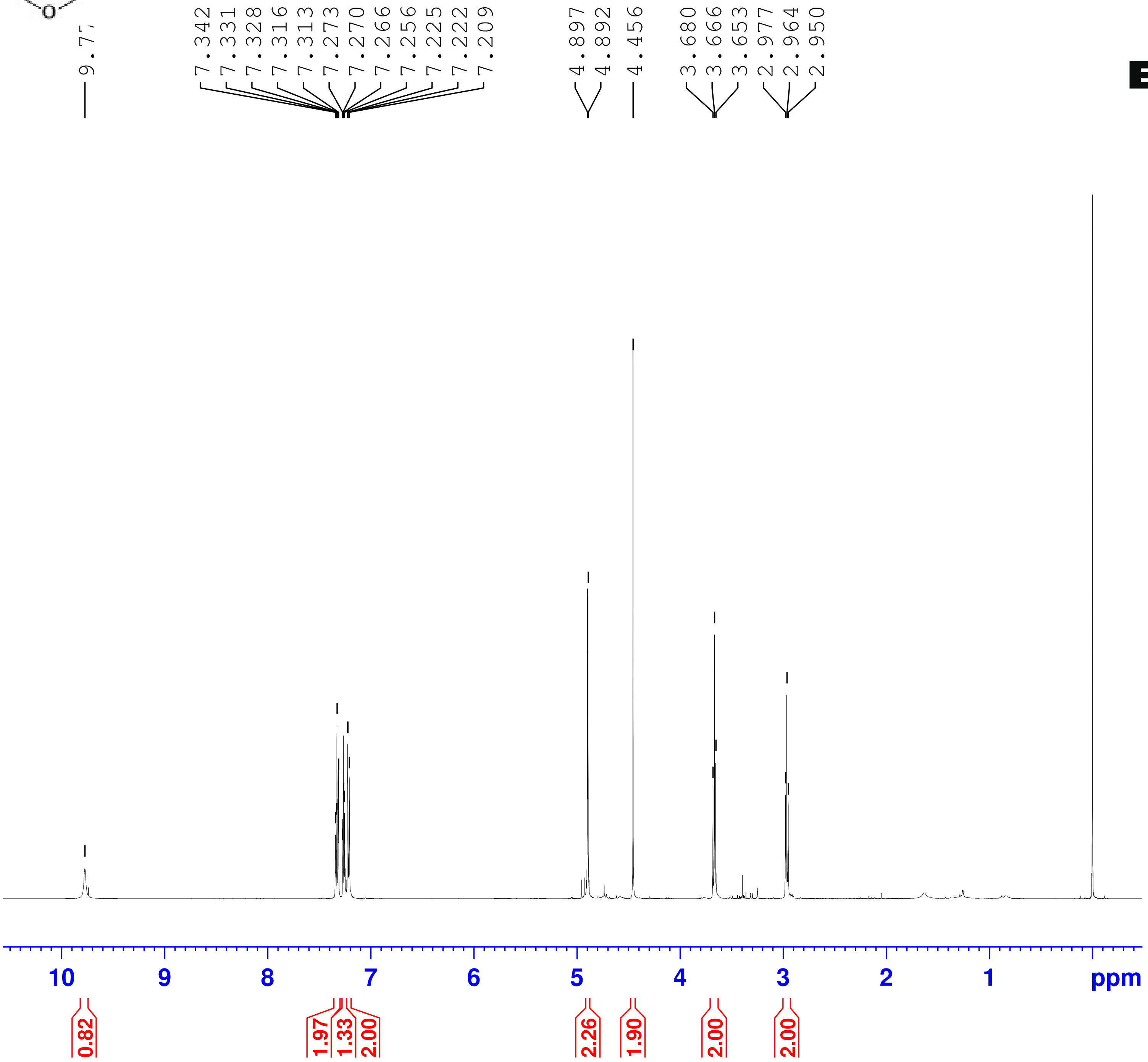


```
Current Data Parameters
NAME                R-31
EXPNO                  1
PROCNO                 1

F2 - Acquisition Parameters
Date_           20180829
Time               17.35 h
INSTRUM            spect
PROBHD    Z119470_0187 (
PULPROG             zg30
TD                 65536
SOLVENT            CDCl3
NS                    16
DS                     2
SWH            10000.000 Hz
FIDRES          0.305176 Hz
AQ             3.2767999 sec
RG                139.09
DW                50.000 usec
DE                  6.50 usec
TE                 291.8 K
D1            1.00000000 sec
TD0                    1
SFO1         500.1830886 MHz
NUC1                  1H
P0                  3.33 usec
P1                 10.00 usec
PLW1         18.10400009 W

F2 - Processing parameters
SI                 65536
SF           500.1800090 MHz
WDW                   EM
SSB                    0
LB                  0.30 Hz
GB                     0
PC                  1.00
```


^{1}H NMR (500 MHz, $CDCl_3$) of ***R*-31**

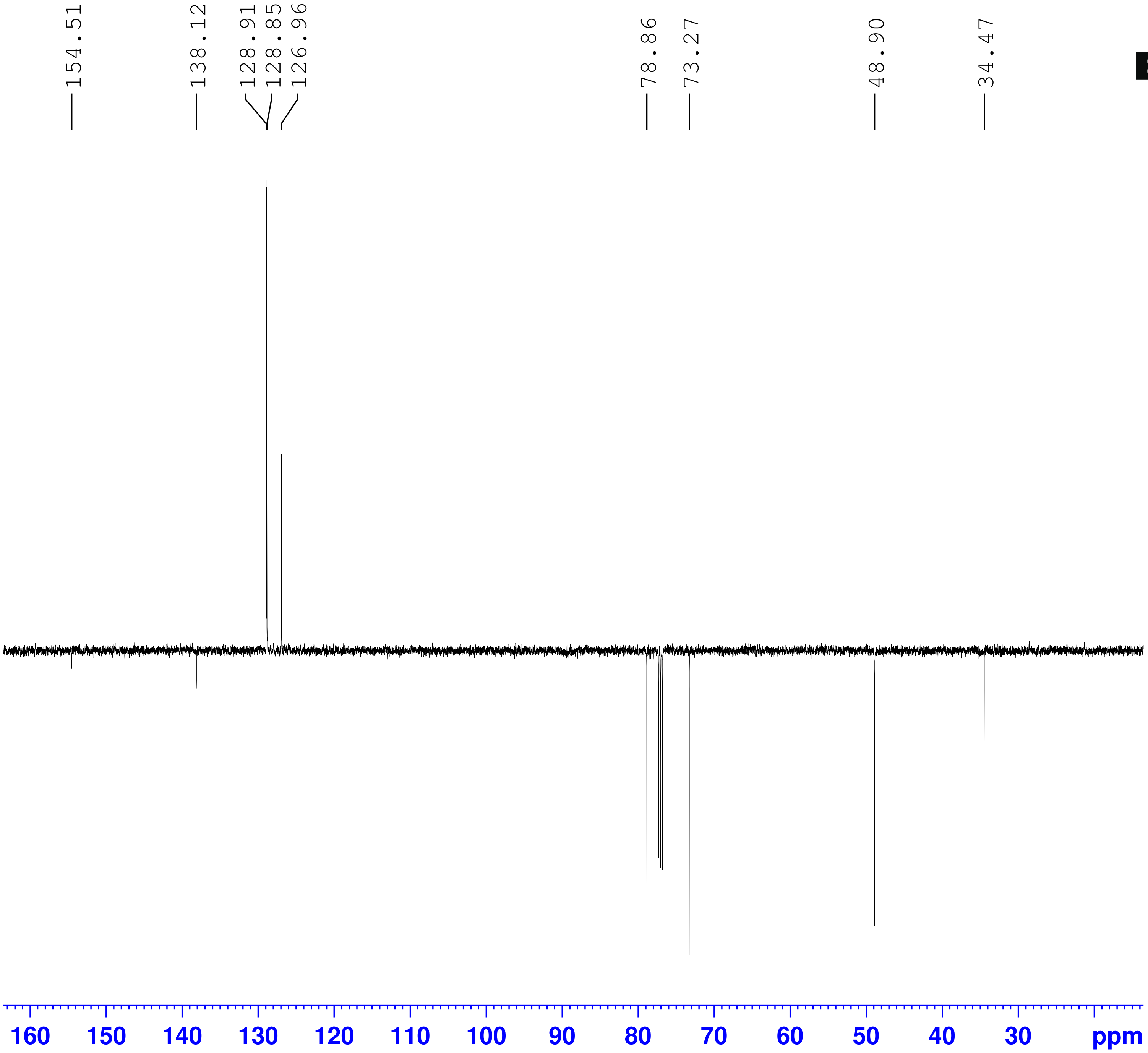

Current Data Parameters
NAME R-31
EXPNO 2
PROCNO 1

F2 - Acquisition Parameters
Date_ 20180829
Time 17.50 h
INSTRUM spect
PROBHD Z119470_0187 (
PULPROG deptq135
TD 65536
SOLVENT CDCl3
NS 256
DS 8
SWH 29761.904 Hz
FIDRES 0.908261 Hz
AQ 1.1010048 sec
RG 192.72
DW 16.800 usec
DE 6.50 usec
TE 292.3 K
CNST2 145.0000000
D1 2.00000000 sec
D2 0.00344828 sec
D12 0.00002000 sec
TD0 1
SFO1 125.7829381 MHz
NUC1 13C
P1 10.00 usec
P2 20.00 usec
PLW1 82.09700012 W
SFO2 500.1820007 MHz
NUC2 1H
CPDPRG[2 waltz16
P0 15.00 usec
P3 10.00 usec
P4 20.00 usec
PCPD2 80.00 usec
PLW2 18.10400009 W
PLW12 0.28288001 W

F2 - Processing parameters
SI 32768
SF 125.7703641 MHz
WDW EM
SSB 0
LB 1.00 Hz
GB 0
PC 1.40

^{13}C (DEPTQ135) NMR (500 MHz, $CDCl_3$) of ***R*-31**

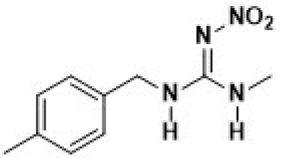

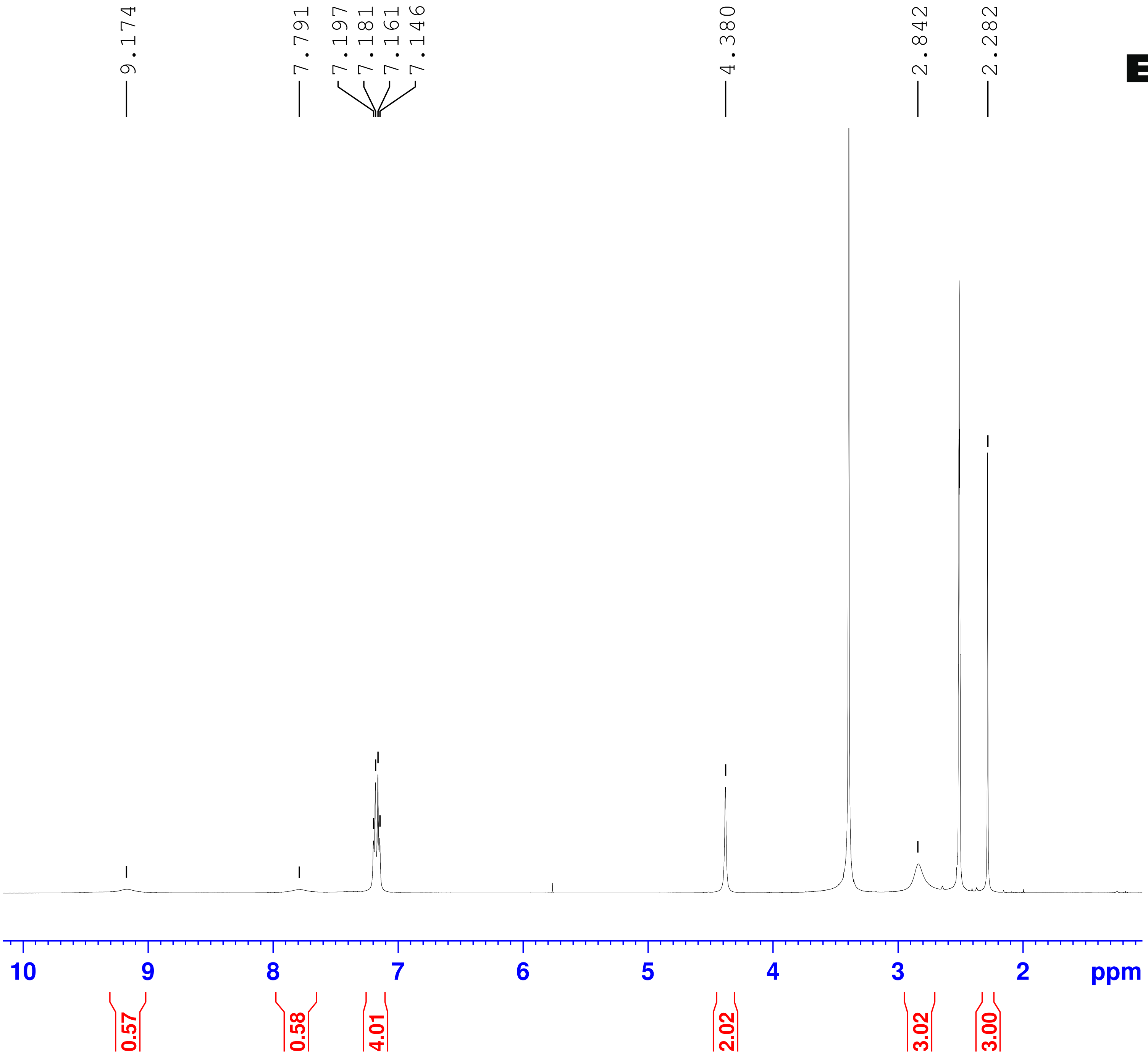


```
Current Data Parameters
NAME                    33
EXPNO                    1
PROCNO                   1

F2 - Acquisition Parameters
Date_             20181012
Time                 18.21 h
INSTRUM              spect
PROBHD    Z119470_0187 (
PULPROG               zg30
TD                   65536
SOLVENT               DMSO
NS                      16
DS                       1
SWH              10000.000 Hz
FIDRES            0.305176 Hz
AQ               3.2767999 sec
RG                   77.78
DW                  50.000 usec
DE                    6.50 usec
TE                   292.0 K
D1              1.00000000 sec
TD0                      1
SFO1           500.1830886 MHz
NUC1                    1H
P0                    3.33 usec
P1                   10.00 usec
PLW1           18.10400009 W

F2 - Processing parameters
SI                   65536
SF             500.1800000 MHz
WDW                     EM
SSB                      0
LB                    0.30 Hz
GB                       0
PC                    1.00
```


^{1}H NMR (500 MHz, DMSO-d_6) of **33**

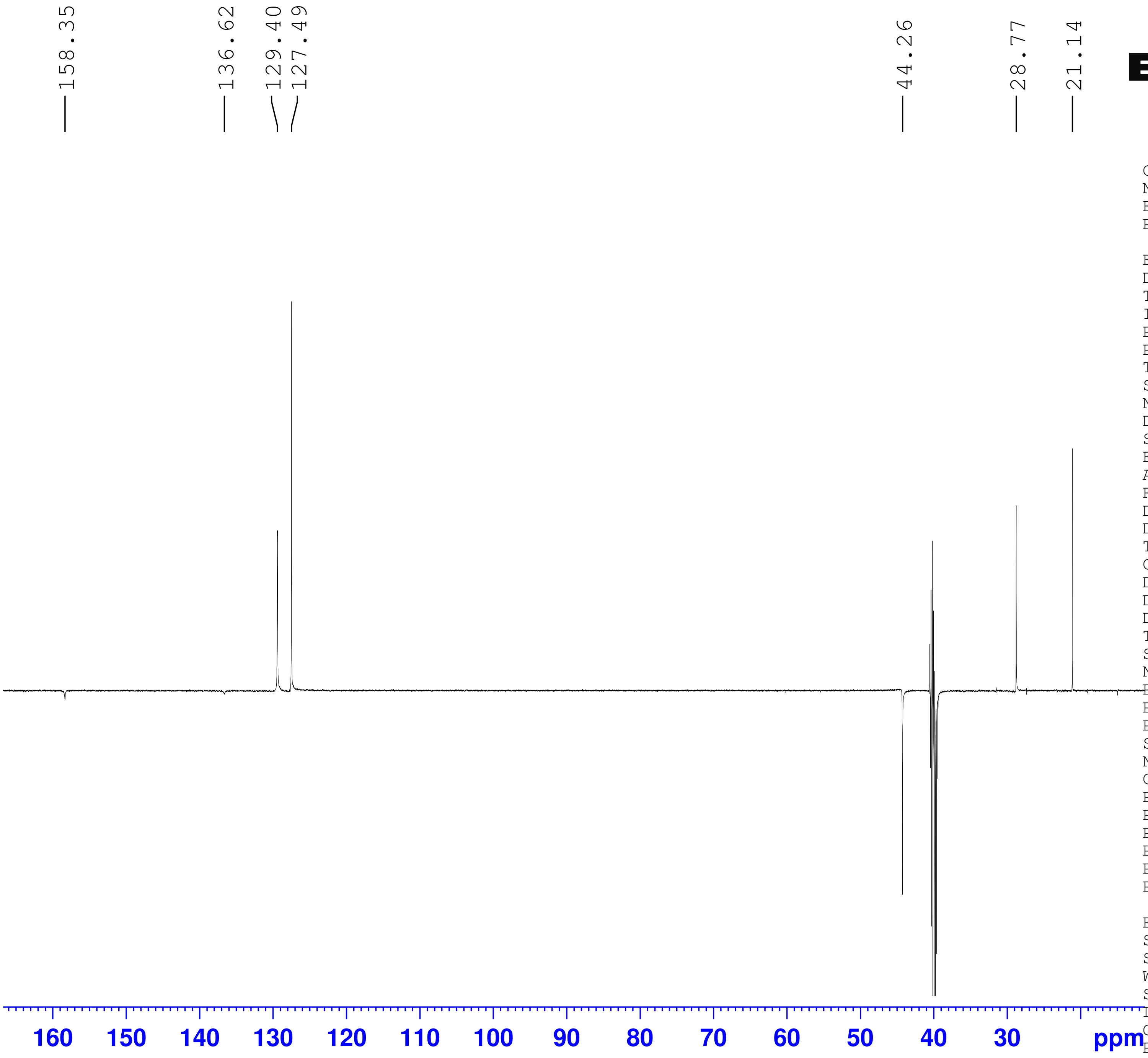

Current Data Parameters
NAME 33
EXPNO 2
PROCNO 1

F2 - Acquisition Parameters
Date_ 20181013
Time 12.17 h
INSTRUM spect
PROBHD Z119470_0187 (
PULPROG deptq135
TD 65536
SOLVENT DMSO
NS 20480
DS 8
SWH 29761.904 Hz
FIDRES 0.908261 Hz
AQ 1.1010048 sec
RG 192.72
DW 16.800 usec
DE 6.50 usec
TE 292.1 K
CNST2 145.0000000
D1 2.00000000 sec
D2 0.00344828 sec
D12 0.00002000 sec
TD0 1
SFO1 125.7829381 MHz
NUC1 13C
P1 10.00 usec
P2 20.00 usec
PLW1 82.09700012 W
SFO2 500.1820007 MHz
NUC2 1H
CPDPRG[2 waltz16
P0 15.00 usec
P3 10.00 usec
P4 20.00 usec
PCPD2 80.00 usec
PLW2 18.10400009 W
PLW12 0.28288001 W

F2 - Processing parameters
SI 32768
SF 125.7703610 MHz
WDW EM
SSB 0
LB 1.00 Hz
GB 0
PC 1.40

^{13}C (DEPTQ135) NMR (125 MHz, DMSO-d_6) of **33**

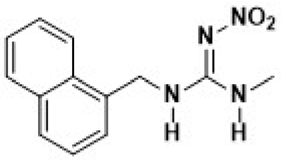

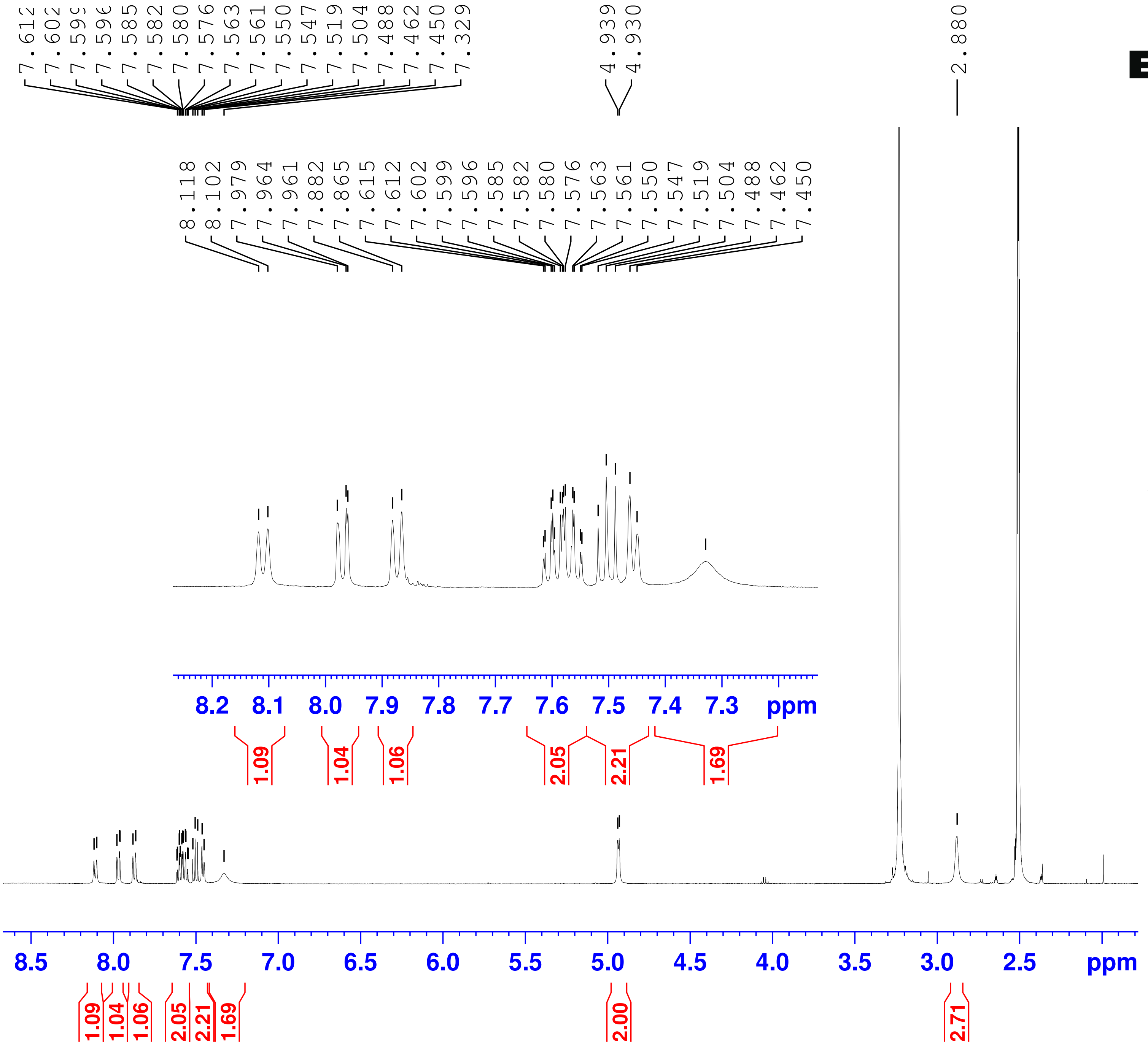


```
Current Data Parameters
NAME                    34
EXPNO                    2
PROCNO                   1

F2 - Acquisition Parameters
Date_             20180904
Time                 17.08 h
INSTRUM              spect
PROBHD    Z119470_0187 (
PULPROG               zg30
TD                   65536
SOLVENT               DMSO
NS                      16
DS                       2
SWH              10000.000 Hz
FIDRES            0.305176 Hz
AQ               3.2767999 sec
RG                  139.09
DW                  50.000 usec
DE                    6.50 usec
TE                   323.3 K
D1              1.00000000 sec
TD0                      1
SFO1           500.1830886 MHz
NUC1                    1H
P0                    3.33 usec
P1                   10.00 usec
PLW1           18.10400009 W

F2 - Processing parameters
SI                   65536
SF             500.1800000 MHz
WDW                     EM
SSB                      0
LB                    0.30 Hz
GB                       0
PC                    1.00
```


^{1}H NMR (500 MHz, DMSO-d_6, 50°C) of **34**

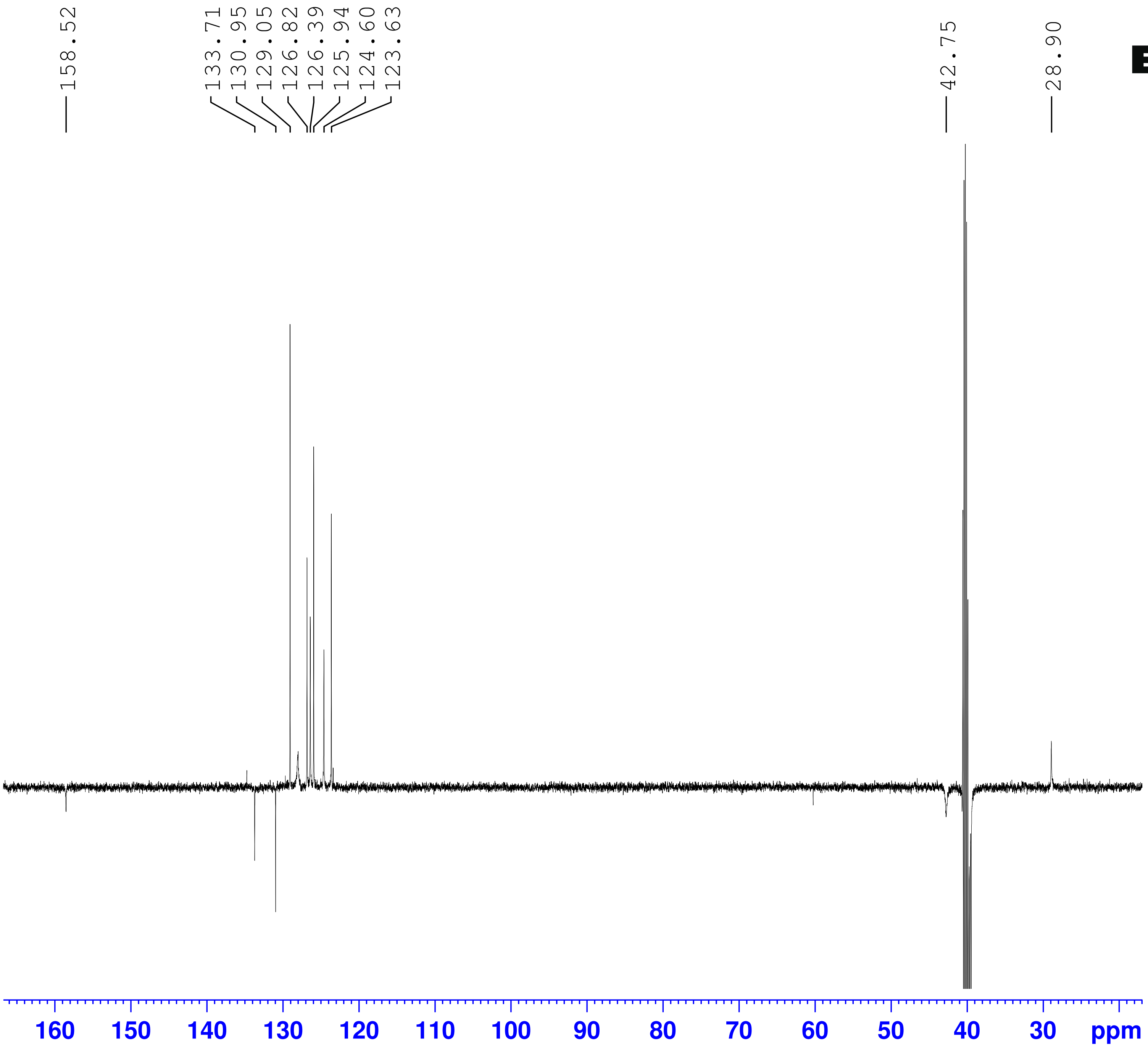

Current Data Parameters
NAME 34
EXPNO 3
PROCNO 1

F2 - Acquisition Parameters
Date_ 20180905
Time 9.23 h
INSTRUM spect
PROBHD Z119470_0187 (
PULPROG deptq135
TD 65536
SOLVENT DMSO
NS 18519
DS 8
SWH 29761.904 Hz
FIDRES 0.908261 Hz
AQ 1.1010048 sec
RG 192.72
DW 16.800 usec
DE 6.50 usec
TE 297.9 K
CNST2 145.0000000
D1 2.00000000 sec
D2 0.00344828 sec
D12 0.00002000 sec
TD0 1
SFO1 125.7829381 MHz
NUC1 13C
P1 10.00 usec
P2 20.00 usec
PLW1 82.09700012 W
SFO2 500.1820007 MHz
NUC2 1H
CPDPRG[2 waltz16
P0 15.00 usec
P3 10.00 usec
P4 20.00 usec
PCPD2 80.00 usec
PLW2 18.10400009 W
PLW12 0.28288001 W

F2 - Processing parameters
SI 32768
SF 125.7703610 MHz
WDW EM
SSB 0
LB 1.00 Hz
GB 0
PC 1.40

^{13}C (DEPTQ135) NMR (125 MHz, DMSO-d_6) of **34**

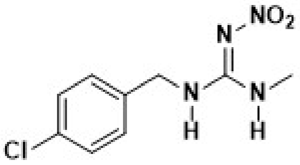

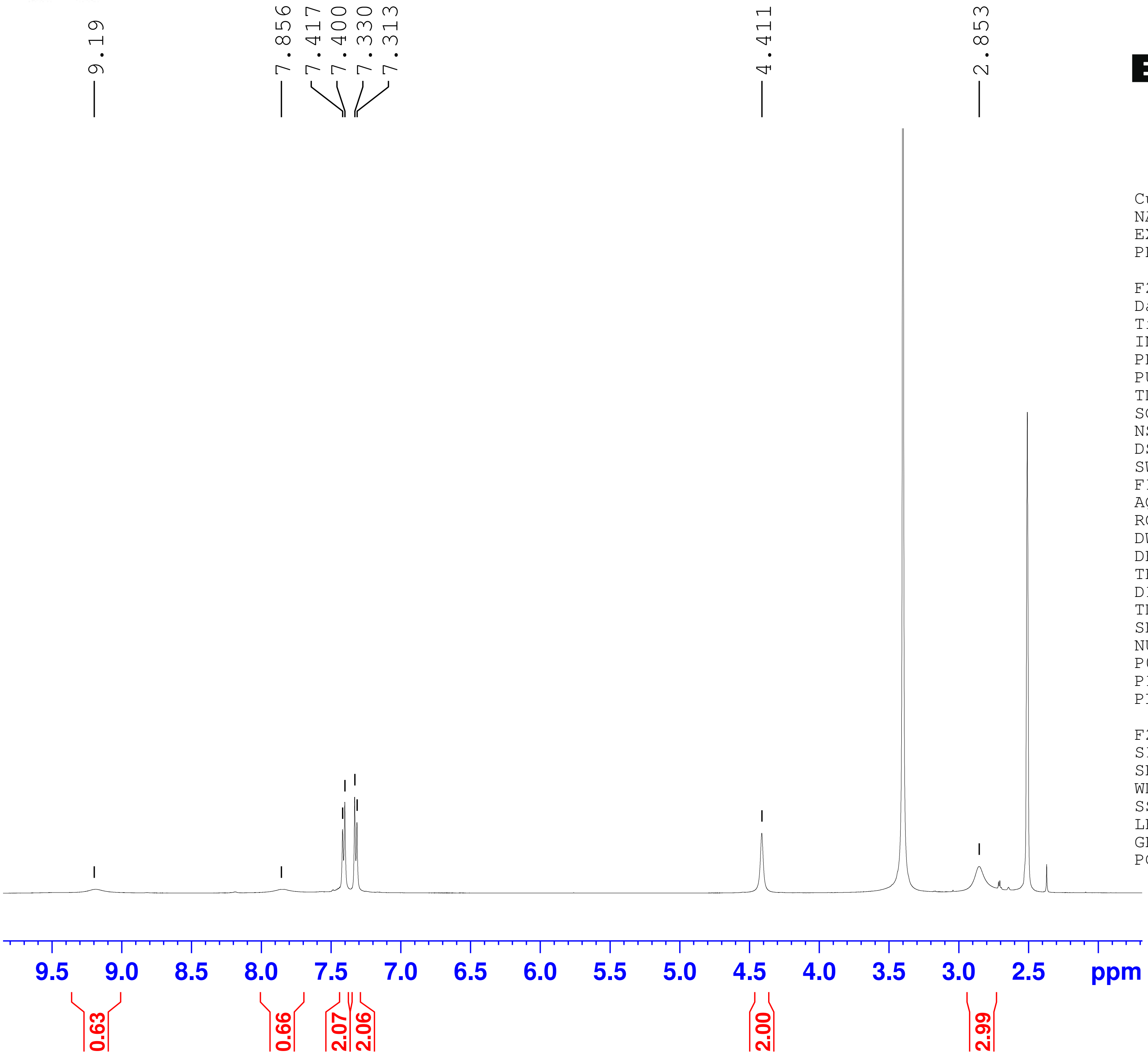


```
Current Data Parameters
NAME                 35
EXPNO                 1
PROCNO                1

F2 - Acquisition Parameters
Date_          20180831
Time              12.22 h
INSTRUM           spect
PROBHD   Z119470_0187 (
PULPROG            zg30
TD                65536
SOLVENT            DMSO
NS                   16
DS                    2
SWH           10000.000 Hz
FIDRES         0.305176 Hz
AQ            3.2767999 sec
RG                77.78
DW               50.000 usec
DE                 6.50 usec
TE                292.8 K
D1           1.00000000 sec
TD0                   1
SFO1        500.1830886 MHz
NUC1                 1H
P0                 3.33 usec
P1                10.00 usec
PLW1        18.10400009 W

F2 - Processing parameters
SI                65536
SF          500.1800000 MHz
WDW                  EM
SSB                   0
LB                 0.30 Hz
GB                    0
PC                 1.00
```

^{1}H NMR (500 MHz, DMSO-d_6) of **35**

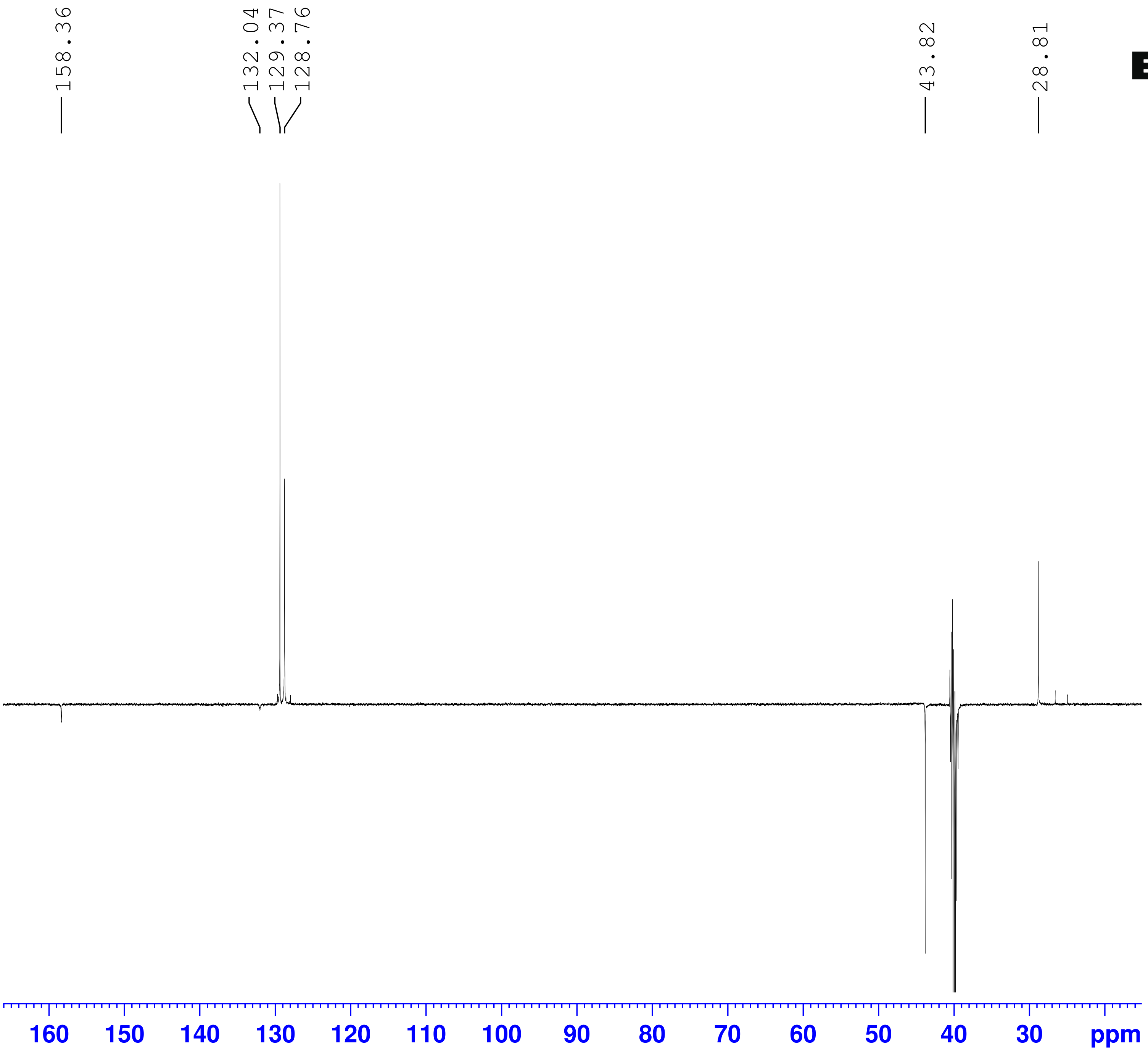

Current Data Parameters
NAME 35
EXPNO 2
PROCNO 1

F2 - Acquisition Parameters
Date_ 20180831
Time 18.49 h
INSTRUM spect
PROBHD Z119470_0187 (
PULPROG deptq135
TD 65536
SOLVENT DMSO
NS 6952
DS 8
SWH 29761.904 Hz
FIDRES 0.908261 Hz
AQ 1.1010048 sec
RG 192.72
DW 16.800 usec
DE 6.50 usec
TE 294.1 K
CNST2 145.0000000
D1 2.00000000 sec
D2 0.00344828 sec
D12 0.00002000 sec
TD0 1
SFO1 125.7829381 MHz
NUC1 13C
P1 10.00 usec
P2 20.00 usec
PLW1 82.09700012 W
SFO2 500.1820007 MHz
NUC2 1H
CPDPRG[2 waltz16
P0 15.00 usec
P3 10.00 usec
P4 20.00 usec
PCPD2 80.00 usec
PLW2 18.10400009 W
PLW12 0.28680280 W

F2 - Processing parameters
SI 32768
SF 125.7703610 MHz
WDW EM
SSB 0
LB 1.00 Hz
GB 0
PC 1.40

^{13}C (DEPTQ135) NMR (125 MHz, DMSO-d_6) of **35**

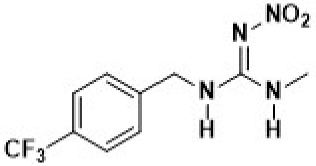

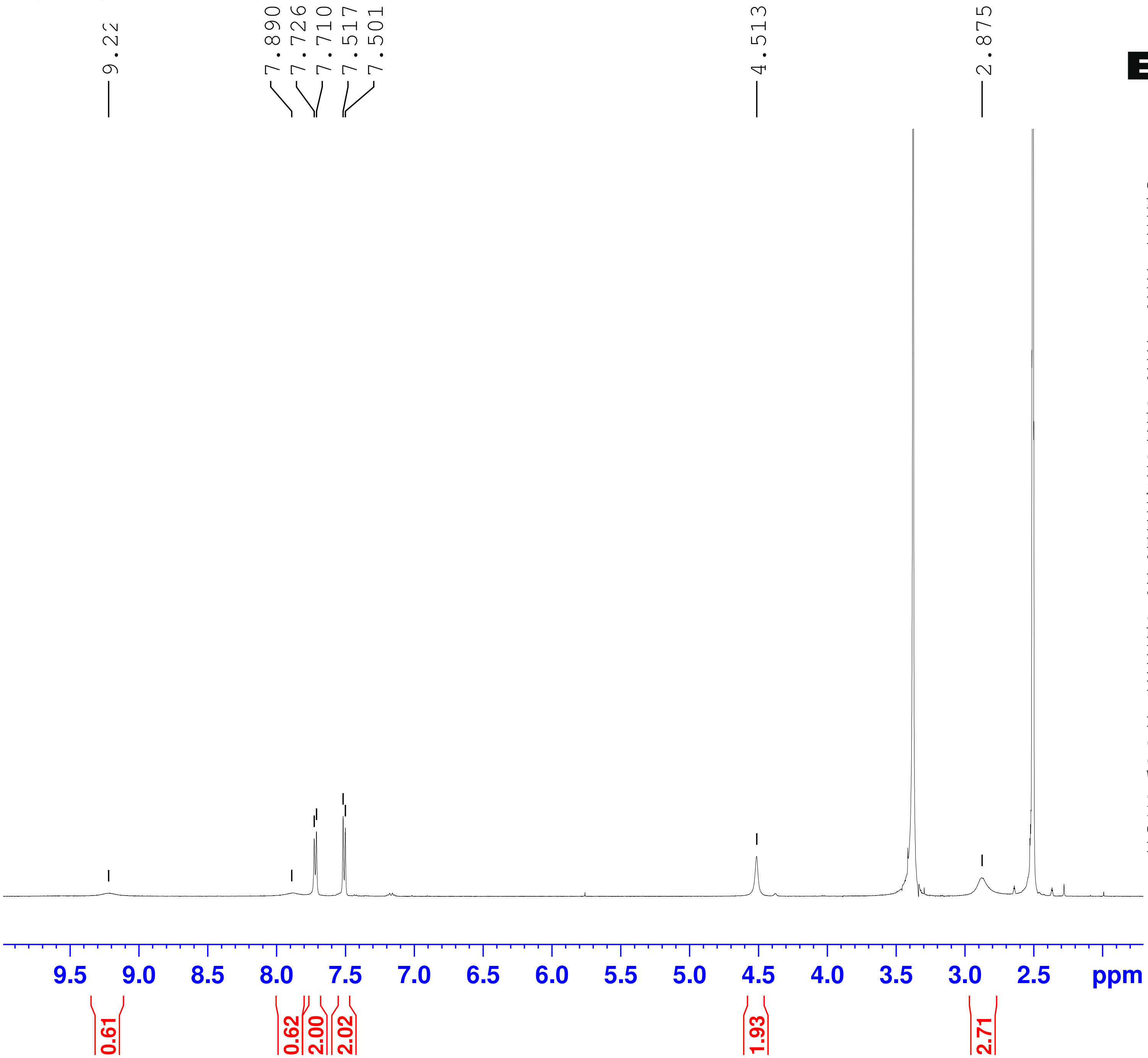

Current Data Parameters

NAME	36
EXPNO	2
PROCNO	1

F2 - Acquisition Parameters

Date_	20180928
Time	12.49 h
INSTRUM	spect
PROBHD	Z119470_0187 (
PULPROG	zg30
TD	65536
SOLVENT	DMSO
NS	5
DS	1
SWH	10000.000 Hz
FIDRES	0.305176 Hz
AQ	3.2767999 sec
RG	108.3
DW	50.000 usec
DE	6.50 usec
TE	293.1 K
D1	1.00000000 sec
TD0	1
SFO1	500.1830886 MHz
NUC1	1H
P0	3.33 usec
P1	10.00 usec
PLW1	18.10400009 W

F2 - Processing parameters

SI	65536
SF	500.1800000 MHz
WDW	EM
SSB	0
LB	0.30 Hz
GB	0
PC	1.00

^{1}H NMR (500 MHz, DMSO-d_6) of **36**

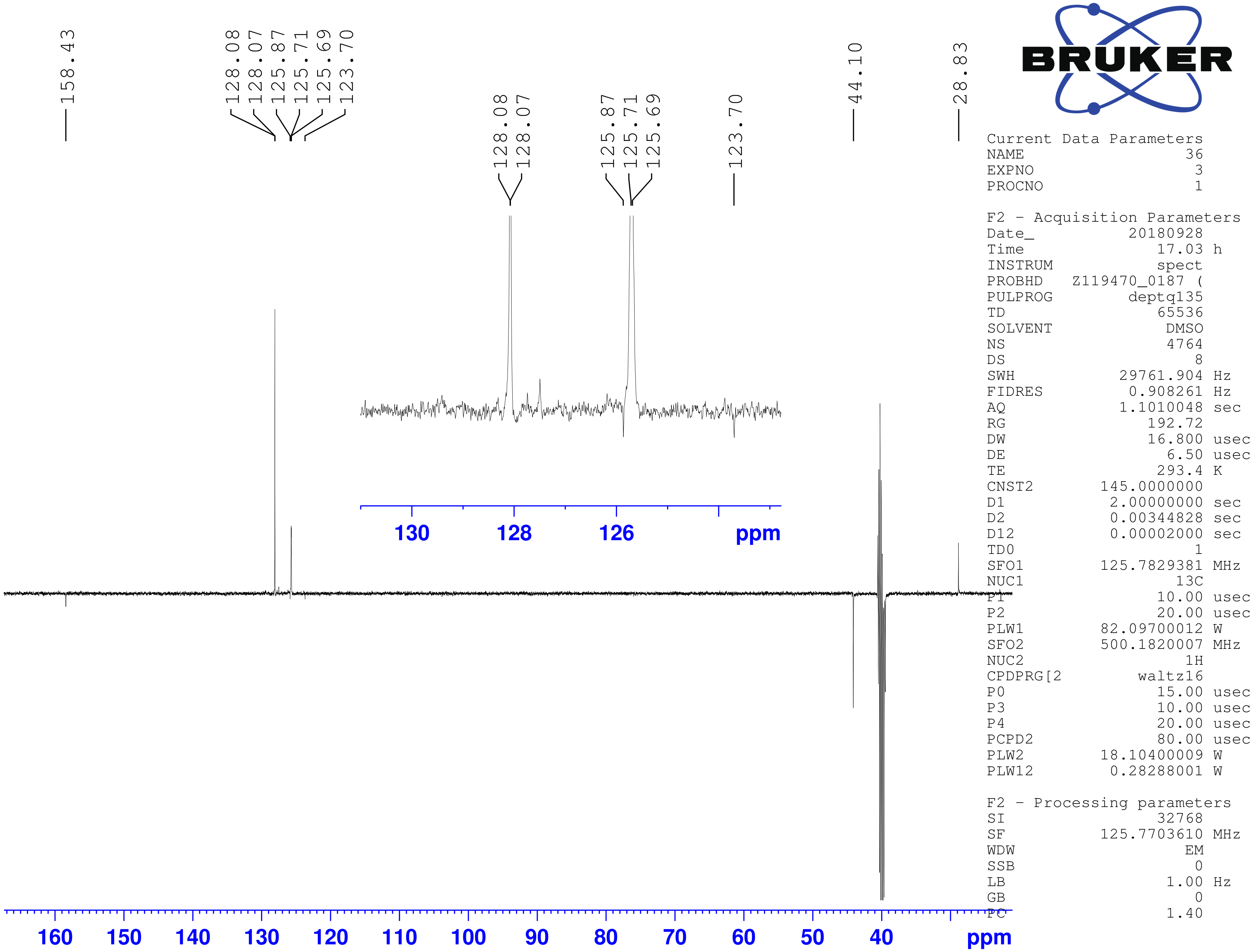

^{13}C (DEPTQ135) NMR (500 MHz, DMSO-d_6) of **36**

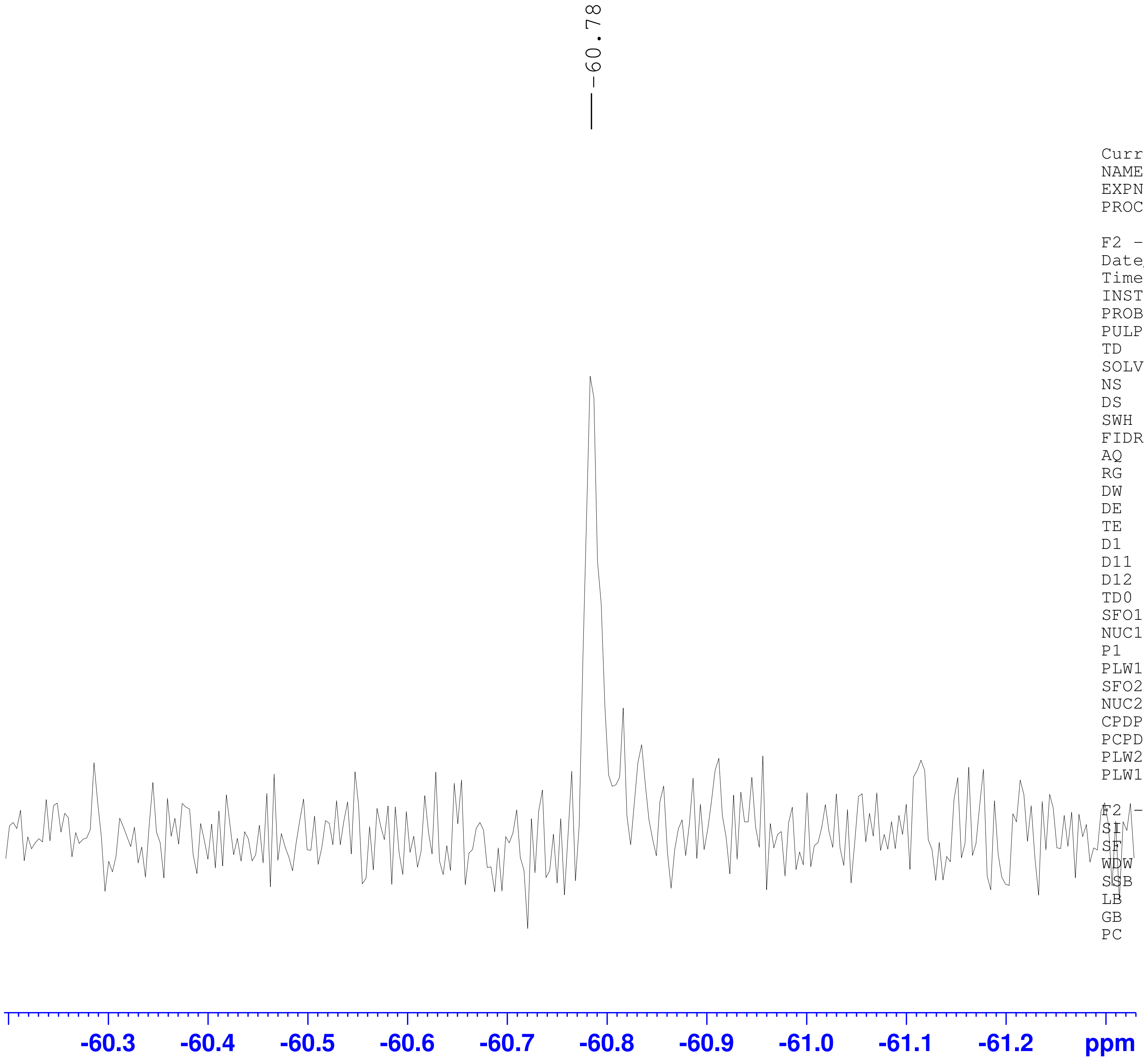

BRUKER

```
Current Data Parameters
NAME                ARH-14
EXPNO                    1
PROCNO                   1

F2 - Acquisition Parameters
Date_             20190801
Time                 14.40 h
INSTRUM              spect
PROBHD    Z119470_0187 (
PULPROG         zgfhigqn.2
TD                  131072
SOLVENT               DMSO
NS                      16
DS                       4
SWH             113636.367 Hz
FIDRES            1.733953 Hz
AQ               0.5767168 sec
RG                   11.27
DW                   4.400 usec
DE                    6.50 usec
TE                   293.4 K
D1              1.00000000 sec
D11             0.03000000 sec
D12             0.00002000 sec
TD0                      1
SFO1           470.5923603 MHz
NUC1                   19F
P1                   16.00 usec
PLW1           36.93899918 W
SFO2           500.1820007 MHz
NUC2                    1H
CPDPRG[2           waltz16
PCPD2                80.00 usec
PLW2           18.10400009 W
PLW12           0.31009001 W

F2 - Processing parameters
SI                   65536
SF             470.6394242 MHz
WDW                     EM
SSB                      0
LB                    0.30 Hz
GB                       0
PC                    1.00
```

^{19}F NMR (470 MHz, DMSO-d_6) of **36**

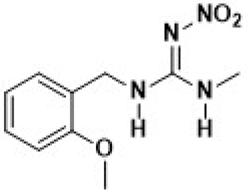

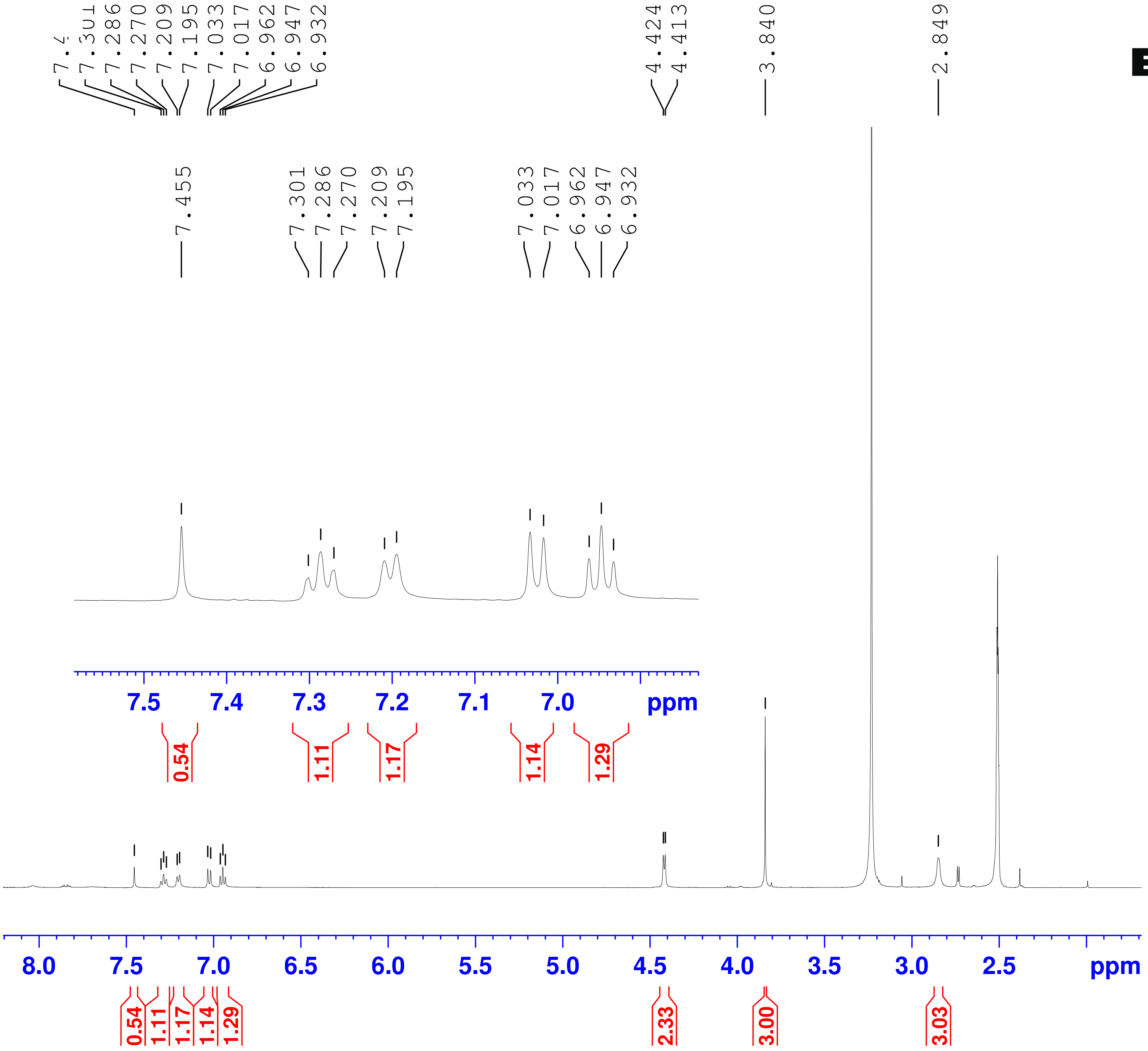

Current Data Parameters

NAME	37
EXPNO	2
PROCNO	1

F2 - Acquisition Parameters

Date_	20180829
Time	18.09 h
INSTRUM	spect
PROBHD	Z119470_0187 (
PULPROG	zg30
TD	65536
SOLVENT	DMSO
NS	16
DS	2
SWH	10000.000 Hz
FIDRES	0.305176 Hz
AQ	3.2767999 sec
RG	139.09
DW	50.000 usec
DE	6.50 usec
TE	323.0 K
D1	1.00000000 sec
TD0	1
SFO1	500.1830886 MHz
NUC1	1H
P0	3.33 usec
P1	10.00 usec
PLW1	18.10400009 W

F2 - Processing parameters

SI	65536
SF	500.1800000 MHz
WDW	EM
SSB	0
LB	0.30 Hz
GB	0
PC	1.00

^{1}H NMR (500 MHz, DMSO-d_6, 50°C) of **37**

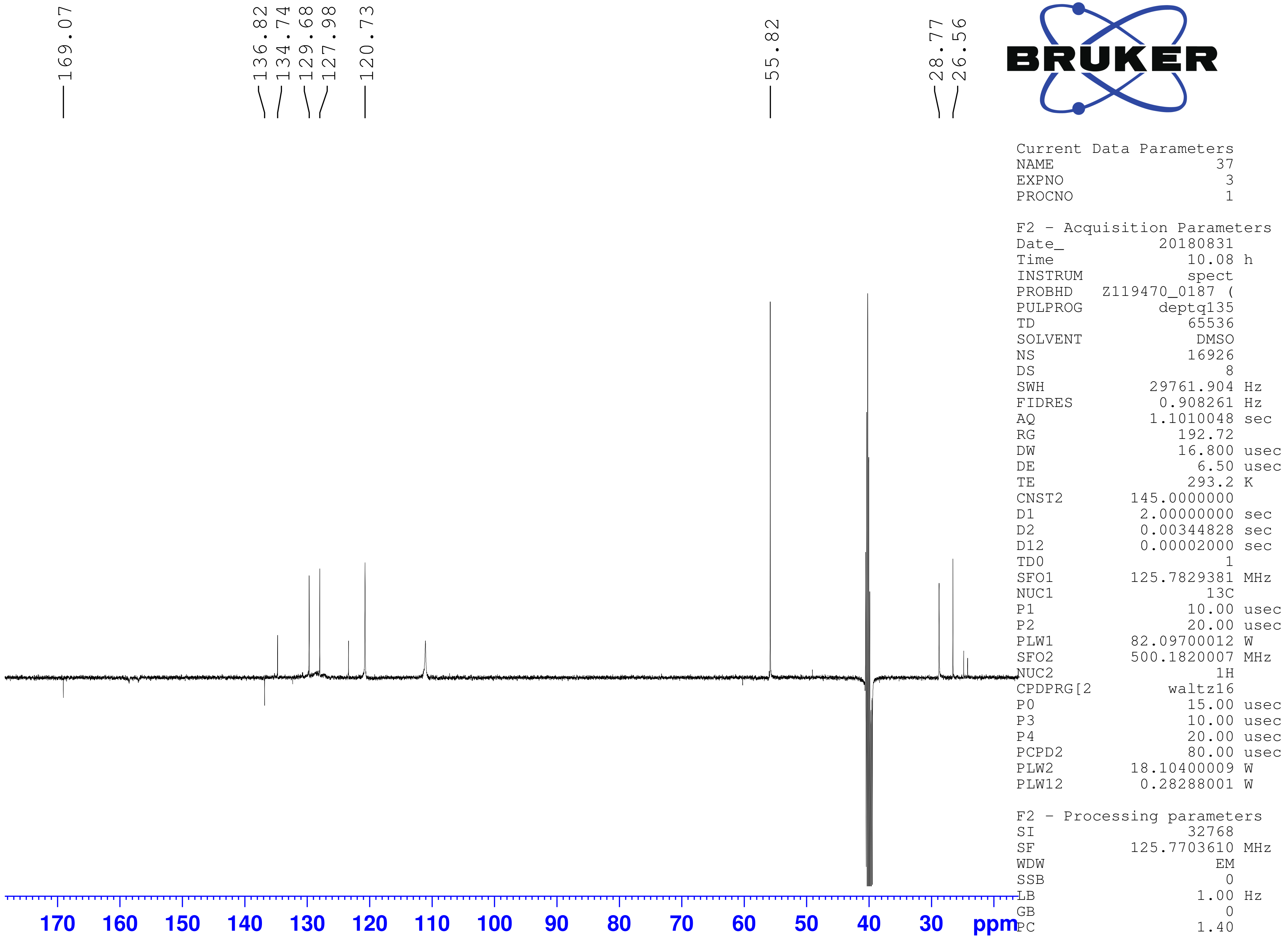

^{13}C (DEPTQ135) NMR (125 MHz, DMSO-d_6, 50°C) of **37**

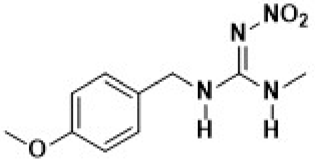

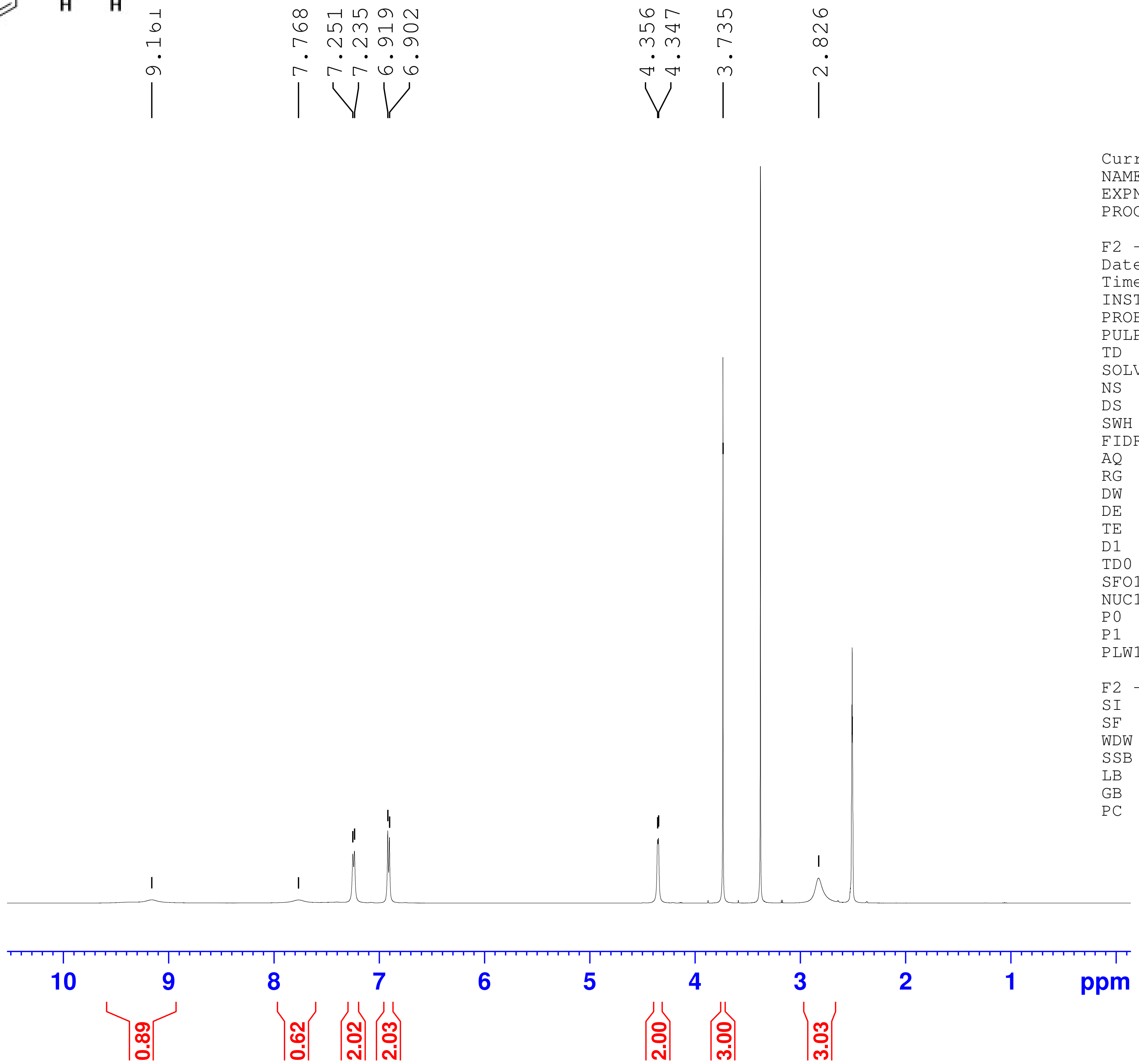

Current Data Parameters

NAME	38
EXPNO	1
PROCNO	1

F2 - Acquisition Parameters

Date_	20181205
Time	10.53 h
INSTRUM	spect
PROBHD	Z119470_0187 (
PULPROG	zg30
TD	65536
SOLVENT	DMSO
NS	16
DS	2
SWH	10000.000 Hz
FIDRES	0.305176 Hz
AQ	3.2767999 sec
RG	70.58
DW	50.000 usec
DE	6.50 usec
TE	291.1 K
D1	1.00000000 sec
TD0	1
SFO1	500.1830886 MHz
NUC1	1H
P0	3.33 usec
P1	10.00 usec
PLW1	18.10400009 W

F2 - Processing parameters

SI	65536
SF	500.1800000 MHz
WDW	EM
SSB	0
LB	0.30 Hz
GB	0
PC	1.00

^{1}H NMR (500 MHz, DMSO-d_6, 50°C of **38**

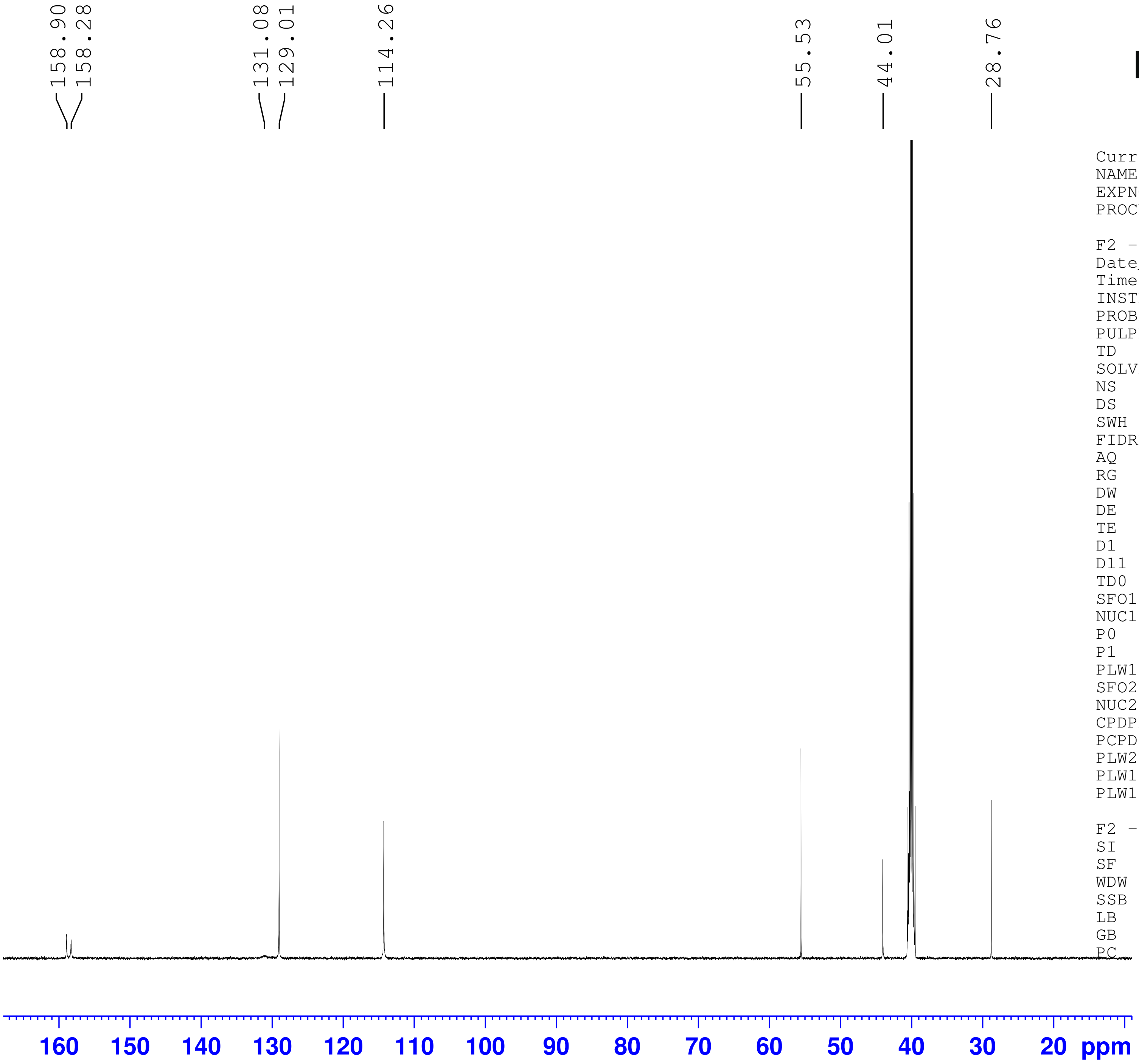

Current Data Parameters
NAME 38
EXPNO 3
PROCNO 1

F2 - Acquisition Parameters
Date_ 20181205
Time 12.54 h
INSTRUM spect
PROBHD Z119470_0187 (
PULPROG zgpg30
TD 65536
SOLVENT DMSO
NS 1973
DS 8
SWH 29761.904 Hz
FIDRES 0.908261 Hz
AQ 1.1010048 sec
RG 192.72
DW 16.800 usec
DE 6.50 usec
TE 291.6 K
D1 2.00000000 sec
D11 0.03000000 sec
TD0 1
SFO1 125.7829381 MHz
NUC1 13C
P0 3.33 usec
P1 10.00 usec
PLW1 82.09700012 W
SFO2 500.1820007 MHz
NUC2 1H
CPDPRG[2 waltz65
PCPD2 80.00 usec
PLW2 18.10400009 W
PLW12 0.28288001 W
PLW13 0.14228000 W

F2 - Processing parameters
SI 32768
SF 125.7703610 MHz
WDW EM
SSB 0
LB 1.00 Hz
GB 0
PC 1.40

^{13}C NMR (125 MHz, DMSO-d_6) of **38**

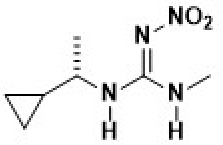

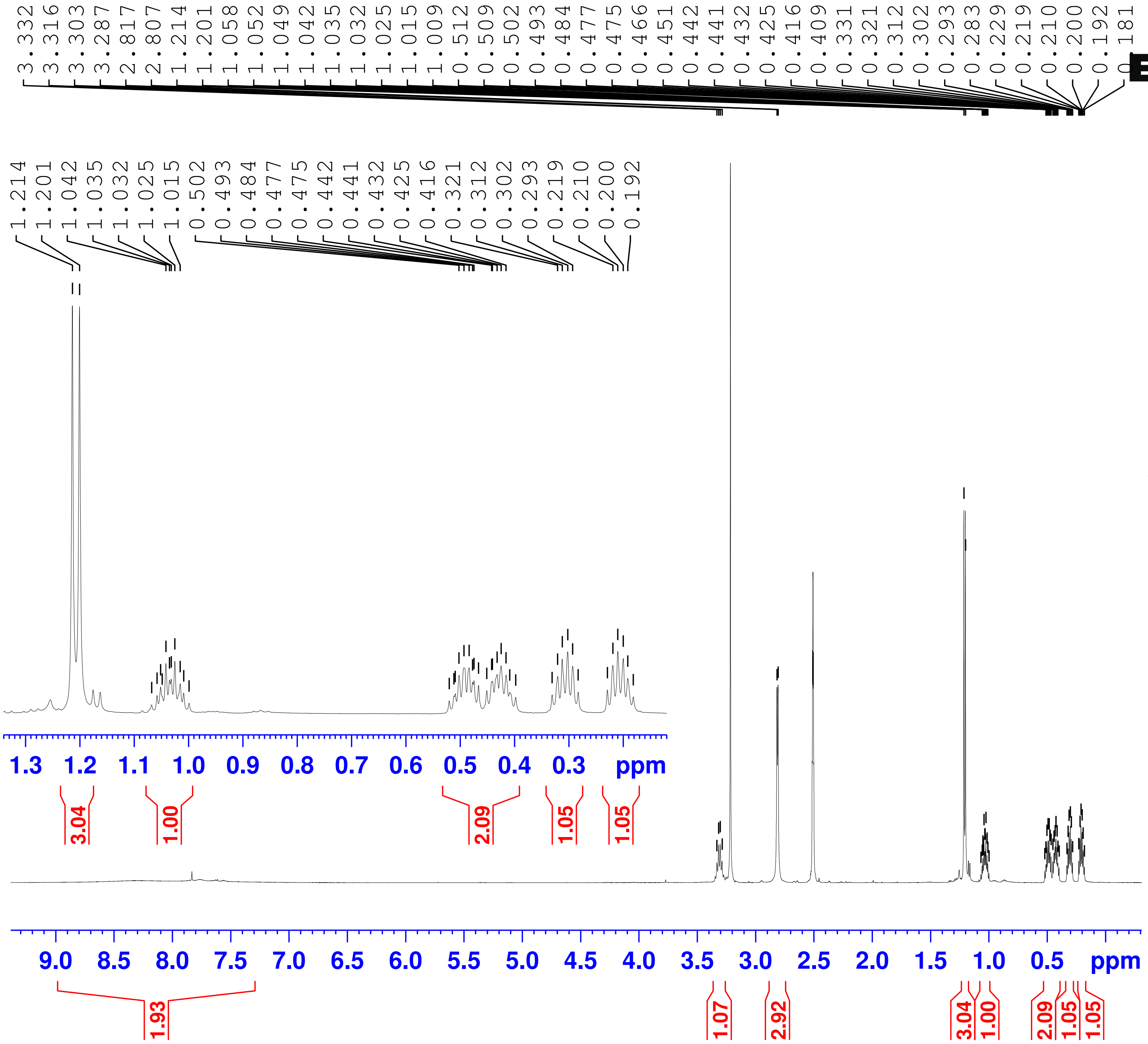


```
Current Data Parameters
NAME                S-39
EXPNO                  2
PROCNO                 1

F2 - Acquisition Parameters
Date_           20180905
Time             17.57 h
INSTRUM            spect
PROBHD   Z119470_0187 (
PULPROG             zg30
TD                 65536
SOLVENT             DMSO
NS                     5
DS                     1
SWH           10000.000 Hz
FIDRES         0.305176 Hz
AQ            3.2767999 sec
RG                 77.78
DW               50.000 usec
DE                 6.50 usec
TE                323.2 K
D1           1.00000000 sec
TD0                    1
SFO1        500.1830886 MHz
NUC1                  1H
P0                 3.33 usec
P1                10.00 usec
PLW1        18.10400009 W

F2 - Processing parameters
SI                 65536
SF          500.1800000 MHz
WDW                   EM
SSB                    0
LB                 0.30 Hz
GB                     0
PC                  1.00
```

^{1}H NMR (500 MHz, DMSO-d_6, 50°C) of ***S*-39**

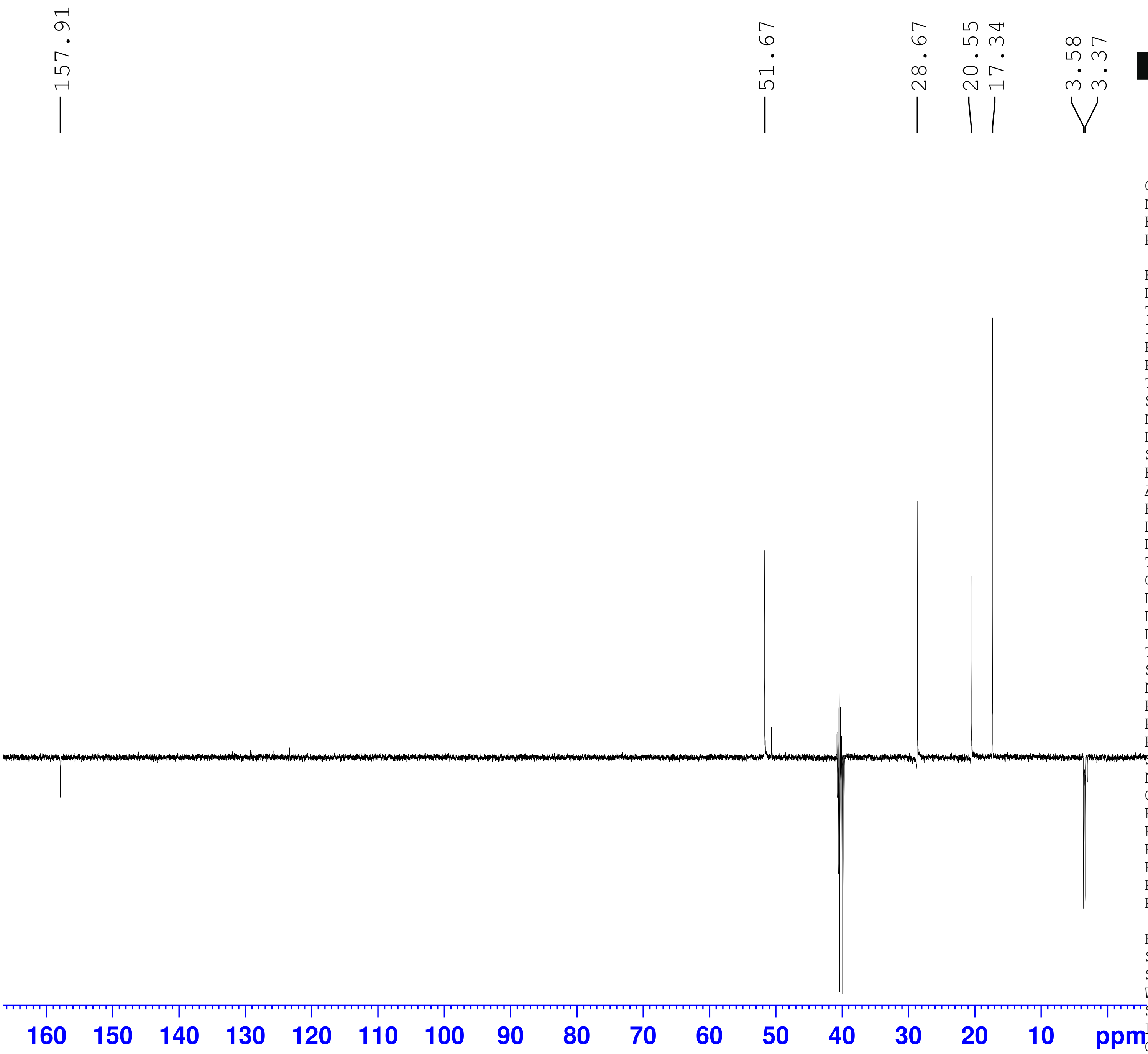

Current Data Parameters
NAME S-39
EXPNO 4
PROCNO 1

F2 - Acquisition Parameters
Date_ 20180906
Time 11.34 h
INSTRUM spect
PROBHD Z119470_0187 (
PULPROG deptq135
TD 65536
SOLVENT DMSO
NS 1087
DS 8
SWH 29761.904 Hz
FIDRES 0.908261 Hz
AQ 1.1010048 sec
RG 192.72
DW 16.800 usec
DE 6.50 usec
TE 323.3 K
CNST2 145.0000000
D1 2.00000000 sec
D2 0.00344828 sec
D12 0.00002000 sec
TD0 1
SFO1 125.7829381 MHz
NUC1 13C
P1 10.00 usec
P2 20.00 usec
PLW1 82.09700012 W
SFO2 500.1820007 MHz
NUC2 1H
CPDPRG[2 waltz16
P0 15.00 usec
P3 10.00 usec
P4 20.00 usec
PCPD2 80.00 usec
PLW2 18.10400009 W
PLW12 0.28288001 W

F2 - Processing parameters
SI 32768
SF 125.7703610 MHz
WDW EM
SSB 0
LB 1.00 Hz
GB 0
PC 1.40

^{13}C (DEPTQ135) NMR (125 MHz, DMSO-d_6, 50°C) of ***S*-39**

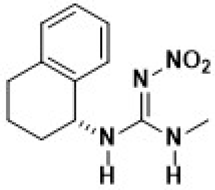

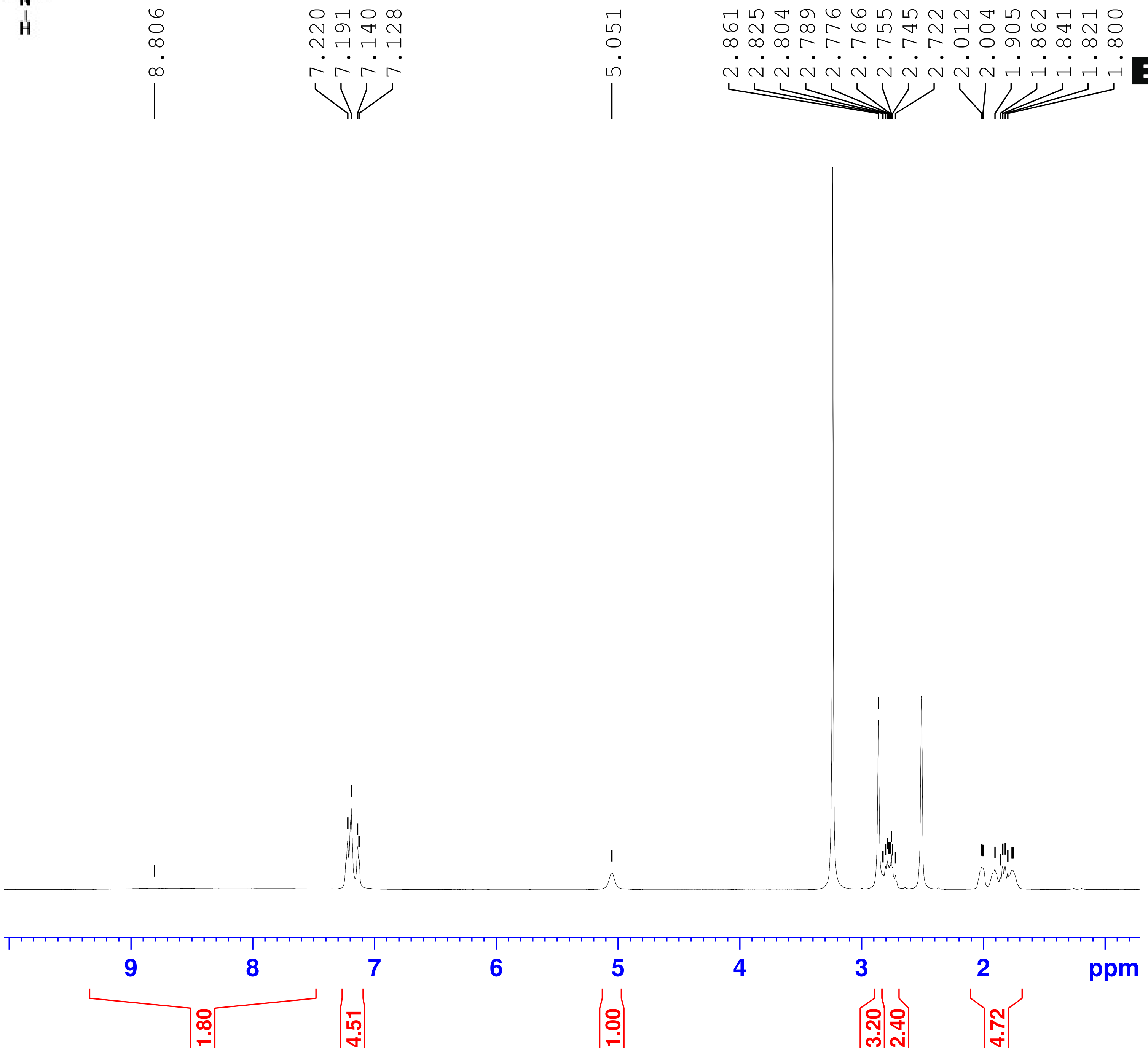

Current Data Parameters
NAME S-40
EXPNO 2
PROCNO 1

F2 - Acquisition Parameters
Date_ 20180920
Time 10.42 h
INSTRUM spect
PROBHD Z119470_0187 (
PULPROG zg30
TD 65536
SOLVENT DMSO
NS 16
DS 2
SWH 10000.000 Hz
FIDRES 0.305176 Hz
AQ 3.2767999 sec
RG 77.78
DW 50.000 usec
DE 6.50 usec
TE 323.0 K
D1 1.00000000 sec
TD0 1
SFO1 500.1830886 MHz
NUC1 1H
P0 3.33 usec
P1 10.00 usec
PLW1 18.10400009 W

F2 - Processing parameters
SI 65536
SF 500.1800000 MHz
WDW EM
SSB 0
LB 0.30 Hz
GB 0
PC 1.00

^{1}H NMR (500 MHz, DMSO-d$_6$, 50°C) of ***S*-40**

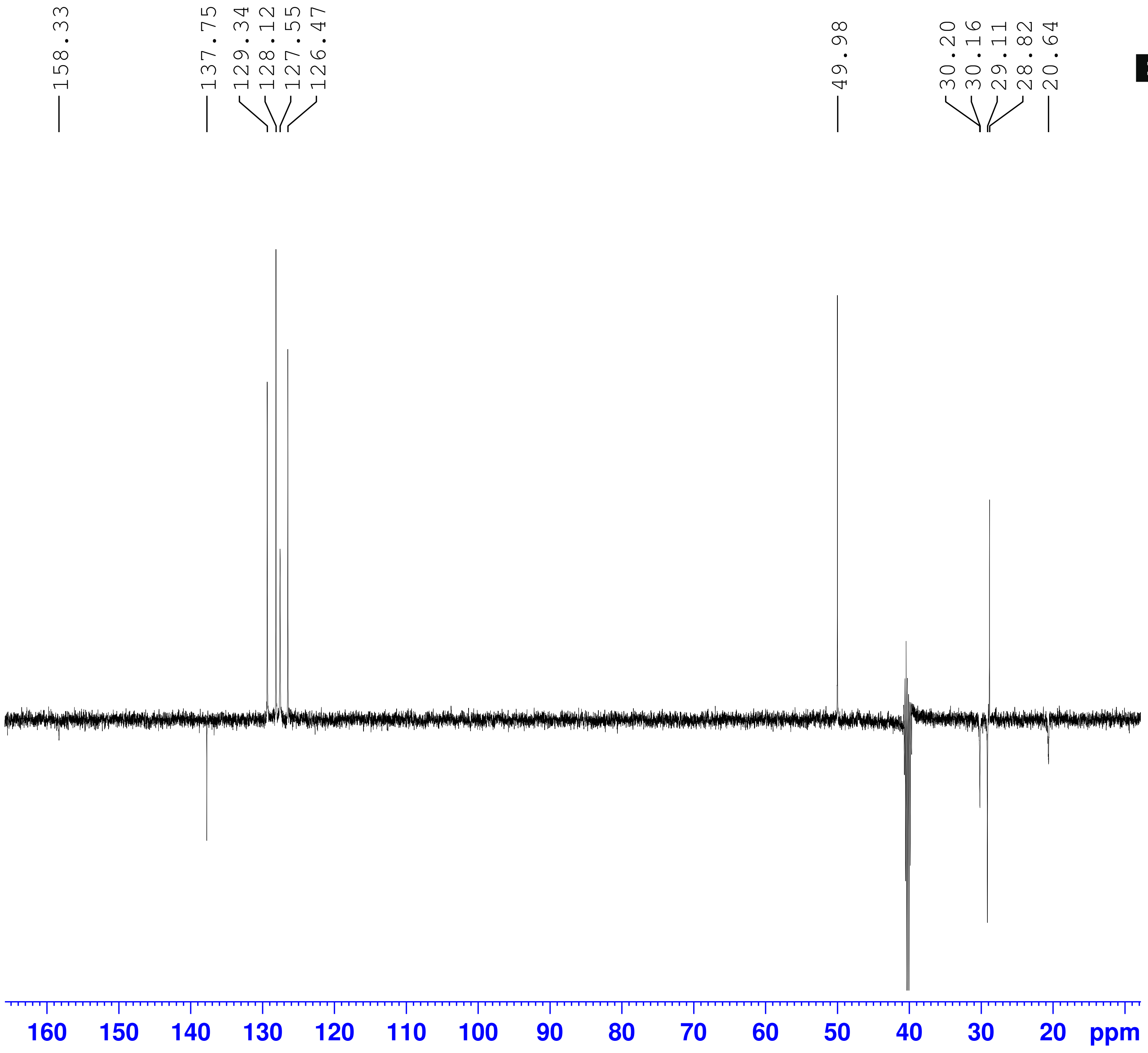

Current Data Parameters
NAME S-40
EXPNO 3
PROCNO 1

F2 - Acquisition Parameters

Date_	20180920	
Time	10.59	h
INSTRUM	spect	
PROBHD	Z119470_0187 (	
PULPROG	deptq135	
TD	65536	
SOLVENT	DMSO	
NS	256	
DS	8	
SWH	29761.904	Hz
FIDRES	0.908261	Hz
AQ	1.1010048	sec
RG	192.72	
DW	16.800	usec
DE	6.50	usec
TE	323.2	K
CNST2	145.0000000	
D1	2.00000000	sec
D2	0.00344828	sec
D12	0.00002000	sec
TD0	1	
SFO1	125.7829381	MHz
NUC1	13C	
P1	10.00	usec
P2	20.00	usec
PLW1	82.09700012	W
SFO2	500.1820007	MHz
NUC2	1H	
CPDPRG[2	waltz16	
P0	15.00	usec
P3	10.00	usec
P4	20.00	usec
PCPD2	80.00	usec
PLW2	18.10400009	W
PLW12	0.28288001	W

F2 - Processing parameters

SI	32768	
SF	125.7703610	MHz
WDW	EM	
SSB	0	
LB	1.00	Hz
GB	0	
PC	1.40	

^{13}C (DEPTQ135) NMR (125 MHz, DMSO-d_6, 50°C) of ***S-40***

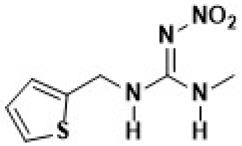

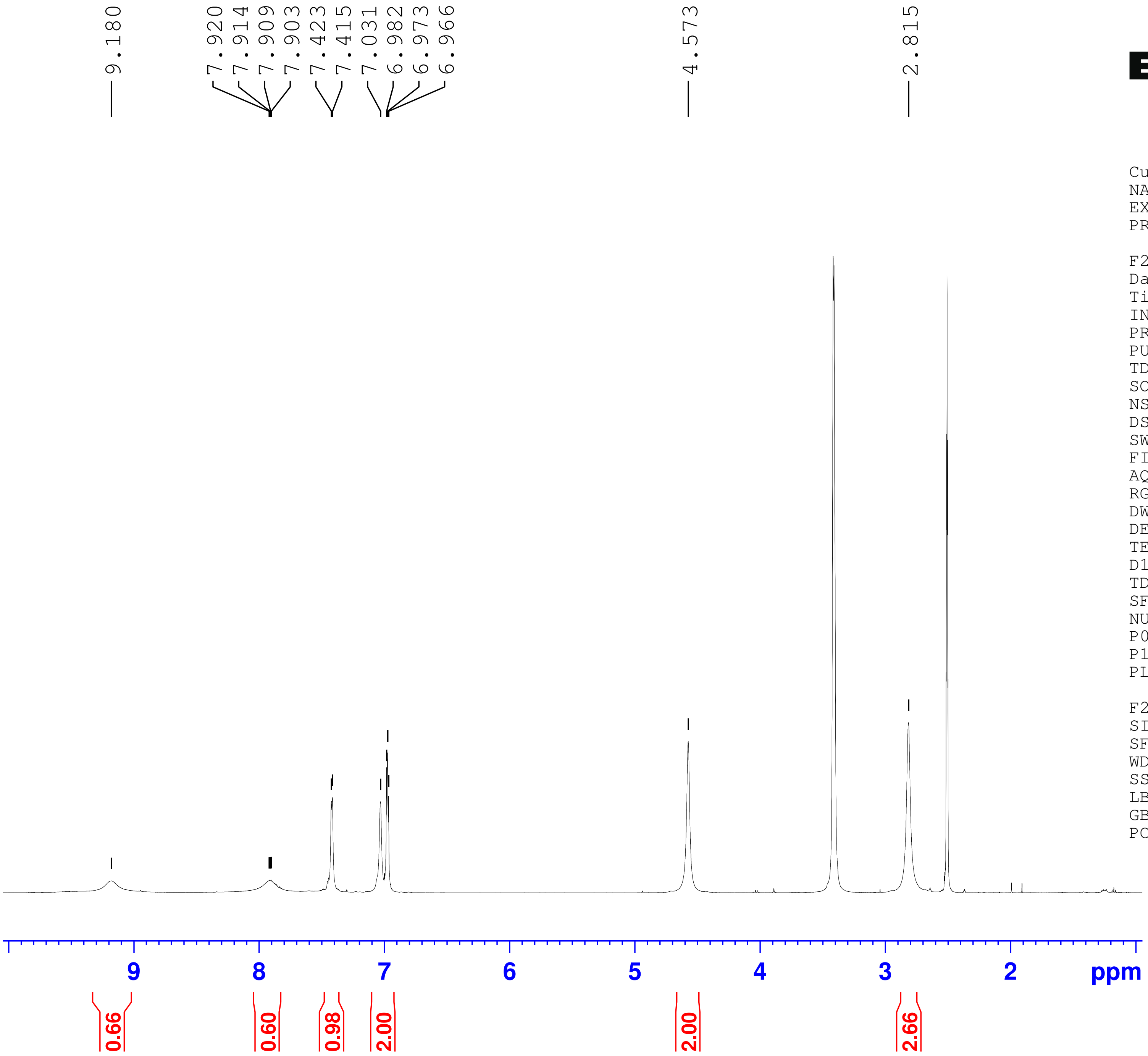


```
Current Data Parameters
NAME                41
EXPNO                1
PROCNO               1

F2 - Acquisition Parameters
Date_          20181008
Time              18.53 h
INSTRUM           spect
PROBHD   Z119470_0187 (
PULPROG            zg30
TD                65536
SOLVENT            DMSO
NS                    5
DS                    2
SWH           10000.000 Hz
FIDRES         0.305176 Hz
AQ            3.2767999 sec
RG                62.71
DW               50.000 usec
DE                 6.50 usec
TE                291.7 K
D1           1.00000000 sec
TD0                   1
SFO1        500.1830886 MHz
NUC1                 1H
P0                 3.33 usec
P1                10.00 usec
PLW1        18.10400009 W

F2 - Processing parameters
SI                65536
SF          500.1800000 MHz
WDW                  EM
SSB                   0
LB                 0.30 Hz
GB                    0
PC                 1.00
```

^{1}H NMR (500 MHz, DMSO-d_6) of **41**

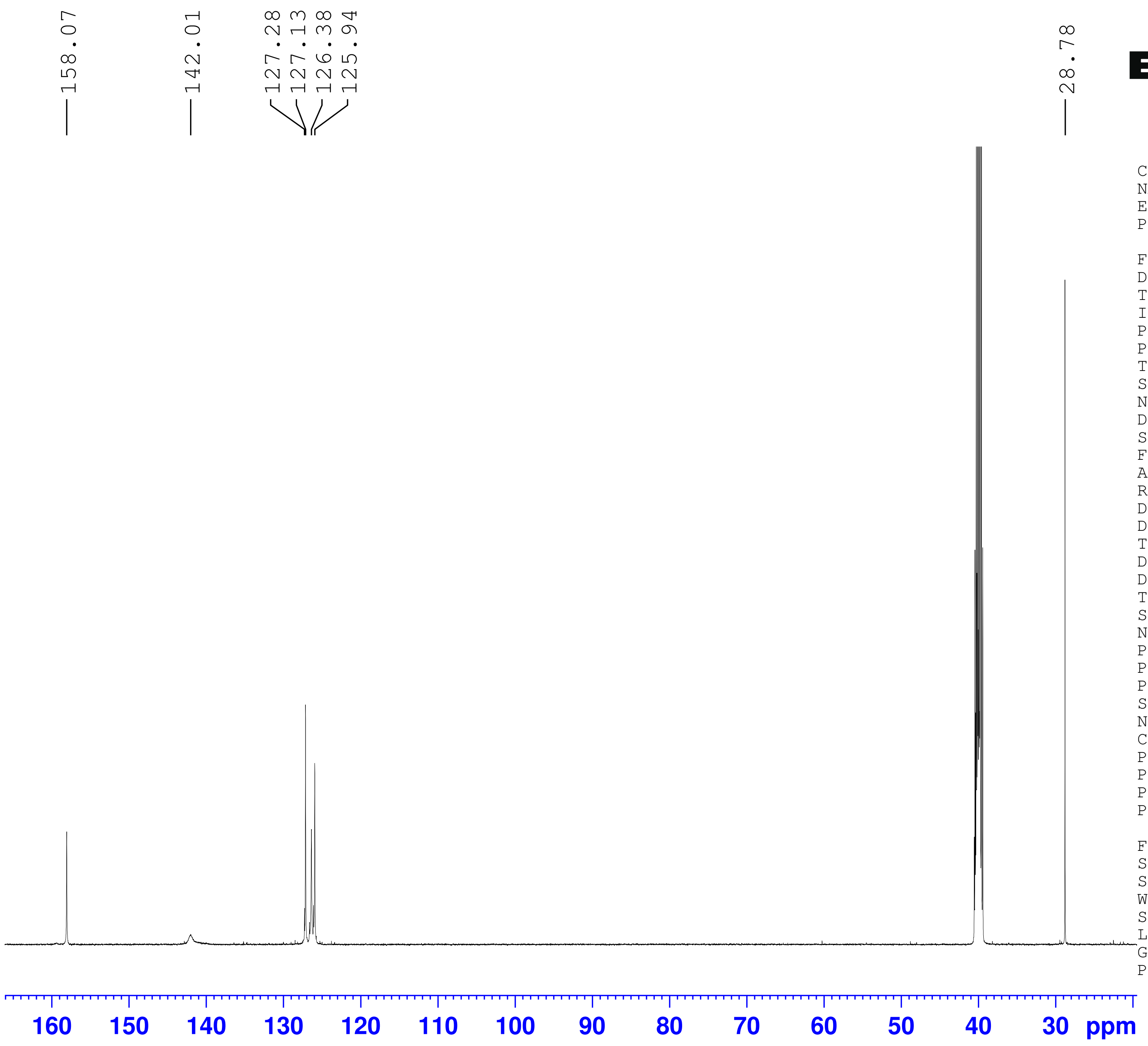

Current Data Parameters
NAME 41
EXPNO 3
PROCNO 1

F2 - Acquisition Parameters
Date_ 20181016
Time 21.44 h
INSTRUM spect
PROBHD Z119470_0187 (
PULPROG zgpg30
TD 65536
SOLVENT DMSO
NS 30720
DS 8
SWH 29761.904 Hz
FIDRES 0.908261 Hz
AQ 1.1010048 sec
RG 192.72
DW 16.800 usec
DE 6.50 usec
TE 292.7 K
D1 2.00000000 sec
D11 0.03000000 sec
TD0 1
SFO1 125.7829381 MHz
NUC1 13C
P0 3.33 usec
P1 10.00 usec
PLW1 82.09700012 W
SFO2 500.1820007 MHz
NUC2 1H
CPDPRG[2 waltz65
PCPD2 80.00 usec
PLW2 18.10400009 W
PLW12 0.28288001 W
PLW13 0.14228000 W

F2 - Processing parameters
SI 32768
SF 125.7703610 MHz
WDW EM
SSB 0
LB 1.00 Hz
GB 0
PC 1.40

^{13}C NMR (125 MHz, DMSO-d_6) of **41**

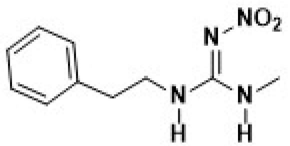

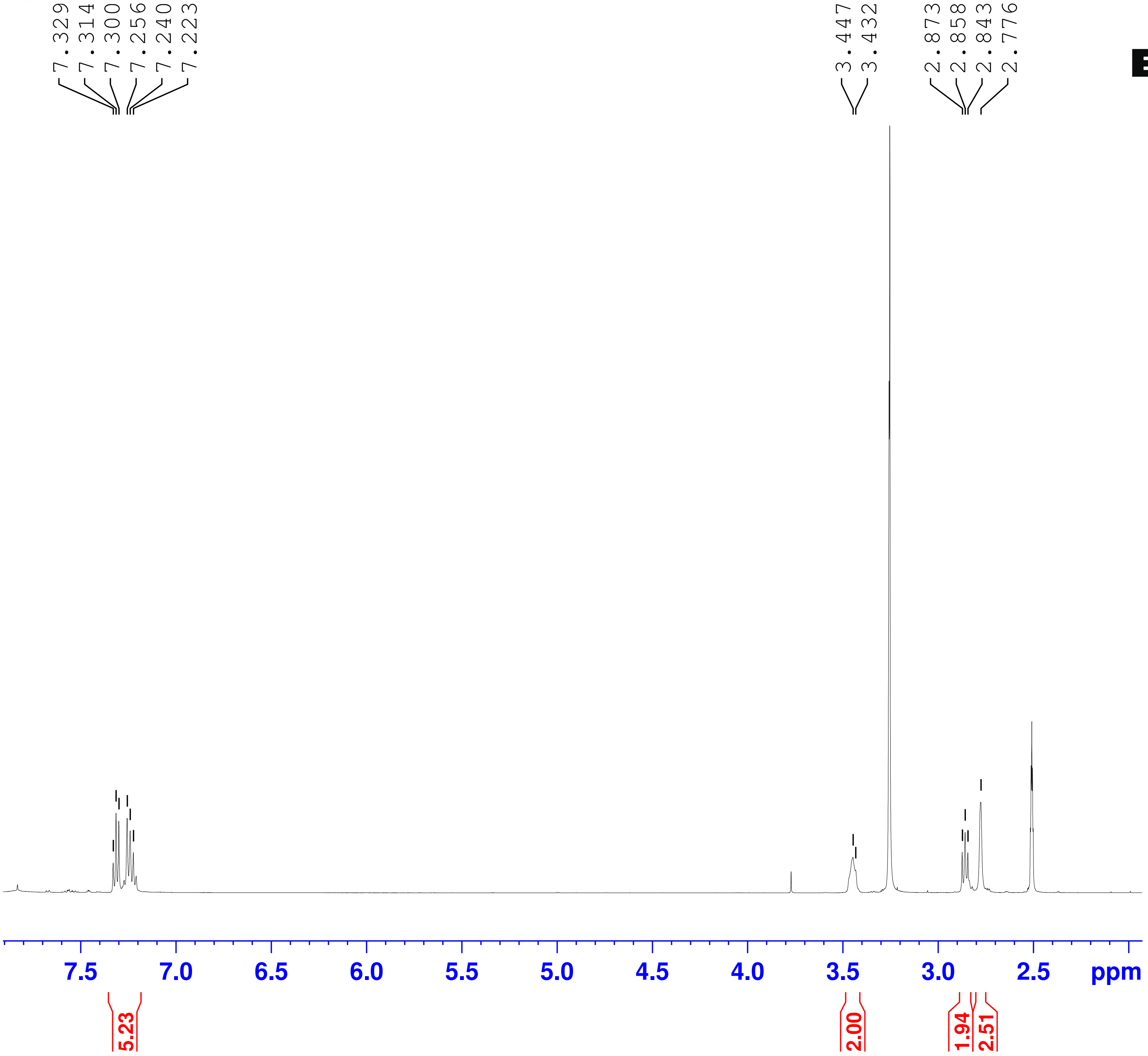


```
Current Data Parameters
NAME                    42
EXPNO                    1
PROCNO                   1

F2 - Acquisition Parameters
Date_             20180920
Time                 11.05 h
INSTRUM              spect
PROBHD    Z119470_0187 (
PULPROG               zg30
TD                   65536
SOLVENT               DMSO
NS                       5
DS                       1
SWH              10000.000 Hz
FIDRES            0.305176 Hz
AQ               3.2767999 sec
RG                   77.78
DW                  50.000 usec
DE                    6.50 usec
TE                   323.0 K
D1              1.00000000 sec
TD0                      1
SFO1           500.1830886 MHz
NUC1                    1H
P0                    3.33 usec
P1                   10.00 usec
PLW1           18.10400009 W

F2 - Processing parameters
SI                   65536
SF             500.1800000 MHz
WDW                     EM
SSB                      0
LB                    0.30 Hz
GB                       0
PC                    1.00
```


^{1}H NMR (500 MHz, DMSO-d_6) of **42**

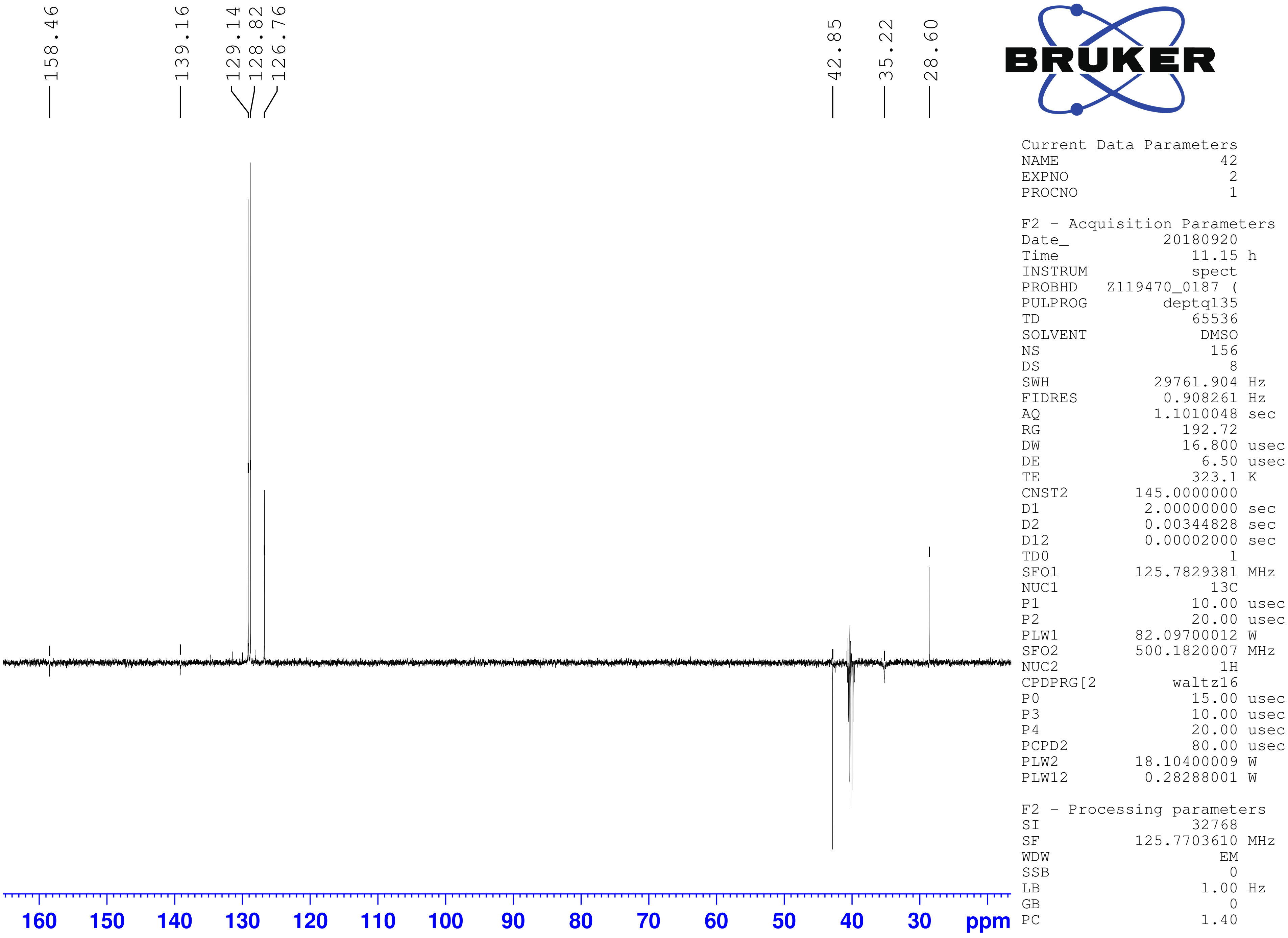

^{13}C (DEPTQ135) NMR (125 MHz, DMSO-d_6) of **42**

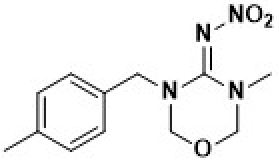

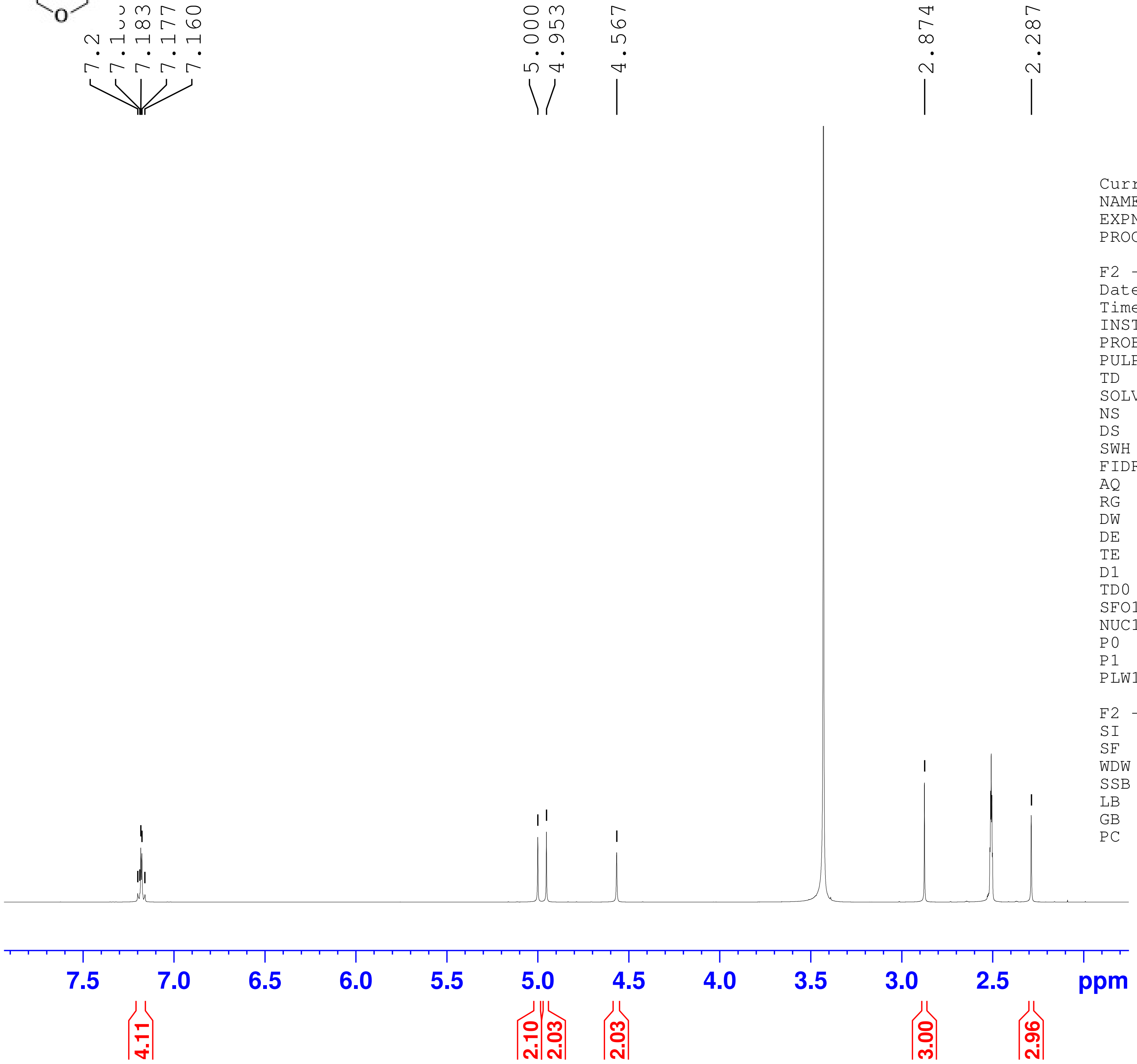

Current Data Parameters	
NAME	43
EXPNO	1
PROCNO	1

F2 - Acquisition Parameters	
Date_	20181126
Time	18.15 h
INSTRUM	spect
PROBHD	Z119470_0187 (
PULPROG	zg30
TD	65536
SOLVENT	DMSO
NS	16
DS	2
SWH	10000.000 Hz
FIDRES	0.305176 Hz
AQ	3.2767999 sec
RG	62.71
DW	50.000 usec
DE	6.50 usec
TE	291.0 K
D1	1.00000000 sec
TD0	1
SFO1	500.1830886 MHz
NUC1	1H
P0	3.33 usec
P1	10.00 usec
PLW1	18.10400009 W

F2 - Processing parameters	
SI	65536
SF	500.1800000 MHz
WDW	EM
SSB	0
LB	0.30 Hz
GB	0
PC	1.00

^{1}H NMR (500 MHz, DMSO-d_6) of **43**

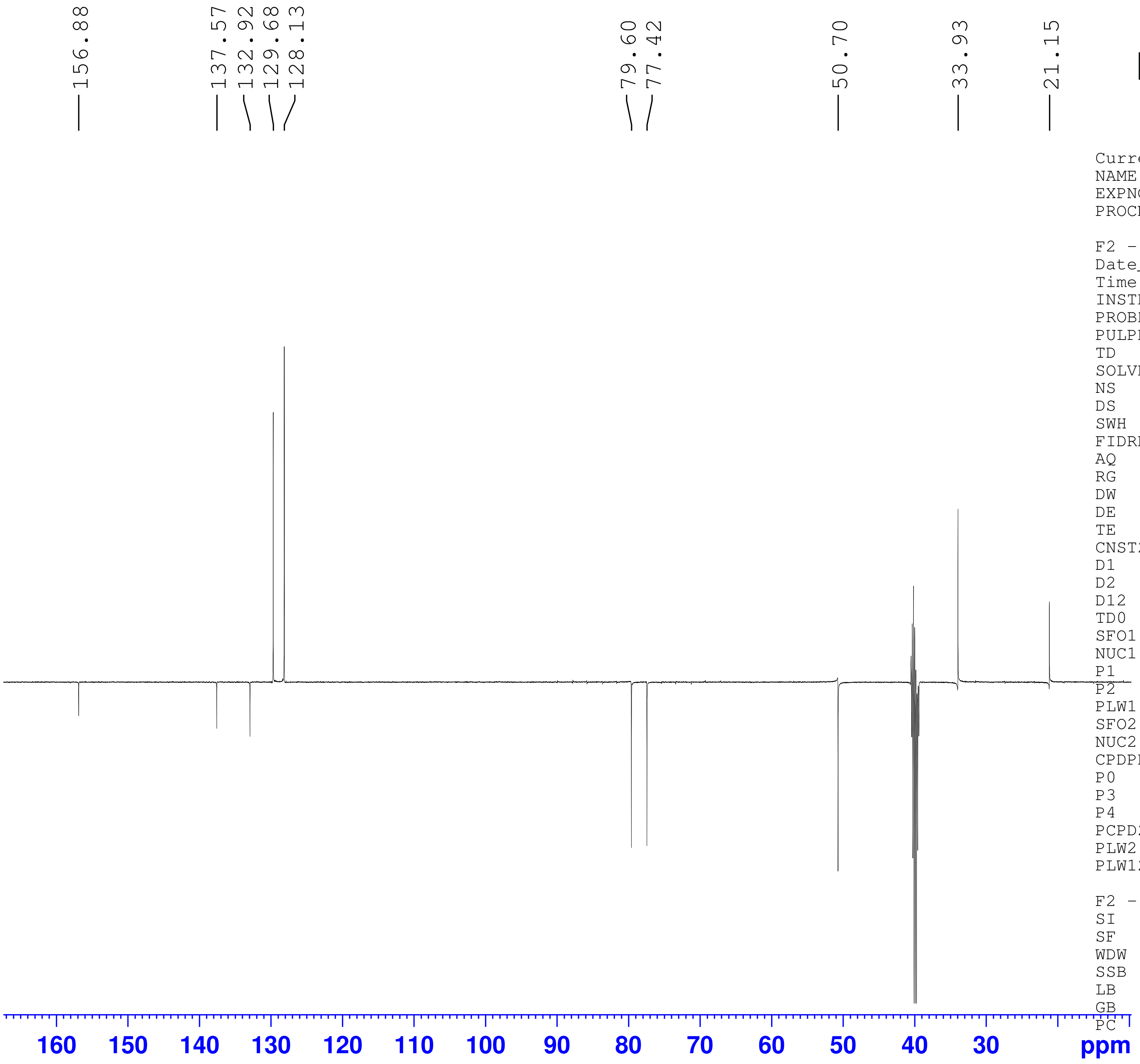


```
Current Data Parameters
NAME                  43
EXPNO                  2
PROCNO                 1

F2 - Acquisition Parameters
Date_           20181128
Time                9.25 h
INSTRUM            spect
PROBHD   Z119470_0187 (
PULPROG        deptq135
TD                 65536
SOLVENT             DMSO
NS                 16720
DS                     8
SWH            29761.904 Hz
FIDRES          0.908261 Hz
AQ             1.1010048 sec
RG                192.72
DW                16.800 usec
DE                  6.50 usec
TE                 291.7 K
CNST2        145.0000000
D1            2.00000000 sec
D2            0.00344828 sec
D12           0.00002000 sec
TD0                    1
SFO1         125.7829381 MHz
NUC1                 13C
P1                 10.00 usec
P2                 20.00 usec
PLW1         82.09700012 W
SFO2         500.1820007 MHz
NUC2                  1H
CPDPRG[2         waltz16
P0                 15.00 usec
P3                 10.00 usec
P4                 20.00 usec
PCPD2              80.00 usec
PLW2         18.10400009 W
PLW12         0.28288001 W

F2 - Processing parameters
SI                 32768
SF           125.7703610 MHz
WDW                   EM
SSB                    0
LB                  1.00 Hz
GB                     0
PC                  1.40
```

^{13}C (DEPTQ135) NMR (125 MHz, DMSO-d_6) of **43**

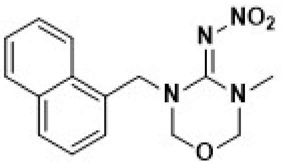

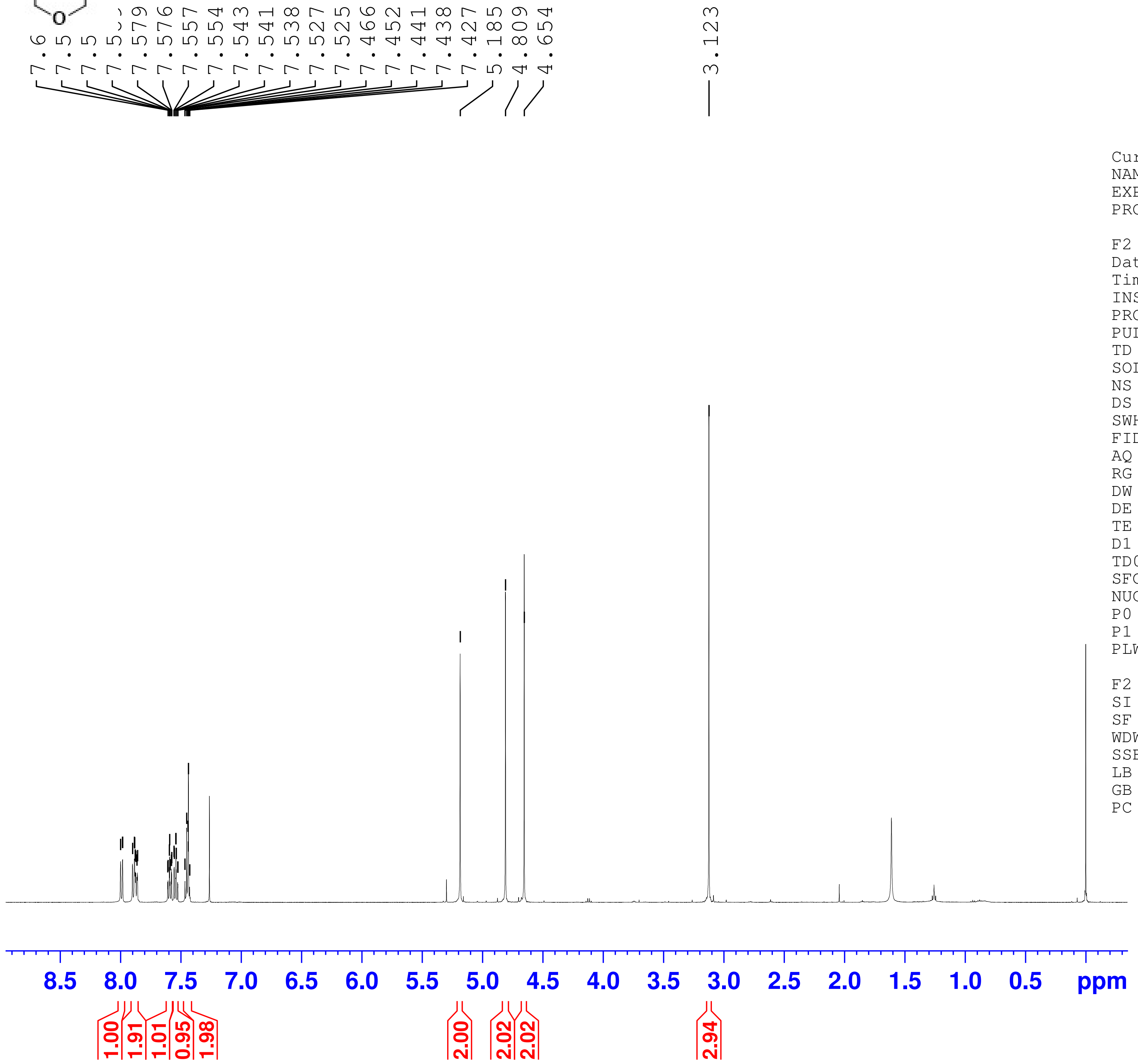


```
Current Data Parameters
NAME                  44
EXPNO                  1
PROCNO                 1

F2 - Acquisition Parameters
Date_           20190613
Time               18.37 h
INSTRUM            spect
PROBHD   Z119470_0187 (
PULPROG             zg30
TD                 65536
SOLVENT            CDCl3
NS                     5
DS                     2
SWH            10000.000 Hz
FIDRES          0.305176 Hz
AQ             3.2767999 sec
RG                139.09
DW                50.000 usec
DE                  6.50 usec
TE                 298.0 K
D1            1.00000000 sec
TD0                    1
SFO1         500.1830886 MHz
NUC1                  1H
P0                  3.49 usec
P1                 10.47 usec
PLW1         18.10400009 W

F2 - Processing parameters
SI                 65536
SF           500.1800104 MHz
WDW                   EM
SSB                    0
LB                  0.30 Hz
GB                     0
PC                  1.00
```


^{1}H NMR (500 MHz, $CDCl_3$) of **44**

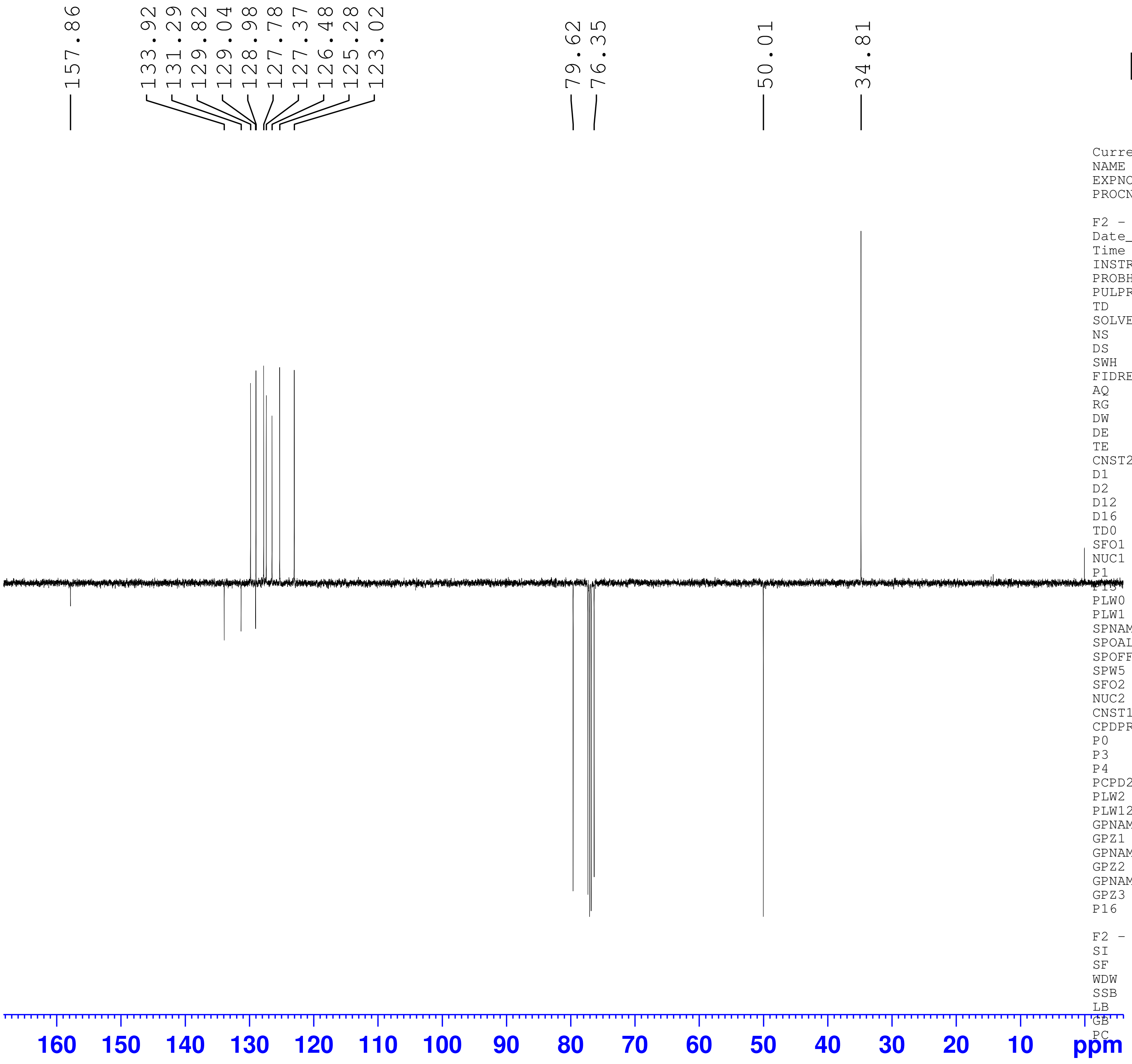

Current Data Parameters		
NAME	44	
EXPNO	2	
PROCNO	1	
F2 - Acquisition Parameters		
Date_	20190620	
Time	18.54	h
INSTRUM	spect	
PROBHD	Z119470_0187 (	
PULPROG	deptqgpsp	
TD	65536	
SOLVENT	CDCl3	
NS	1024	
DS	8	
SWH	29761.904	Hz
FIDRES	0.908261	Hz
AQ	1.1010048	sec
RG	192.72	
DW	16.800	usec
DE	6.50	usec
TE	291.1	K
CNST2	145.0000000	
D1	2.00000000	sec
D2	0.00344828	sec
D12	0.00002000	sec
D16	0.00020000	sec
TD0	1	
SFO1	125.7829381	MHz
NUC1	13C	
P1	10.00	usec
P13	2000.00	usec
PLW0	0	W
PLW1	82.09700012	W
SPNAM[5]	Crp60comp.4	
SPOAL5	0.500	
SPOFFS5	0	Hz
SPW5	12.54300022	W
SFO2	500.1820007	MHz
NUC2	1H	
CNST12	1.5000000	
CPDPRG[2	waltz65	
P0	15.70	usec
P3	10.47	usec
P4	20.94	usec
PCPD2	80.00	usec
PLW2	18.10400009	W
PLW12	0.31009001	W
GPNAM[1]	SMSQ10.100	
GPZ1	31.00	%
GPNAM[2]	SMSQ10.100	
GPZ2	31.00	%
GPNAM[3]	SMSQ10.100	
GPZ3	31.00	%
P16	1000.00	usec
F2 - Processing parameters		
SI	32768	
SF	125.7703610	MHz
WDW	EM	
SSB	0	
LB	1.00	Hz
GB	0	
PC	1.40	

^{13}C (DEPTQ135) NMR (125 MHz, $CDCl_3$) of **44**

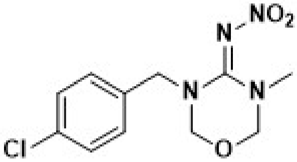

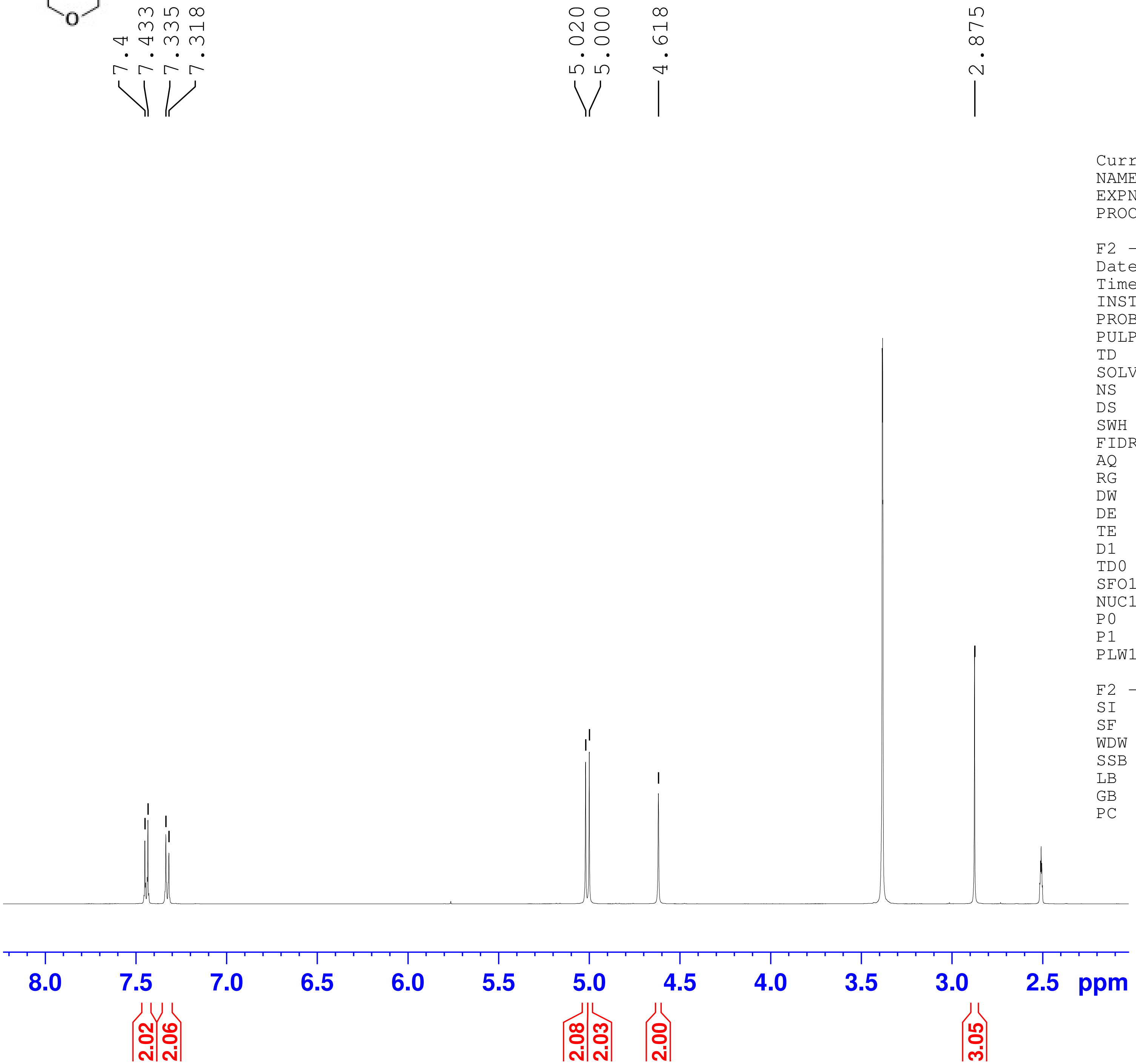

Current Data Parameters
NAME 45
EXPNO 1
PROCNO 1

F2 - Acquisition Parameters
Date_ 20190115
Time 10.26 h
INSTRUM spect
PROBHD Z119470_0187 (
PULPROG zg30
TD 65536
SOLVENT DMSO
NS 16
DS 2
SWH 10000.000 Hz
FIDRES 0.305176 Hz
AQ 3.2767999 sec
RG 95.16
DW 50.000 usec
DE 6.50 usec
TE 291.4 K
D1 1.00000000 sec
TD0 1
SFO1 500.1830886 MHz
NUC1 1H
P0 3.33 usec
P1 10.00 usec
PLW1 18.10400009 W

F2 - Processing parameters
SI 65536
SF 500.1800000 MHz
WDW EM
SSB 0
LB 0.30 Hz
GB 0
PC 1.00

^{1}H NMR (500 MHz, DMSO-d_6) of **45**

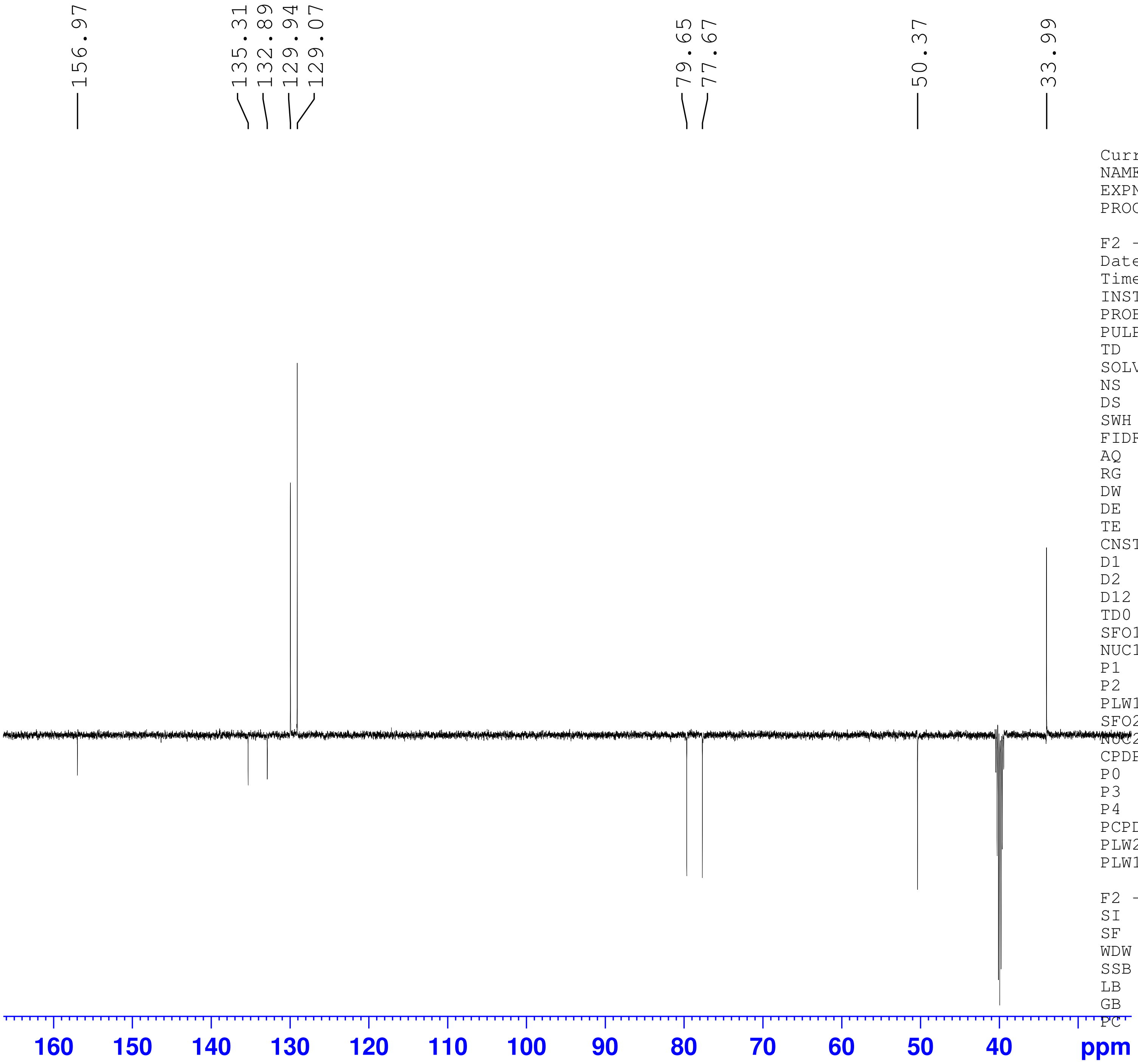

Current Data Parameters
NAME 45
EXPNO 2
PROCNO 1

F2 - Acquisition Parameters
Date_ 20190115
Time 10.33 h
INSTRUM spect
PROBHD Z119470_0187 (
PULPROG deptq135
TD 65536
SOLVENT DMSO
NS 116
DS 8
SWH 29761.904 Hz
FIDRES 0.908261 Hz
AQ 1.1010048 sec
RG 192.72
DW 16.800 usec
DE 6.50 usec
TE 291.8 K
CNST2 145.0000000
D1 2.00000000 sec
D2 0.00344828 sec
D12 0.00002000 sec
TD0 1
SFO1 125.7829381 MHz
NUC1 13C
P1 10.00 usec
P2 20.00 usec
PLW1 82.09700012 W
SFO2 500.1820007 MHz
NUC2 1H
CPDPRG[2 waltz16
P0 15.00 usec
P3 10.00 usec
P4 20.00 usec
PCPD2 80.00 usec
PLW2 18.10400009 W
PLW12 0.28288001 W

F2 - Processing parameters
SI 32768
SF 125.7703610 MHz
WDW EM
SSB 0
LB 1.00 Hz
GB 0
PC 1.40

^{13}C (DEPTQ135) NMR (125 MHz, DMSO-d_6) of **45**

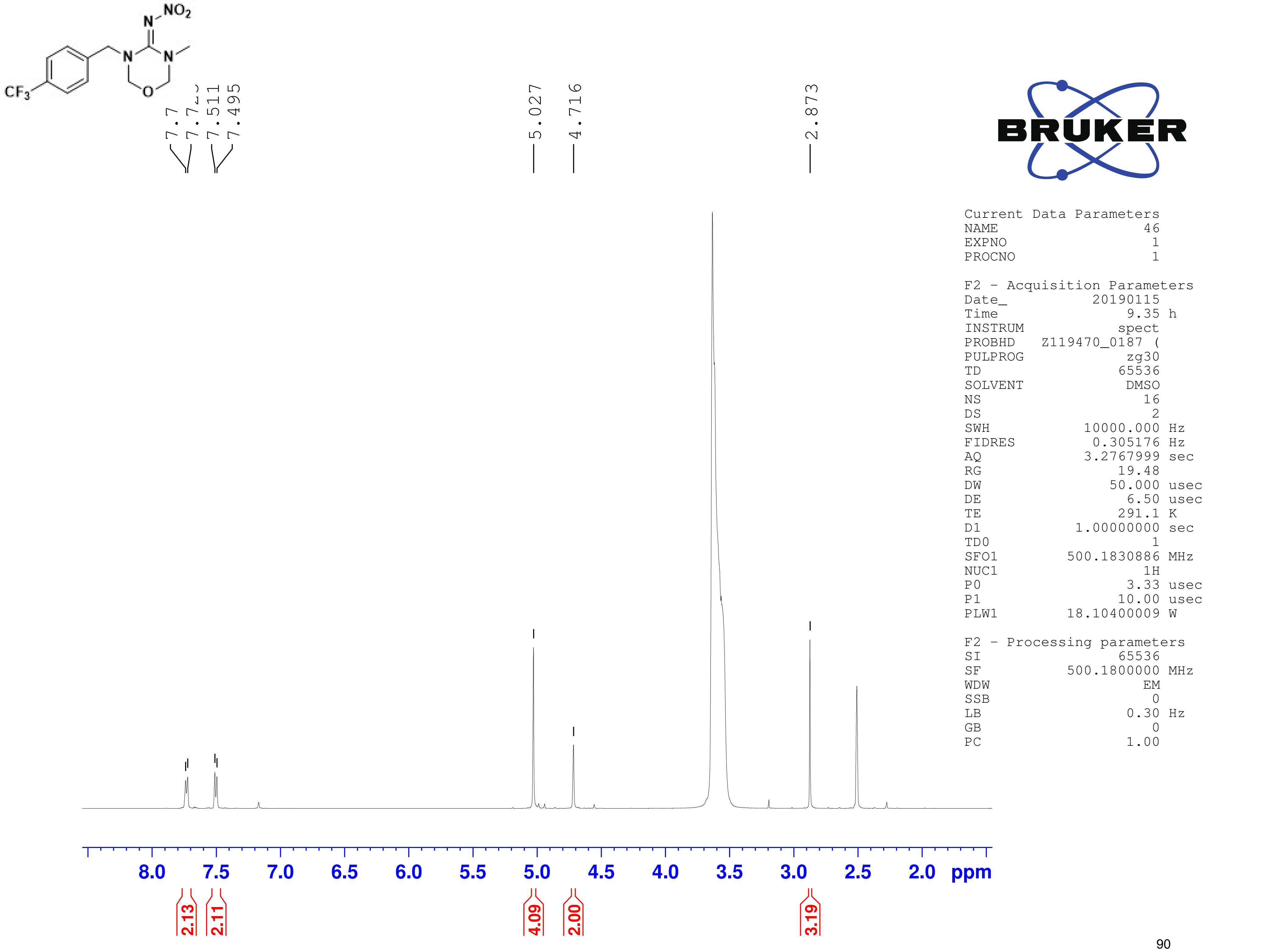

^{1}H NMR (500 MHz, DMSO-d_6) of **46**

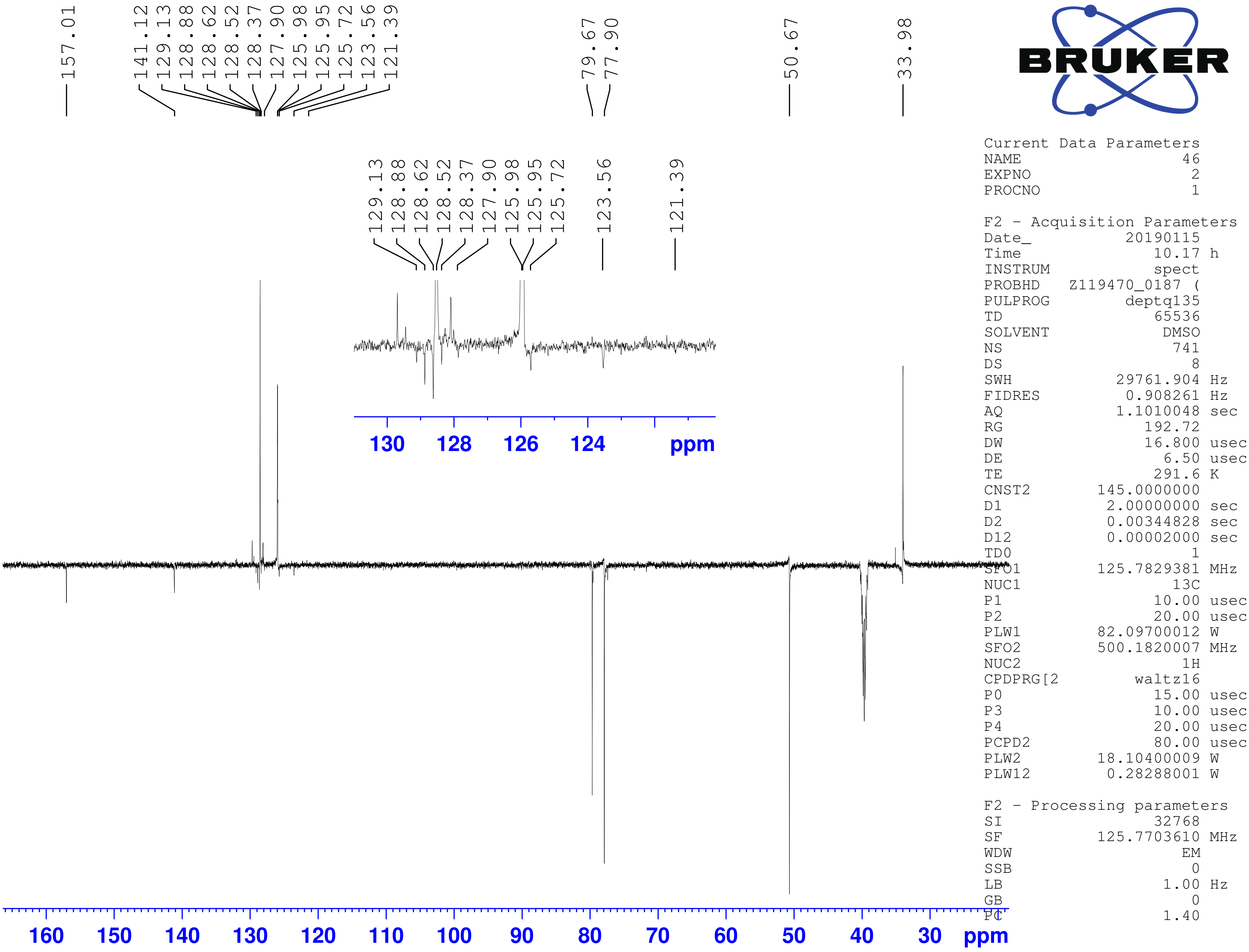

^{13}C (DEPTQ135) NMR (125 MHz, DMSO-d_6) of **46**

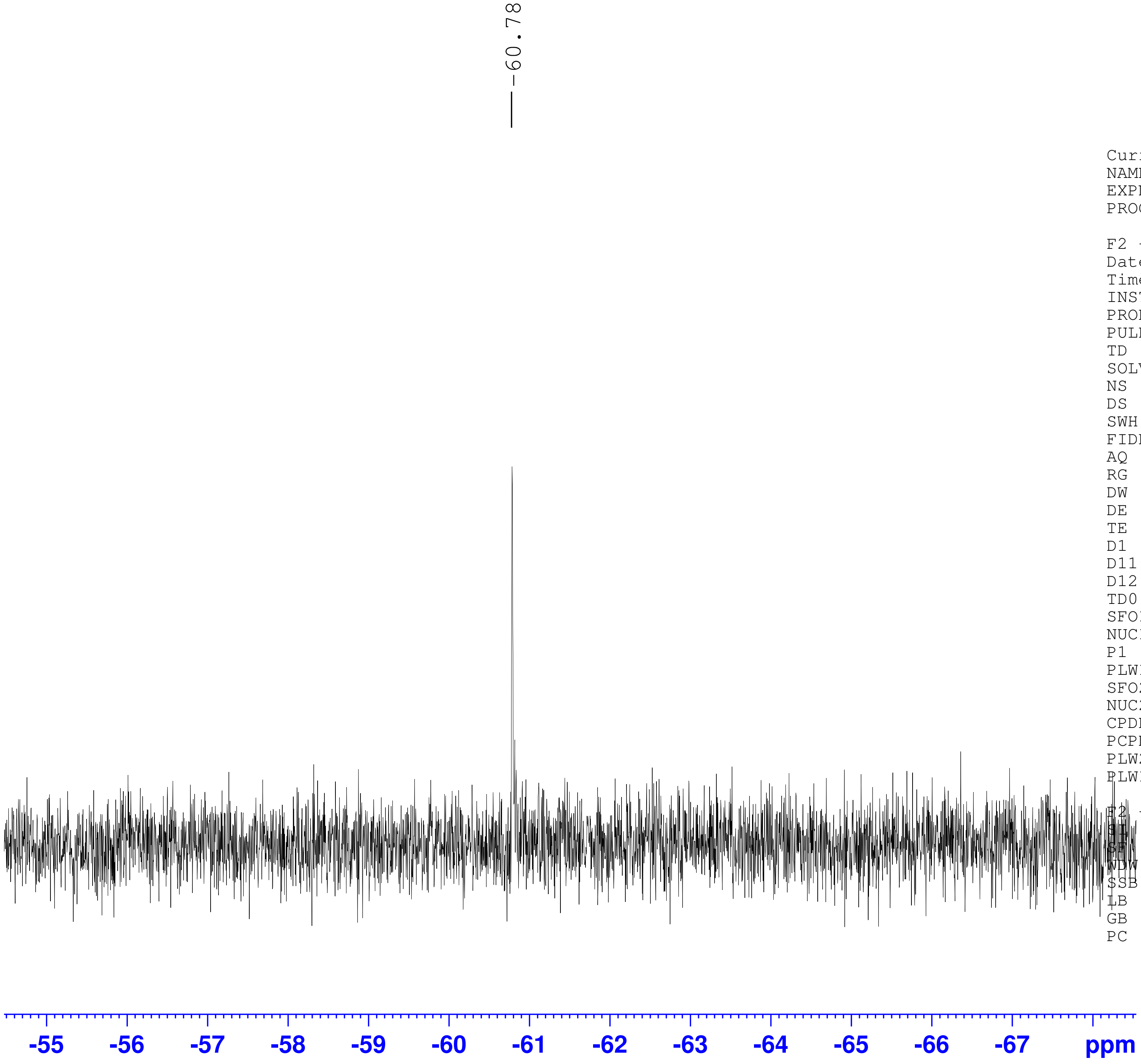

Current Data Parameters

NAME	46
EXPNO	3
PROCNO	1

F2 - Acquisition Parameters

Date_	20190801
Time	14.40 h
INSTRUM	spect
PROBHD	Z119470_0187 (
PULPROG	zgfhigqn.2
TD	131072
SOLVENT	DMSO
NS	16
DS	4
SWH	113636.367 Hz
FIDRES	1.733953 Hz
AQ	0.5767168 sec
RG	11.27
DW	4.400 usec
DE	6.50 usec
TE	293.4 K
D1	1.00000000 sec
D11	0.03000000 sec
D12	0.00002000 sec
TD0	1
SFO1	470.5923603 MHz
NUC1	19F
P1	16.00 usec
PLW1	36.93899918 W
SFO2	500.1820007 MHz
NUC2	1H
CPDPRG[2	waltz16
PCPD2	80.00 usec
PLW2	18.10400009 W
PLW12	0.31009001 W

F2 - Processing parameters

SI	65536
SF	470.6394242 MHz
WDW	EM
SSB	0
LB	0.30 Hz
GB	0
PC	1.00

^{19}F NMR (470 MHz, DMSO-d_6) of **46**

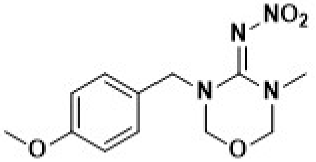

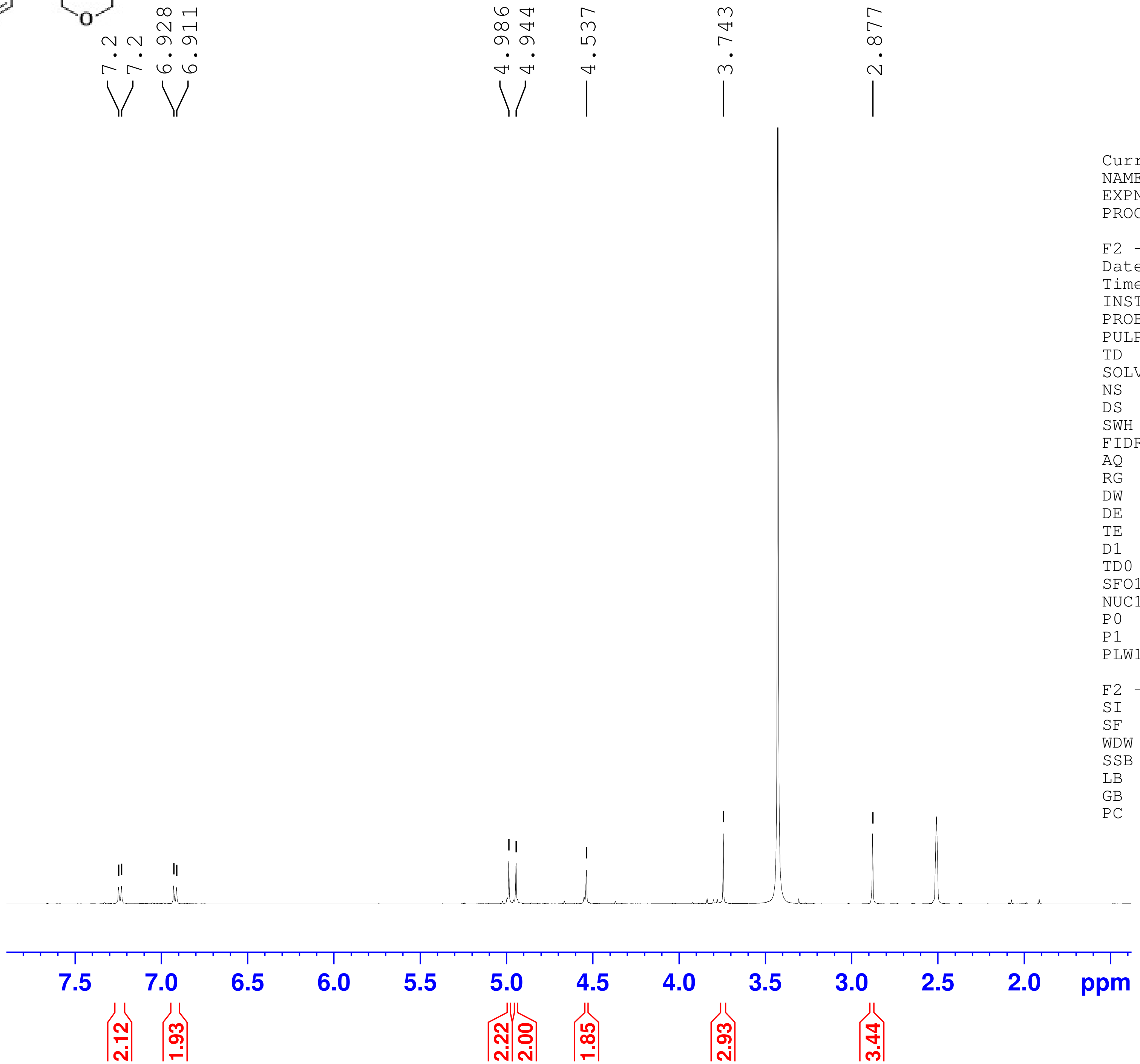

Current Data Parameters

NAME	47
EXPNO	1
PROCNO	1

F2 - Acquisition Parameters

Date_	20190611
Time	18.00 h
INSTRUM	spect
PROBHD	Z119470_0187 (
PULPROG	zg30
TD	65536
SOLVENT	DMSO
NS	16
DS	2
SWH	10000.000 Hz
FIDRES	0.305176 Hz
AQ	3.2767999 sec
RG	48.27
DW	50.000 usec
DE	6.50 usec
TE	298.0 K
D1	1.00000000 sec
TD0	1
SFO1	500.1830886 MHz
NUC1	1H
P0	3.49 usec
P1	10.47 usec
PLW1	18.10400009 W

F2 - Processing parameters

SI	65536
SF	500.1800000 MHz
WDW	EM
SSB	0
LB	0.30 Hz
GB	0
PC	1.00

^1H NMR (500 MHz, DMSO-d_6) of **47**

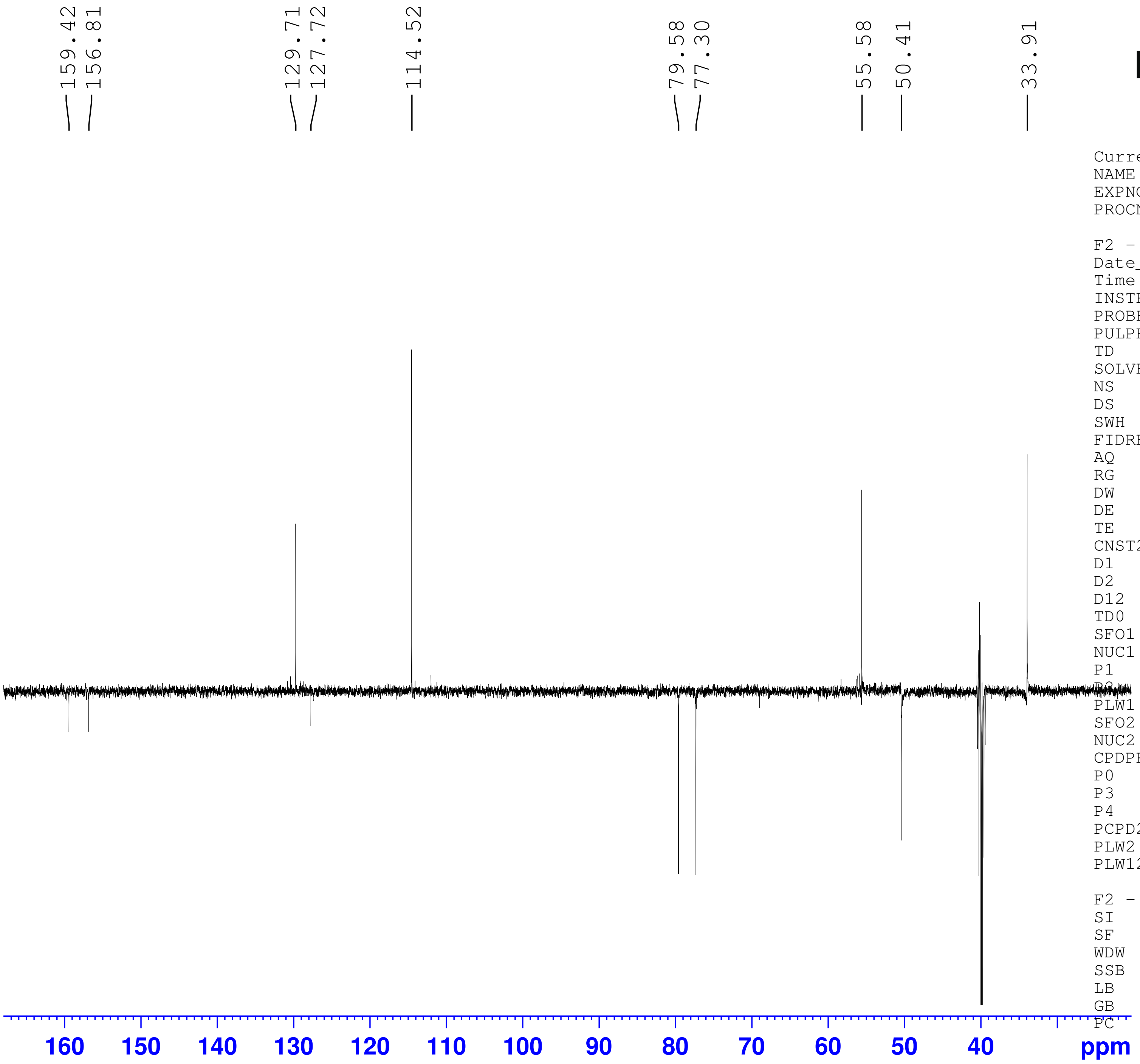


```
Current Data Parameters
NAME                    47
EXPNO                    3
PROCNO                   1

F2 - Acquisition Parameters
Date_             20190611
Time                 18.18 h
INSTRUM              spect
PROBHD     Z119470_0187 (
PULPROG          deptq135
TD                   65536
SOLVENT               DMSO
NS                     200
DS                       8
SWH              29761.904 Hz
FIDRES            0.908261 Hz
AQ               1.1010048 sec
RG                  192.72
DW                  16.800 usec
DE                    6.50 usec
TE                   298.2 K
CNST2        145.0000000
D1              2.00000000 sec
D2              0.00344828 sec
D12             0.00002000 sec
TD0                      1
SFO1        125.7829381 MHz
NUC1                   13C
P1                   10.00 usec
P2                   20.00 usec
PLW1           82.09700012 W
SFO2        500.1820007 MHz
NUC2                    1H
CPDPRG[2           waltz16
P0                   15.70 usec
P3                   10.47 usec
P4                   20.94 usec
PCPD2                80.00 usec
PLW2           18.10400009 W
PLW12           0.31009001 W

F2 - Processing parameters
SI                   32768
SF          125.7703610 MHz
WDW                     EM
SSB                      0
LB                    1.00 Hz
GB                       0
PC                    1.40
```

^{13}C (DEPTQ135) NMR (125 MHz, DMSO-d_6) of **47**
